# Both Genetic Manipulation and Acupuncture Alleviate Depressive‐Like Behavior Mediated by Astrocytic P2X7 Receptors

**DOI:** 10.1002/mco2.71042

**Published:** 2026-10-04

**Authors:** Wen‐Jing Ren, Muhammad Tahir Khan, Xing‐Ying Ren, Xuan Li, Patrizia Rubini, Hai‐Yan Yin, Alexei Verkhratsky, Peter Illes, Yong Tang

**Affiliations:** ^1^ International Joint Research Centre on Purinergic Signalling School of Health and Rehabilitation Chengdu University of Traditional Chinese Medicine Chengdu China; ^2^ School of Acupuncture and Tuina Chengdu University of Traditional Chinese Medicine Chengdu China; ^3^ Faculty of Biology, Medicine and Health The University of Manchester Manchester UK; ^4^ Rudolf Boehm Institute for Pharmacology and Toxicology University of Leipzig Leipzig Germany

**Keywords:** astrocytic P2X7 receptors, depressive‐like behavior, electroacupuncture, extracellular ATP, major depressive disorder, neuronal P2X7 receptors

## Abstract

Major depressive disorder (MDD) has been associated with P2X7 receptors (P2X7Rs), yet the contribution of astrocytic P2X7Rs to neuronal dysfunction remains unclear. We investigated the role of astrocytic P2X7Rs in depression‐like behaviors using a chronic unpredictable mild stress (CUMS) mouse model and further examined whether electroacupuncture (EA) exerts antidepressant effects by modulating astrocytic P2X7Rs. Astrocytic P2X7Rs in the medial prefrontal cortex (mPFC) were selectively knocked down or overexpressed via gene manipulation. Depression‐like behaviors were assessed after CUMS exposure and EA intervention, and in vivo fiber photometry was used to monitor calcium dynamics in excitatory and inhibitory neurons within the mPFC. CUMS markedly increased P2X7R expression in the mPFC and induced depression‐like behaviors. Genetic deletion or knockdown of astrocytic P2X7Rs alleviated these abnormalities, whereas overexpression of astrocytic P2X7Rs exacerbated them and diminished the antidepressant efficacy of EA. Fiber photometry revealed that astrocytic P2X7Rs contributed to stress‐induced dysregulation of neuronal calcium activity. EA restored the balance between excitatory and inhibitory neuronal activity in the mPFC, and this effect depended on appropriate regulation of astrocytic P2X7Rs. These findings suggest that astrocytic P2X7Rs are key mediators of stress‐induced depressive phenotypes and may represent a promising therapeutic target for MDD.

## Graphical abstract

The efficacy of the chronic unpredictable mild stress (CUMS) model in mice was assessed using the tail suspension test (TST; A) and the forced swim test (FST; B). In both major depressive disorder (MDD) mouse models, CUMS significantly increased immobility time, indicating the development of depressive‐like behavior. P2X7 receptor (P2X7R) mRNA and protein expression were reduced by injecting P2X7R‐selective shRNA into the medial prefrontal cortex. (C) Representative Western blots and quantification of the P2X7R/GAPDH (glycerinaldehyd‐3‐phosphat‐dehydrogenase protein ratio; mean ± SEM, *n* = 5) demonstrated a significant reduction in P2X7R protein expression compared with mice receiving control shRNA (*p* < 0.05) or GFAP‐selective shRNA. (D) In contrast, injection of shRNA designed to enhance P2X7R expression in the mPFC increased P2X7R protein levels (mean ± SEM, *n* = 6; *p* < 0.01). The respective changes in P2X7R mRNA values accorded with the changes in the P2X7R protein values. Together, these findings demonstrate that astrocytic P2X7Rs prevent the development of CUMS‐induced depressive‐like behavior.

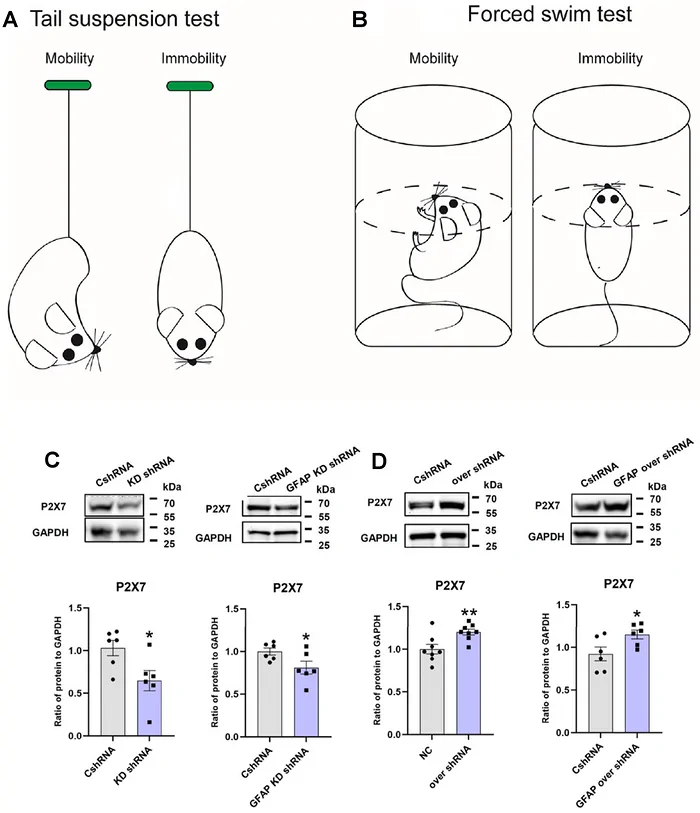

## 1 Introduction

Major depressive disorder (MDD) affects an estimated 300 million people worldwide and is projected to become one of the three leading contributors to the global disease burden by 2030 [1, 2]. MDD is characterized by a wide range of symptoms including depressed mood, anhedonia, feelings of worthlessness, suicidal ideation, impaired interpersonal relationships, and disturbances in sleep and appetite [3].

In the central nervous system (CNS), various purinergic signaling molecules such as adenosine 5′‐triphosphate (ATP) and its enzymatic degradation products ADP, AMP, and adenosine are released into the extracellular space to participate in neuron–neuron and neuron–glia communication [4, 5]. ATP‐sensitive P2X7 receptors (P2X7Rs) are preferentially localized in microglial cells, the resident immunocytes of the brain. They are also present at low densities in astrocytes and oligodendrocytes. However, whether P2X7Rs are functionally expressed in neurons remains a matter of debate [6, 7, 8, 9]. Increasing evidence suggests that aberrant P2X7R signaling contributes to the pathophysiology of several neuropsychiatric disorders, including MDD.

Several studies have implicated P2X7Rs in the development of depressive‐like behaviors. The pharmacological inhibition or genetic deletion of P2X7Rs attenuates stress‐induced behavioral abnormalities in rodents [10, 11, 12, 13]. However, most of these findings have been derived from acute stress paradigms such as the tail suspension test (TST), forced swim test (FST), and foot‐shock‐based models. These acute paradigms do not lead to abnormal adaptive behaviors typical of MDD and are therefore considered to only incompletely model human diseases. Therefore, the role of P2X7Rs in chronic stress‐induced depression remains unclear.

Previous pathological neuronal functions are thought to be the cause of MDD and depression‐like behaviors. Recently, neuroglial cells, which dynamically interact with synapses, have been assumed to orchestrate this pathology via P2X7R‐mediated glia–neuron interaction [14, 15, 16]. Modification of astrocyte function or their decreased presence in stress‐relevant areas of the brain is associated with MDD [17, 18, 19]. In addition, astrocytes secrete numerous gliotransmitters that modulate synaptic connectivity by supporting neurotransmitter metabolism, synaptogenesis, synaptic elimination, and synaptic plasticity. Dysregulation of these astrocytic functions may contribute to depressive‐like behaviors and has been implicated in the pathophysiology of MDD [20, 21]. Among the depression‐related brain regions, the medial prefrontal cortex (mPFC) is critically involved in emotional regulation and is highly susceptible to stress‐induced structural and functional alterations.

The pharmacological management of MDD is relatively limited, with many patients being resistant to traditional antidepressants, and it is important to take their known adverse effects, including nausea, vomiting, tolerance, addiction, excessive sedation, and neurological damage, into consideration [22, 23]. These limitations have prompted an increasing interest in complementary non‐pharmacological interventions for depression. Electroacupuncture (EA), which combines traditional acupuncture with electrical stimulation, has been widely used as an adjunctive therapy for MDD, especially in Asia. The body of clinical evidence from randomized controlled trials, systematic reviews, and meta‐analyses suggests that acupuncture and EA may alleviate depressive symptoms and improve clinical outcomes, either as standalone interventions or in combination with conventional antidepressants [24, 25, 26]. For example, a large pragmatic randomized controlled trial demonstrated significant improvements in depressive symptoms following acupuncture treatment, and recent meta‐analyses further support the efficacy of acupuncture‐assisted therapies in patients with depression [27, 28]. Moreover, acupuncture is generally well tolerated and has been associated with fewer and milder adverse events than pharmacological treatments [24, 25, 26, 29]. Nevertheless, the heterogeneity of clinical studies and limited understanding of the underlying biological mechanisms continue to hinder the broader clinical translation of EA. Therefore, elucidating the neurobiological basis of the EA‐mediated antidepressant effects remains an important research priority. Emerging evidence suggests that purinergic signaling may contribute to the biological effects of acupuncture [30]. However, whether astrocytic P2X7R signaling participates in the antidepressant effects of EA remains unknown.

The aim of this study was twofold. First, we intended to collect evidence for astrocytic, in contrast to neuronal P2X7Rs, as the primary causes of MDD using chronic unpredictable mild stress (CUMS)‐treated mice and astrocyte‐specific small hairpin (sh)RNA. Second, we aimed to clarify whether EA acts as an antidepressant therapy in CUMS by targeting astrocytic P2X7Rs.

## 2 Results

### 2.1 CUMS‐Induced Depressive‐Like Behavior in Mice in an EA‐ and Fluoxetine‐Reversible Manner

In the present study, the FST, TST, sucrose preference test (SPT), and open field test (OFT) were used to evaluate CUMS‐induced depressive‐like behaviors in mice (the timeline of the workflow is shown in Table S1). The antidepressant effects of EA applied to the left Zusanli acupoint (ST36) were compared with those of the selective serotonin reuptake inhibitor fluoxetine (Figure 1). Exposure to CUMS induced significant depressive‐like behaviors that were markedly attenuated by both EA and fluoxetine treatment. Specifically, CUMS significantly increased the immobility time in both the FST and TST, whereas EA and fluoxetine effectively reversed these changes (Figure 1A,B). In contrast, administration of sham EA and phosphate‐buffered saline (PBS) (fluoxetine solvent) failed to exert significant effects. Similarly, sucrose preference, an indicator of anhedonia, was significantly reduced after CUMS exposure, whereas EA treatment restored sucrose preference levels (Figure 1C). Neither sham EA nor PBS administration affected this parameter. In the OFT, CUMS markedly reduced exploratory activity, as reflected by a decrease in total running distance (Figure 1D,E). Both EA and fluoxetine significantly alleviated this reduction, further supporting their antidepressant‐like effects. Moreover, CUMS significantly decreased the time spent in the central zone of an open‐field arena and increased the time spent at the border, consistent with its well‐established anxiogenic effect [31]. EA, but not fluoxetine, significantly reversed this behavioral alteration (Figure 1D,F,G). In addition, rearing reactions measured in the OFT clearly conformed to depressive‐like behavior (Figure 1D,H) [32]. These findings demonstrate that CUMS successfully induced anxiogenic/depressive‐like behaviors in mice, which were effectively ameliorated by EA treatment. The behavioral outcomes observed in the OFT were consistent with those observed in the FST, TST, and SPT.

**FIGURE 1:**
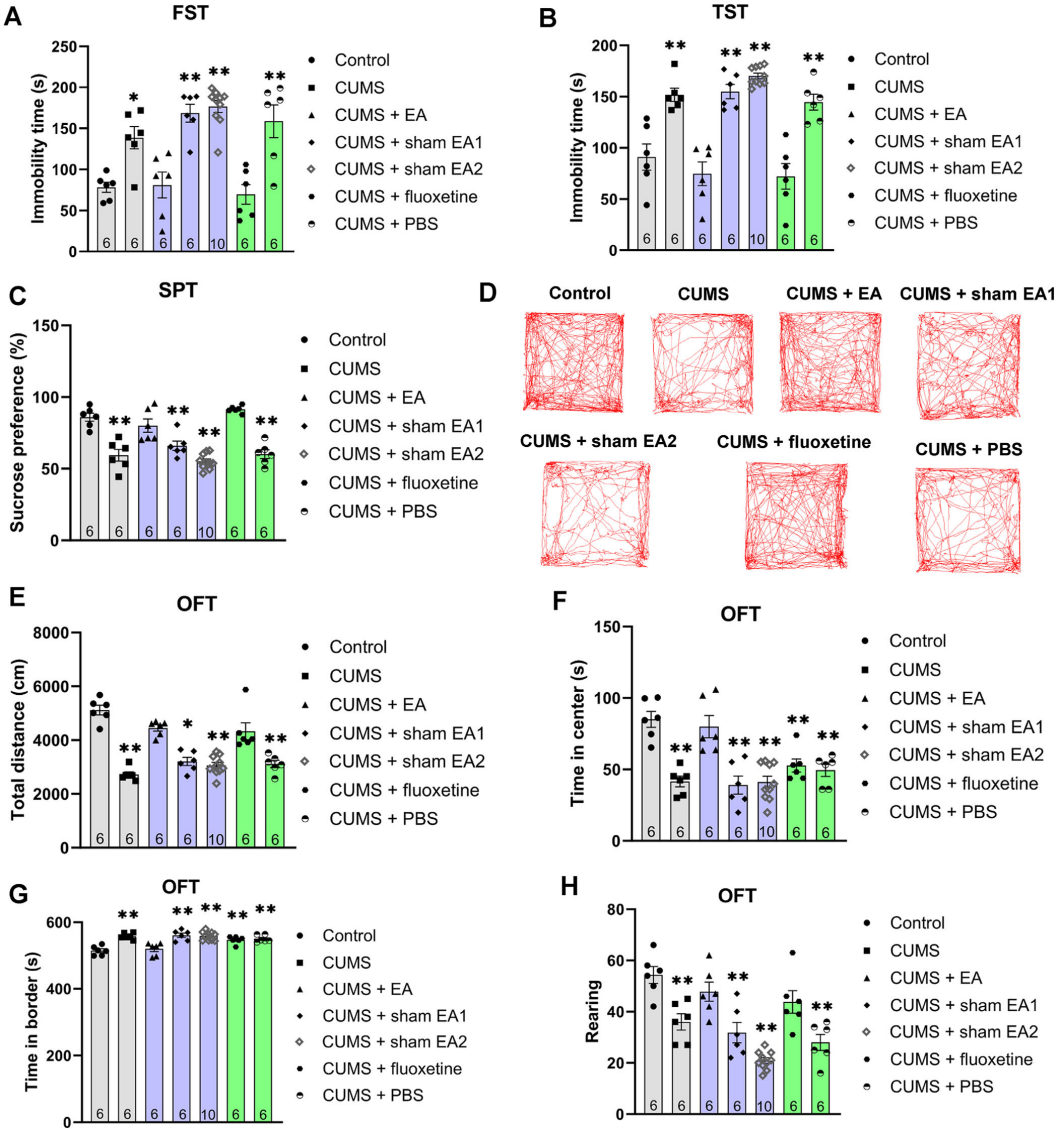
Treatments with electroacupuncture (EA) and fluoxetine prevent chronic unpredictable mild stress (CUMS)‐induced development of depressive‐like behaviors. (A) Forced swim test (FST) immobility time. (B) Tail suspension test (TST) immobility time. (C) Sucrose preference test. (D) Representative running traces in open field test (OFT) during the different treatment regimens; for observation times, see Section 4. (E–H) Average values for the total distance covered (E), time spent in the center (F), time spent in the border (G), and the number of rearing events (H). In the sham EA1 group, the needle was positioned at a non‐acupoint on the tail without electrical stimulation. In the sham EA2 group, a wooden toothpick was placed onto the ST36 acupoint, but was not inserted into it. All data are shown as mean ± SEM. One‐way ANOVA followed by the Holm–Sidak test was used to calculate the levels of statistical significance in case of normal distribution (A–C, F–H). The Kruskal–Wallis test was used to calculate statistical significance in case of non‐normal distribution (E). ^*^ *p* < 0.05, ^**^ *p* < 0.01 compared to the control group. The number of individual animals is indicated in each column.

### 2.2 CUMS Upregulated the Expression of P2X7Rs in the mPFC of Mice in an EA‐Reversible Manner

Subsequently, we determined changes in the expression of P2X7Rs. The number of enzymes participating in the decomposition of ATP/adenosine (CD39 and CD73 [5, 33]), synaptic proteins involved in the exocytotic release of ATP from neurons and astrocytes (SNAP23, SNAP25 [34]), and calcium homeostasis modulator 2 (CALHM2), which serves as one of the plasma membrane channels allowing the non‐exocytotic extrusion of ATP from astrocytes [35], was also measured. As shown in Figure 2A, CUMS exposure significantly increased P2X7R mRNA (“p2x7”) expression in the mPFC compared with the control group. While EA reversed the effect of CUMS on the P2X7R mRNA, sham EA had no comparable effect (Figure 2A). In contrast, CUMS did not significantly affect the mRNA expression of CD39, CD73, SNAP23, SNAP25, or CALHM2 (Figure 2B–F). In addition, the protein levels of P2X7, CD39, CD73, SNAP23, SNAP25, and CALHM2 were measured (Figure 2G–L). Consistent with the P2X7R mRNA expression, P2X7R protein levels were elevated following CUMS treatment (Figure 2G). Similarly, EA reversed the change in P2X7R protein by CUMS, whereas sham EA was ineffective (Figure 2G). CUMS did not alter the protein expression of CD39, CD73, SNAP23, SNAP25, or CALHM2 (Figure 2H–L). These results indicated that the observed increase in P2X7R expression was not accompanied by detectable changes in the expression of proteins involved in ATP degradation or release. Interestingly, the sham EA treatment reduced SNAP23 mRNA expression (Figure 2D). However, this change was not reflected at the protein level, as SNAP23 protein expression remained unchanged (Figure 2J), suggesting the limited biological significance of this finding. Thus, EA selectively reversed the CUMS‐induced increase in P2X7R mRNA and protein in the mPFC, but had no effect on P2X7R mRNA and protein in the mPFC of control mice (Figure 2M,N). These observations are consistent with previous reports, indicating that P2X7Rs are preferentially activated under pathological conditions associated with elevated extracellular ATP concentrations [36, 37].

**FIGURE 2:**
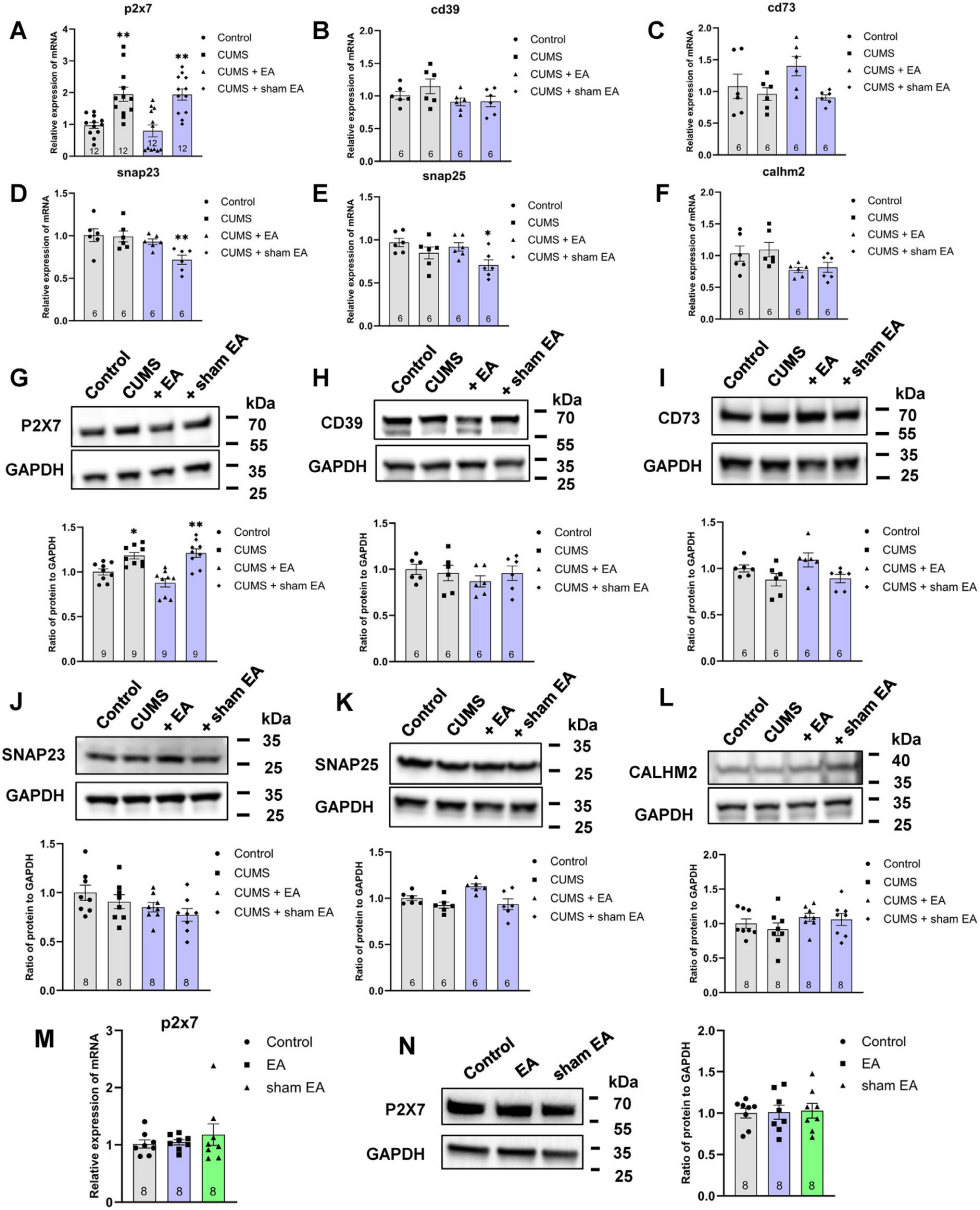
Treatment with EA prevents the CUMS‐induced increase in expression of P2X7Rs. Determination by quantitative (q)PCR or Western blot of mRNA and protein for P2X7Rs, the purine‐metabolizing enzymes CD39 and CD73, the exocytotic vesicular proteins SNAP23 and SNAP25, and the ATP cellular release protein CALHM2 in mPFC brain slices of mice. p2x7, cd39, cd73, snap23, snap25, calhm2, mRNAs for the respective proteins. Determinations were under control conditions in the medial prefrontal cortex (mPFC) prepared from control mice, as well as in the mPFC of mice treated with CUMS either alone or in combination with EA or sham EA. (A–F) Expression of mRNAs as measured by the ΔΔCt method with respect to β‐actin‐RNA. Relative amounts of p2x7r (A), cd39 (B), cd73 (C), snap23 (D), snap25 (E), and calhm2 (F) mRNAs. (G–L) Representative immunoblots and quantification of the proteins for P2X7R (G), CD39 (H), CD73 (I), SNAP23 (J), SNAP25 (K), and CALHM2 (L) as a ratio of the spot of glycerinaldehyd‐3‐phosphat‐dehydrogenase (GAPDH). (M) Expression of p2x7R mRNA in control mice after EA or sham EA1 (see Section 4) treatment. (N) Expression of P2X7 protein in control mice after EA or sham EA1 treatment. All data are shown as mean ± SEM. One‐way ANOVA was followed by the Holm–Sidak test for statistical analysis in case of normal distribution (A–L); ^*^ *p* < 0.05, ^**^ *p* < 0.01 compared to the control group. The number of individual animals is indicated in each column.

Altogether, these results suggest that the upregulation of P2X7R expression is associated with CUMS‐induced depressive‐like behaviors and might contribute to the behavioral alterations observed in Figure 1.

### 2.3 Blockade of Astrocytic, but Not Neuronal P2X7Rs in the mPFC of Mice Reversed the Depressive‐Like Behavior Induced by CUMS Treatment

These findings suggest that P2X7Rs may play a pivotal role in the evolution of depressive‐like behaviors induced by CUMS and prompt further investigation into whether astrocytic or neuronal P2X7Rs contribute to this process.

To verify the cell‐type specificity of the astrocyte‐targeting viral construct, we examined the expression pattern of GFAP‐driven P2X7R knockdown (KD) shRNA. Robust expression of the viral construct was observed in GFAP‐immunoreactive (IR) astrocytes, whereas little or no expression was detected in Iba1‐IR microglia or NeuN‐IR neurons (Figure S1A). Specifically, the green fluorescence signal associated with the GFAP‐driven P2X7R KD shRNA colocalized extensively with the astrocytic marker S100β, but showed no detectable colocalization with the microglial marker Iba1 and only minimal colocalization with the neuronal marker NeuN. Quantitative analysis of the colocalization data confirmed the high specificity of viral expression in astrocytes (Figure S1A). These findings are consistent with those of previous studies using the same viral construct [38].

RT‐PCR analysis confirmed the effectiveness of astrocyte‐specific viral manipulation. Compared to the corresponding control virus (GFAP CshRNA), GFAP‐driven P2X7R KD (GFAP KD shRNA) significantly reduced P2X7R mRNA expression in the mPFC (Figure S1B), whereas the GFAP‐driven P2X7R overexpression construct (GFAP over shRNA) significantly increased P2X7R mRNA expression (Figure S1C). Similarly, downregulation of P2X7Rs in all cellular constituents of the CNS by “KD shRNA” decreased (Figure S1D), and the overexpression of P2X7Rs by “over shRNA” increased (Figure S1E) the P2X7R mRNA. Comparable results were obtained when Western blots were used to determine the quantity of P2X7R protein (Figure S1F–I).

CUMS increased the immobility time in the FST and TST (Figure 3A,B). The prolongation of the duration of behavioral despair was counteracted both by the genetic deletion of P2X7Rs in P2X7R KO mice and by the knockdown of P2X7Rs with astrocyte‐specific (GFAP‐containing) KD shRNA (Figure 3A,B). In contrast, the inactive control shRNA raised against astrocytic P2X7Rs did not alter the duration of behavioral despair in CUMS‐treated mice and was ineffective in normal mice. As shown in Figure 1C, sucrose preference decreased after CUMS treatment (Figure 3C). Both the genetic deletion of P2X7Rs and astrocyte‐specific knockdown abolished this effect. The respective control shRNAs failed to induce any changes in sucrose preference in CUMS‐untreated or CUMS‐treated mice.

**FIGURE 3:**
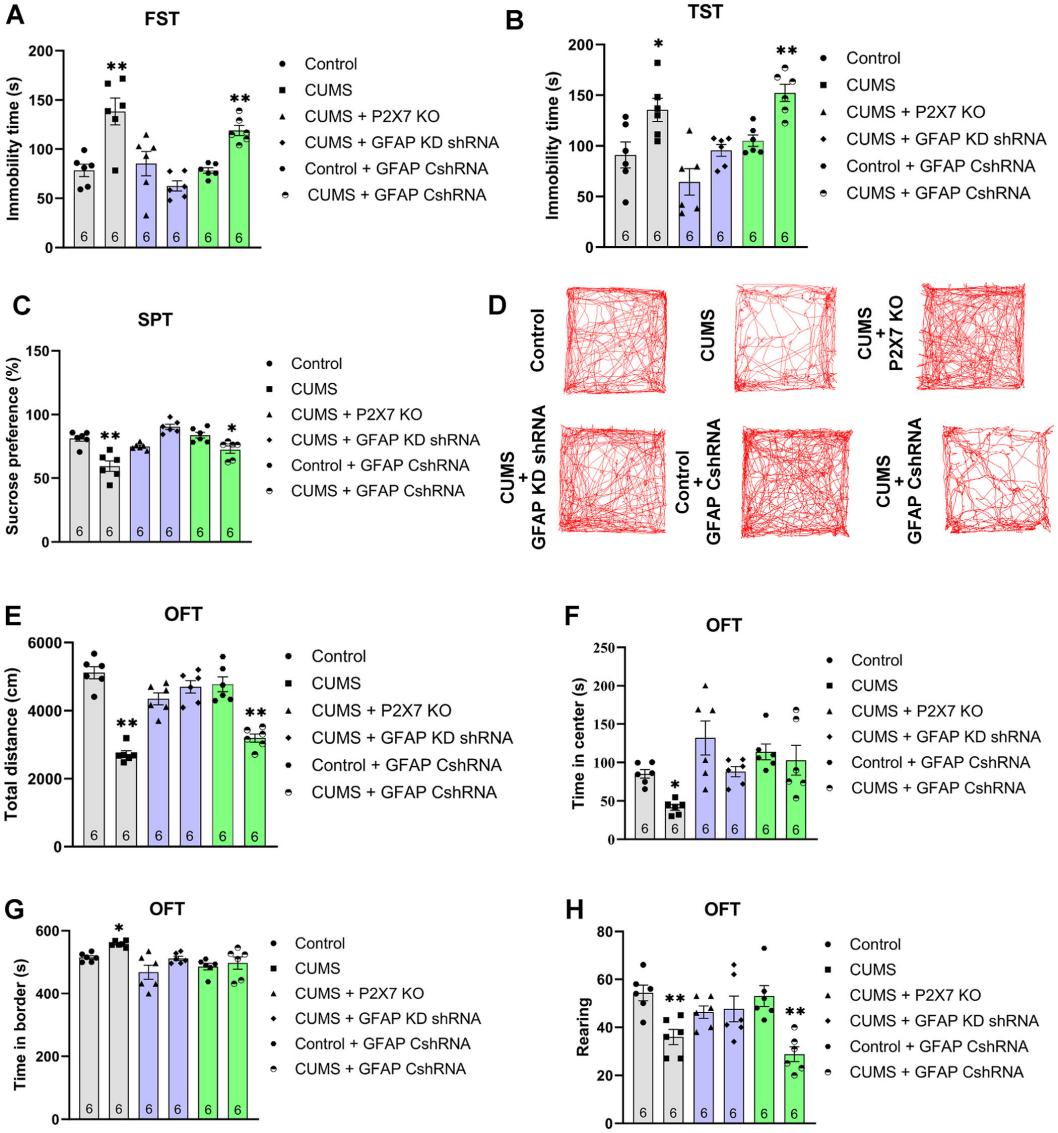
Downregulation of astrocytic P2X7Rs prevents CUMS‐induced development of depressive‐like behaviors. (A) FST immobility time. (B) TST immobility time. (C) Sucrose preference test. (D) Representative running traces in open field test (OFT) during the different treatment regimens; for observation times, see Section 4. (E–H) Average values for the total distance covered (E), time spent in the center (F), time spent in the border (G), and the number of rearing events (H). All data are shown as mean ± SEM. Two‐way ANOVA was followed by Tukey's test for statistical analysis (A–H); ^*^ *p* < 0.05, ^**^ *p* < 0.01 compared to the control group. The number of individual animals is indicated in each column.

The running distance covered by the mice was decreased by CUMS treatment and was reversed by the general deletion of P2X7Rs in KO animals and by the selective knockdown of P2X7Rs in astrocytes (Figure 3D,E). As expected, the inactive control shRNA with astrocyte‐specificity did not act in either CUMS‐treated mice or their untreated counterparts. The time spent in the center decreased, time spent in the border increased, and the number of rearings decreased by CUMS (Figure 3F–H). All three effects were abolished in P2X7R KO mice and astrocyte‐specific KD shRNA‐injected mice (Figure 3F–H). However, only the number of rearings appeared to be a reliable measure of anxiogenic state (Figure 3H, but not Figure 3F) because the control shRNA failed to alter the CUMS‐induced reduction in this case.

In contrast to the findings observed with astrocyte‐specific shRNA that knocked down P2X7Rs, the neuron‐specific (Syn‐containing) shRNA variant did not modify the CUMS‐induced increase in immobility in the FST and TST (Figure 4A,B). Correspondingly, the inactive control version of the shRNA had no effect on the prolongation of immobility time caused by CUMS. Comparable results were obtained with neuron‐specific P2X7R shRNA in the SPT (Figure 4C). The OFT measurements also failed to document the effects of the neuronal shRNA variants on any of the parameters measured in these experiments (Figure 4D–H).

**FIGURE 4:**
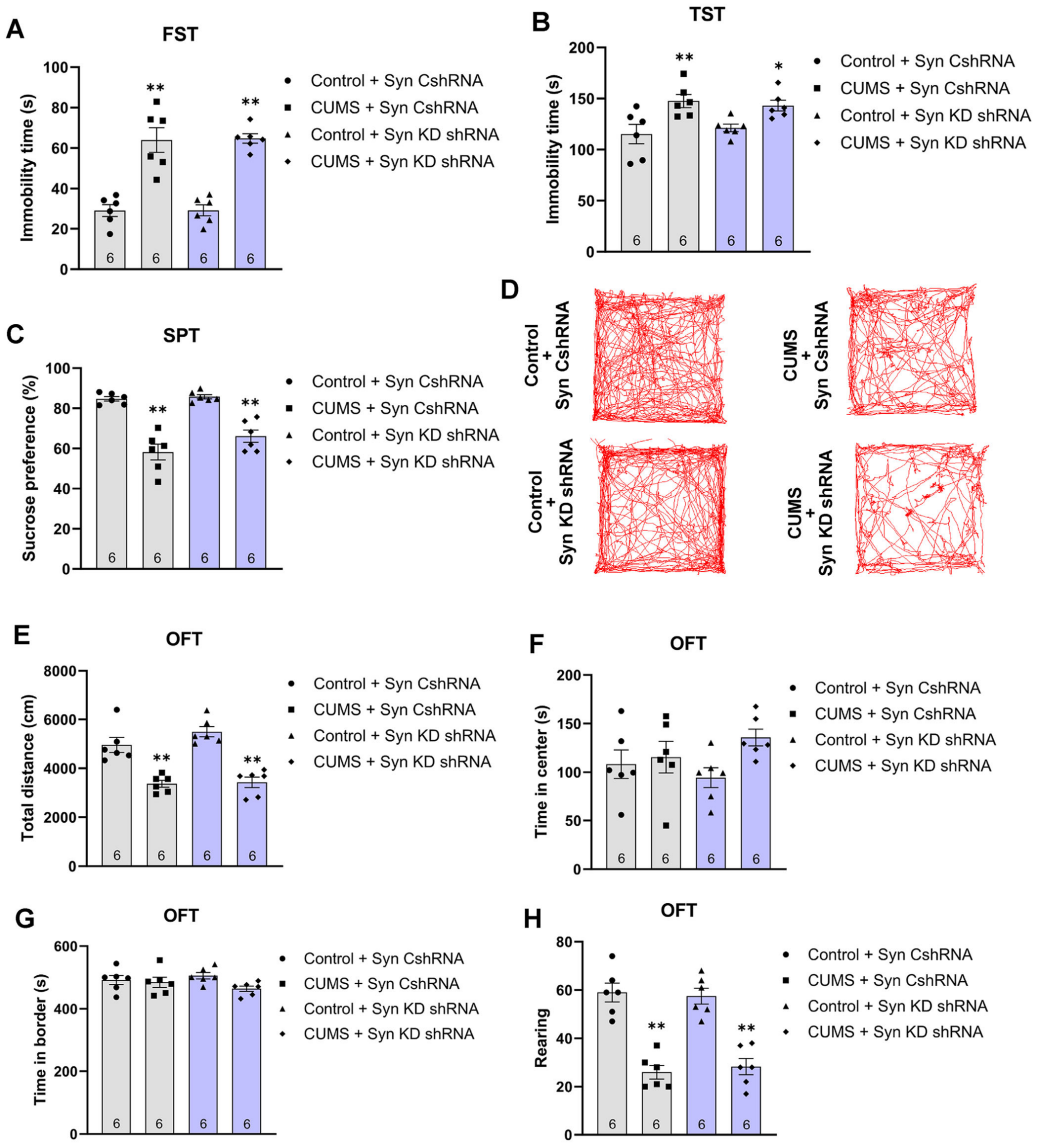
Downregulation of neuronal P2X7Rs has no effect on CUMS‐induced development of depressive‐like behaviors. (A) FST immobility time. (B) TST immobility time. (C) Sucrose preference test. (D) Representative running traces in open field test (OFT) during the different treatment regimens; for observation times, see Section 4. (E–H) Average values for the total distance covered (E), time spent in the center (F), time spent in the border (G), and the number of rearing events (H). All data are shown as mean ± SEM. One‐way ANOVA was followed by the Holm–Sidak test for statistical analysis (A–C, E–H); ^*^ *p* < 0.05, ^**^ *p* < 0.01 compared to the control group. The number of individual animals is indicated in each column.

### 2.4 The Antidepressant Effect of EA Was Reversed After Overexpression of Astrocytic P2X7Rs in the mPFC

After observing that both P2X7R knockout/knockdown and EA counteracted the effect of CUMS and to determine whether P2X7R overexpression would interfere with depressive‐like behavior, mice received a viral construct inducing astrocyte‐specific overexpression of P2X7Rs (GFAP over shRNA) or the corresponding control virus (GFAP over CshRNA) (Figure 5). After overexpression of astrocytic P2X7Rs, the effectiveness of EA on depression‐like symptoms in FST, TST, SPT, and OFT was eliminated (Figure 5A–C). At the same time, the control virus complex had no effect on either the CUMS‐untreated or CUMS‐treated animals in the FST, TST, and SPT. Similarly, the OFT results were consistent with those shown in Figure 3D–H. Astrocytic P2X7R overexpression prevented the EA‐induced restoration of exploratory and anxiety‐related behaviors, whereas the control virus had no detectable effect (Figure 5D–H).

**FIGURE 5:**
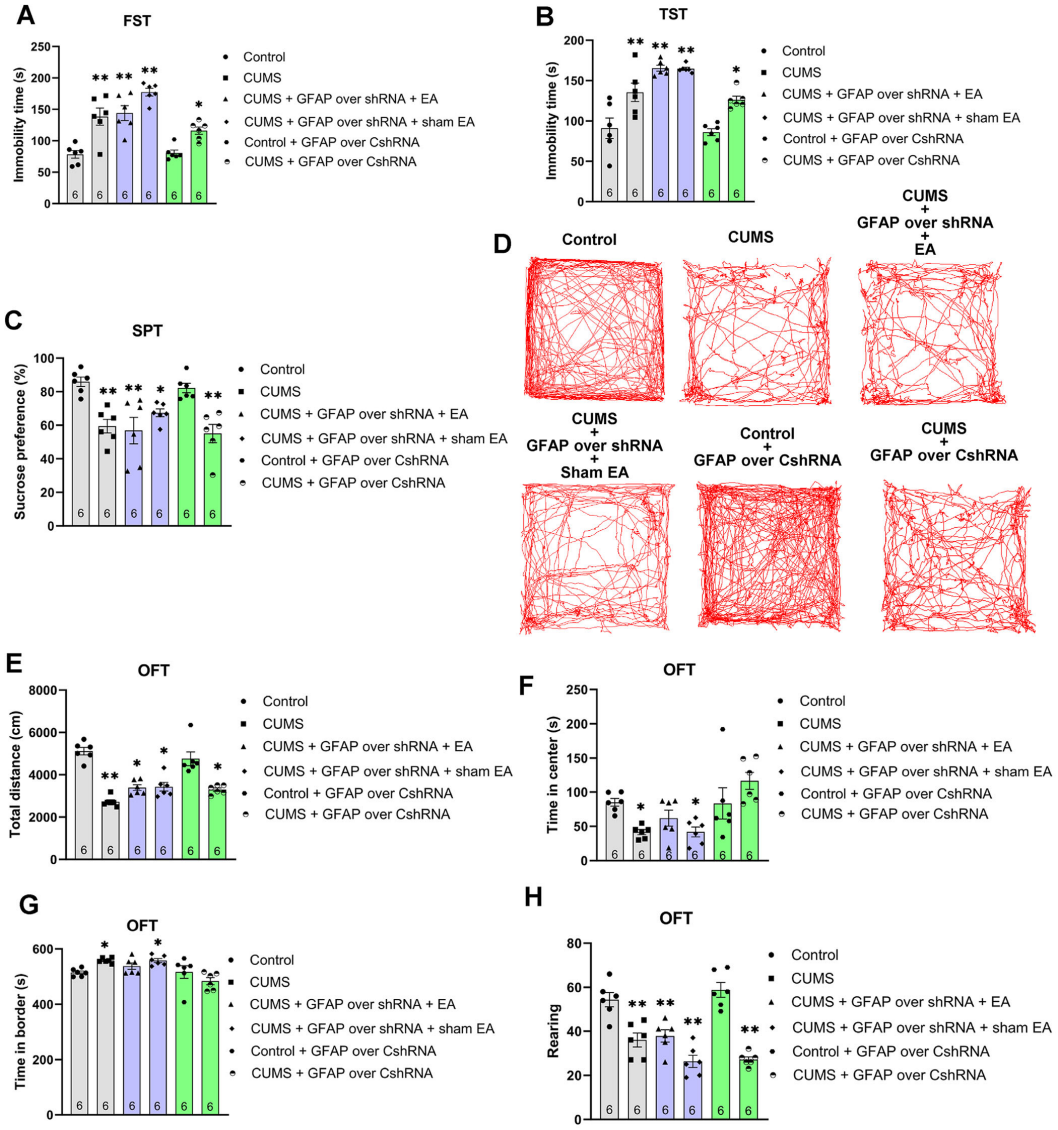
The therapeutic effect of EA on CUMS‐induced depressive‐like behaviors was reversed by overexpression of astrocytic P2X7Rs. (A) FST immobility time. (B) TST immobility time. (C) Sucrose preference test. (D) Representative running traces in open field test (OFT) during the different treatment regimens; for observation times, see Section 4. (E–H) Average values for the total distance covered (E), time spent in the center (F), time spent in the border (G), and the number of rearing events (H). All data are shown as mean ± SEM. One‐way ANOVA followed by the Holm–Sidak test was used to calculate the levels of statistical significance in case of normal distribution (A–C, H). The Kruskal–Wallis test was used to calculate statistical significance in case of non‐normal distribution (E–G). ^*^ *p* < 0.05, ^**^ *p* < 0.01 compared to the control group. The number of individual animals is indicated in each column.

### 2.5 Astrocytic P2X7Rs Regulate Intracellular Ca^2+^ Signals in Excitatory and Inhibitory Neurons During the Depressive‐Like State and Its Reversal by EA

Astrocytes communicate with excitatory (most likely glutamatergic) and inhibitory (most likely GABAergic) neurons to regulate depression‐like neuronal reactions. To investigate this possibility, we expressed genetically encoded calcium indicators in excitatory and inhibitory neuronal populations in the mPFC and monitored the intracellular Ca^2+^ ([Ca^2+^]_i_) dynamics using fiber photometry. Calcium signals were recorded 2 weeks after viral injection. As shown in Figure 6A–D, TST and foot shock, known to cause depressive‐like/anhedonic behavior, decreased [Ca^2+^]_i_ signaling in excitatory neurons but increased it in inhibitory neurons. Overexpression of P2X7Rs in astrocytes induced a reduction in excitatory neuronal activity during the TST and foot shock (Figure 6A,C), indicating the prolongation of behavioral despair. In contrast, the same change in P2X7R‐density induced an increase in inhibitory neuronal activity during the TST and foot shock (Figure 6B,D), leading to an identical behavioral response. Genetic deletion of P2X7Rs in astrocytes had a clear‐cut opposite effect only on the TST‐induced increase in [Ca^2+^]_i_ in excitatory neurons (Figure 6A) but failed to alter the TST‐induced [Ca^2+^]_i_ responses in inhibitory neurons (Figure 6B) or in both types of neuronal populations during foot shock stimulation (Figure 6C,D). Taken together, these findings suggest that astrocytic P2X7Rs contribute to stress‐induced alterations in excitatory and inhibitory neuronal activity within the mPFC.

FIGURE 6Astrocytic P2X7Rs regulate intracellular Ca^2+^ signals in excitatory (likely glutamatergic) and inhibitory (likely GABAergic) neurons evoked by stressful stimulation, and are differentially modulated by EA. (A and B) Whereas the excitatory (Glu) neuronal Ca^2+^ signals evoked by TST were inhibited by genetic deletion of P2X7Rs (“P2X7 KO”), the inhibitory (GABA) neuronal Ca^2+^ signals were increased by overexpression of astrocytic P2X7Rs (“GFAP over shRNA”). (C and D) Whereas the excitatory (Glu) neuronal Ca^2+^ signals evoked by foot shock were inhibited by genetic deletion of P2X7Rs (“P2X7 KO”), the inhibitory neuronal Ca^2+^ signals were increased by overexpression of astrocytic P2X7Rs (“GFAP over shRNA”). TST lasted for 6 min and foot shock lasted for 20 min (see Section 4). (E and F) The excitatory and inhibitory neuronal Ca^2+^ signals evoked by restraint stress (duration: 30 min) were modified by EA treatment. The restraint stress‐induced Ca^2+^ signaling was increased by EA in the excitatory neurons, but was decreased in the inhibitory ones. This effect was long‐lasting and persisted after ceasing EA. (G and H) All effects were abolished by pretreatment of the two neuronal cell types by “glutamate‐ or GABA‐specific,” “over shRNA.” The left panels of A–H show the changes in Δ*F*/*F*_0_ (%) of the intracellular Ca^2+^ signal in dependence of time, the middle panels show the heatmaps, and the right panels display the area under the curve (AUC; %) during a 20‐min evaluation time. The data are shown as mean ± SEM. Two‐way ANOVA was followed by Tukey's test for statistical analysis in A–H; Kruskal–Wallis ANOVA on ranks, followed by Tukey's test was used in I, and one‐way ANOVA, followed by Holm‐Sidak test was used in J. ^*^ *p* < 0.05, ^**^ *p* < 0.01 compared to the control groups; ^§§§^ *p* < 0.001, compared to foot shock in I and J. The number of individual animals is indicated in each column.

**Figure:**
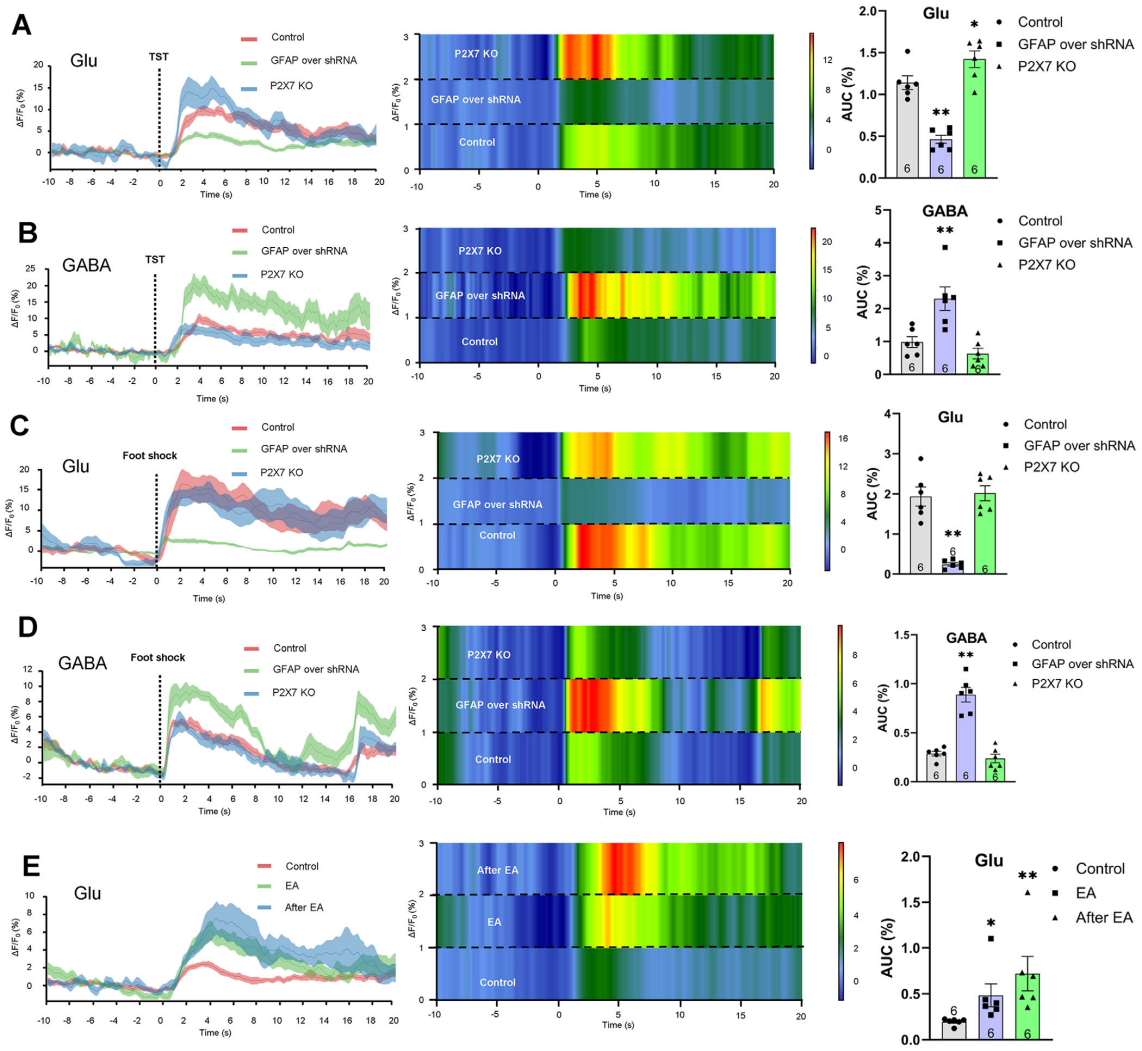

**Figure:**
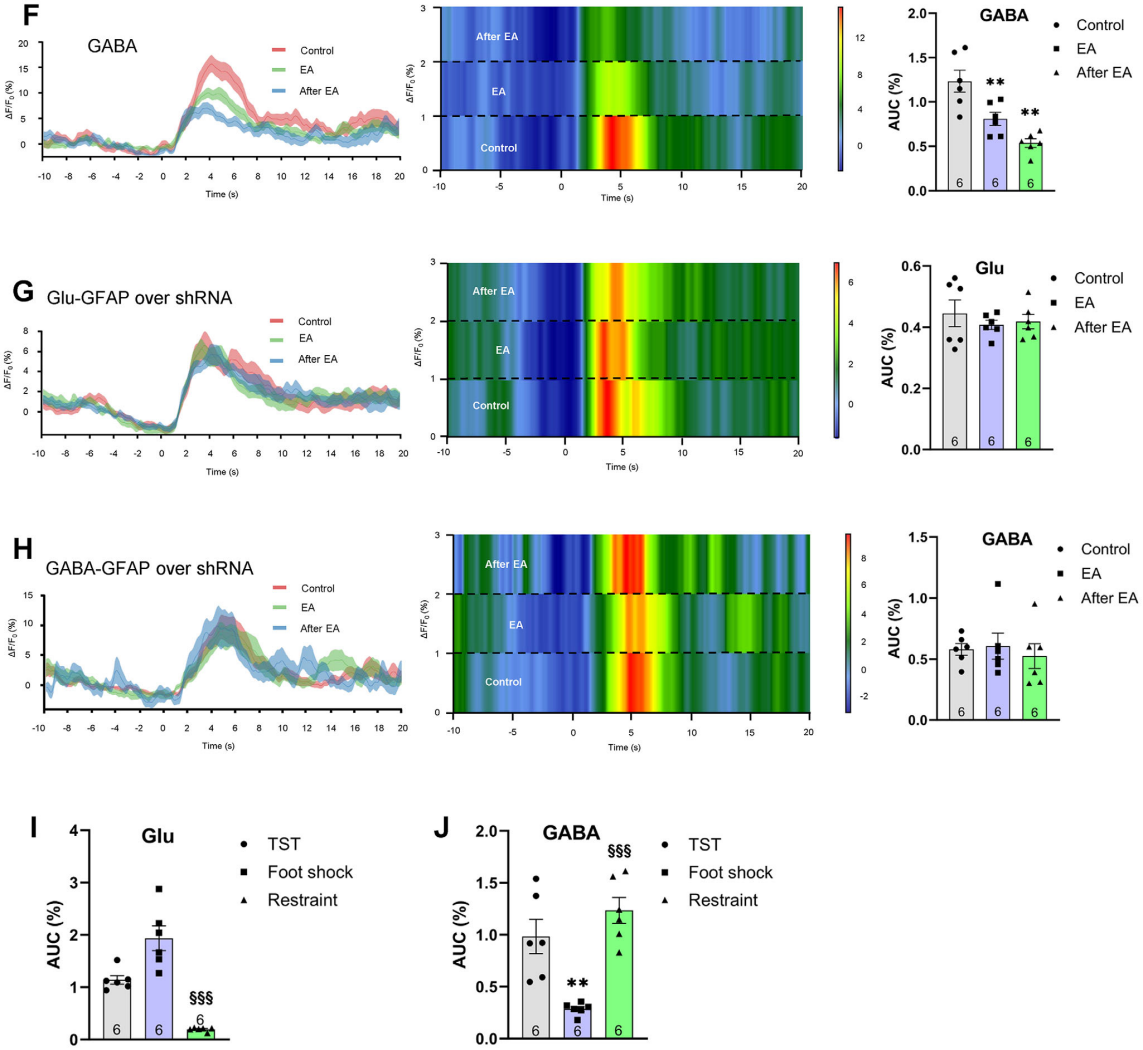

We did not confirm reduced excitatory and enhanced inhibitory neuronal activity, which reflects the altered [Ca^2+^]_i_ concentration following astrocytic P2X7R overexpression, but assumed that this might certainly be a result of alterations in the release of the gliotransmitters glutamate and GABA [39, 40]. Similarly, the pathways downstream of EA‐regulated changes in neuronal calcium signaling via astrocytic P2X7Rs remain unclear.

Next, we examined calcium signaling in excitatory and inhibitory neurons in response to restraint stress, another stress paradigm known to induce depressive‐like behaviors. In this study, we investigated the effects of EA on stress‐induced neuronal calcium activity. Restraint stress produced prolonged changes in intracellular calcium levels in both neuronal populations, extending well beyond the 20‐min analysis window routinely used in our experiments (see Section 4; Figure S4A–C). Therefore, calcium responses were analyzed both during the initial 20‐min period and during the subsequent 20‐min period following EA treatment (“after EA”). EA potentiated the effect of restraint in excitatory neurons but decreased the effect of restraint in inhibitory neurons (Figure 6E,F). Notably, these effects were abolished when the mPFC was pre‐injected with the astrocyte‐specific P2X7R overexpression construct (GFAP over shRNA) 2 weeks before calcium imaging (Figure 6G,H). These findings suggested that the modulatory effects of EA on neuronal calcium activity depend, at least in part, on astrocytic P2X7R signaling. Interestingly, the effects of EA on excitatory and inhibitory neuronal activity differed from those observed following the genetic manipulation of astrocytic P2X7Rs under other stress paradigms. In addition, astrocyte‐specific deletion of P2X7Rs affected calcium responses in excitatory neurons during the TST but not during foot shock stimulation, indicating that distinct stressors may engage different neuronal circuits.

As summarized in Figure 6I, the three stress paradigms induced distinct patterns of neuronal calcium activity. Restraint stress produced little or no increase in calcium signals in excitatory neurons but markedly increased calcium responses in inhibitory neurons. In contrast, foot shock stimulation elicited robust calcium responses in excitatory neurons while producing minimal effects in inhibitory neurons. These observations further supported the hypothesis that different stressors recruit different neuronal populations and circuit mechanisms.

### 2.6 CUMS Does Not Affect Numbers of GFAP‐ or S100β‐Immunopositive Cells in Two Subfields of Hippocampal Regions, Neither It Modifies the Amplitude of Bz‐ATP‐Induced Currents in the Hippocampal CA1 Region

In view of the marked changes described to occur after CUMS treatment in mPFC astrocytic functions, and to determine whether the number of astrocytes is decreased by CUMS, we performed this measurement in the hippocampus rather than in the mPFC itself, because our previous electrophysiological experiments indicated that acute stress by TST reduces the amplitude of Bz‐ATP currents in rat CA1 astrocytes of the hippocampus, but not in those of the mPFC [15]. The hippocampus and mPFC are both critically involved in emotional and affective functions and are considered depression‐relevant areas of the brain [41, 42]. An important afferent connection of the mPFC, the hippocampal‐prefrontal pathway (a monosynaptic unidirectional connection), is highly sensitive to stress, which is a major precipitating factor for the symptoms of depression [43].

However, CUMS did not significantly alter the number of GFAP‐ or S100β‐IR astrocytes in either the CA1 region or the stratum oriens of the mouse hippocampus (Figure S2A–D). Likewise, CUMS had no significant effect on dibenzoyl‐ATP (Bz‐ATP, 1000 µM)‐evoked currents in either neurons or astrocytes of the CA1 region (Figure S3A–C). Similarly, AMPA‐ and muscimol (100 µM each)‐induced currents were not affected by CUMS exposure. As shown in Figure S3A, neurons and astrocytes were differentiated by current injection into their respective cells. Neurons responded to depolarizing current injections above a certain threshold by firing action potentials, whereas astrocytes produced only passive depolarization. Consistent with the cellular quantification results, CUMS did not significantly alter the Bz‐ATP‐induced current responses in either CA1 neurons or astrocytes (Figure S3B,C). Similarly, AMPA‐ and muscimol‐induced currents remained unchanged following CUMS treatment in both cell types.

## 3 Discussion

CUMS is considered a reliable and effective rodent model of MDD that can induce several symptoms associated with depression, such as behavioral despair and anhedonia [44, 45]. In the present study, CUMS treatment significantly altered behavior in the TST, FST, SPT, and OFT, confirming that the model was effective under our experimental conditions.

Astrocytes play an important role in synaptic maintenance, uptake of neurotransmitters, recycling of glutamate and GABA, neuron–glia communication, and the formation of the blood‐brain barrier and perivascular space [46, 47]. Astrocyte malfunction has been consistently observed in patients with MDD, including morphometric changes and reduced cell numbers, which may lead to an imbalance in neurotransmission and aberrant synaptic connectivity [48, 49]. In contrast to those human reports, the present study found no reduction in astrocyte numbers (GFAP‐ or S100β‐immunopositive cells) in the hippocampal CA1 and stratum oriens regions of CUMS‐exposed mice. Furthermore, the amplitudes of neuronal and astrocytic currents evoked by Bz‐ATP, a prototypic agonist of P2X7Rs, did not change in the CA1 area of mice exposed to CUMS. The currents evoked by AMPA (an ionotropic glutamate receptor agonist) and muscimol (an agonist of ionotropic A‐type γ‐aminobutyric acid [GABA_A_] receptors) were not altered.

The remaining experiments focused on the mPFC, a brain region considered a critical depression‐related area [50, 51]. The number of astrocytes has been reported to decrease in the mPFC of patients with depression, and the selective elimination of astrocytes in rodents is sufficient to induce depressive‐like behavior [52]. Previous studies have largely focused on microglial P2X7Rs as mediators of neuroinflammation [53, 54]. However, whether P2X7Rs in astrocytes are directly involved in depression, in contrast to indirectly involved neurons, microglia were not at the forefront of the present investigations, especially because the decisive role of microglial P2X7Rs in acute, but not chronic, models of stress‐induced behavioral reactions was excluded in a previous study [38]. Consistent with this notion, astrocyte‐specific manipulation of P2X7Rs in the present study produced marked behavioral effects, indicating that astrocytic P2X7Rs represent a previously underappreciated contributor to chronic stress‐induced depression.

Previous studies have demonstrated that astrocytic P2X7Rs are involved in depressive‐like behaviors induced by acute stress [14, 15]. To investigate whether astrocytic P2X7Rs in the mPFC were altered in the chronic stress model, an astrocyte‐specific P2X7R shRNA was generated. The observed upregulation of astrocytic P2X7Rs in the mPFC following CUMS exposure suggests that purinergic signaling contributes to chronic stress‐induced neuropathology. Reversal of these changes by astrocyte‐specific knockdown supports the causal role of astrocytic P2X7Rs in the development of depression‐like phenotypes. These data indicate that astrocytic P2X7Rs are involved in the chronic CUMS model of MDD.

In addition, exposure to unavoidable stress has been reported to reduce excitatory inputs to the prefrontal cortex, indicating a decline in functional plasticity of glutamatergic synapses [55, 56]. Dynamic two‐way communication between astrocytes and neurons at the synapses produces excitatory or inhibitory responses [47]. Previous studies have indicated that P2X7Rs are present in nearby astrocytes and indirectly regulate neuronal activity [57]. In the rat hippocampal CA1 region, astrocytic P2X7Rs release glutamate and GABA from astrocytes to stimulate neighboring neurons, where functional P2X7Rs are absent [58]. The present study examined whether astrocytic P2X7Rs regulated the activity of mPFC neurons during CUMS.

Ca^2+^ signaling is a key component of the mechanisms regulating neuronal excitability and synaptic transmission efficiency [59, 60]. Fiber photometry recordings of [Ca^2+^]_i_ changes in mPFC neurons showed that genetic deletion of astrocytic P2X7Rs or their conditional overexpression each altered [Ca^2+^]_i_ levels in both excitatory and inhibitory neurons under CUMS. These findings indicate that astrocytic P2X7Rs modulate the balance between excitation and inhibition of neuronal circuits during depression‐like states.

Long‐term therapeutic experience, especially in Asia, has demonstrated that acupuncture is an effective, safe, and well‐tolerated treatment for depression [61]. Accumulating evidence has shown that acupuncture can relieve symptoms and improve the quality of life of patients with depression or alleviate depression‐like symptoms in animal models of depression [62, 63]. EA is used for the treatment of depression by acting through multiple previously defined mechanisms, such as regulating neurotransmitter balance [64], synaptic remodeling [65, 66], inhibiting neuroinflammation [67, 68], and affecting the neuroendocrine and metabolic systems [69].

In the present study, behavioral assessments using the FST, TST, SPT, and OFT were performed after EA treatment of mice exposed to CUMS. The FST and TST are common tests of behavioral despair in rodents that are widely used to evaluate antidepressive effects [70, 71, 72, 73], whereas the SPT is used to assess depression‐induced anhedonia. The OFT is commonly designed to measure anxiety and depression as well as behaviors such as general locomotor activity and willingness to explore a novel environment. CUMS treatment induced a depressive‐like phenotype, as evidenced by prolonged immobility times in both the FST and TST, decreased sucrose consumption in the SPT, significantly reduced total distance traveled in the OFT, and impaired rearing frequency. EA was applied to the ST36 acupoint, which has been reported to beneficially influence depression‐induced astrocytic atrophy in the mPFC [18], modulate excitation‐inhibition balance in the cortex [74], and trigger functional connectivity in several brain areas, including the amygdala, caudate putamen, thalamus, and cerebral cortex in depressive‐like states [75].

Acupuncture leads to the release of large amounts of ATP into the skin, which then occupies specific receptor subtypes expressed on sensory nerve terminals to send afferent impulses and connect with interneurons, likely in the midbrain/medulla oblongata, to modulate neuronal activity in the brain [76]. Purinergic signaling has been identified as a key pathway in the mechanism of acupuncture, particularly in the context of analgesia [77, 78, 79].

EA treatment significantly alleviated CUMS‐induced hippocampal neuroinflammation, as reflected by increased expression of P2X7R, IL‐18, TNFα, and IL‐6 [80]. In the present study, exposure of mice to the CUMS regimen led to a significant increase in P2X7R expression in the mPFC, which was completely prevented by EA delivered to the ST36 acupoint. Sham acupuncture was ineffective, indicating a selective effect of EA. In addition, overexpression of P2X7Rs in mPFC astrocytes ameliorated the effects of EA. These findings are consistent with previous reports that acupuncture improves CUMS‐induced depressive‐like behaviors, promotes the recovery of neural plasticity in the prefrontal cortex [81], and restores CA1 neuronal excitability after chronic social defeat stress (CSDS) [82].

Similarly, [Ca^2+^]_i_ measurements in the mPFC showed that P2X7R stimulation by TST and foot shock enhanced excitatory neuronal activity and decreased inhibitory neuronal activity in the mouse mPFC. Both effects were abolished by overexpression of astrocytic P2X7Rs in excitatory and inhibitory neurons, and this manipulation blocked the effect of EA on these neuronal types.

Post hoc power analyses indicated adequate statistical power for the majority of the outcome measures (Figure S4). For several endpoints that exhibited small effect sizes and substantial biological variability, the sample sizes required to achieve 95% power exceeded the commonly accepted standards for animal studies. Therefore, the nonsignificant findings in these experiments are more likely to reflect limited biological effects rather than insufficient statistical power.

In conclusion, the present study provides experimental evidence that astrocytic P2X7Rs play a pivotal role in the development of chronic stress‐induced depressive‐like behavior. These data further indicate that EA modulates the balance between excitatory and inhibitory neuronal activity via a permissive role on astrocytic P2X7Rs in the mPFC, thereby exerting an antidepressant effect.

## 4 Materials and Methods

### 4.1 Animals

C57BL/6J male mice (from Chengdu Dossy Experimental Animal Co., Chengdu, China), 7 weeks old, were used for all experiments reported in this study. In some experiments, P2X7R KO mice (*P2rX7tm1.2Jde*; MGI:6203042) and their wild‐type (WT) controls were used (generated by Jan M. Deussing, Max Planck Institute of Psychiatry, Munich, Germany).

### 4.2 Chronic Unpredictable Mild Stress

CUMS and all other experimental procedures were performed in accordance with the time course shown in Figure S5. Mice were exposed to 24‐h random sequences of stressors for 4 weeks, as previously described [14]. These stressors included water and food deprivation (12 h), cage tilt (12 h), group housing (12 h), swimming in 4°C water (5 min), foot shock (1 mA current strength, 1 s duration for 5 min), noise (120 dB for 3 h), tail suspension (5 min), damp bedding (12 h), cage shaking (40/min for 5 min), and restraint (1 h) (Table S1). To ensure the unpredictability of the stimulation, these stressors were changed randomly once daily.

### 4.3 Experimental Groups and Treatments

Eighteen experimental animal groups were established. For clarity, the groups were categorized according to the type of intervention as follows: (1) Control and CUMS groups. In the control group, mice were housed under standard conditions and received no treatment. In the CUMS group, the mice were exposed to CUMS alone. (2) Pharmacological intervention groups. CUMS+fluoxetine: The mice were subjected to CUMS and received fluoxetine (10 mg/kg, intraperitoneally (ip); Sigma‐Aldrich, St. Louis, MO, USA; dissolved in phosphate buffered saline (PBS)) once weekly. CUMS+PBS: The CUMS‐exposed mice were injected with PBS (0.2 mL [ip]) once weekly (vehicle control). (3) EA intervention groups. EA: The mice were subjected to CUMS and received daily EA treatment (parameters described below). Sham EA1: Mice were subjected to CUMS and underwent needle placement at a non‐acupoint on the tail without electrical stimulation. Sham EA2: Mice were subjected to CUMS, and a wooden toothpick was placed onto the ST36 acupoint without skin penetration. (4) Genetic manipulation groups. CUMS+P2X7 KO: CUMS‐exposed mice with a global P2X7R knockout. CUMS+GFAP KD shRNA: CUMS‐exposed mice injected with an astrocyte‐specific conditional P2X7R KD virus. Control+GFAP CshRNA: Non‐stressed mice injected with the control virus for astrocyte‐specific knockdown. CUMS+GFAP CshRNA: CUMS‐exposed mice injected with the control virus. CUMS+GFAP over shRNA+EA: CUMS‐exposed mice receiving daily EA and injected with astrocyte‐specific conditional P2X7R overexpression virus. Control+GFAP over CshRNA: Non‐stressed mice injected with the control virus for astrocyte‐specific overexpression. CUMS+GFAP over CshRNA: CUMS‐exposed mice were injected with control virus for astrocyte‐specific overexpression. Control+Syn CshRNA: Non‐stressed mice injected with the control virus for neuron‐specific P2X7R KD. CUMS+Syn CshRNA: CUMS‐exposed mice injected with the same control virus. Control+Syn KD shRNA: Non‐stressed mice injected with neuron‐specific conditional P2X7R KD virus. CUMS+Syn KD shRNA: CUMS‐exposed mice injected with the same knockdown virus.

#### 4.3.1 EA Procedure

EA was administered at approximately the same time of day (10:00 a.m. to 11:00 a.m.) to awake mice. Mice were immobilized with two Velcro hooks and loop fasteners, as well as additional tape fixed to a wooden block for the duration of EA. EA was delivered to the therapeutically relevant ST36 acupoint (located on the left knee, approximately 2 mm below the fibular head). Stainless steel needles (2.5 cm long, 0.25 mm diameter; Hwato‐Med. Co., Jiangsu, China) were inserted 2–3 mm deep unilaterally at ST36. An electrical current of 0.5 mA and 2 Hz frequency was applied for 30 min using an acupoint nerve electrical stimulator (HANS‐200, Nanjing Jisheng Medical Technology Co., Jiangsu, China). For sham EA, two procedures were used: (1) needle insertion at a non‐acupoint (tail) without electrical stimulation or (2) a wooden toothpick placed onto ST36 without insertion. Both sham treatments were administered to separate groups of CUMS‐exposed mice.

### 4.4 Behavioral Tests and Stressful Stimuli

#### 4.4.1 Tail Suspension Test

The TST was performed as previously described [14]. Animals were suspended on a 55 cm high laboratory rack using adhesive tape positioned about 1 cm from the tail tip. The approximate distance between the animal's nose and the operating floor was 20–25 cm. The animals were separated from each other using baffles to prevent mutual interference. The mice were suspended for 6 min. The cumulative immobility time was recorded by an observer blinded to the treatment groups. Mice were considered immobile only when they remained completely motionless; mice that climbed along their tails were not included in the evaluation. The data were video‐recorded and analyzed later.

#### 4.4.2 Forced Swim Test

The FST was performed as previously described [14]. This test was made in a clear glass cylinder filled with water (temperature 23–25°C, height 30 cm, diameter 20 cm) and the water level was 15 cm from the bottom. The duration of immobility within the 6‐min test was recorded. Following the swimming session, the mice were removed from the water using their tails, gently dried with towels, and kept warm under a lamp in their home cages. A mouse was considered immobile whenever it stopped swimming and remained passively floating, keeping its head above the water surface.

#### 4.4.3 Open Field Test

The OFT was performed as previously described [14]. The apparatus consisted of a rectangular chamber (50 × 50 × 50 cm) made of white, high‐density, non‐porous plastic. Mice were gently placed in the center of an open‐field chamber. The camera recorded the mice's routes as they moved for 10 min. The total distance, rearing frequency, and time spent in the center versus the periphery of the chamber were recorded using a camera interfaced with a computer running an automated video‐tracking system (EthoVision XT 9.0, Noldus, Netherlands). The chamber was thoroughly cleaned with 95% ethanol and dried prior to use and before subsequent tests to remove any scent clues left by the previous mouse.

#### 4.4.4 Sucrose Preference Test

The SPT is a reward‐based test performed as previously described [15]. The mice were singly caged for 3 days and given two 50 mL bottles containing water or water‐based 1% sucrose solution (wt/vol). Bottle positions were switched daily to avoid side biases. Following a 24‐h period of water and food deprivation, the preference for sucrose or water was determined overnight. The sucrose preference (%) was quantified as (vol sucrose/(vol sucrose+vol water)) × 100.

#### 4.4.5 Inescapable Foot Shock

An inescapable foot shock experiment was performed using a Learned Helplessness Test System (Shanghai Xin‐Ruan Information Technology Co., Shanghai, China). Mice were placed in the apparatus and received electric foot shocks at a current intensity of 1 mA for 20 min; each stimulus lasted for 5–15 s with random intervals between them.

#### 4.4.6 Restraint Stress

Mice were immobilized for 30 min using the procedure described above for EA treatment but without EA.

### 4.5 Viruses

To knockdown murine P2X7R (*mp2rx7*) expression in specific cell types, different adeno‐associated viruses (AAVs) were applied expressing shRNA directed against *mp2rx7* (5‐GCG GAA AGA GCC TGT TAT CAG‐3), or control, inefficient shRNA, both driven by cell type‐specific promoters. The following viruses were purchased from Guangzhou PackGene Biotechnology Co., Ltd. (Guangzhou, China): (1) General P2X7R KD shRNA and its control: AAV9[scAAV.U6.mP2X7 shRNA.CAG.EGFP.WPRE.SV40pA] and AAV9[scAAV.U6. N‐control shRNA.CAG.EGFP.WPRE.SV40pA]; (2) neuron‐specific P2X7R KD shRNA and its control: AAV9_hSyn‐EGFP‐miR30 shRNA(mP2X7)‐WPRESV40pA and AAV9_hSyn‐EGFP‐miR30shRNA(NC)‐WPRE‐SV40pA; (3) astrocyte‐specific P2X7R KD shRNA and its control: AAV8_GFAP(681 bp)‐EGFPmiR30shRNA(mP2X7)‐WPRE‐SV40pA and AAV8_GFAP(681 bp)‐EGFP‐miR30shRNA(NC)‐WPRE‐SV40pA; (4) general P2X7R overexpression shRNA and its control: AAV2/9‐CMV_bGI‐mCherry‐P2A‐mP2RX7‐3xFlag‐WPRE‐pA and AAV2/9‐CMV_bGI‐mCherry‐WPRE‐pA; and (5) astrocyte‐specific P2X7R overexpression shRNA and its control: AAV2/5‐gfaABC1D‐mCherry‐P2A‐mP2X7‐3xFlag‐WPRE‐pA and AAV2/5‐gfaABC1D‐mCherry‐WPRE‐pA. Two weeks after the virus injection, the experiments were started.

### 4.6 Stereotaxic Surgery

Mice were anesthetized with isoflurane (5% induction; 2% maintenance; RWD Life Science, San Diego, CA, USA) and fixed on a stereotactic platform (RWD Life Science). They were placed on heating pads (37°C) during the surgery to maintain a stable body temperature. A 1‐µL Hamilton syringe fixed with beveled glass pipettes was inserted into the bilateral mPFC (+1.5 mm anteroposterior [AP], ±0.5 mm mediolateral [ML] from the bregma, −2.5 mm dorsoventral [DV] from the skull [83]). Cell‐specific shRNAs were injected at a volume of 0.5 µL. Glass pipettes were left in place for at least 5 min. At the end of surgery, 5 mg/kg enrofloxacin (Bayer, Leverkusen, Germany) was administered subcutaneously (sc) to the animals to prevent postoperative infection. After injection, the animals were allowed to completely recover under a warming blanket and returned to their home cages. An interval of 2 weeks was maintained between shRNA application and the start of behavioral or immunohistochemical experiments.

### 4.7 In Vivo Fiber Photometry Recording

Fiber photometry recording was performed as previously described [84]. Fourteen days before recording the calcium signals, the AAVs rAAV‐CaMKIIa‐GCaMP6s‐WPRE‐pA (generates in excitatory neurons the genetic Ca^2+^ indicator) and rAAV‐VGAT1‐GCaMp6m‐WPRE‐hGH (generates in inhibitory neurons the genetic Ca^2+^indicator) were injected into the mPFC in a volume of 0.2 µL (both from Brain Case, Wuhan, China). For the viral injection, a glass pipette was mounted on a Nanoject 3 infusion system (Drummond Scientific, Broomall, PA, USA). Injection was into the left mPFC with the stereotaxic coordinates AP = 1.78 mm, ML = −0.35 mm, DV = −2.25 mm. Glass pipettes were left in place for at least 5 min.

Population calcium signals were collected using a commercial fiber photometry system (Thinker Tech Inc., Singapore). Fourteen days after injection of the viruses to induce the genetic Ca^2+^ indicators in excitatory and inhibitory neurons, an optic fiber coated with ceramic ferrule (diameter: 1.25 mm, Tinker Tech Inc.) was implanted into the prelimbic area of the mPFC at the stereotactic coordinates AP = 1.94 mm, ML = ±0.45 mm, DV = −2.4 mm [85, 86]. The base of the optical fiber was fixed to the skull with a screw. The correctness of the implantation site was controlled histologically after some experiments, and the intended location of the optic fiber was confirmed.

Before recording, the mice were habituated to the fiber‐optic cord and empty cage for 10 min in a home cage with normal bedding. During recording, the voltage signal data for 410 nm (as movement artifacts) and 465 nm (for the GCaMP signal) were exported. The fluorescence signals were filtered (35 Hz cutoff) and normalized to calculate the fluorescent change (Δ*F*/*F*_0_), where F_0_ was the baseline fluorescent level. The intensity of the fluorescence signal was calculated between 0 and 20 s, and expressed as the area under the curve (AUC) using MATLAB 2017B program (MathWorks, Natick, MA, USA).

[Ca^2+^]_i_ measurements were made before (−10 min) and during (6 min TST; 20 min foot shock; 30 min restraint, with or without EA) for 30 min. The “after EA” value was determined over a 30‐min period, after stopping EA.

### 4.8 Immunofluorescence Staining

Immunofluorescence staining was performed as previously described [38, 84] with minor modifications. Mice were perfused through the ascending aorta with cold paraformaldehyde (PFA, 4% w/v in PBS) after an ip injection of 1% sodium pentobarbital (0.4 mL). Brains were removed and post‐fixed overnight in 4% PFA. Then, they were dehydrated with a gradient (20%–30%) sucrose in PBS at 4°C. Coronal 40‐µm‐thick sections were prepared and incubated in a cryostat (Leica CM1860; Leica Biosystem, Muttenz, Switzerland) at −80°C until use. Sections were then incubated in a blocking solution containing 4% bovine serum albumin (Sigma‐Aldrich) and 0.5% Triton X‐100 (Solarbio, Beijing, China) for 2 h at room temperature. Subsequently, the sections were incubated with the primary antibody (mouse anti‐GFAP, 1:500; Cell Signaling Technology, Boston, MA, USA; mouse anti‐S100β, 1:200; Abcam, Cambridge, MA, USA; mouse anti‐NeuN 1:1000; Abcam, Cambridge, MA, USA; rabbit anti‐Iba1, 1:200; Abcam, Cambridge, MA, USA) overnight at 4°C. After washing in PBS three times, the slices were incubated for 2 h in a saturation solution containing the relevant secondary antibodies (goat anti‐mouse AF594 and goat anti‐rabbit AF594, both 1:200; Abcam, Cambridge, MA, USA). After washing three times in PBS and labeling the cell nuclei with DAPI (1:10,000; Abmole Bioscience, TX, USA), the coverslips were mounted on slides using an anti‐fade solution (Solarbio, Beijing, China). Images were acquired with a confocal laser scanning microscope (Olympus IXplore Spin SR; Olympus, Tokyo, Japan).

Each mouse delivered approximately to 4–6 slices, and for each slice, 3–6 images were captured to ensure that the complete CA1 or stratum oriens regions were covered. These images were then combined into a single full image (Figure S2). Random regions of interest (ROIs) of 300 × 300 µm or 100 × 100 µm were selected for GFAP or S100β counting, as demonstrated in Figure S1.

The colocalization of S100β‐, Iba1‐, and NeuN‐IR with EGFP‐IR was quantified using the Cell Counter plugin in ImageJ (NIH, Bethesda, MD, USA). Quantification of each cell type was performed as the ratio of double‐positive cells to the EGFP‐positive population.

### 4.9 Western Blotting

Western blotting was performed as described previously. The mPFC was dissected and lysed for total protein extraction in Lysis Buffer (Thermo Fisher Scientific, Massachusetts, USA) together with a protease inhibitor, EDTA (Roche, Almere, The Netherlands). Protein concentrations were assessed using the BCA Protein Assay Kit (Thermo Fisher Scientific, Waltham, MA, USA), and the samples were boiled in sample buffer. 50 µg of protein was applied to an SDS‐polyacrylamide gel and subsequently transferred to PVDF membranes. Membranes were blocked with 5% skimmed milk in Tris‐buffered saline with Tween 20 for 1 h at room temperature and subsequently incubated overnight at 4°C with the following primary antibodies: P2X7R (1:1000; Abcam, Cambridge, UK), CD39 (1:1000; Abcam), CD73 (1:1000; Proteintech, Rosemont, USA), SNAP23 (1:1000; Proteintech), SNAP25 (1:1000; Abcam), CALHM2 (1:400; Proteintech), and GAPDH (1:1000; Proteintech). After incubation with a horseradish peroxidase‐conjugated anti‐mouse antibody (1:5,000; Proteintech) at room temperature for 1 h, the immune complex was detected using ECL Prime Western blotting detection reagent (KeyGENBioTECH, Nanjing, China). The ChemiDocXRS + imaging system with Image Lab Software (BIO‐RAD, Hercules, CA, USA) was used for development. The intensities of the bands on the membranes were analyzed using ImageJ software. The original, uncropped Western blot images with unadjusted contrast are shown in Figure S6.

### 4.10 Quantitative Real‐Time RT‐PCR (qPCR)

The mPFC region from the brain was collected and stored in liquid nitrogen. Total RNA was extracted from the mPFC using an RNeasy Mini Kit (Qiagen, Venlo, The Netherlands), and a Nanodrop (Thermo Fisher Scientific, Waltham, MA, USA) was used to evaluate the concentration and quality (260/280 absorbance ratio) of the total RNA. For each reaction, 1 µg total RNA was added to a final volume of 20 µL of mixed reagent for reverse transcrition into cDNA using the QuantiNova Reverse Transcription Kit (Qiagen). The mRNA expression levels in the mPFC were estimated using qPCR with specific primers. The reactions were performed with QuantiNova SYBR Green PCR Kit (Qiagen) in QuantStudio 6 Flex Real‐Time PCR System (Thermo Fischer Scientific) under the following reaction conditions: 95°C for 10 min, followed by 40 cycles of 95°C for 15 s and 60°C for 15 s, 95°C for 15 s, 60°C for 15 s, 95°C for 20 min, and 95°C for 15 s. The relative expression levels of mRNAs were calculated by the 2‐ΔΔCt method. β‐actin was used as a reference gene. The forward and reverse primer sequences were the following, respectively: β‐actin, GGCTGTATTCCCCTCCATCG and CCAGTTGGTAACAATGCCATGT; P2X7, TGTCCCTATCTCCAGACTCAC and ATTTCCACACTGGCACCAACTCGG; SNAP23, CACTATGCTGGATGAGCAAGG and CAACACTTGTTGAGTTCTGTTAAAGTC; SNAP25, CCTTCTGCTGTGCTCTCCTC and CAATGGGGGTGACTGACTCT; CD39, CACCAAGGTTCAGCAGATCCGC and GTTCATCAATGGGCGACCGG; CD73, CACCAAGGTTCAGCAGATCCGC and GTTCATCAATGGGCGACCGG; Cahlm2, CCTGACCAAGTGCCTCAAACA and GGTCCTCGTTGGTACGGTACTG.

### 4.11 Patch‐Clamp Recording

C57BL mice, 7 weeks old, were utilized for this experiment. After 4 weeks of exposure to CUMS, the mice were euthanized with an ip injection of 1% sodium pentobarbital (0.4 mL; Sigma‐Aldrich, St. Louis, MO, USA). Hippocampal slices were prepared and patch‐clamp procedures were performed as described previously [15]. After decapitation, the brain was placed in ice‐cold, oxygenated (95% O_2_ + 5% CO_2_) artificial cerebrospinal fluid (aCSF) with the following composition (mM): NaCl 126, KCl 2.5, CaCl_2_ 2.4, MgCl_2_ 1.3, NaH_2_PO_4_ 1.2, NaHCO_3_ 25, and glucose 11. Hippocampal slices were cut at a thickness of 200 µm by using a vibratome (VT1200S; Leica Biosystem, Muttenz, Switzerland). Whole‐cell current and voltage‐clamp recordings were performed using a patch‐clamp amplifier (MultiClamp 700 B; Molecular Devices, San Jose, CA, USA). Patch pipettes were filled with an intracellular solution with the following composition (in mM): K‐gluconic acid 140, NaCl 10, MgCl_2_ 1, HEPES 10, EGTA 11, Mg‐ATP 1.5, Li‐GTP 0.3, and pH 7.3, adjusted with KOH. In the voltage‐clamp recording mode of the amplifier, the holding potential of astrocytes was set to −80 mV, and that of neurons to −70 mV.

Astrocytes were discriminated from neurons by visual observation and by their failure to fire action potentials in response to suprathreshold depolarizing current injection. AMPA (100 µM), Bz‐ATP (1000 µM), and muscimol (100 µM), all from Sigma‐Aldrich, were applied locally to astrocytes and neurons by means of a computer‐controlled solenoid valve‐driven pressurized superfusion system (VC^3^8; ALA Scientific Instruments, Farmingdale, NY, USA). The drug application tip of the pressurized superfusion system touched the surface of the brain slice and was placed 100–150 µm afar from the patched cell. Agonists were applied for 10 s every 3 min in a low X^2+^ aCSF solution (MgCl_2_ was omitted from the medium and the CaCl_2_ concentration was decreased to 0.5 mM). When the same concentration of an agonist was applied twice, the mean current response was calculated for statistical evaluation.

### 4.12 Data Analysis

Statistical analyses were performed using GraphPad Prism 8.0. All data were expressed as means ± SEM of *n* observations, where *n* signifies, in the behavioral and Ca^2+^ imaging experiments, the number of mice, or, in the residual experiments, the number of cells taken from at least three mice. The distribution of the data was checked using the Shapiro–Wilk test. Two datasets were compared using the parametric Student's *t*‐test or non‐parametric Mann–Whitney rank sum test, as appropriate. Data from more than two groups and one variable were tested for significance using one‐way ANOVA test followed by the Holm–Sidak test. Multiple comparisons between data and one variable were tested in case of non‐normal distribution using Kruskal–Wallis ANOVA on ranks, followed by Tukey's test. Data from more than two groups and two variables were tested using a two‐way ANOVA followed by Tukey's test. A probability level of 0.05 or less was considered to be statistically significant.

## Author Contributions

W.‐J.R., P.I., and Y.T. wrote the original version of the manuscript. M.T.K., X.L., and X.‐Y.R. contributed to specific sections and revised the manuscript. P.R., H.‐Y.Y., and A.V. also revised the manuscript. All authors have read and approved the final version of the manuscript.

## Funding

We are grateful for the financial support from the National Science Foundation of China (NSFC82505421), NSFC‐RSF (82261138557), the Sino‐German Center (GZ919, M‐0679), and the Sichuan Science and Technology Program (2025YFHZ0121, 2026NSFSC1857).

## Ethics Statement

The animal study was reviewed and approved by the Institutional Review Board of Chengdu University of Traditional Chinese Medicine, Chengdu, China (protocol code AF2475; April 1, 2020).

## Conflicts of Interest

Author Peter Illes and Alexei Verkhratsky are the Editorial board member of MedComm. They were not involved in the journal's review of or decisions related to this manuscript. The other authors declared no conflict of interest.

## Supporting information




**Supporting Fie**: mco271042‐sup‐0001‐SuppMat.pdf.

## Data Availability

Data supporting the findings of this study are available from the corresponding authors upon reasonable request.
