# Supplementary material for: Both Genetic Manipulation and Acupuncture Alleviate Depressive‐Like Behavior Mediated by Astrocytic P2X7 Receptors
Source: MedComm (2020). 2026 Oct 4;7(10):e71042. doi: 10.1002/mco2.71042 (PMC13635788; doi:10.1002/mco2.71042)
Supplement: Supplementary file 1 [file MCO2-7-e71042-s001.pdf]

# Both genetic manipulation and acupuncture alleviate depressive-like behavior mediated by astrocytic P2X7 receptors

Running title: Astrocytic P2X7 receptors and chronic stress

Wen-Jing Ren[1], Muhammad Tahir Khan[1], Xing-Ying Ren[1], Xuan Li[1], Patrizia Rubini[1,2], Hai-Yan Yin[2], Alexei Verkhratsky[1,3], Peter Illes[1,4]*, Yong Tang[1]*

[1]International Research Centre on Purinergic Signaling/School of Health and Rehabilitation, Chengdu University of Traditional Chinese Medicine, Chengdu, China

[2]School of Acupuncture and Tuina, Chengdu University of Traditional Chinese Medicine, Chengdu, China

[3]Faculty of Biology, Medicine and Health, The University of Manchester, Manchester, UK

[4]Rudolf Boehm Institute for Pharmacology and Toxicology, University of Leipzig, Germany

*These authors contributed equally to the paper

Send correspondence to: Yong Tang (tangyong@cdutcm.edu.cn)
Peter Illes (peter.illes@medizin.uni-leipzig.de)

Suppl. Figure 1. The efficiency of conditional shRNAs was confirmed by immunofluorescence, qPCR and western-blotting. (A) The upmost panel shows that the EGFP fluorescence is expressed in the bilateral mPFC after viral injection of the P2X7R-selective shRNA. The co-localization of astrocytic shRNA with S100β-labelled astrocytes (upper row of left panels), Iba1-labelled microglia (middle row of left panels), and NeuN-labelled neurons (lower row of left panels) was investigated by immunofluorescence and was presented at two different magnifications each (see different length of scale bars). Cell nuclei were labeled with DAPI (blue). Quantitative evaluation of the ratio of astrocytes, microglia and neurons co-stained with EGFP as a percentage of the total EGFP staining (right panel). Kruskal-Wallis ANOVA on ranks followed by the Tukey's test. The data are presented as mean±SEM. ***$p < 0.001$ compared to the 'EGFP-S100β group'. (B-E) Expression of the p2x7r-mRNA by the ΔΔCt method with respect to β-actin-RNA after interference with general P2X7R knock down shRNA (B), astrocytic P2X7R knock down shRNA (C), general P2X7R over expression shRNA (D) or astrocytic P2X7R over expression shRNA (E). One-way ANOVA was used in (A). The unpaired t-test was used for evaluation in the case of normal distribution of data (B, C). The Mann-Whitney test was used for evaluation in the case of non-normal distribution of data (D, E). All data are presented as mean ± SEM. *$p < 0.05$, **$p < 0.01$ compared to the respective control groups. (F-I) Representative immunoblots and quantification of P2X7R-protein after interference with general P2X7R knock down shRNA (F), astrocytic P2X7R knock down shRNA (G), general P2X7R over expression shRNA (H) or astrocytic P2X7R over expression shRNA (I). The unpaired t-test was used for evaluation in the case of normal distribution of data (F-I). All data are presented as mean ± SEM. *$p < 0.05$, **$p < 0.01$, compared to the respective controls. The number of individual animals is indicated in each column.

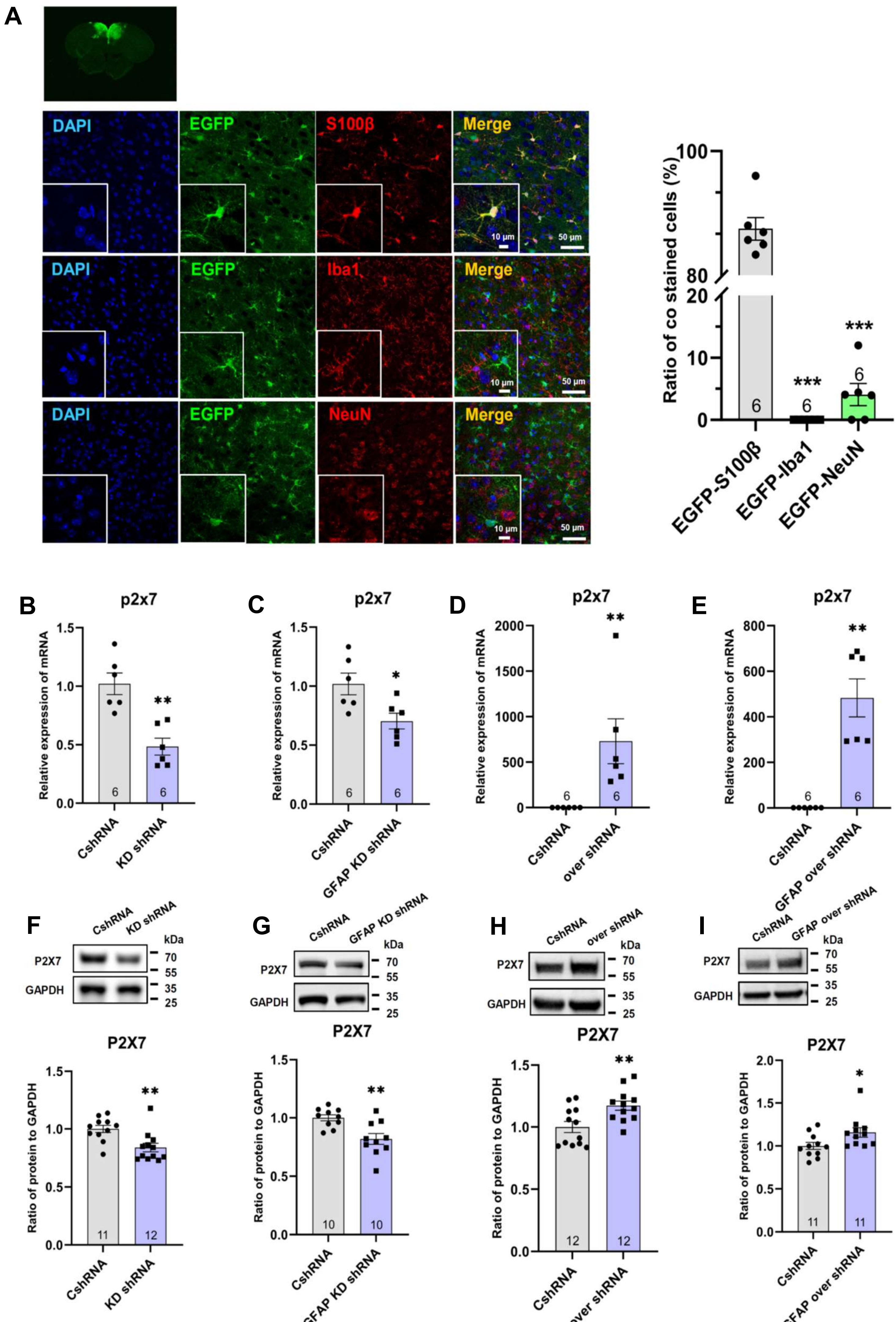

A
DAPI
EGFP
S100β
Merge
Iba1
NeuN
10 μm
50 μm
Ratio of co stained cells (%)
EGFP-S100β
EGFP-Iba1
EGFP-NeuN
B
C
D
E
p2x7
Relative expression of mRNA
CshRNA
KD shRNA
GFAP KD shRNA
over shRNA
GFAP over shRNA
F
G
H
I
P2X7
GAPDH
kDa
P2X7
Ratio of protein to GAPDH

Suppl. Figure 2. The number of astrocytes in the mouse hippocampal CA1 and stratum oriens regions did not change after CUMS-treatment of mice. Localization of the astrocytic marker glial fibrillary acidic protein (GFAP), on the CA1 (A) or stratum oriens (B) regions, by immunofluorescence staining, after exposure of the mice to CUMS. Localization of the astrocytic marker S100β on CA1 (C) or stratum oriens (D) regions, by immunofluorescence staining after exposing the mice to CUMS. The boxed areas in the left two panels of A-D signify stitch images, which are stitched together into a single full image, quantified in the right graph. All data are presented as mean ± SEM. The data for individual animals are indicated in each column also giving information on their number, which was 6 in each case. The Student's *t*-test was used for statistical analysis. There were not statistical differences in the number of GFAP- or S100β-immunopositive neurons in the CA1 or stratum oriens regions of the hippocampus. The number of individual animals is indicated in each column.

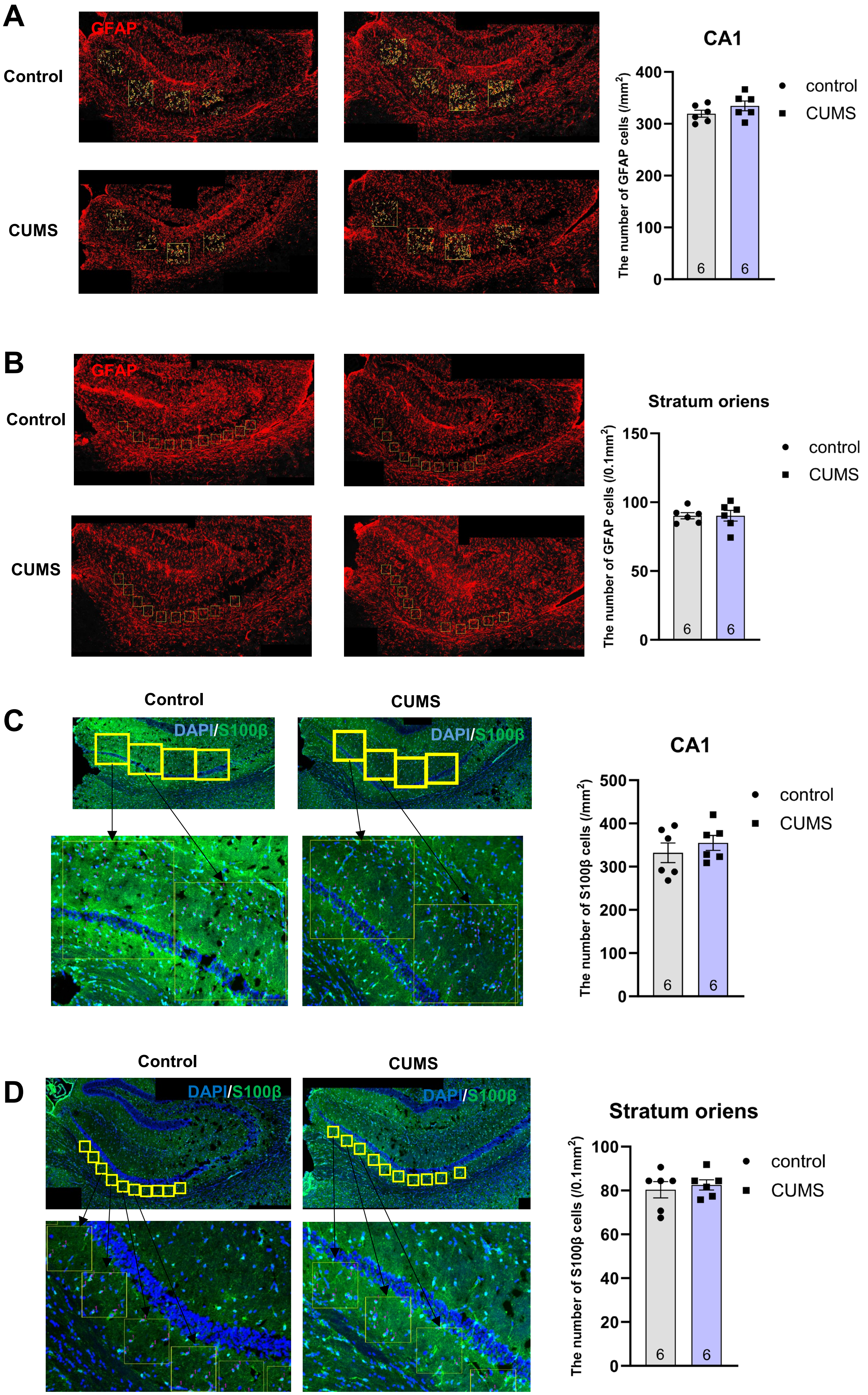

A
Control
CUMS
GFAP
CA1
The number of GFAP cells (/mm^2)
400
300
200
100
0
6
6
control
CUMS
B
Control
CUMS
GFAP
Stratum oriens
The number of GFAP cells (/0.1mm^2)
150
100
50
0
6
6
control
CUMS
C
Control
CUMS
DAPI/S100β
DAPI/S100β
CA1
The number of S100β cells (/mm^2)
500
400
300
200
100
0
6
6
control
CUMS
D
Control
CUMS
DAPI/S100β
DAPI/S100β
Stratum oriens
The number of S100β cells (/0.1mm^2)
100
80
60
40
20
0
6
6
control
CUMS

Suppl. Figure 3. Bz-ATP-induced current responses in the hippocampal CA1 neurons and astrocytes of mice exposed to CUMS. (A) Neurons (left panel) and astrocytes (right panel) were differentiated by the firing of a series of action potentials in the former, and only passive responses in the latter after depolarizing current injection above a certain threshold. (B) Current amplitudes in neurons or astrocytes caused by AMPA (100 µM), Bz-ATP (1000 µM) and muscimol (100 µM) local superfusion in hippocampal slice preparations taken from untreated or CUMS-treated mice. (C) Mean ± SEM of the indicated number of experiments on neurons (left panel) and astrocytes (right panel). One-way ANOVA did not indicate statistical significance between the currents measured without and with CUMS-treatment in response to any of the agonists. The number of individual animals is indicated in each column.

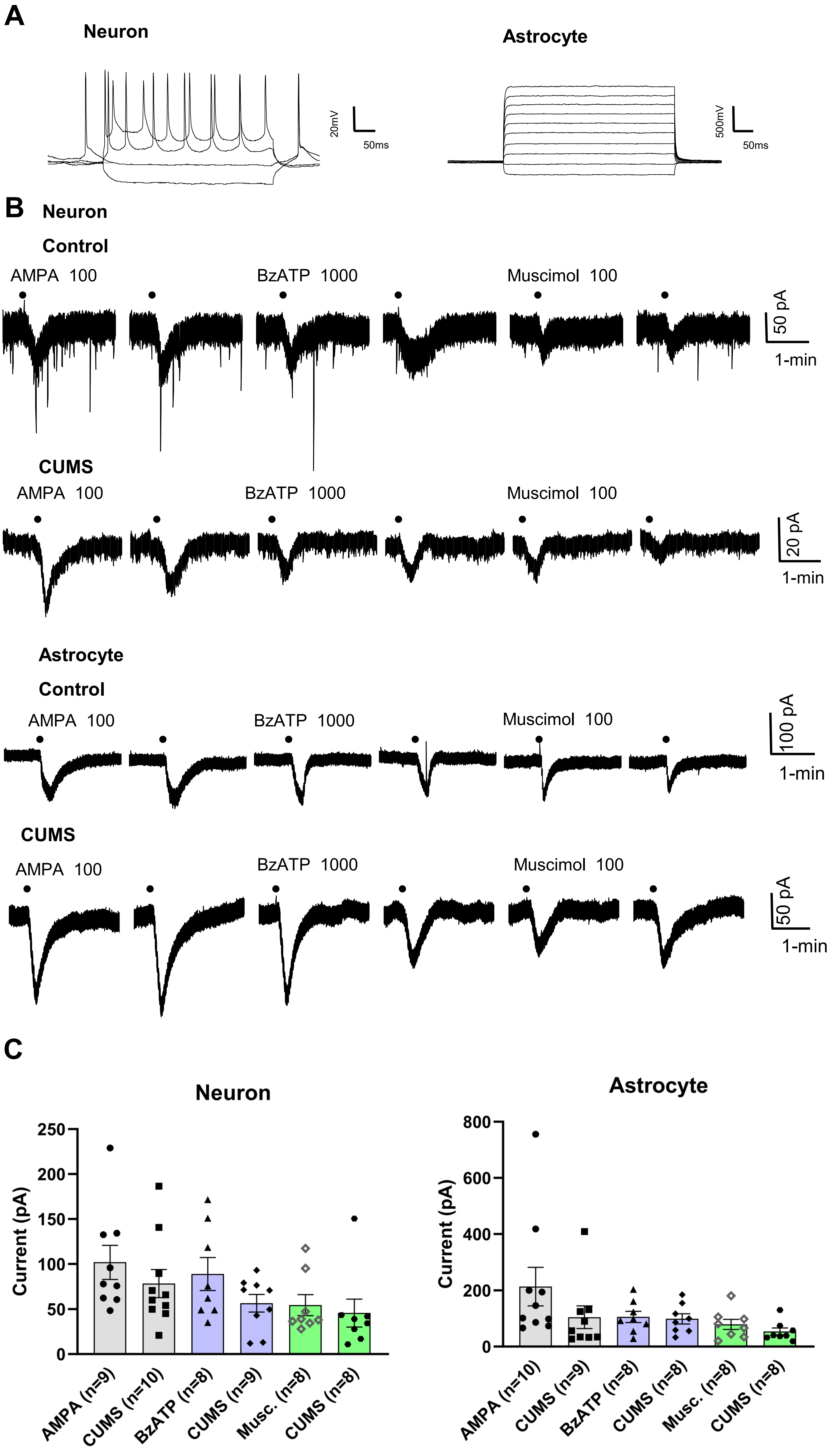

A
Neuron
20mV
50ms
Astrocyte
500mV
50ms
B
Neuron
Control
AMPA 100
BzATP 1000
Muscimol 100
50 pA
1-min
CUMS
AMPA 100
BzATP 1000
Muscimol 100
20 pA
1-min
Astrocyte
Control
AMPA 100
BzATP 1000
Muscimol 100
100 pA
1-min
CUMS
AMPA 100
BzATP 1000
Muscimol 100
50 pA
1-min
C
Neuron
Current (pA)
250
200
150
100
50
0
AMPA (n=9)
CUMS (n=10)
BzATP (n=8)
CUMS (n=9)
Musc. (n=8)
CUMS (n=8)
Astrocyte
Current (pA)
800
600
400
200
0
AMPA (n=10)
CUMS (n=9)
BzATP (n=8)
CUMS (n=8)
Musc. (n=8)
CUMS (n=8)

Suppl. Figure 4. A priory power analysis using G*Power (α=0.05, power=0.95; version 3.1.9.7) for one-way ANOVA (fixed effects, omnibus) based on the observed variability and group differences.

Fig 1. A

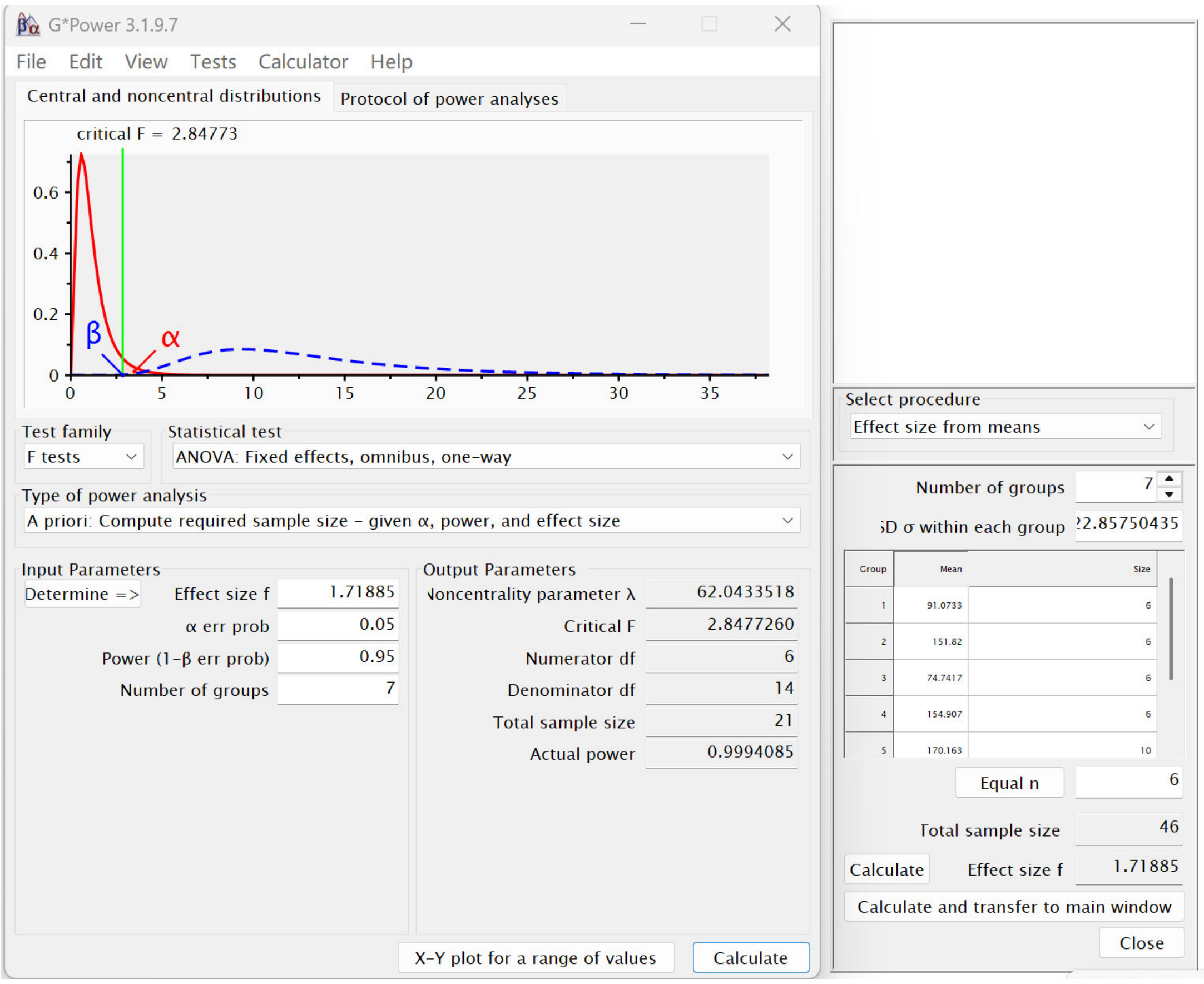

We performed an a priori power analysis using G*Power (version 3.1.9.7) for one-way ANOVA (fixed effects, omnibus), with α = 0.05 and power = 0.95. Based on pooled SD (SD=22.9), the estimated effect size was f = 1.7, indicating a minimum requirement of animals is 21. We included 46 mice in 7 groups, providing adequate statistical power.

Fig 1. B

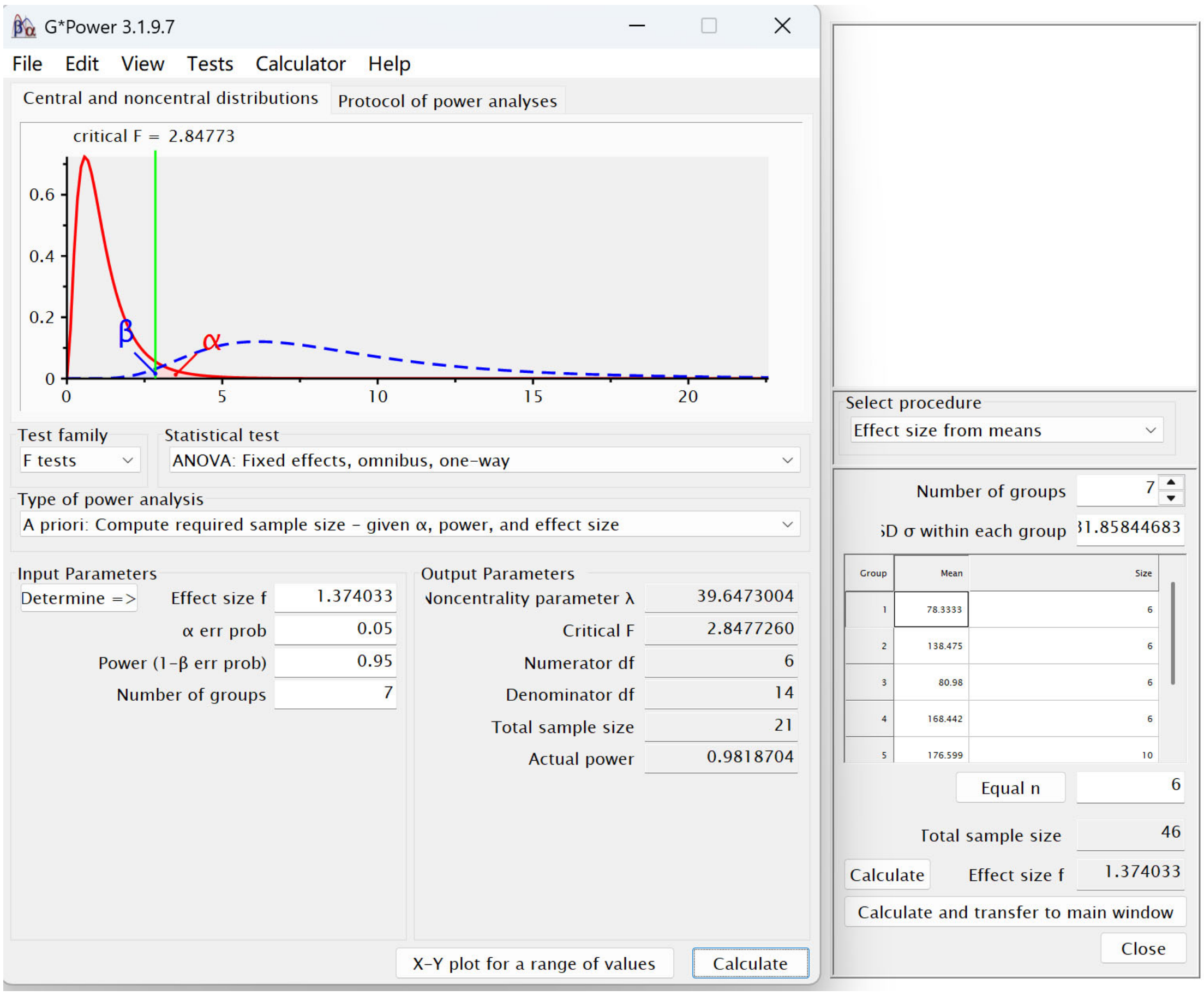

Sample size was estimated using G*Power with α=0.05 and power=0.95. Based on expected group means, pooled SD (SD=31.9) and the effect size (f=1.4), the total required sample size was 21. We used 46 mice and ensured a statistical power.

Fig 1.C

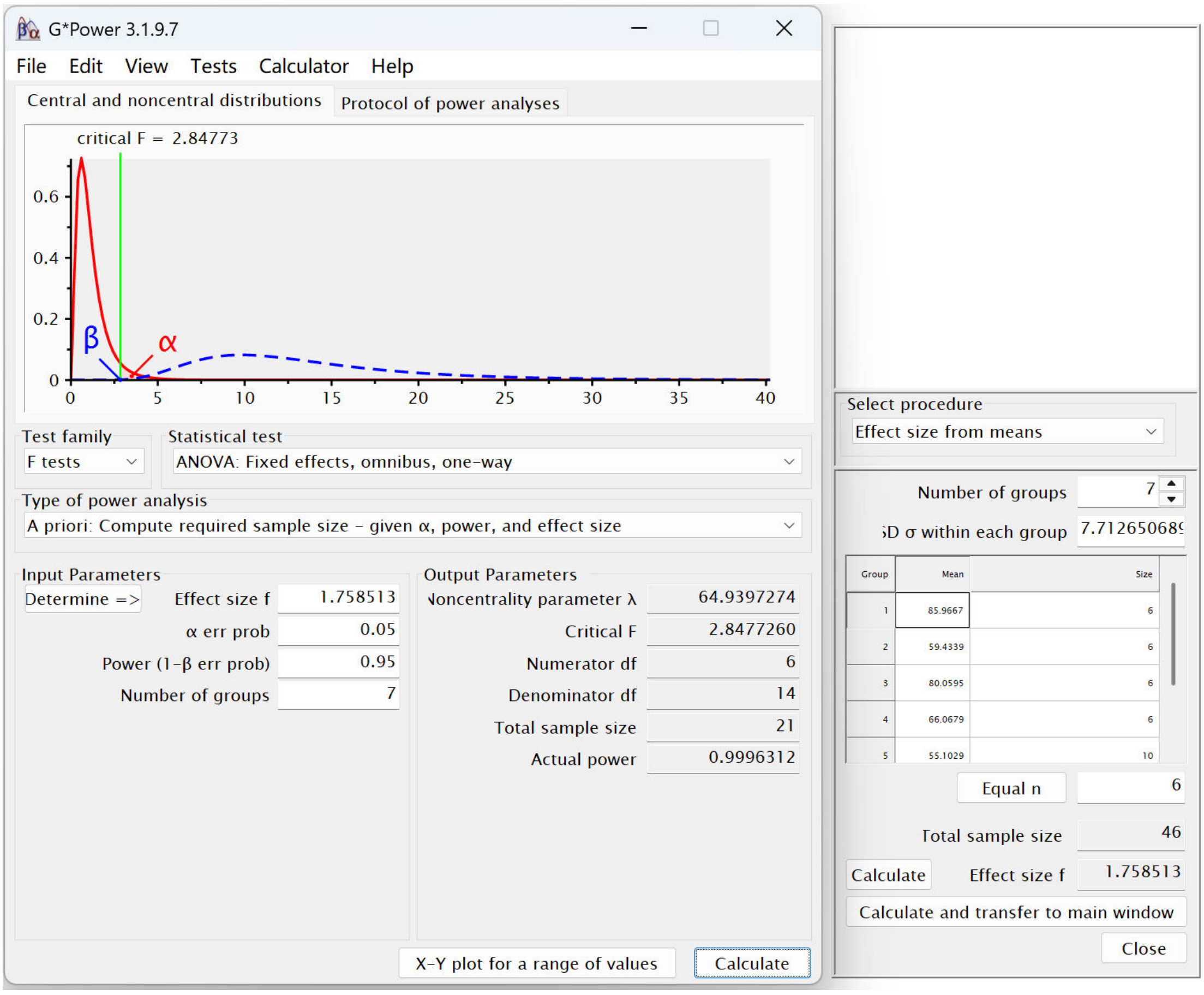

Sample size was estimated using G*Power with a two-sided α = 0.05 and desired power of 0.95. Expected pooled within-group standard deviation was SD = 7.7 and the effect size f = 1.8. The power analysis indicated that a minimum total sample size of n = 21 was required. We used 46 mice in 7 groups and ensured a statistical power.

Fig 1.D

This figure was shown as representative running traces in open field test, and we do not need to do power analysis.

Fig 1.E

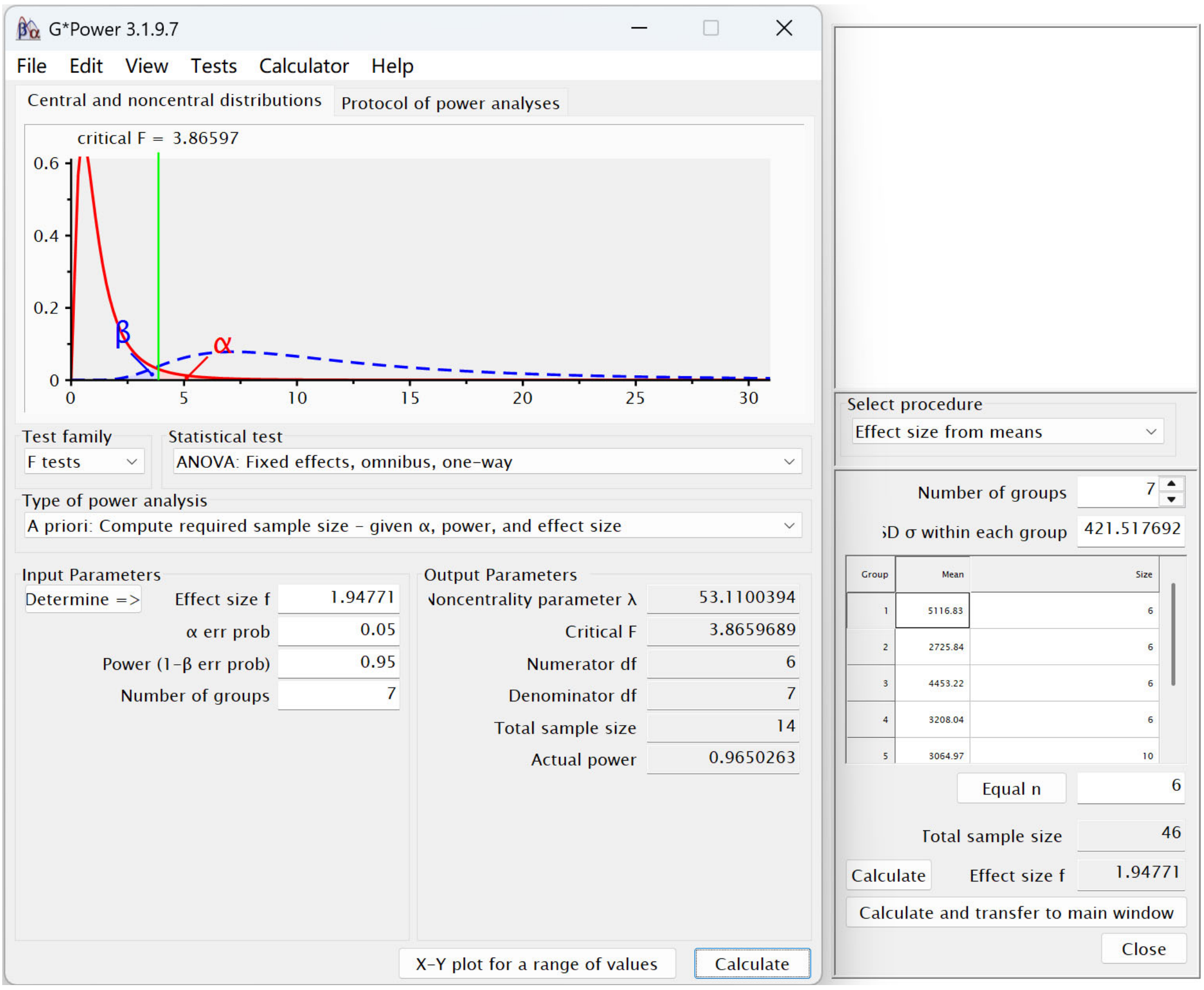

G*Power analysis (one-way ANOVA, α = 0.05, power = 0.95) under the condition of SD = 421.5 and the effect size f = 1.9 indicated that minimum total sample size of n = 14 was sufficient, whereas we used total n = 46 in 7 groups, ensuring adequate statistical power.

Fig 1.F

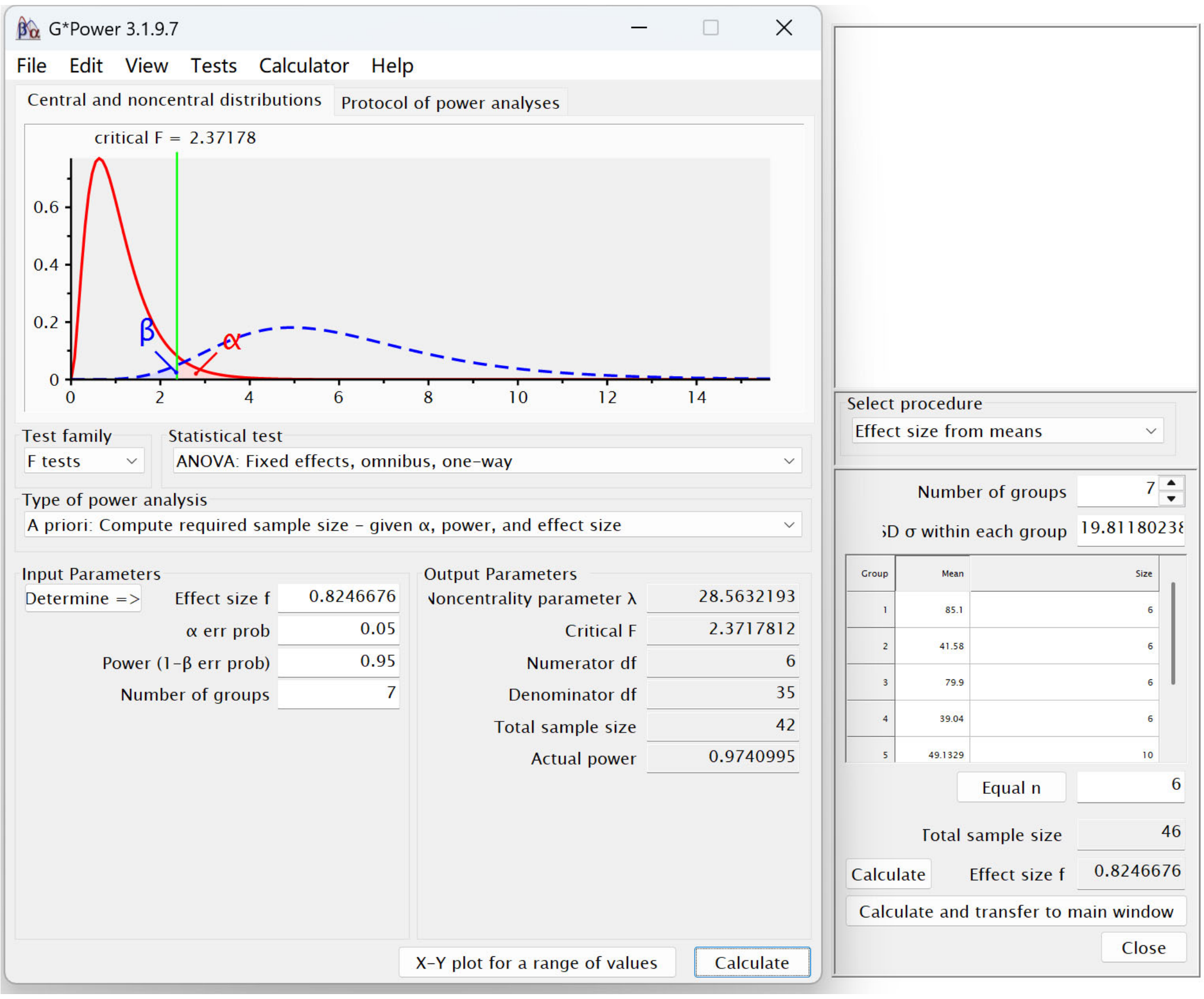

G*Power analysis (one-way ANOVA, α = 0.05, power = 0.95) under the condition of SD = 19.8 and the effect size f = 0.8 indicated that total n = 42 was sufficient, whereas we used total n = 46, ensuring adequate statistical power.

Fig 1.G

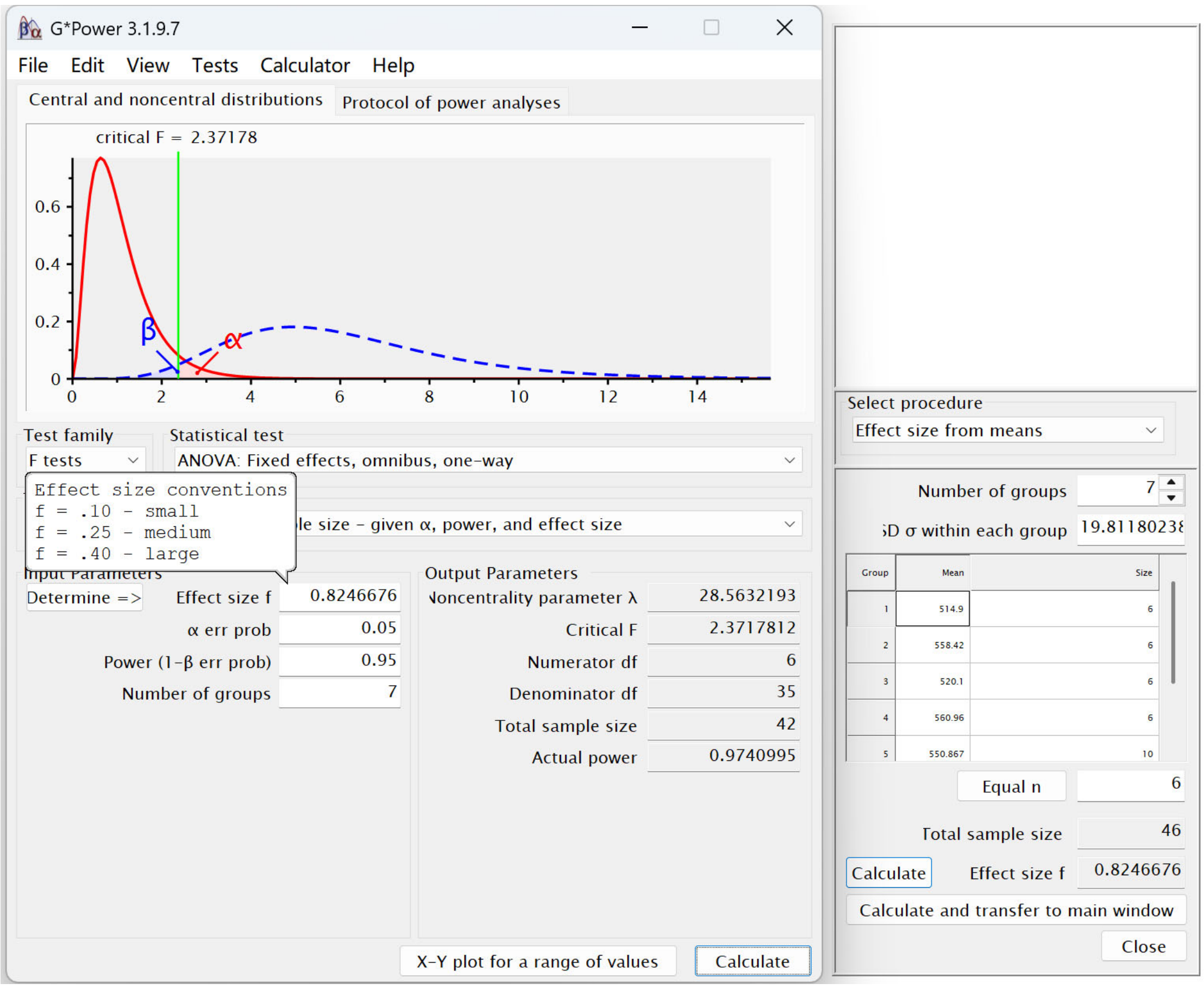

G*Power analysis (one-way ANOVA, α = 0.05, power = 0.95) under the condition of SD = 19.8 and the effect size f = 0.8 indicated that total n = 42 was sufficient, whereas we used total n = 46, ensuring adequate statistical power.

Fig 1.H

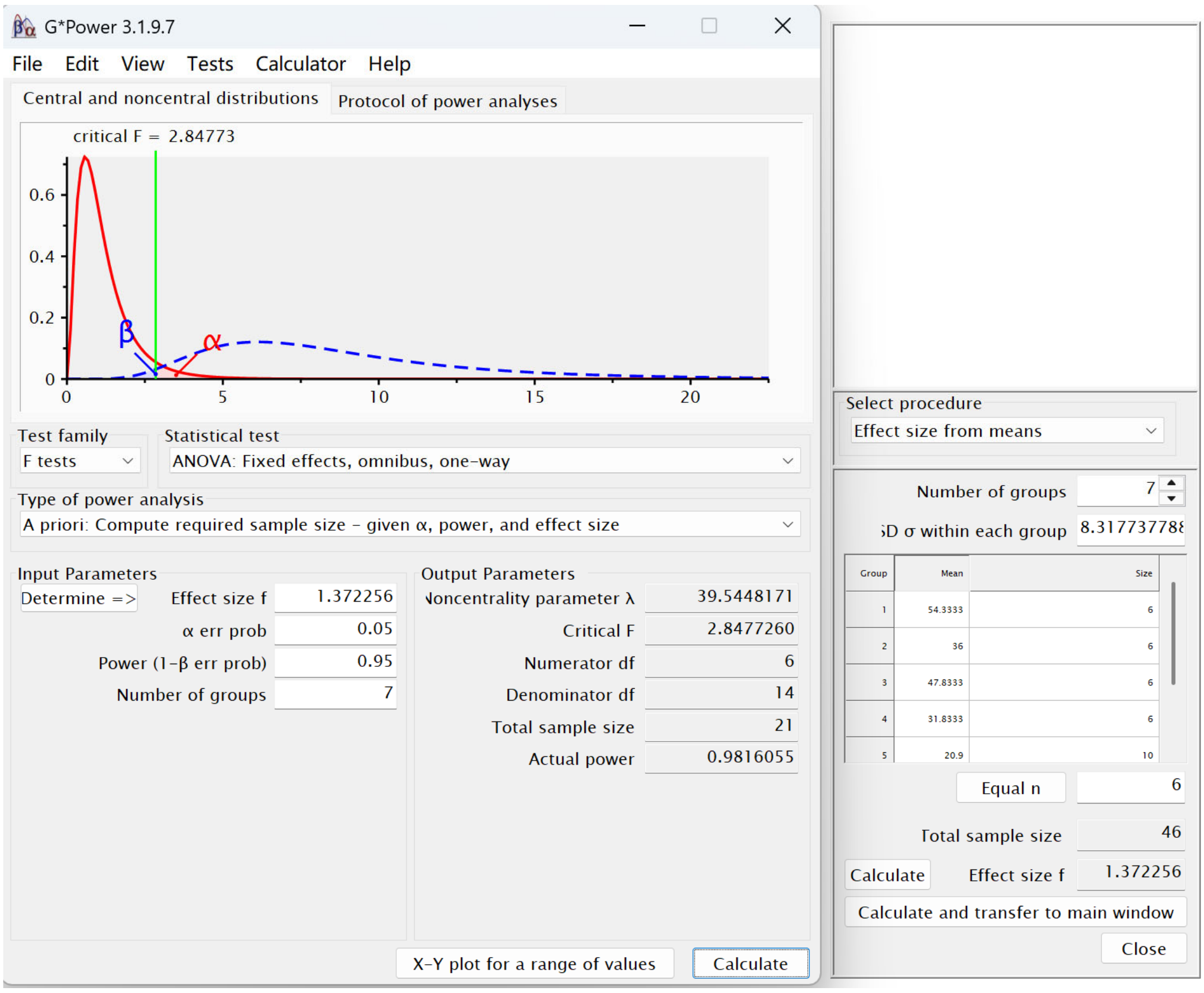

G*Power analysis (one-way ANOVA, α = 0.05, power = 0.95) under the condition of SD = 8.3 and the effect size f = 1.4 indicated that total n = 21 was sufficient, whereas we used total n = 46, ensuring adequate statistical power.

Fig 2.A

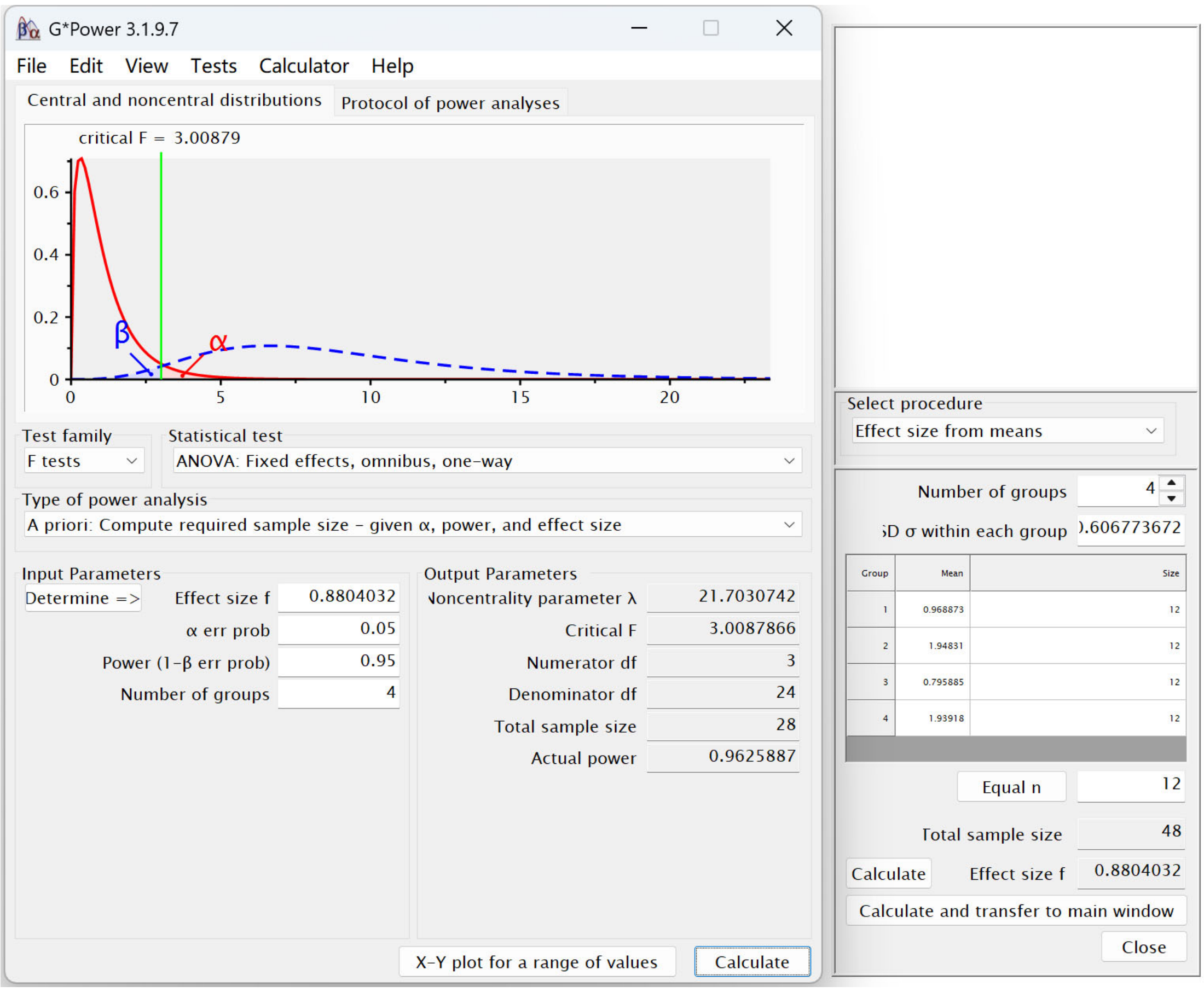

G*Power analysis (one-way ANOVA, α = 0.05, power = 0.95) under the condition of SD = 0.6 and the effect size f = 0.9 indicated that the total required sample size was 28 and we used 48 ensuring sufficient statistical power.

Fig 2.B

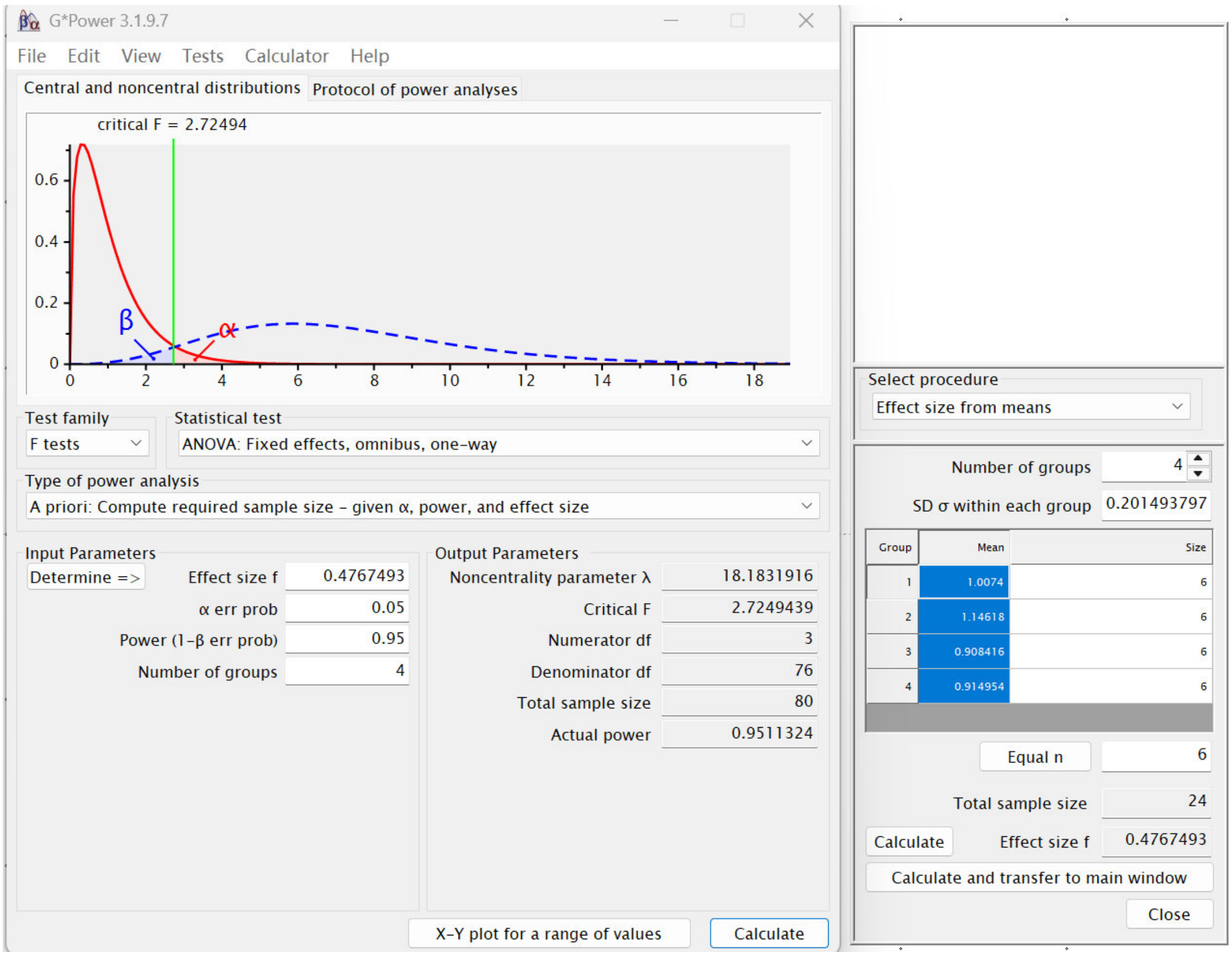

We performed an a priori power analysis using G*Power (α = 0.05, power=0.95) based on the observed variability and group differences. The analysis indicated that achieving a statistical power of 0.95 would require a total sample size of approximately 80 animals for cd39 mRNA expression. This large sample size requirement reflects the relatively modest biologic effect and inherent biological variability in the present model rather than a limitation in experimental design. Importantly, such a sample size is not feasible or ethically justified in animal research and is far beyond the standard practice in the field. Our sample size is consistent with previous studies using similar experimental models and endpoints. Therefore, the absence of statistical significance in our study likely reflects a modest or biologically limited effect rather than insufficient statistical power.

Fig 2.C

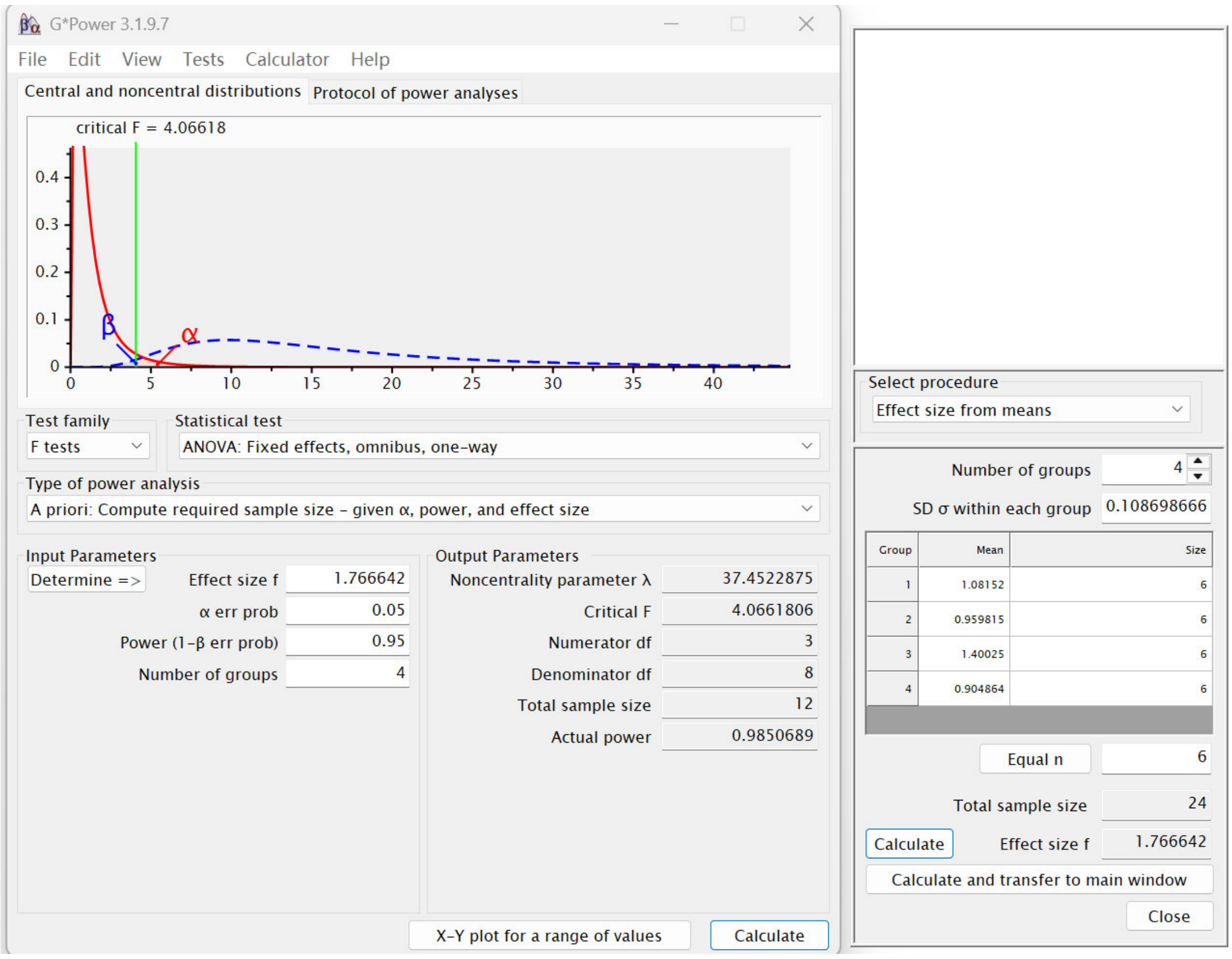

G*Power analysis (one-way ANOVA, α = 0.05, power = 0.95) under the condition of SD = 0.1087 and the effect size f = 1.767 indicated that the total required sample size was 12 and n = 3 per group was sufficient. We used n=6 per group.

Fig 2.D

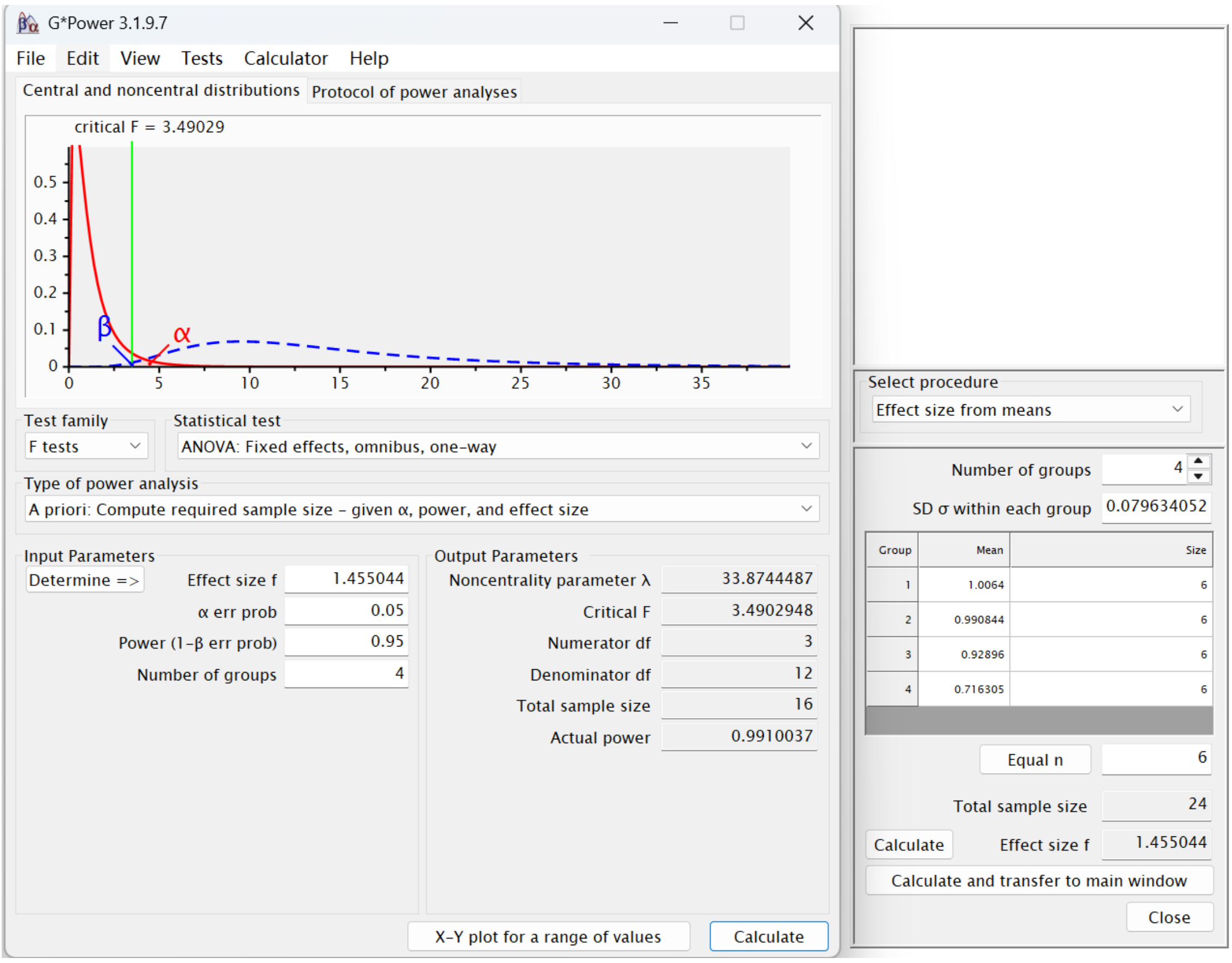

G*Power analysis (one-way ANOVA, α = 0.05, power = 0.95) under the condition of SD = 0.0796 and the effect size f = 1.455 indicated that the total required sample size was 16 and n = 4 per group was sufficient. We used n=6 per group.

Fig 2.E

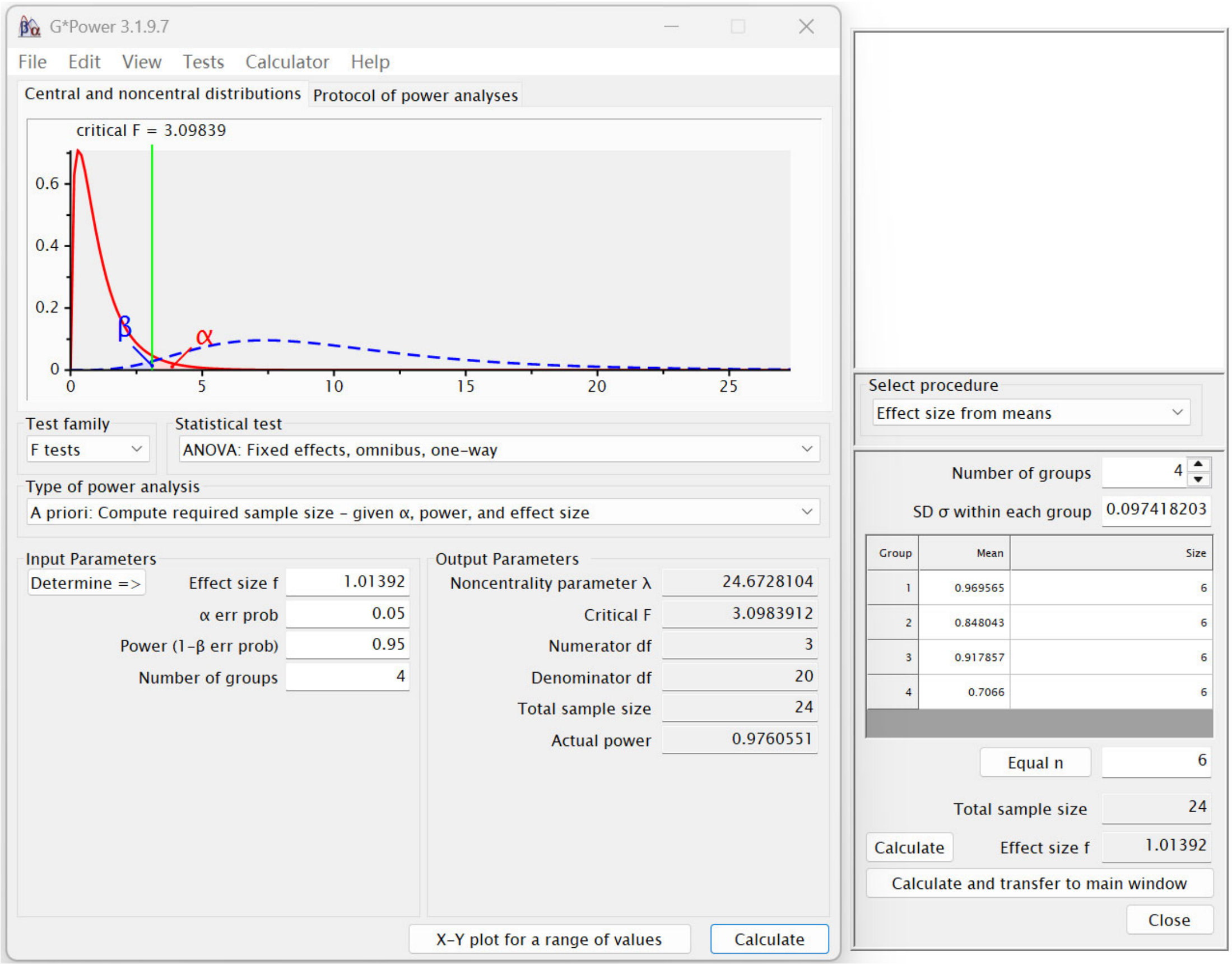

G*Power analysis (one-way ANOVA, α = 0.05, power = 0.95) under the condition of SD = 0.0974 and the effect size f = 1.013 indicated that the total required sample size was 24 and n = 6 per group was sufficient. We used n=6 per group.

Fig 2.F

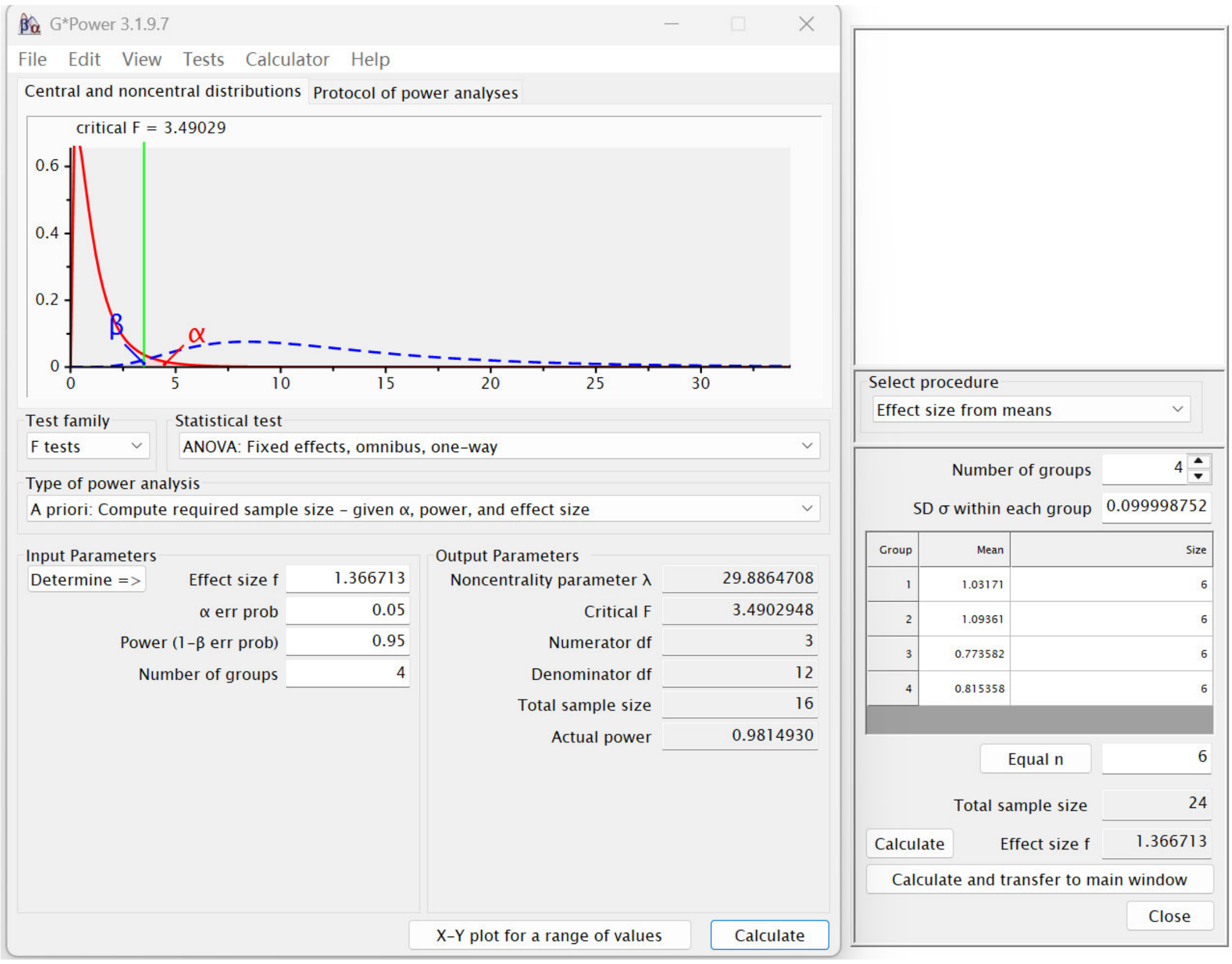

G*Power analysis (one-way ANOVA, α = 0.05, power = 0.95) under the condition of SD = 0.01 and the effect size f = 1.367 indicated that the total required sample size was 16 and n = 4 per group was sufficient. We used n=6 per group.

Fig 2.G

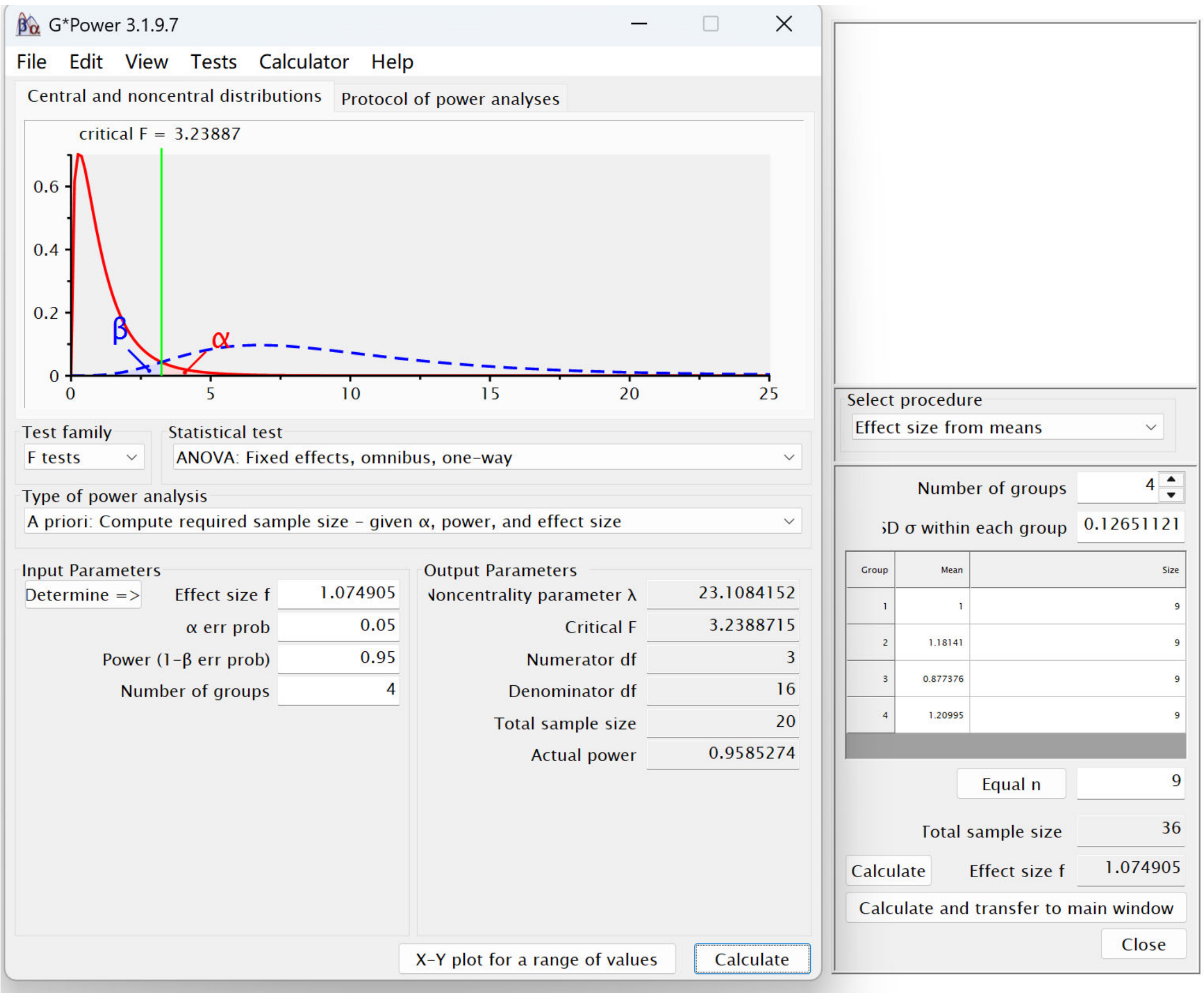

G*Power analysis (one-way ANOVA, α = 0.05, power = 0.95) under the condition of SD = 0.13 and the effect size f = 1.07 indicated that the total required sample size was 20 and n = 5 per group was sufficient. We used n = 9 per group.

Fig 2.H

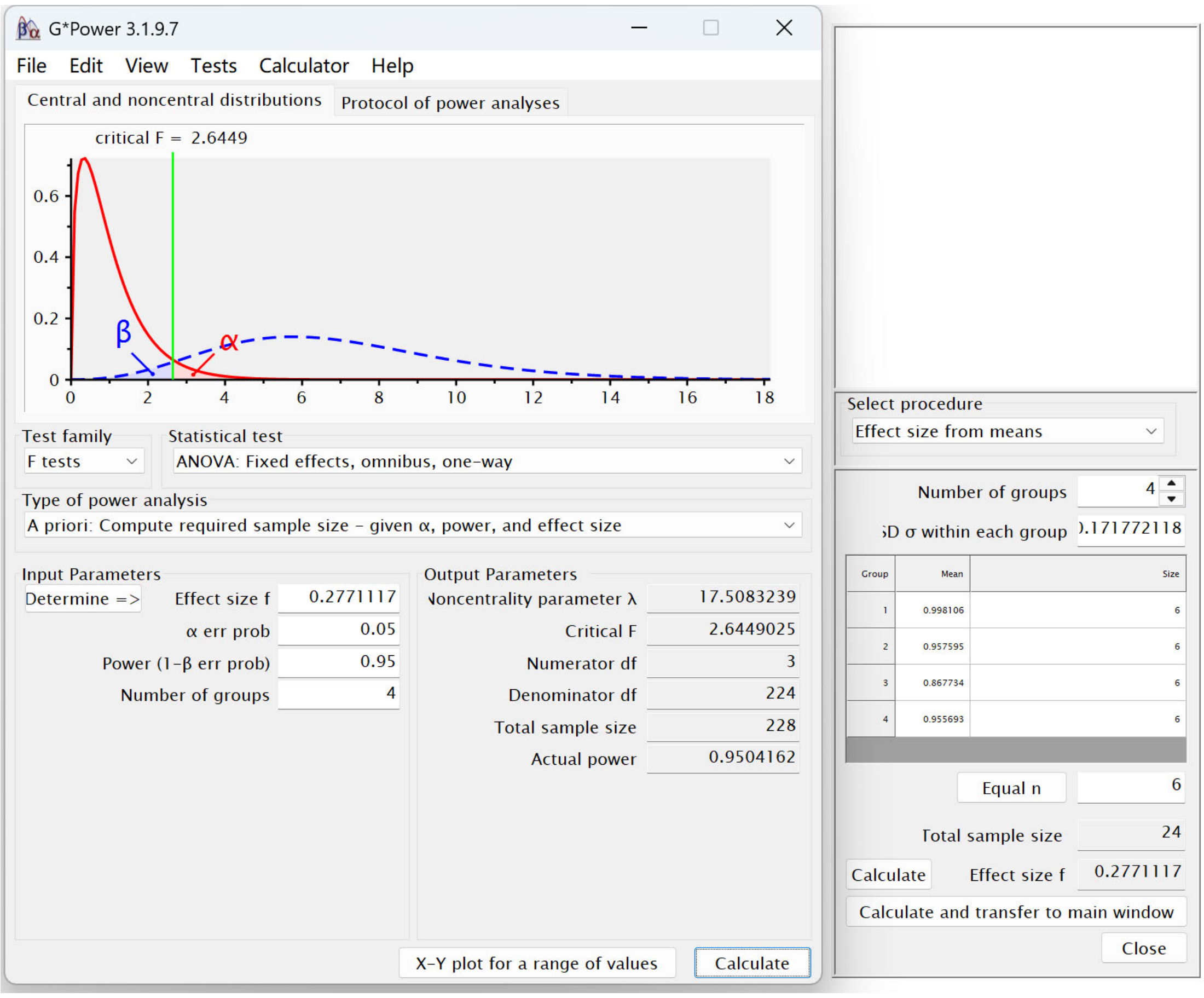

We performed an a priori power analysis using G*Power (α = 0.05, power=0.95) based on the observed variability and group differences. The analysis indicated that achieving a statistical power of 0.95 would require a total sample size of approximately 228 mice for CD39 protein expression. This large sample size requirement reflects the relatively modest biologic effect and inherent biological variability in the present model rather than a limitation in experimental design. Importantly, such a sample size is not feasible or ethically justified in animal research and is far beyond the standard practice in the field. Our sample size is consistent with even more than previous studies using similar experimental models and endpoints[1,2]. Therefore, the absence of statistical significance in our study likely reflects a modest or biologically limited effect rather than insufficient statistical power.

[1] Shu Y, Liu D, Jiang Y, et al. Compound Chaijin Jieyu Fang alleviates insomnia complicated with depression by reshaping actinobacteria to inhibit neuronal apoptosis. *Phytomedicine*. 2026;150:157632. doi:10.1016/j.phymed.2025.157632.

[2] Wang Y, Yu HM, He Y, et al. IL-17A in the hippocampus regulates despair-like behaviors via inhibitory synaptic transmission. *Acta Pharmacol Sin*. Published online March 17, 2026. doi:10.1038/s41401-026-01761-5.

Fig 2.I

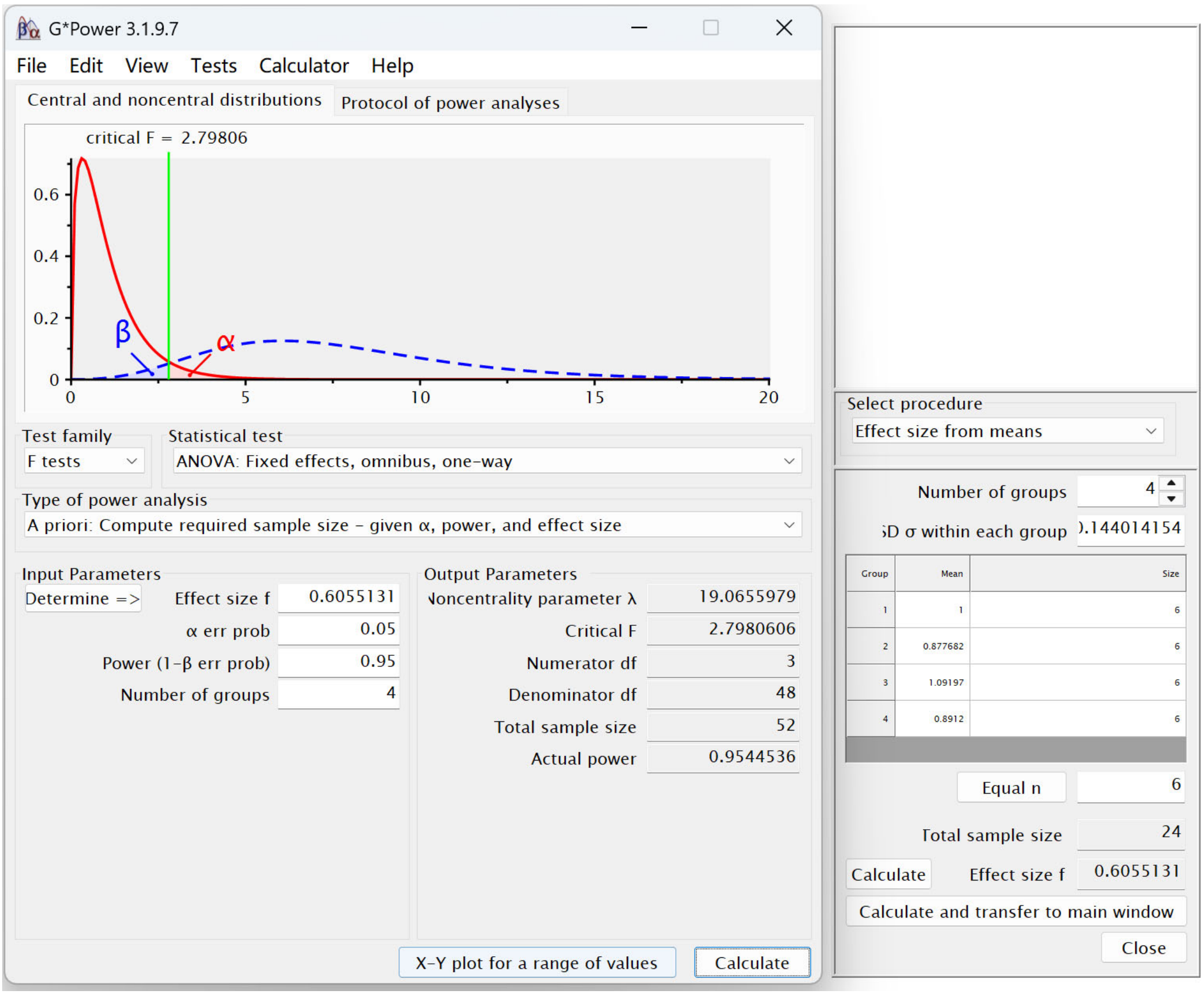

We performed an a priori power analysis using G*Power (α = 0.05, power=0.95) based on the observed variability and group differences. The analysis indicated that achieving a statistical power of 0.95 would require a total sample size of approximately 52 animals for CD73 protein expression. This large sample size requirement reflects the relatively modest biologic effect and inherent biological variability in the present model rather than a limitation in experimental design. Importantly, such a sample size is not feasible or ethically justified in animal research and is far beyond the standard practice in the field. Our sample size is consistent with previous studies using similar experimental models and endpoints[1,2]. Therefore, the absence of statistical significance in our study likely reflects a modest or biologically limited effect rather than insufficient statistical power.

[1] Shu Y, Liu D, Jiang Y, et al. Compound Chaijin Jieyu Fang alleviates insomnia complicated with depression by reshaping actinobacteria to inhibit neuronal apoptosis. *Phytomedicine*. 2026;150:157632. doi:10.1016/j.phymed.2025.157632.

[2] Wang Y, Yu HM, He Y, et al. IL-17A in the hippocampus regulates despair-like behaviors via inhibitory synaptic transmission. *Acta Pharmacol Sin*. Published online March 17, 2026. doi:10.1038/s41401-026-01761-5.

Fig 2.J

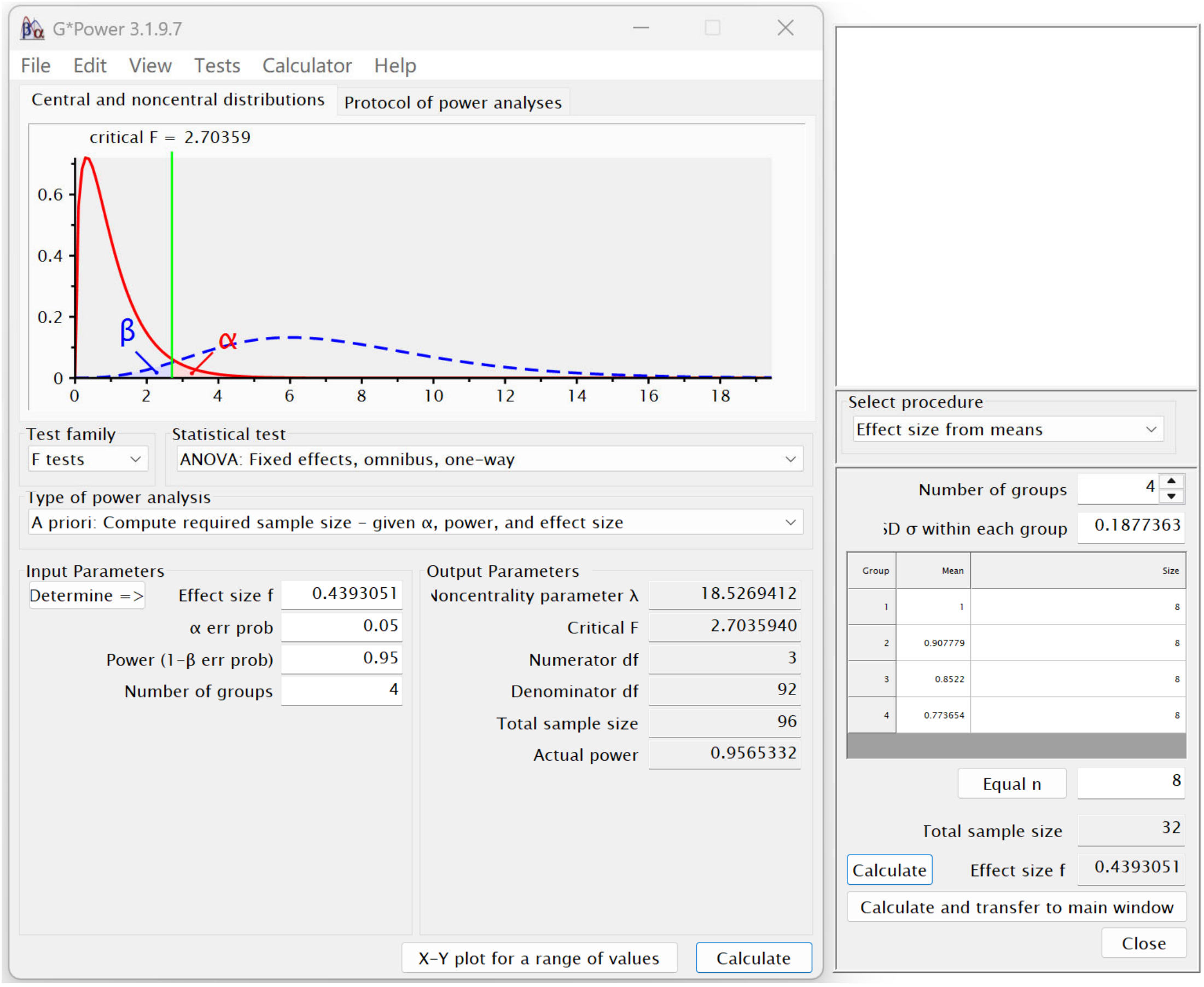

We performed an a priori power analysis using G*Power (α = 0.05, power=0.95) based on the observed variability and group differences. The analysis indicated that achieving a statistical power of 0.95 would require a total sample size of approximately 96 animals for SNAP23 protein expression. This large sample size requirement reflects the relatively modest biologic effect and inherent biological variability in the present model rather than a limitation in experimental design. Importantly, such a sample size is not feasible or ethically justified in animal research and is far beyond the standard

practice in the field. Our sample size is consistent with previous studies using similar experimental models and endpoints[1,2]. Therefore, the absence of statistical significance in our study likely reflects a modest or biologically limited effect rather than insufficient statistical power.

[1] Shu Y, Liu D, Jiang Y, et al. Compound Chaijin Jieyu Fang alleviates insomnia complicated with depression by reshaping actinobacteria to inhibit neuronal apoptosis. *Phytomedicine*. 2026;150:157632. doi:10.1016/j.phymed.2025.157632.

[2] Wang Y, Yu HM, He Y, et al. IL-17A in the hippocampus regulates despair-like behaviors via inhibitory synaptic transmission. *Acta Pharmacol Sin*. Published online March 17, 2026. doi:10.1038/s41401-026-01761-5.

Fig 2.K

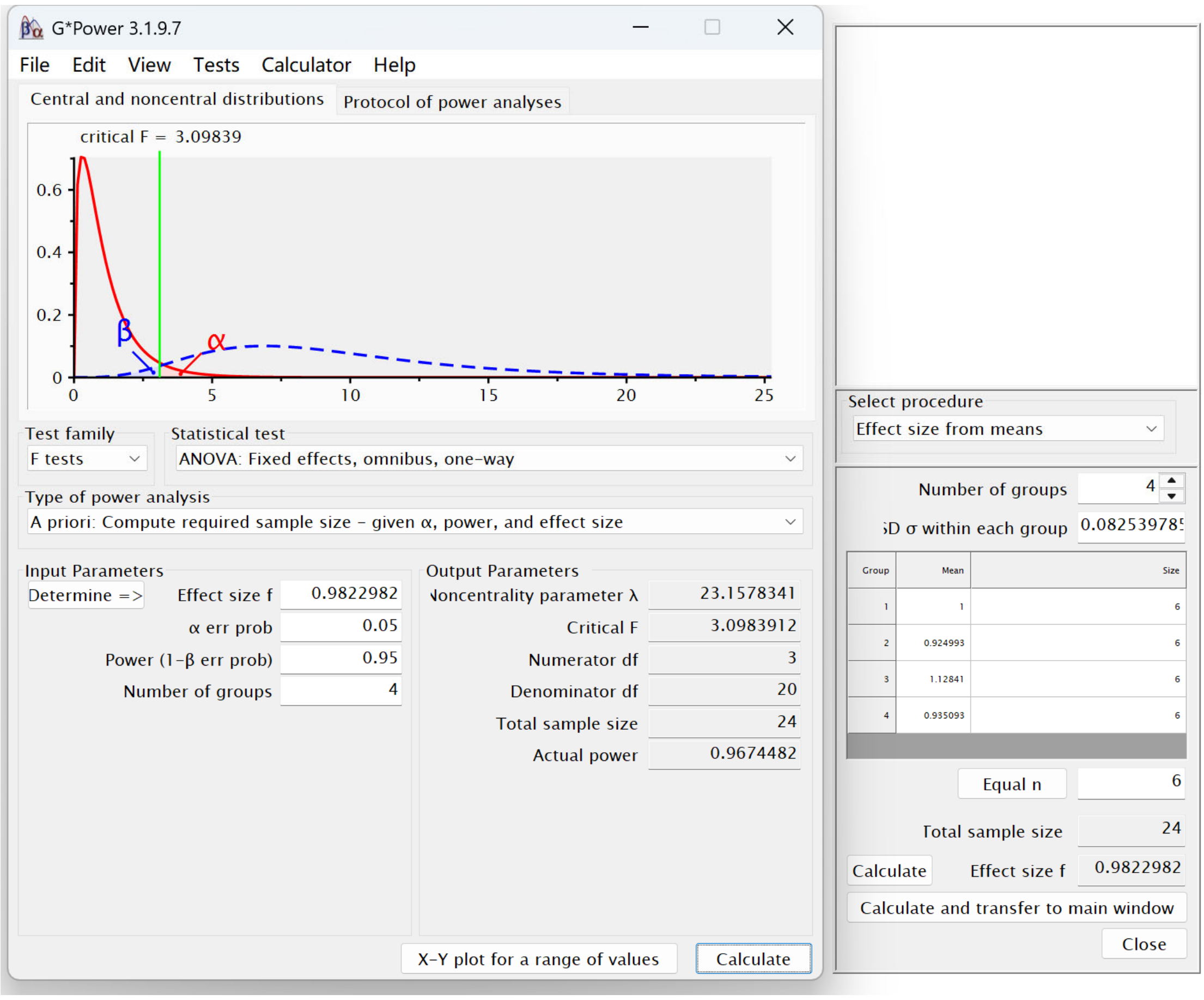

G*Power analysis (one-way ANOVA, α = 0.05, power = 0.95) under the condition of SD = 0.08 and the effect size f = 0.98 indicated that the total required sample size was 24 and n = 6 per group was sufficient. We used n = 6 per group.

Fig 2.L

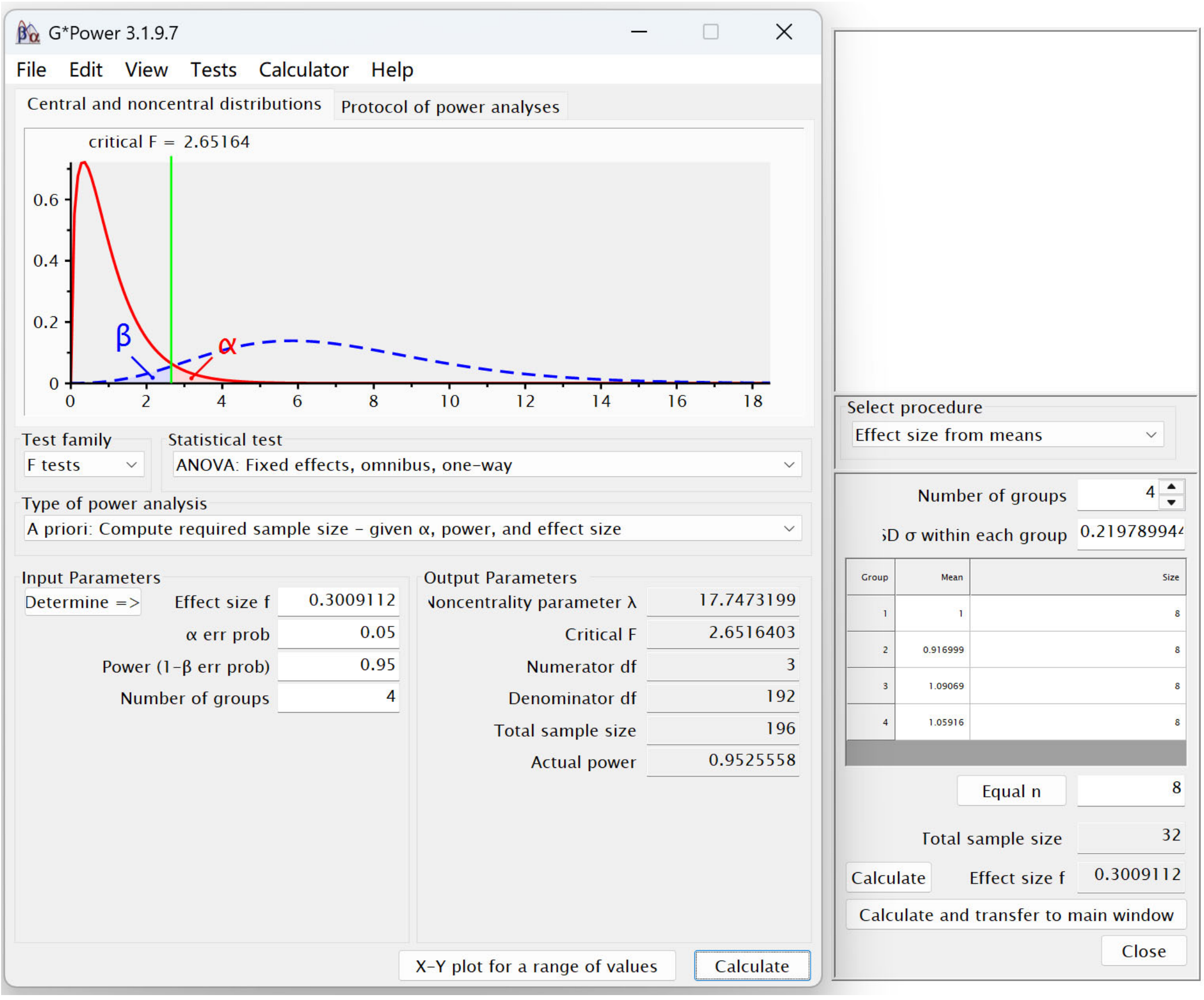

We performed an a priori power analysis using G*Power (α = 0.05, power=0.95) based on the observed variability and group differences. The analysis indicated that achieving a statistical power of 0.95 would require a total sample size of approximately 196 animals for CALHM2 protein expression. This large sample size requirement reflects the relatively modest biologic effect and inherent biological variability in the present model rather than a limitation in experimental design. Importantly, such a sample size is not feasible or ethically justified in animal research and is far beyond the standard practice in the field. Our sample size is consistent with previous studies using similar experimental models and endpoints[1,2]. Therefore, the absence of statistical significance in our study likely reflects a modest or biologically limited effect rather than insufficient statistical power.

[1] Shu Y, Liu D, Jiang Y, et al. Compound Chaijin Jieyu Fang alleviates insomnia complicated with depression by reshaping actinobacteria to inhibit neuronal apoptosis. *Phytomedicine*. 2026;150:157632. doi:10.1016/j.phymed.2025.157632.

[2] Wang Y, Yu HM, He Y, et al. IL-17A in the hippocampus regulates despair-like behaviors via inhibitory synaptic transmission. *Acta Pharmacol Sin*. Published online March 17, 2026. doi:10.1038/s41401-026-01761-5.

Fig 2.M

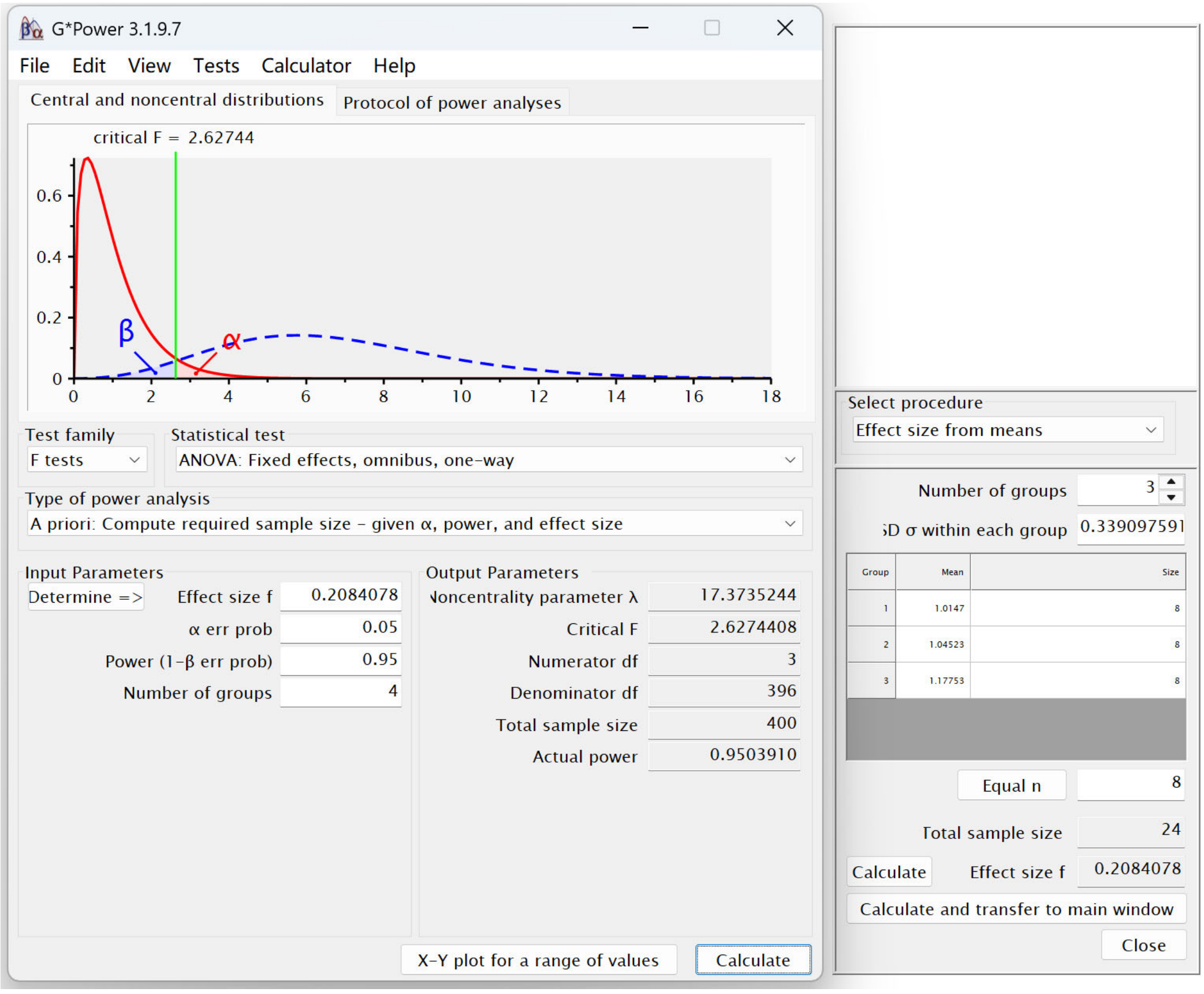

We performed an a priori power analysis using G*Power (α = 0.05, power=0.95) based on the observed variability and group differences. The analysis indicated that achieving a statistical power of 0.95 would require a total sample size of approximately 400 animals for P2X7 mRNA expression after EA and shamEA treated control mice. This large sample size requirement reflects the relatively modest biologic effect and inherent biological variability in the present model rather than a limitation in experimental design. Importantly, such a sample size is not feasible or ethically justified in animal research and is far beyond the standard practice in the field. Our sample size is consistent with previous studies using similar experimental models and endpoints[1,2]. Therefore, the absence of statistical significance in our study likely reflects a modest or biologically limited effect

rather than insufficient statistical power.

[1] Shu Y, Liu D, Jiang Y, et al. Compound Chaijin Jieyu Fang alleviates insomnia complicated with depression by reshaping actinobacteria to inhibit neuronal apoptosis. *Phytomedicine*. 2026;150:157632. doi:10.1016/j.phymed.2025.157632.

[2] Wang Y, Yu HM, He Y, et al. IL-17A in the hippocampus regulates despair-like behaviors via inhibitory synaptic transmission. *Acta Pharmacol Sin*. Published online March 17, 2026. doi:10.1038/s41401-026-01761-5.

Fig 2.N

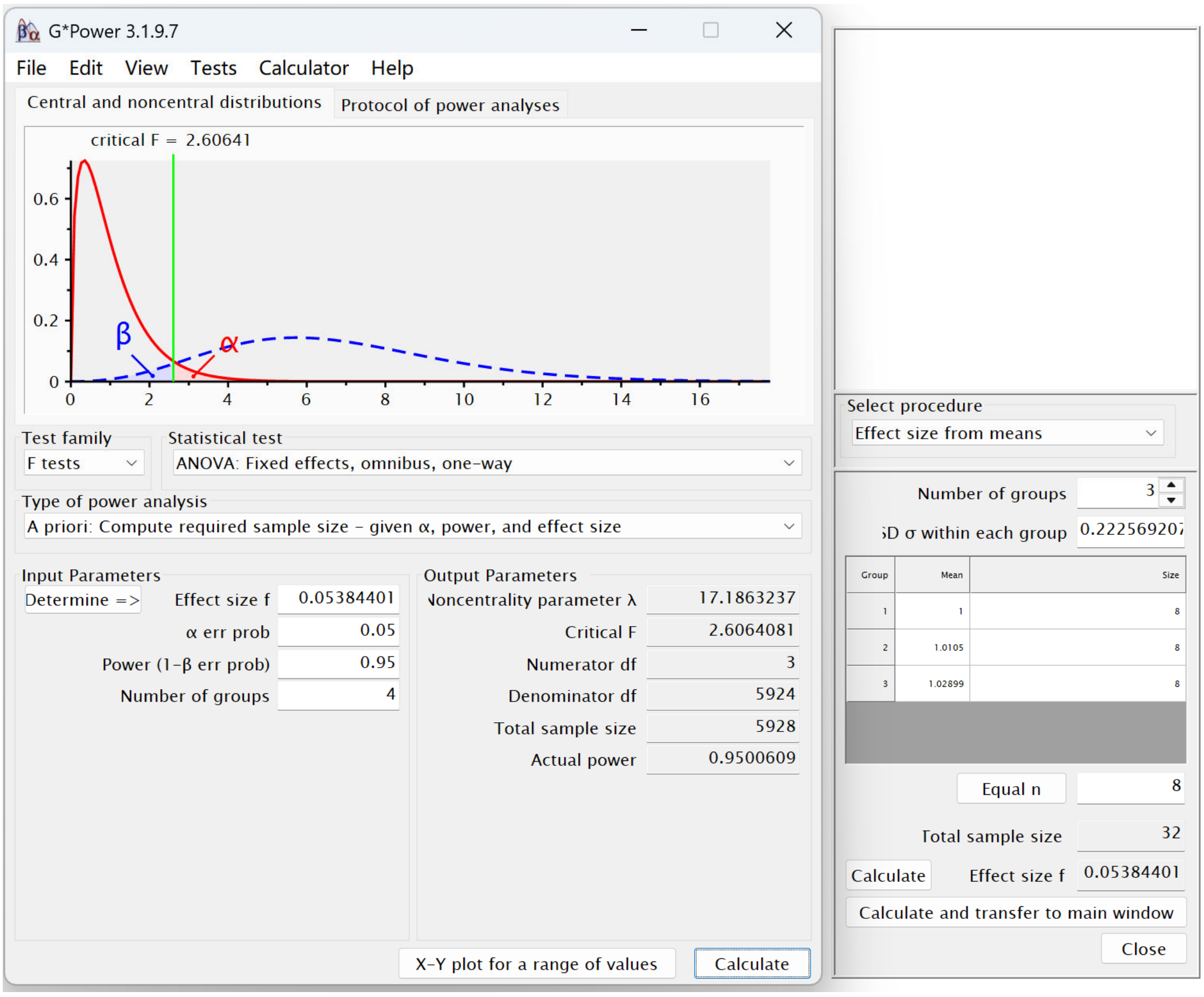

We performed an a priori power analysis using G*Power (α = 0.05, power=0.95) based on the observed variability and group differences. The analysis indicated that achieving a statistical power of 0.95 would require a total sample size of approximately 5928 mice for P2X7 protein expression after EA and shamEA treated control mice. This large sample size requirement reflects the relatively modest biologic effect and inherent biological variability in the present model rather than a limitation in

experimental design. Importantly, such a sample size is not feasible or ethically justified in animal research and is far beyond the standard practice in the field. Our sample size is consistent with previous studies using similar experimental models and endpoints[1,2]. Therefore, the absence of statistical significance in our study likely reflects a modest or biologically limited effect rather than insufficient statistical power.

[1] Shu Y, Liu D, Jiang Y, et al. Compound Chaijin Jieyu Fang alleviates insomnia complicated with depression by reshaping actinobacteria to inhibit neuronal apoptosis. *Phytomedicine*. 2026;150:157632. doi:10.1016/j.phymed.2025.157632.

[2] Wang Y, Yu HM, He Y, et al. IL-17A in the hippocampus regulates despair-like behaviors via inhibitory synaptic transmission. *Acta Pharmacol Sin*. Published online March 17, 2026. doi:10.1038/s41401-026-01761-5.

Fig 3.A

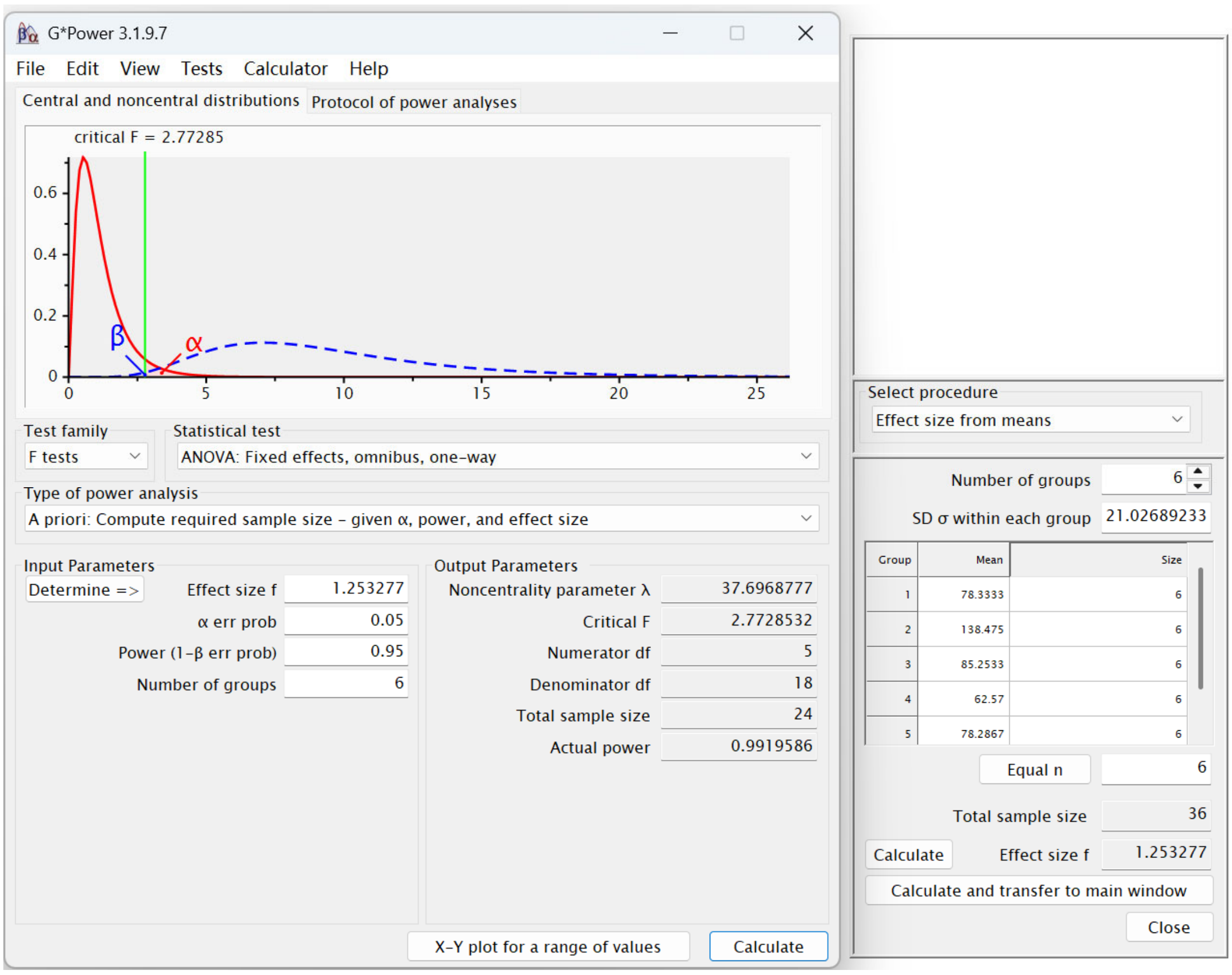

G*Power analysis (one-way ANOVA, α = 0.05, power = 0.95) under the condition of SD = 21.03 and the effect size f = 1.253 indicated that the total

required sample size was 24 and n = 4 per group was sufficient. We used n=6 per group.

Fig 3.B

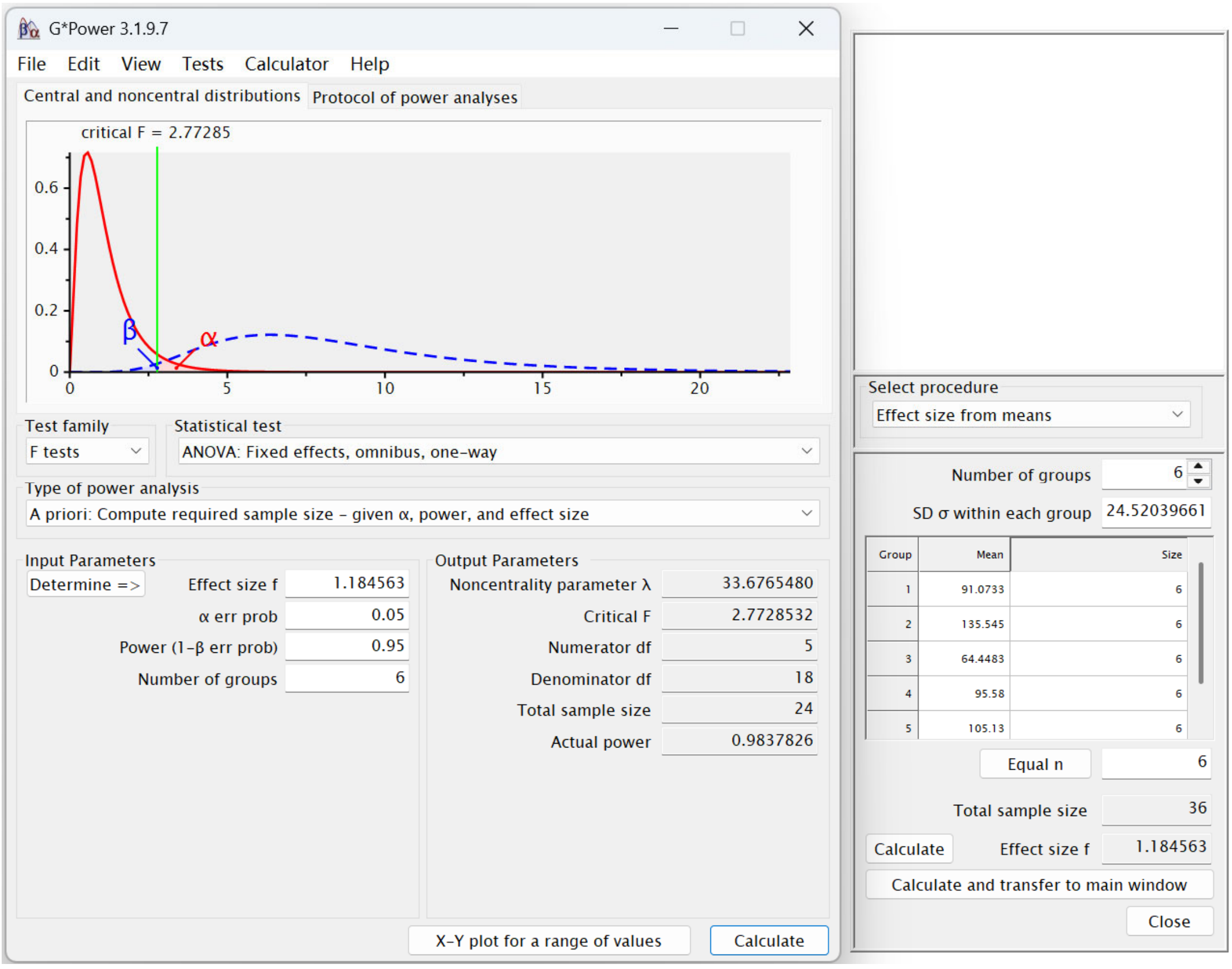

G*Power analysis (one-way ANOVA, α = 0.05, power = 0.95) under the condition of SD = 24.52 and the effect size f = 1.185 indicated that the total required sample size was 24 and n = 4 per group was sufficient. We used n=6 per group.

Fig 3.C

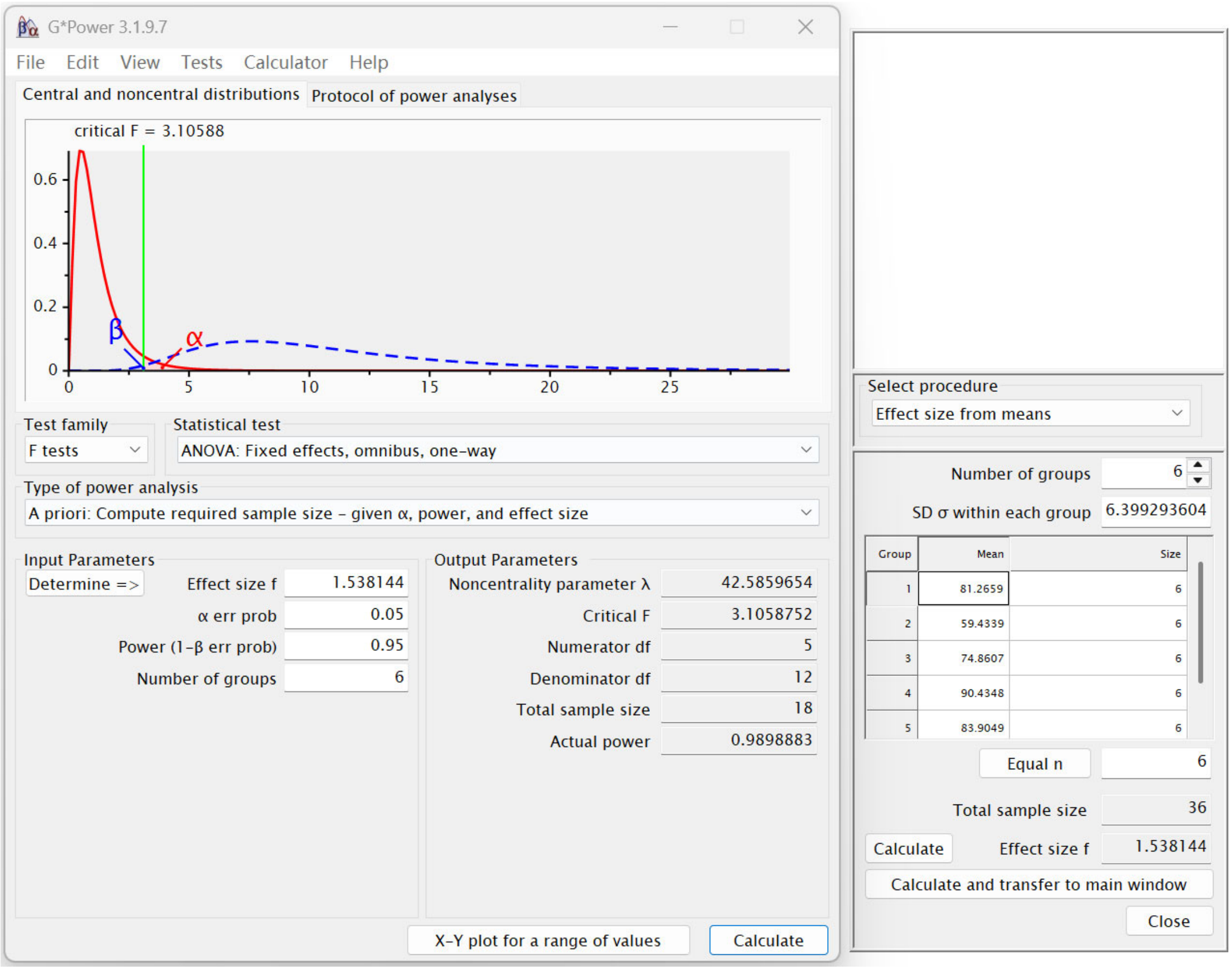

G*Power analysis (one-way ANOVA, α = 0.05, power = 0.95) under the condition of SD = 6.4 and the effect size f = 1.538 indicated that the total required sample size was 18 and n = 3 per group was sufficient. We used n=6 per group.

Fig 3.D

This figure was shown as representative running traces in open field test, and we do not need to do power analysis.

Fig 3.E

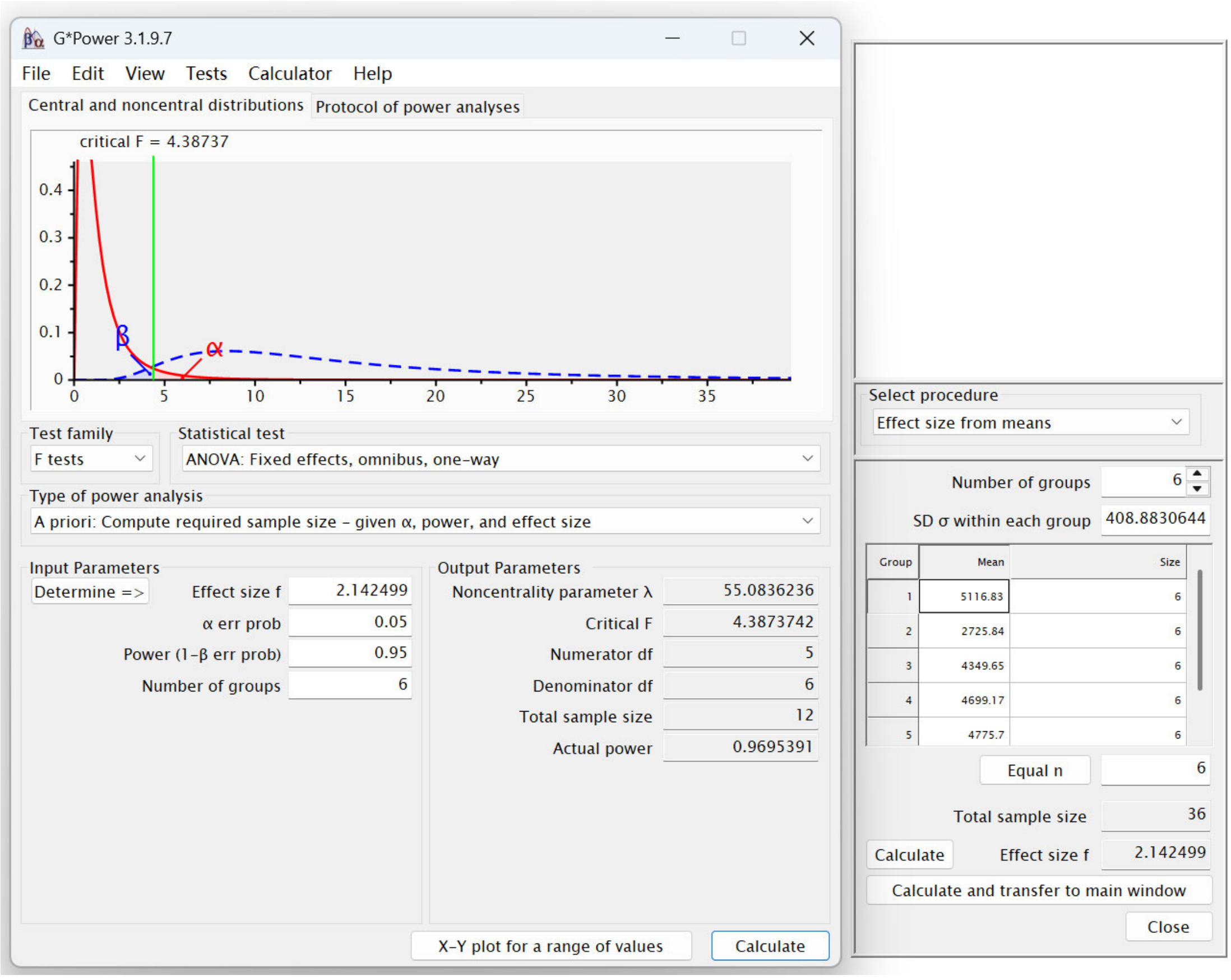

G*Power analysis (one-way ANOVA, α = 0.05, power = 0.95) under the condition of SD = 408.9 and the effect size f = 2.14 indicated that the total required sample size was 12. We used 36.

Fig 3.F

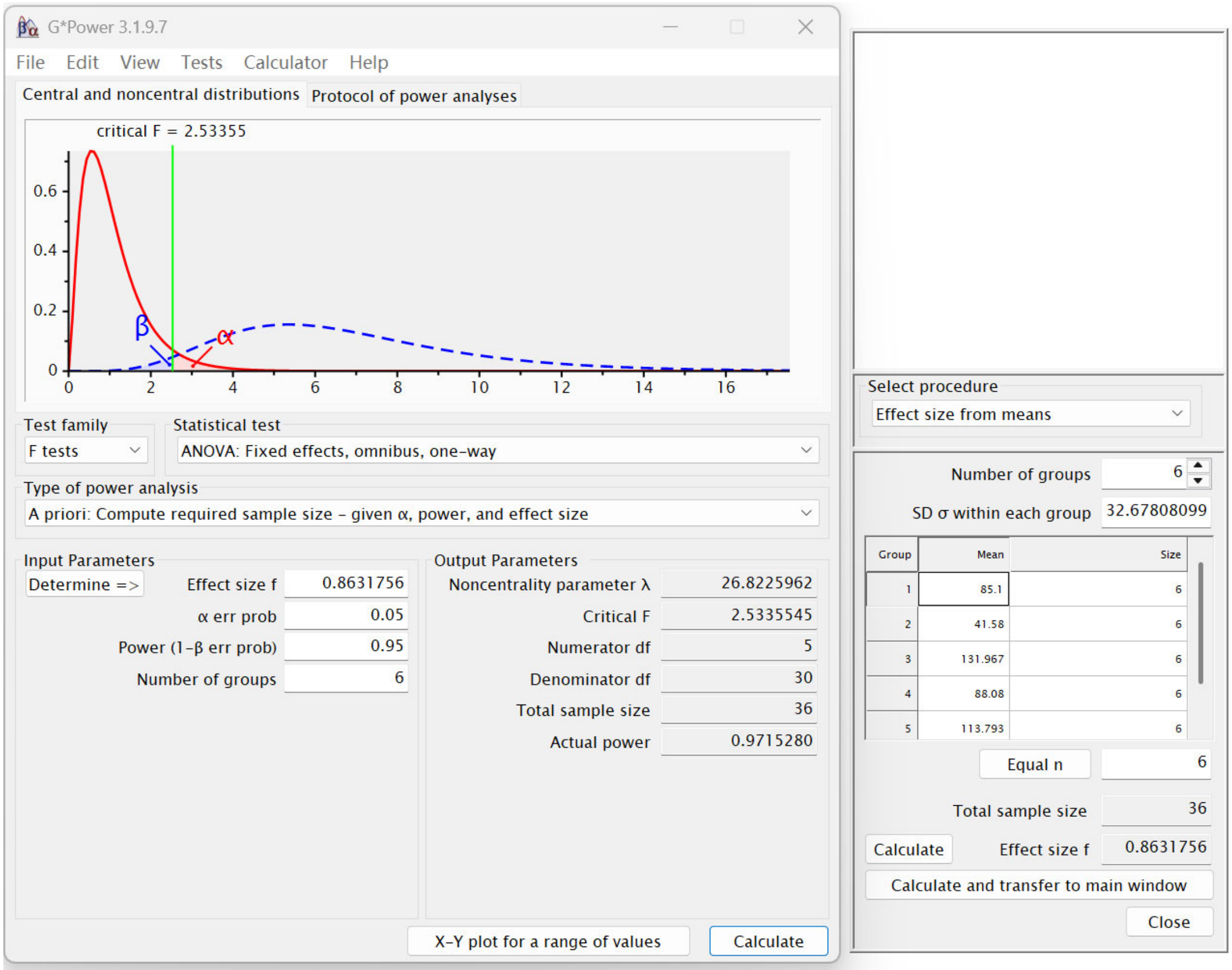

G*Power analysis (one-way ANOVA, α = 0.05, power = 0.95) under the condition of SD = 32.7 and the effect size f = 0.86 indicated that the total required sample size was 36 and n = 6 per group was sufficient. We used n=6 per group.

Fig 3.G

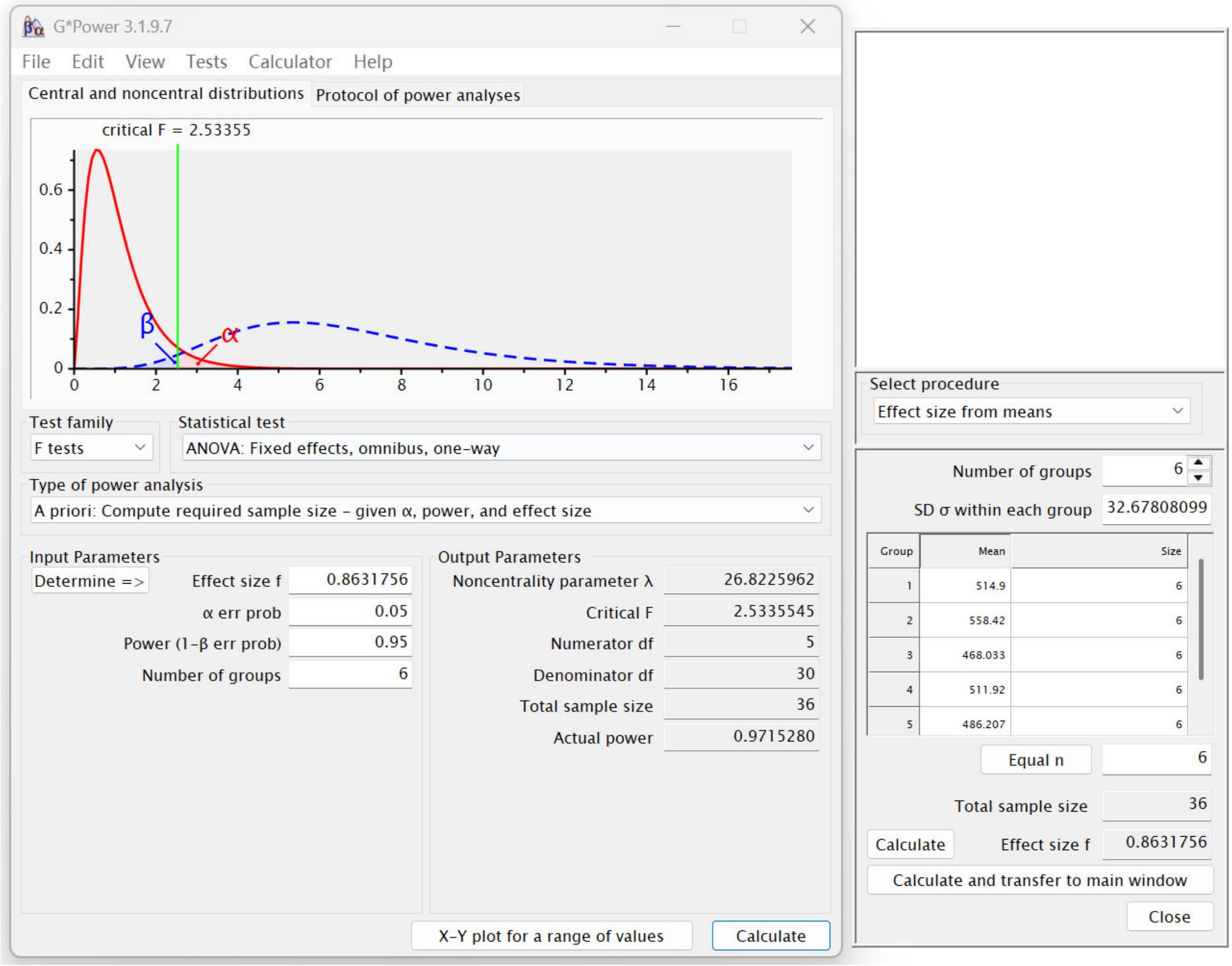

G*Power analysis (one-way ANOVA, α = 0.05, power = 0.95) under the condition of SD = 32.7 and the effect size f = 0.86 indicated that the total required sample size was 36 and n = 6 per group was sufficient. We used n=6 per group.

Fig 3.H

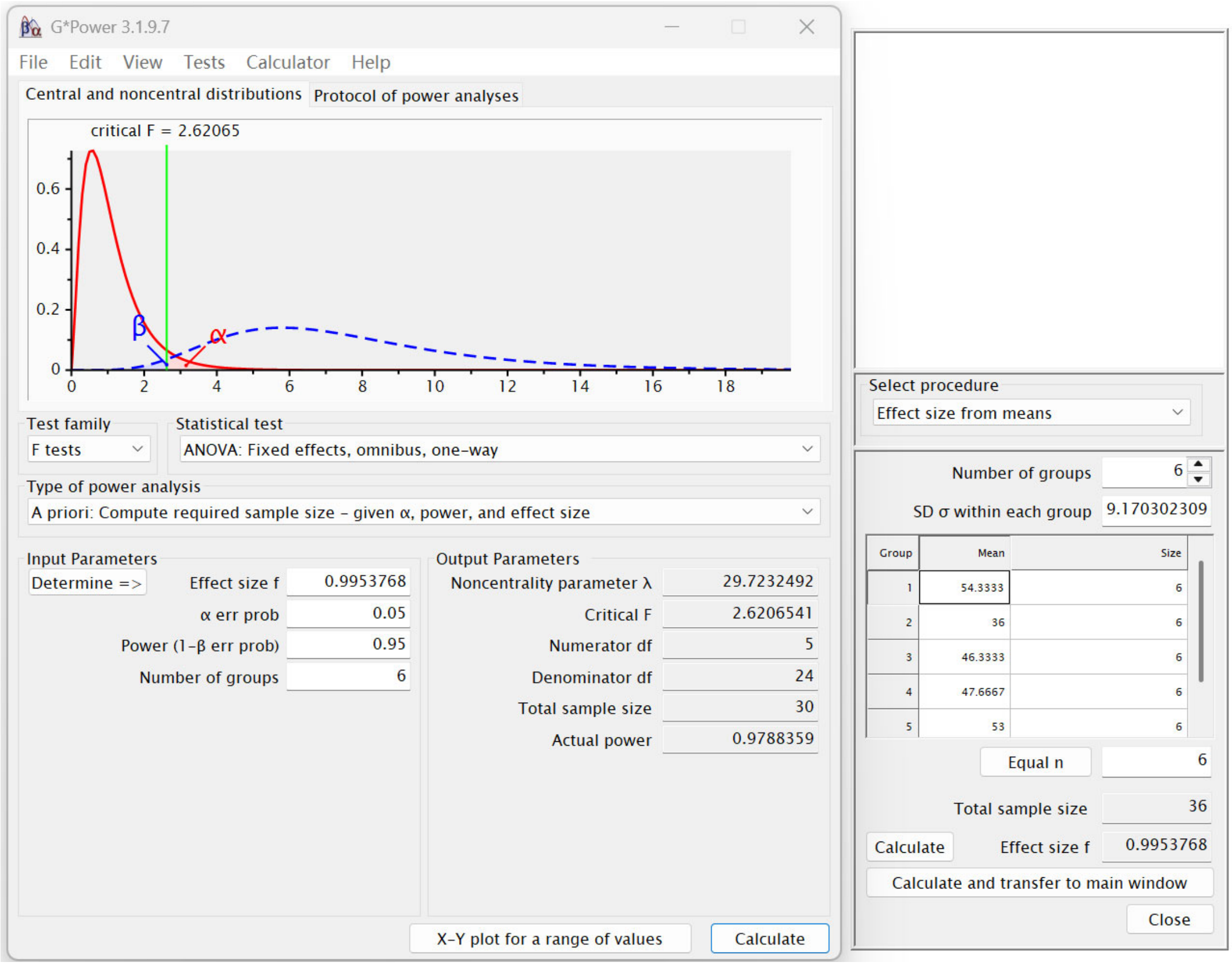

G*Power analysis (one-way ANOVA, α = 0.05, power = 0.95) under the condition of SD = 9.17 and the effect size f = 0.995 indicated that the total required sample size was 30 and n = 5 per group was sufficient. We used n=6 per group.

Fig 4.A

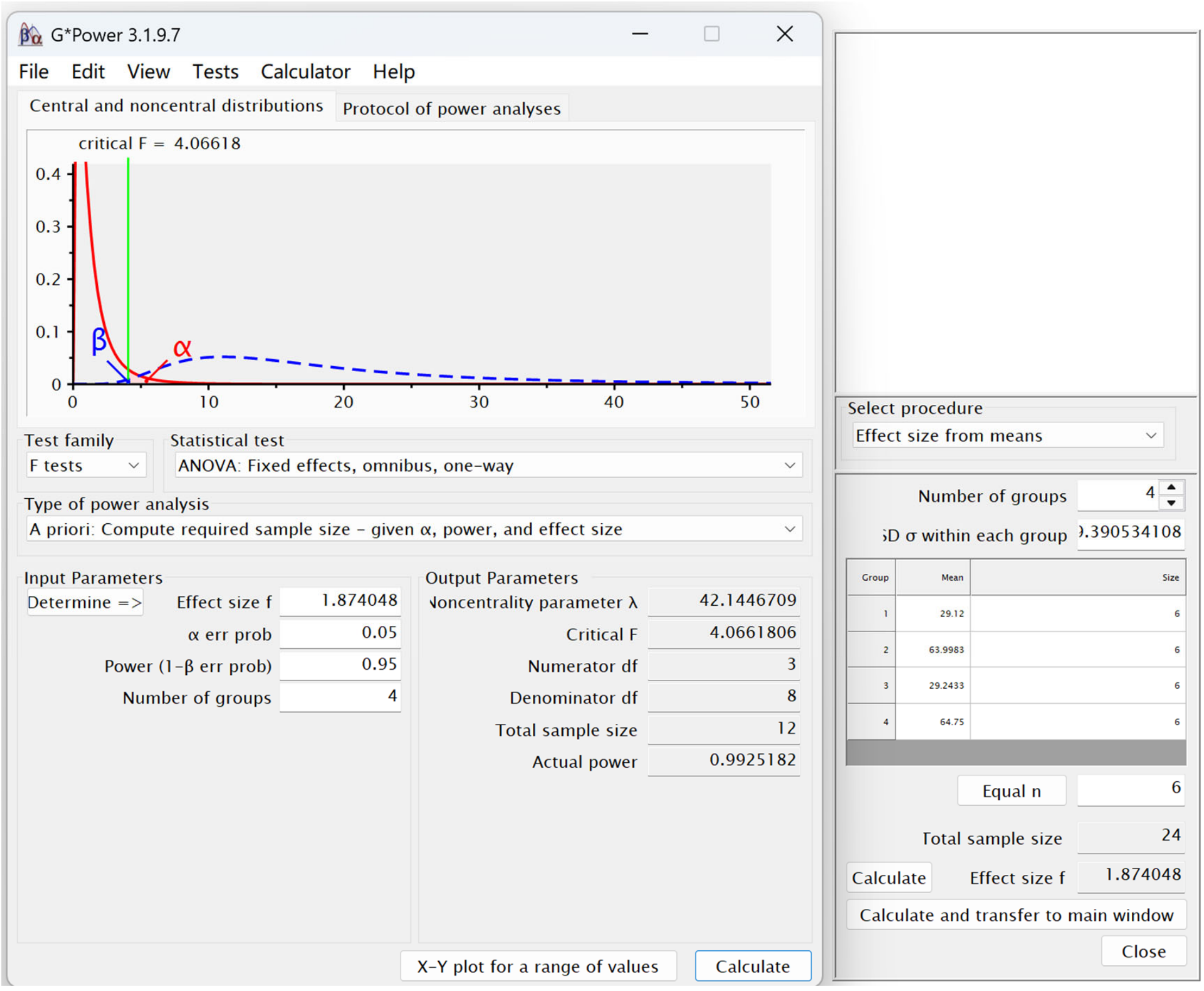

G*Power analysis (one-way ANOVA, α = 0.05, power = 0.95) under the condition of SD = 9.4 and the effect size f = 1.87 indicated that the total required sample size was 12 and n = 3 per group was sufficient. We used n=6 per group.

Fig 4.B

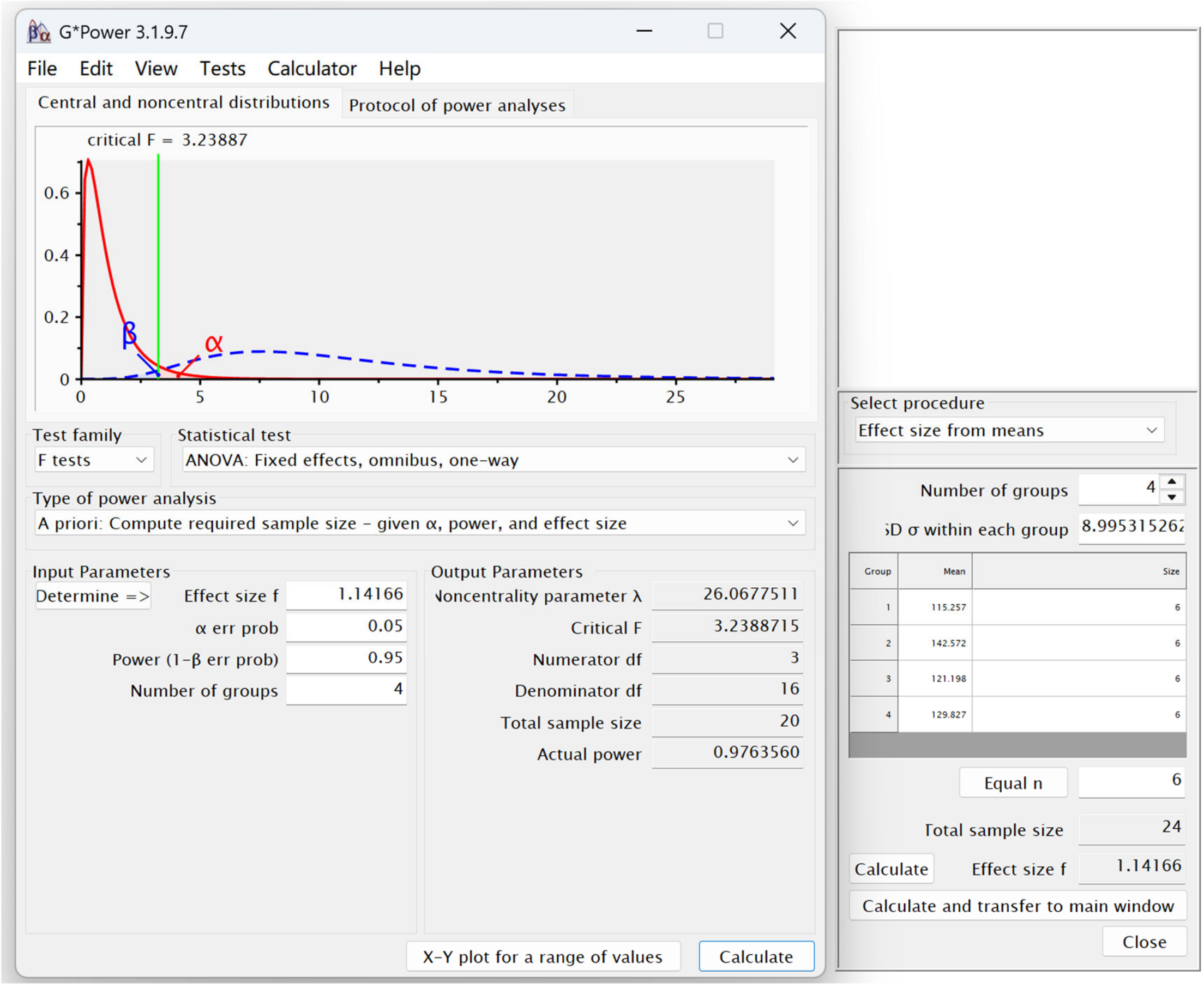

G*Power analysis (one-way ANOVA, α = 0.05, power = 0.95) under the condition of SD = 9.0 and the effect size f = 1.14 indicated that the total required sample size was 20 and n = 5 per group was sufficient. We used n=6 per group.

Fig 4.C

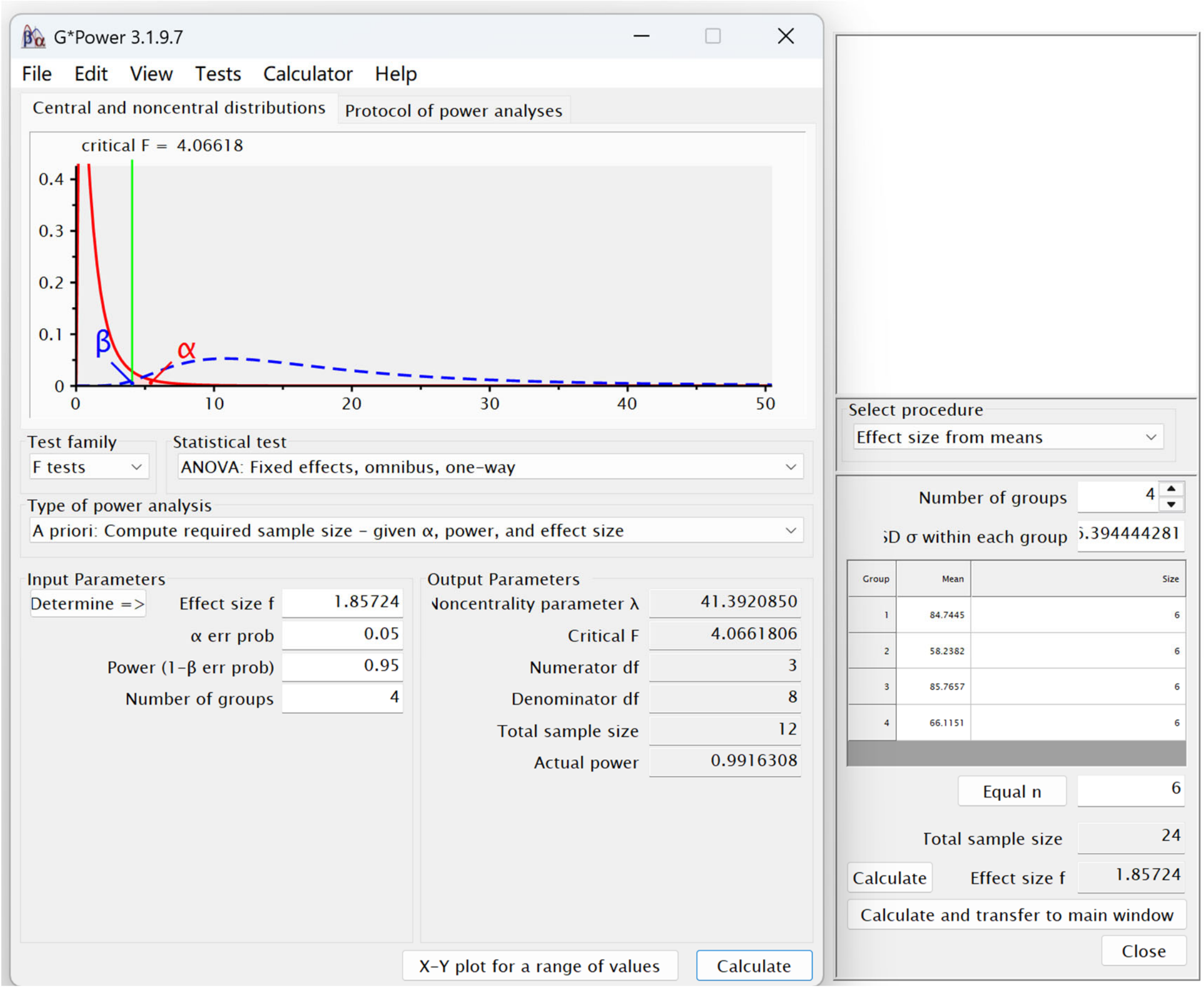

G*Power analysis (one-way ANOVA, α = 0.05, power = 0.95) under the condition of SD = 6.4 and the effect size f = 1.86 indicated that the total required sample size was 12 and n = 3 per group was sufficient. We used total n=24.

Fig 4.D

This figure was shown as representative running traces in open field test, and we do not need to do power analysis.

Fig 4.E

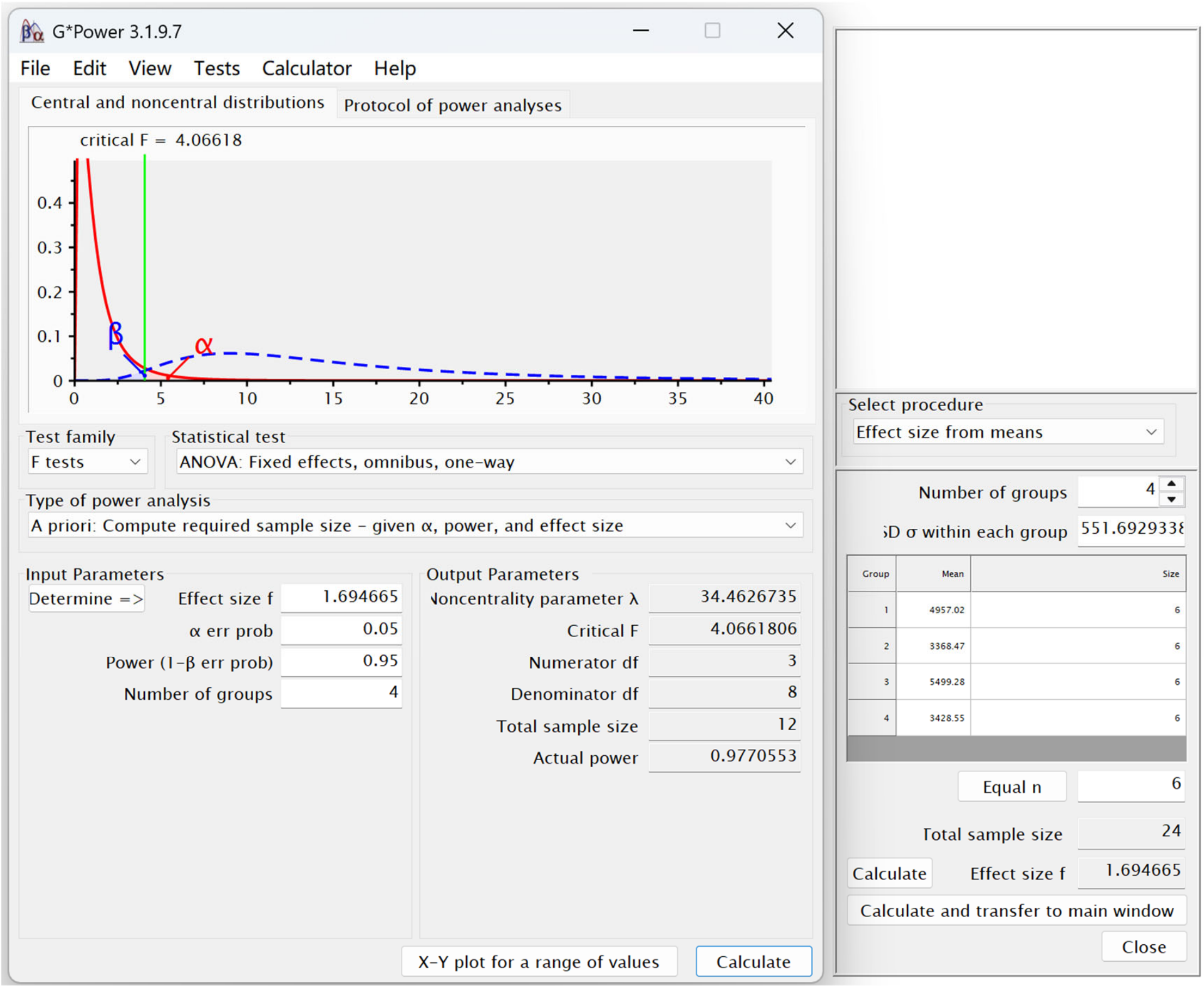

G*Power analysis (one-way ANOVA, α = 0.05, power = 0.95) under the condition of SD = 551.7 and the effect size f = 1.69 indicated that the total required sample size was 12 and n = 3 per group was sufficient. We used total n=24.

Fig 4.F

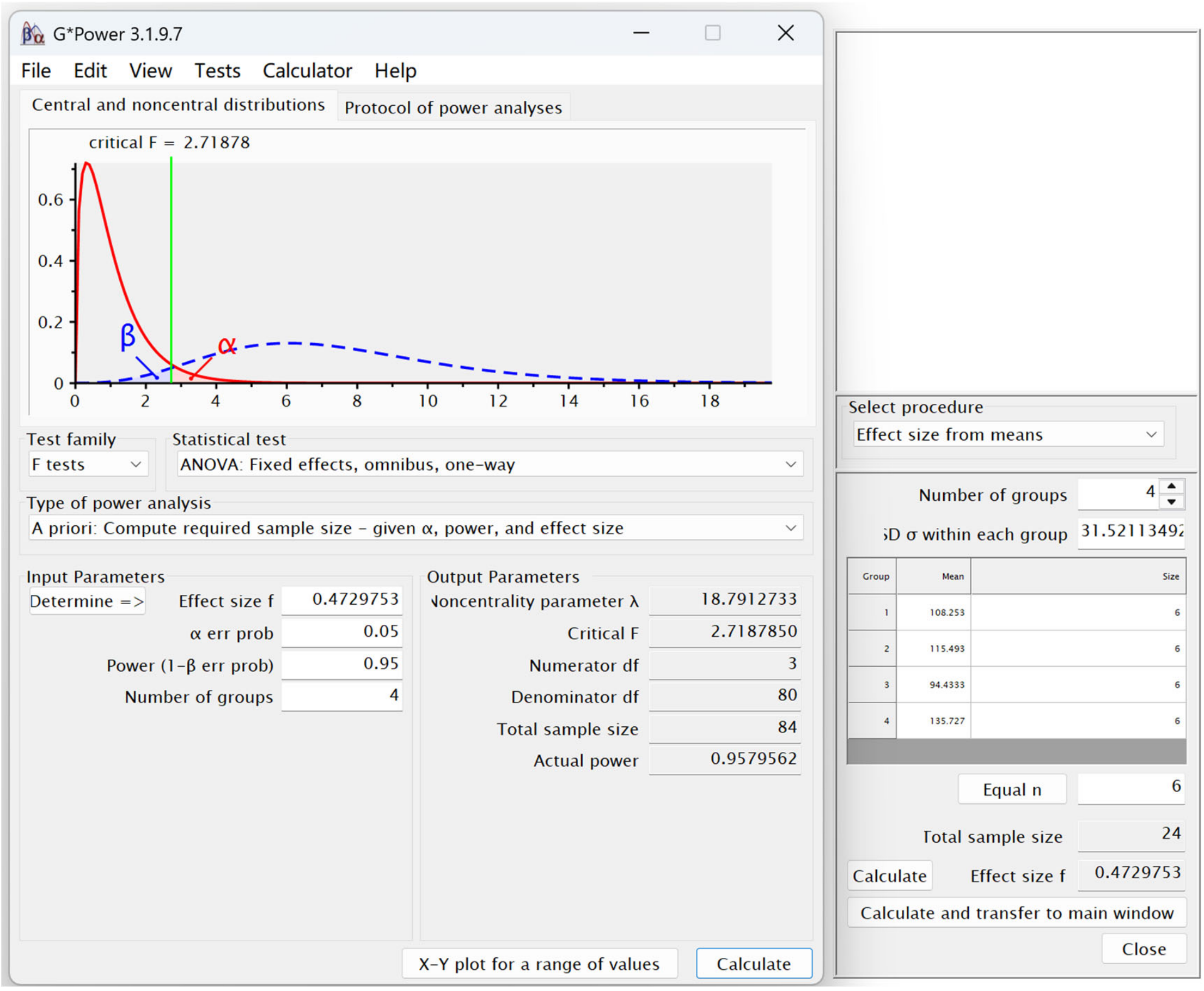

G*Power analysis (one-way ANOVA, α = 0.05, power = 0.95) under the condition of SD = 31.5 and the effect size f = 0.47 indicated that the total required sample size was 84. This large sample size requirement reflects the relatively modest biologic effect and inherent biological variability in the present model rather than a limitation in experimental design. Importantly, such a sample size is not feasible or ethically justified in animal research and is far beyond the standard practice in the field. Our sample size is consistent with previous studies using similar experimental models and endpoints[1,2]. Therefore, the absence of statistical significance in our study likely reflects a modest or biologically limited effect rather than insufficient statistical power.

(1) Song J, Piao X, Liu W, Han X, Su J, Li B. Effects of single bolus exposure to propofol on depression-like behavior in a chronic unpredictable mild stress model in mice. *Anesthesiology*. Published online March 16, 2026. doi:10.1097/ALN.0000000000006042

(2) Lin SS, Zhou B, Chen BJ, et al. Electroacupuncture prevents astrocyte atrophy to alleviate depression. Cell Death Dis. 2023;14(5):343. Published

2023 May 29. doi:10.1038/s41419-023-05839-4

Fig 4.G

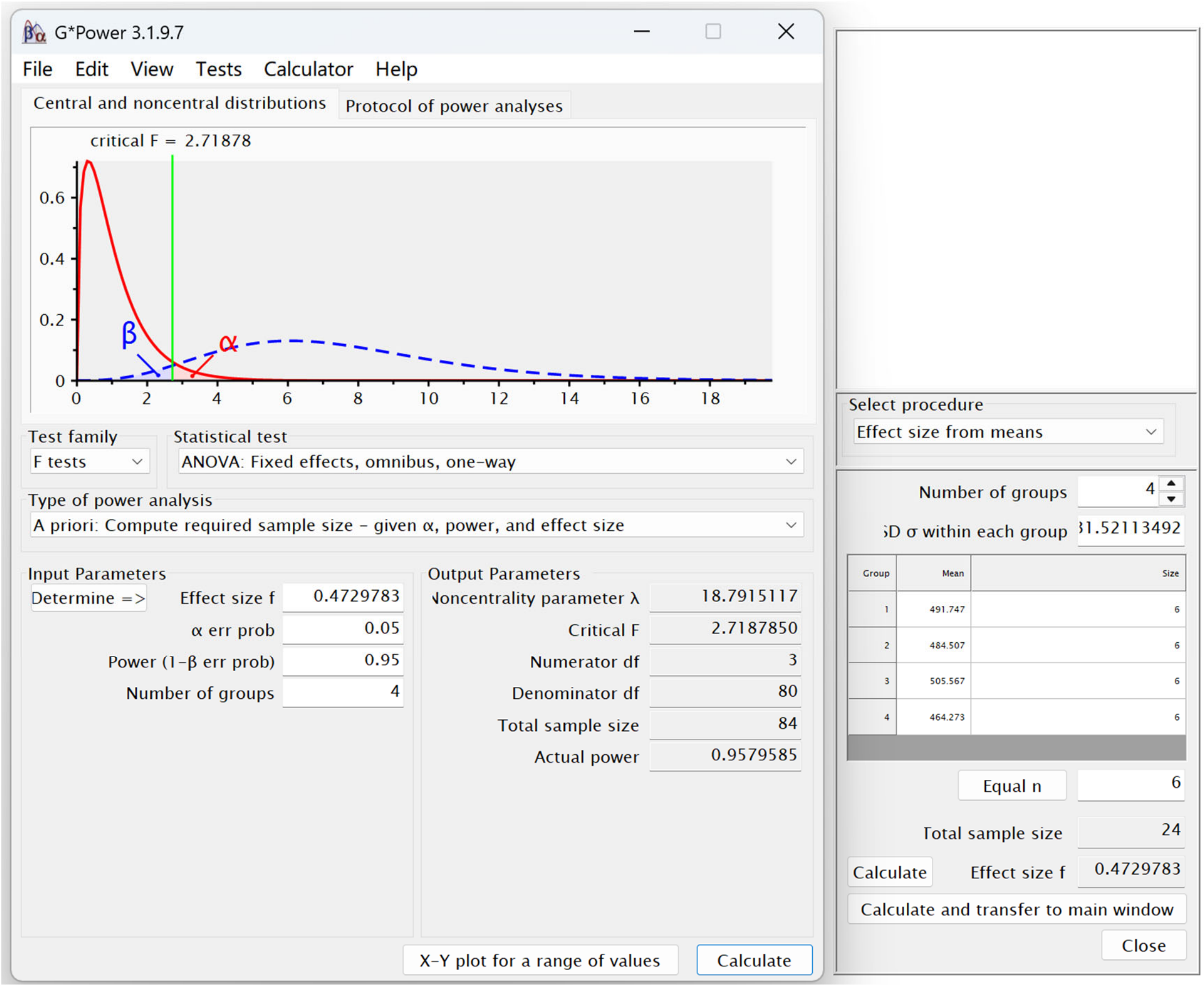

G*Power analysis (one-way ANOVA, α = 0.05, power = 0.95) under the condition of SD = 31.5 and the effect size f = 0.47 indicated that the total required sample size was 84. This large sample size requirement reflects the relatively modest biologic effect and inherent biological variability in the present model rather than a limitation in experimental design. Importantly, such a sample size is not feasible or ethically justified in animal research and is far beyond the standard practice in the field. Our sample size is consistent with previous studies using similar experimental models and endpoints[1,2]. Therefore, the absence of statistical significance in our study likely reflects a modest or biologically limited effect rather than insufficient statistical power.

(1) Song J, Piao X, Liu W, Han X, Su J, Li B. Effects of single bolus exposure to propofol on depression-like behavior in a chronic unpredictable mild stress model in mice. *Anesthesiology*. Published online March 16, 2026. doi:10.1097/ALN.0000000000006042

(2) Lin SS, Zhou B, Chen BJ, et al. Electroacupuncture prevents astrocyte atrophy to alleviate depression. Cell Death Dis. 2023;14(5):343. Published 2023 May 29. doi:10.1038/s41419-023-05839-4

Fig 4.H

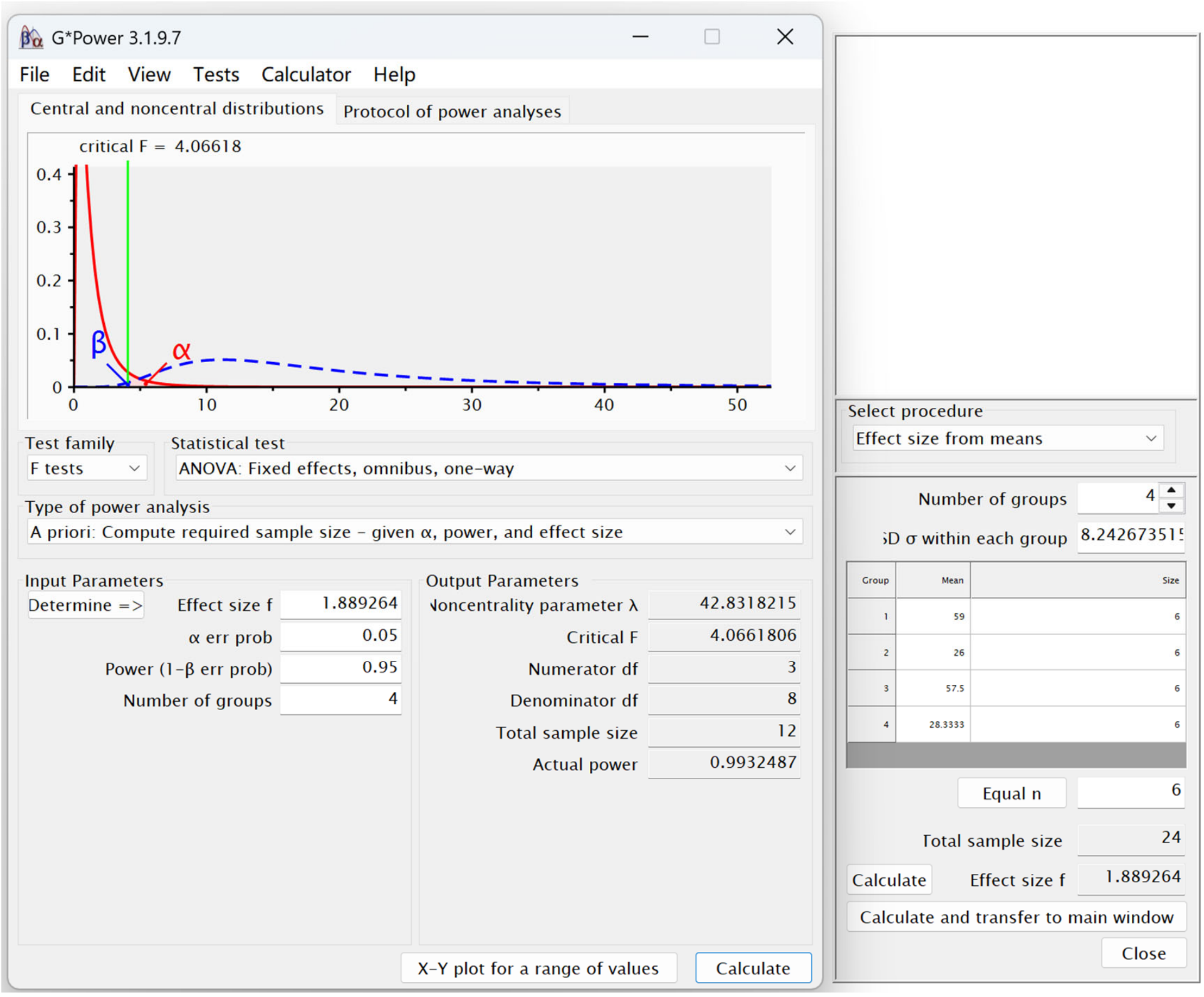

G*Power analysis (one-way ANOVA, α = 0.05, power = 0.95) under the condition of SD = 8.2 and the effect size f = 1.89 indicated that the total required sample size was 12. We used total n=24.

Fig 5.A

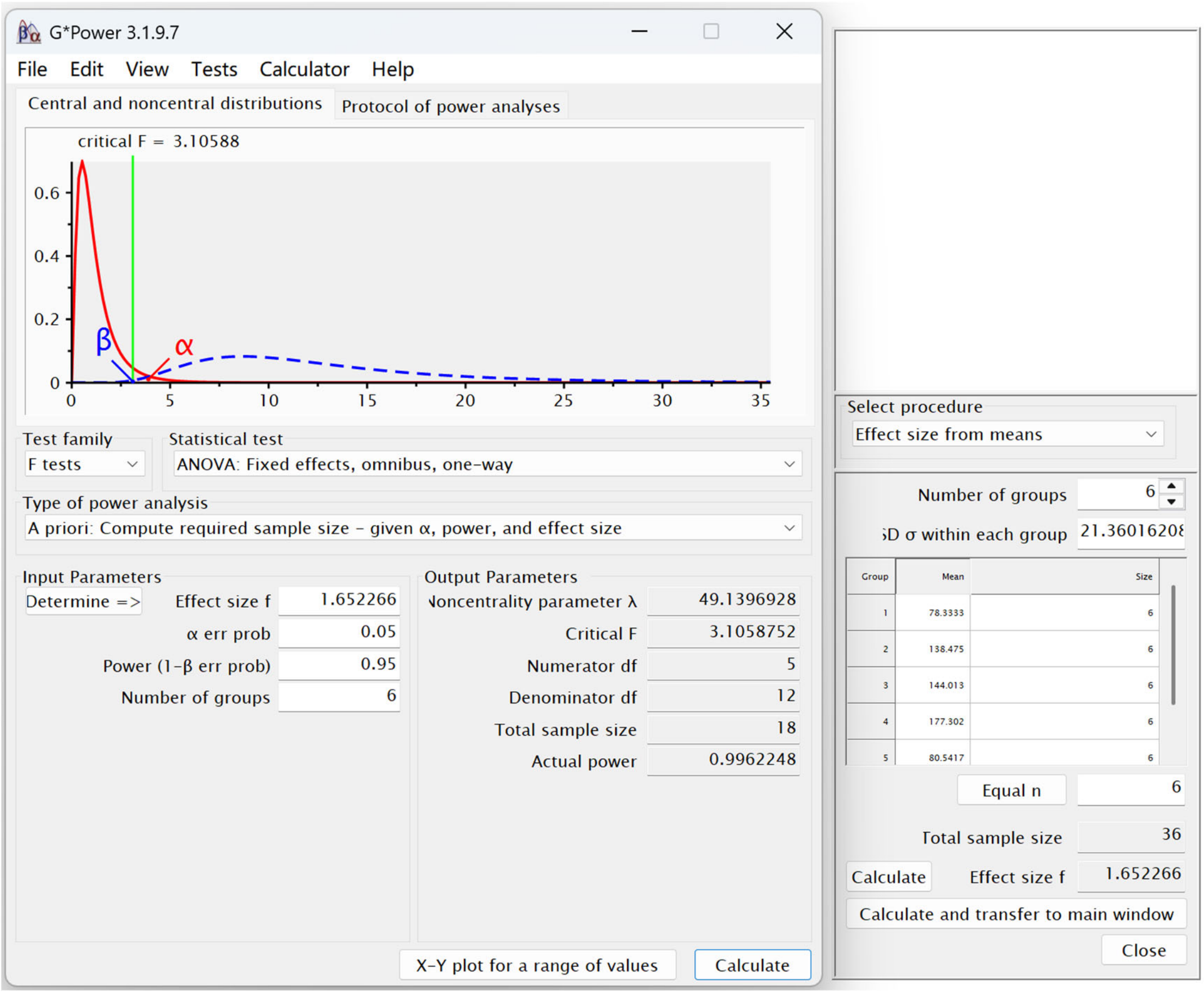

G*Power analysis (one-way ANOVA, α = 0.05, power = 0.95) under the condition of SD = 21.4 and the effect size f = 1.65 indicated that the total required sample size was 18. We used total n=36.

Fig 5.B

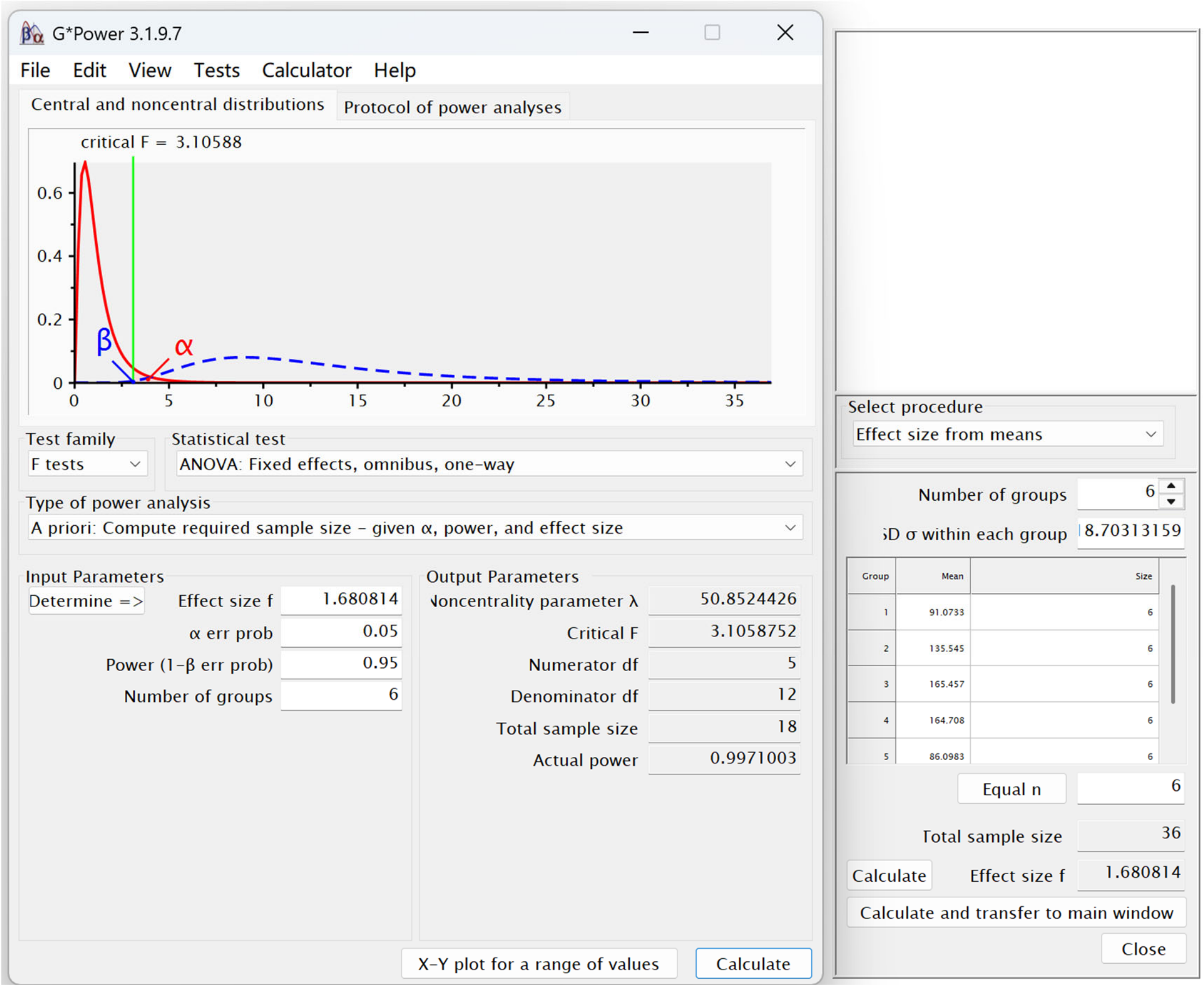

G*Power analysis (one-way ANOVA, α = 0.05, power = 0.95) under the condition of SD = 18.7 and the effect size f = 1.68 indicated that the total required sample size was 18. We used total n=36.

Fig 5.C

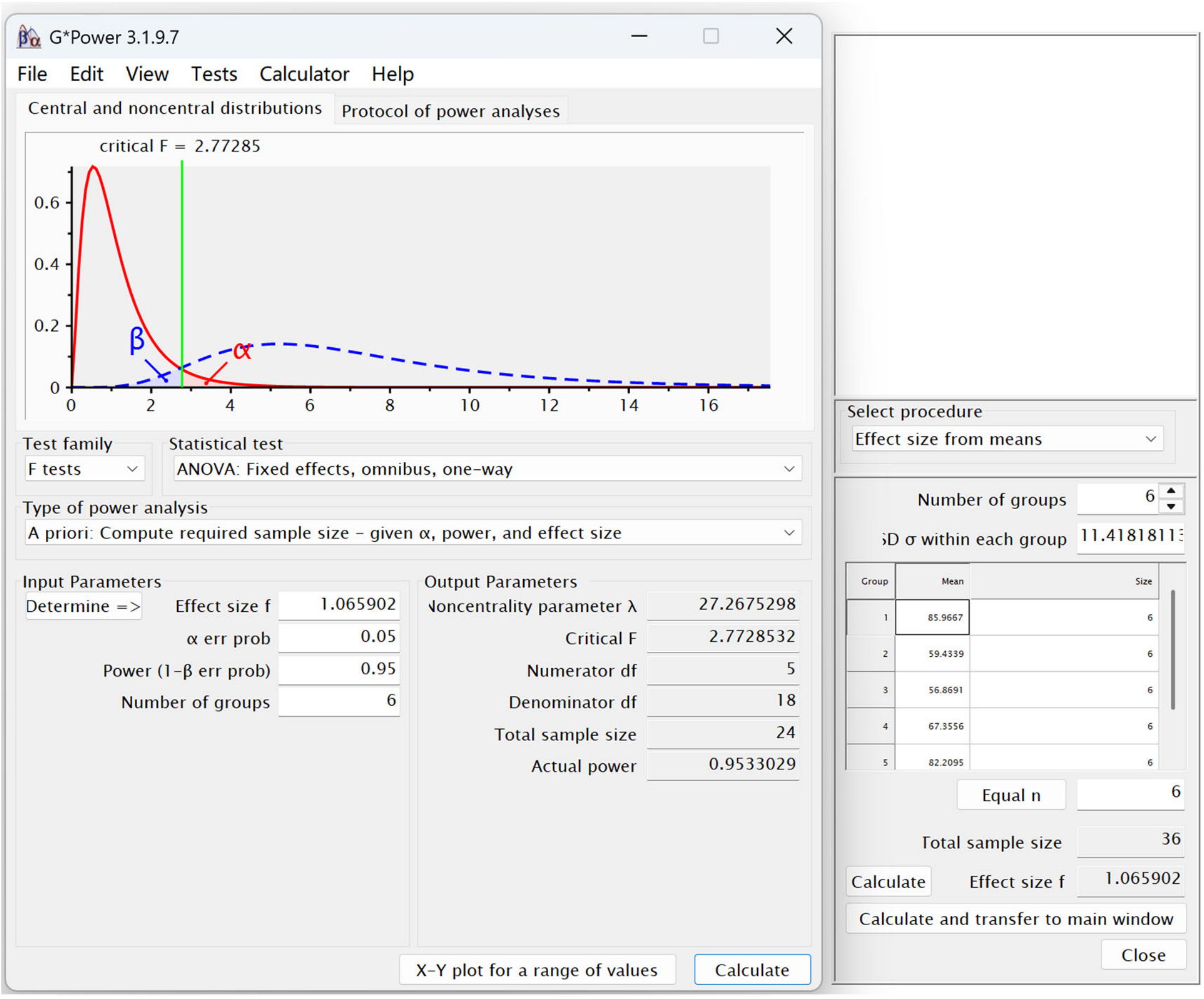

G*Power analysis (one-way ANOVA, α = 0.05, power = 0.95) under the condition of SD = 11.4 and the effect size f = 1.07 indicated that the total required sample size was 24. We used total n=36.

Fig 5.D

This figure was shown as representative running traces in open field test, and we do not need to do power analysis.

Fig 5.E

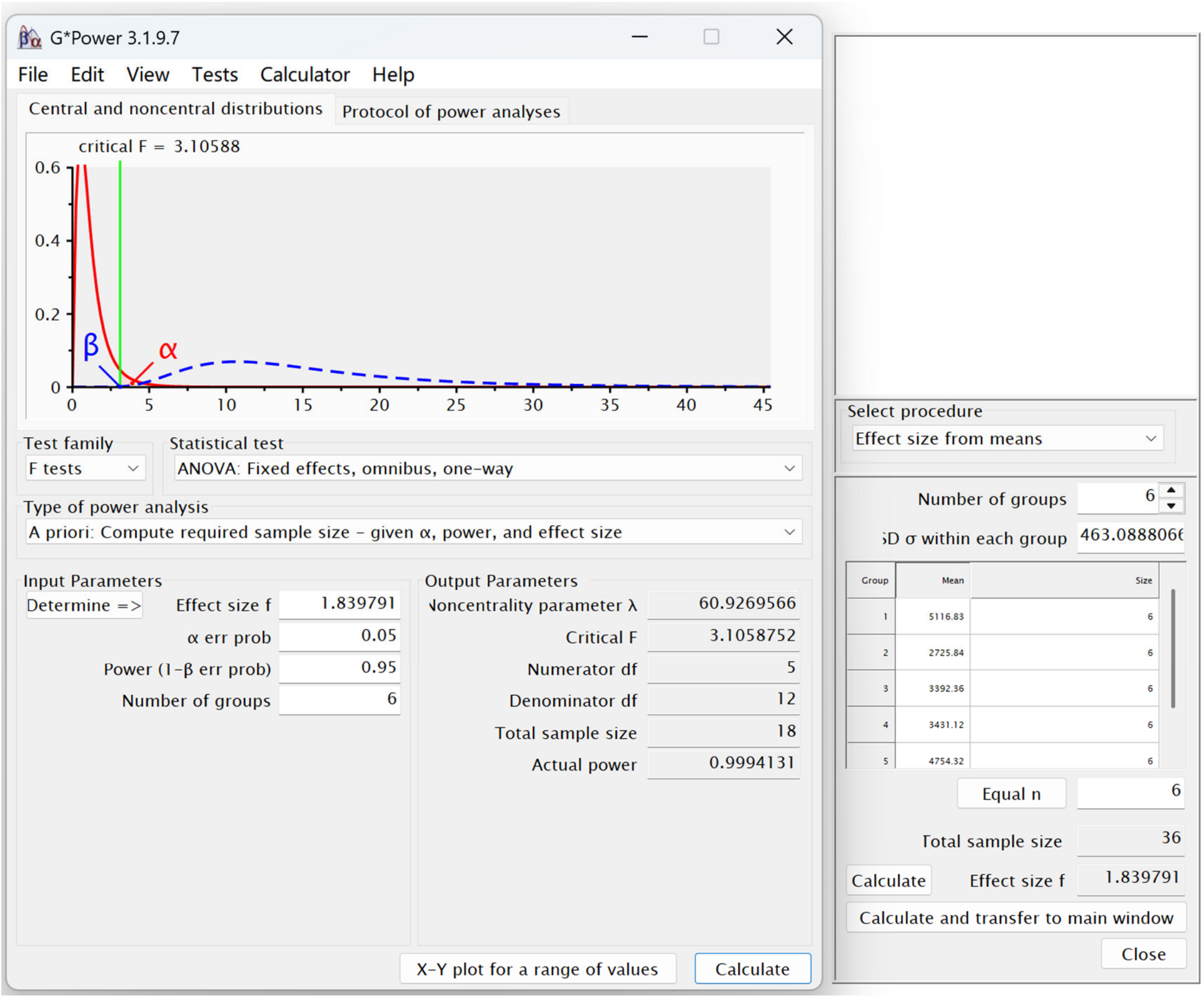

G*Power analysis (one-way ANOVA, α = 0.05, power = 0.95) under the condition of SD = 463.1 and the effect size f = 1.84 indicated that the total required sample size was 18. We used total n=36.

Fig 5.F

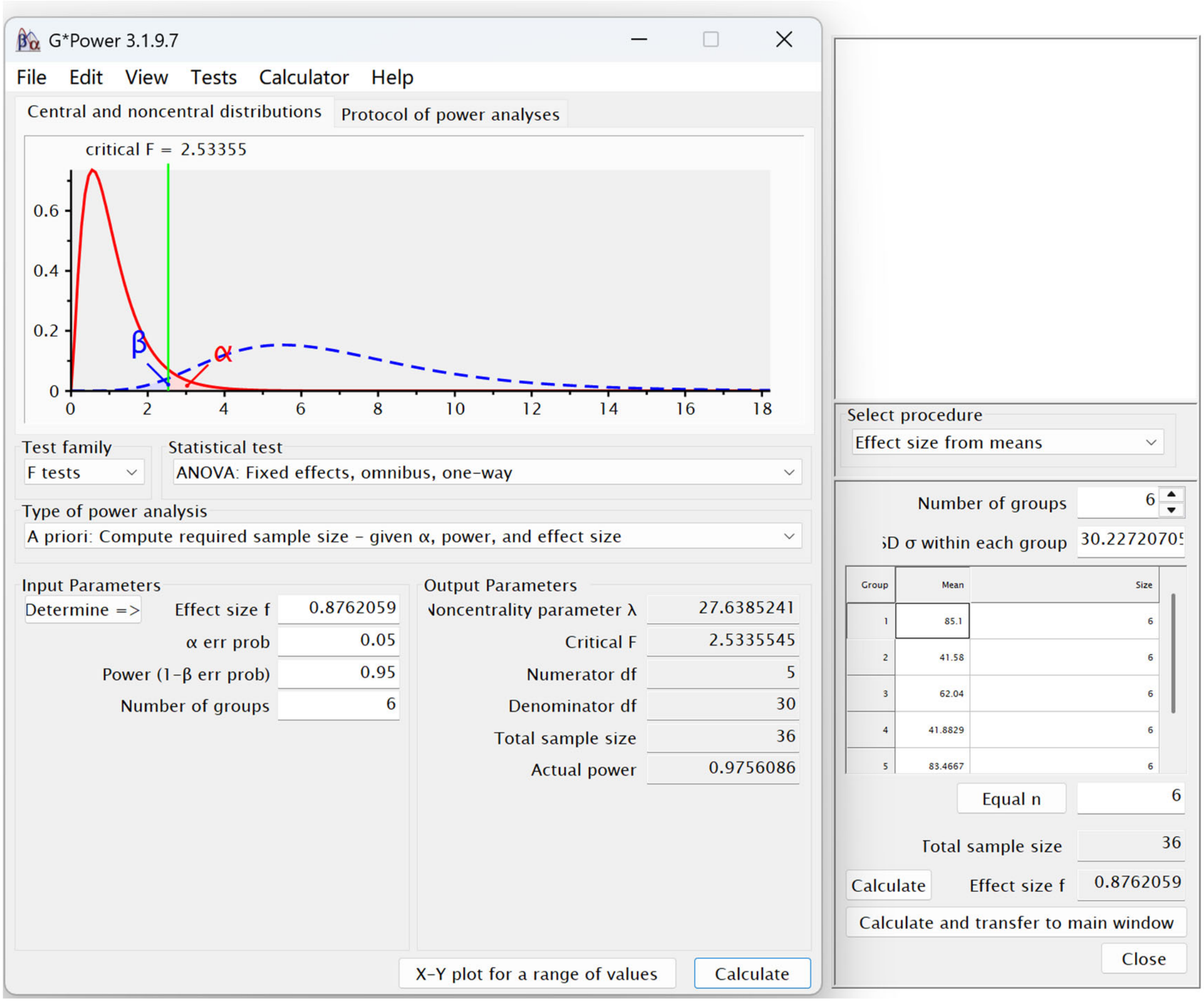

G*Power analysis (one-way ANOVA, α = 0.05, power = 0.95) under the condition of SD = 30.2 and the effect size f = 0.88 indicated that the total required sample size was 36. We used total n=36.

Fig 5.G

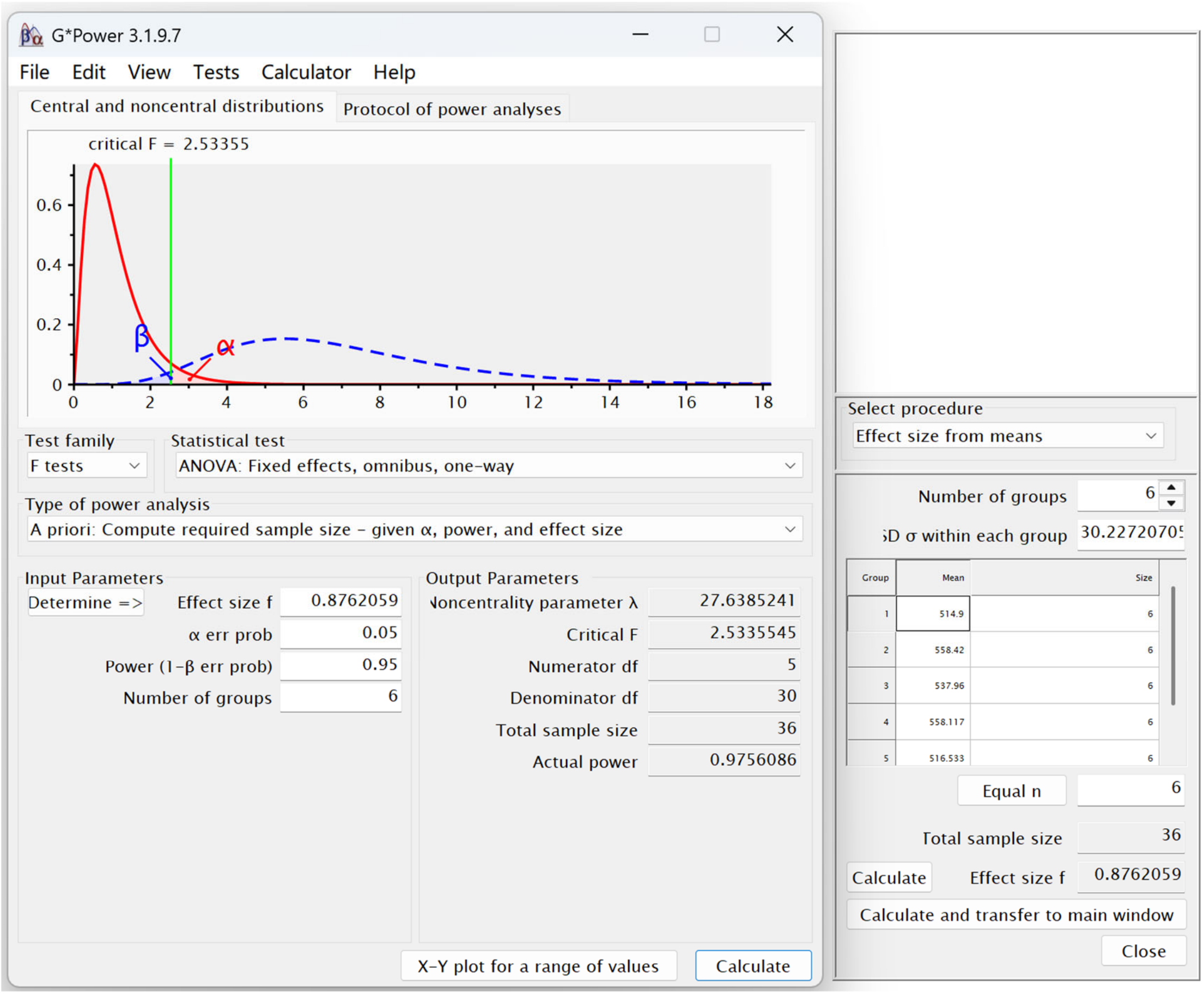

G*Power analysis (one-way ANOVA, α = 0.05, power = 0.95) under the condition of SD = 30.2 and the effect size f = 0.88 indicated that the total required sample size was 36. We used total n=36.

Fig 5.H

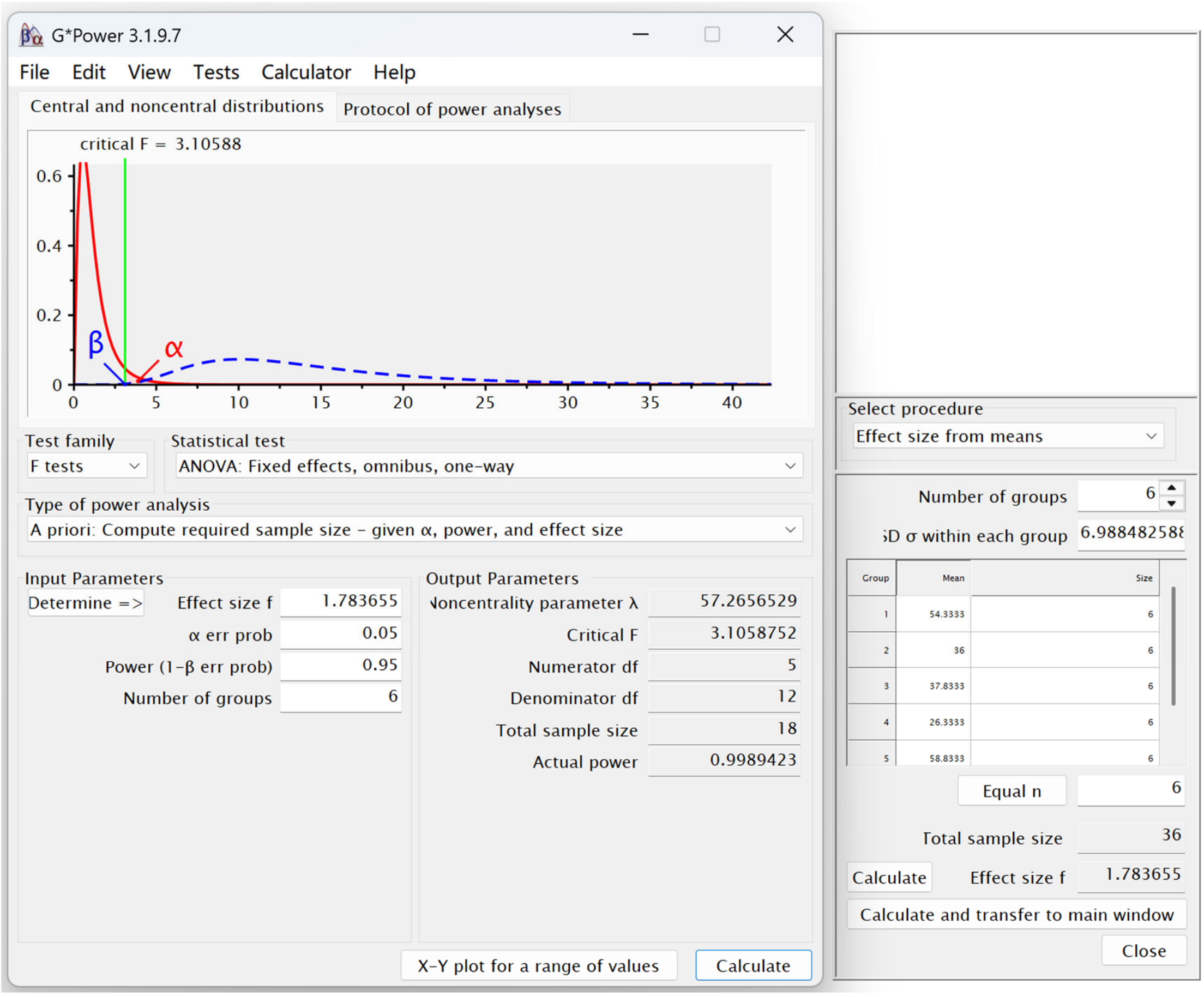

G*Power analysis (one-way ANOVA, α = 0.05, power = 0.95) under the condition of SD = 7.0 and the effect size f = 1.78 indicated that the total required sample size was 18. We used total n=36.

Fig 6.A

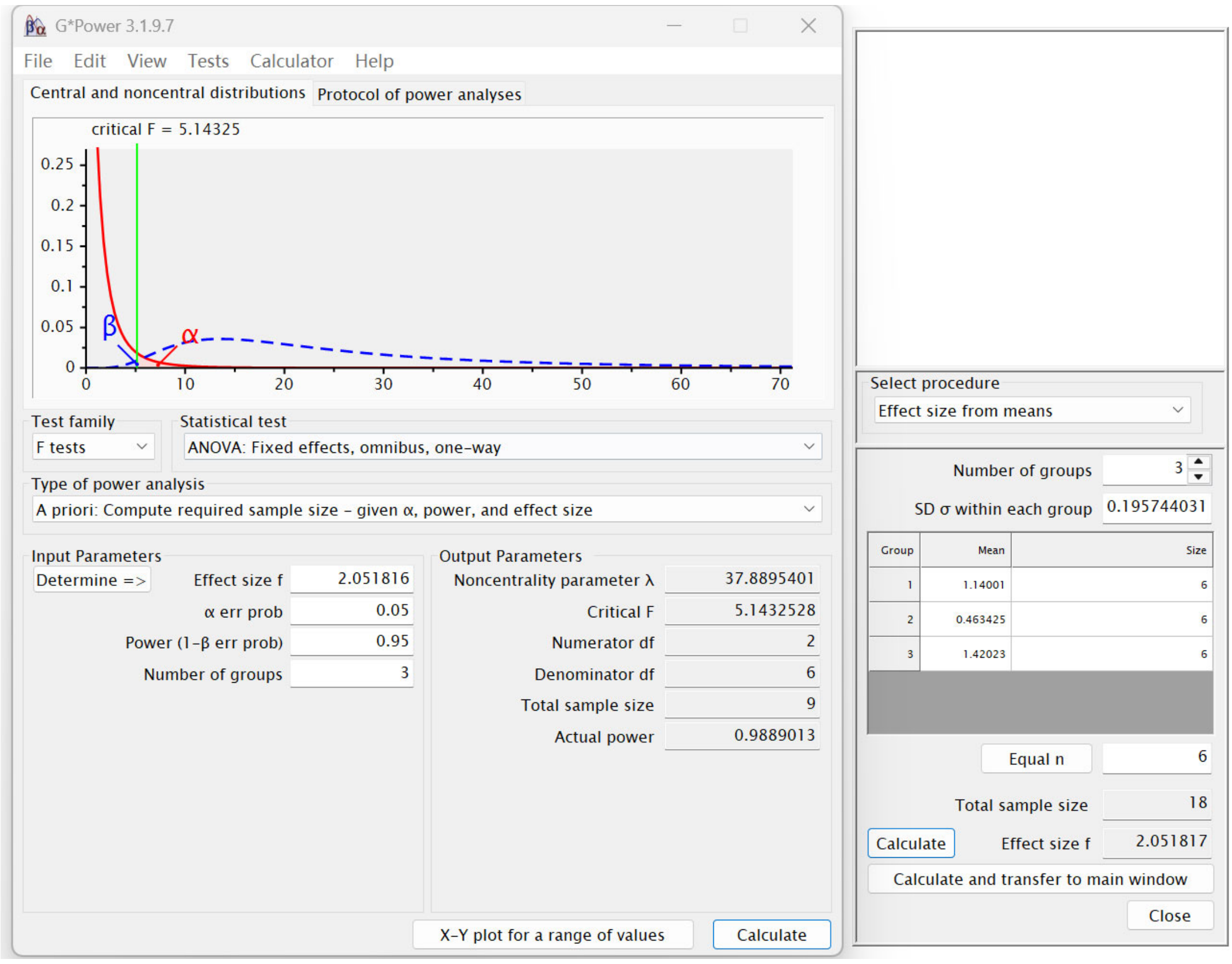

G*Power analysis (one-way ANOVA, α = 0.05, power = 0.95) under the condition of SD = 0.2 and the effect size f = 2.052 indicated that the total required sample size was 9 and n = 3 per group was sufficient. We used n=6 per group.

Fig 6.B

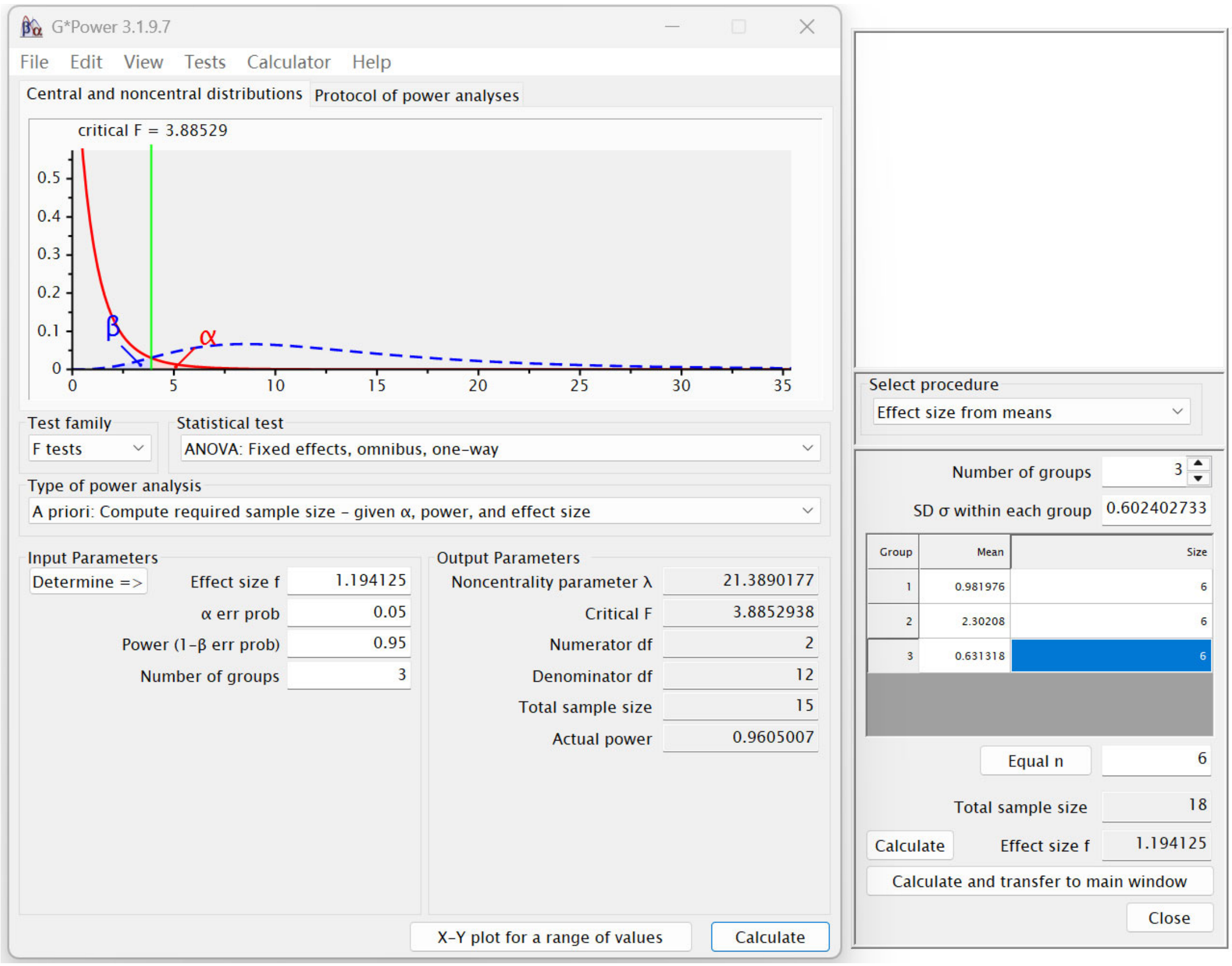

G*Power analysis (one-way ANOVA, α = 0.05, power = 0.95) under the condition of SD = 0.6024 and the effect size f = 1.194 indicated that the total required sample size was 15 and n = 5 per group was sufficient. We used n=6 per group.

Fig 6.C

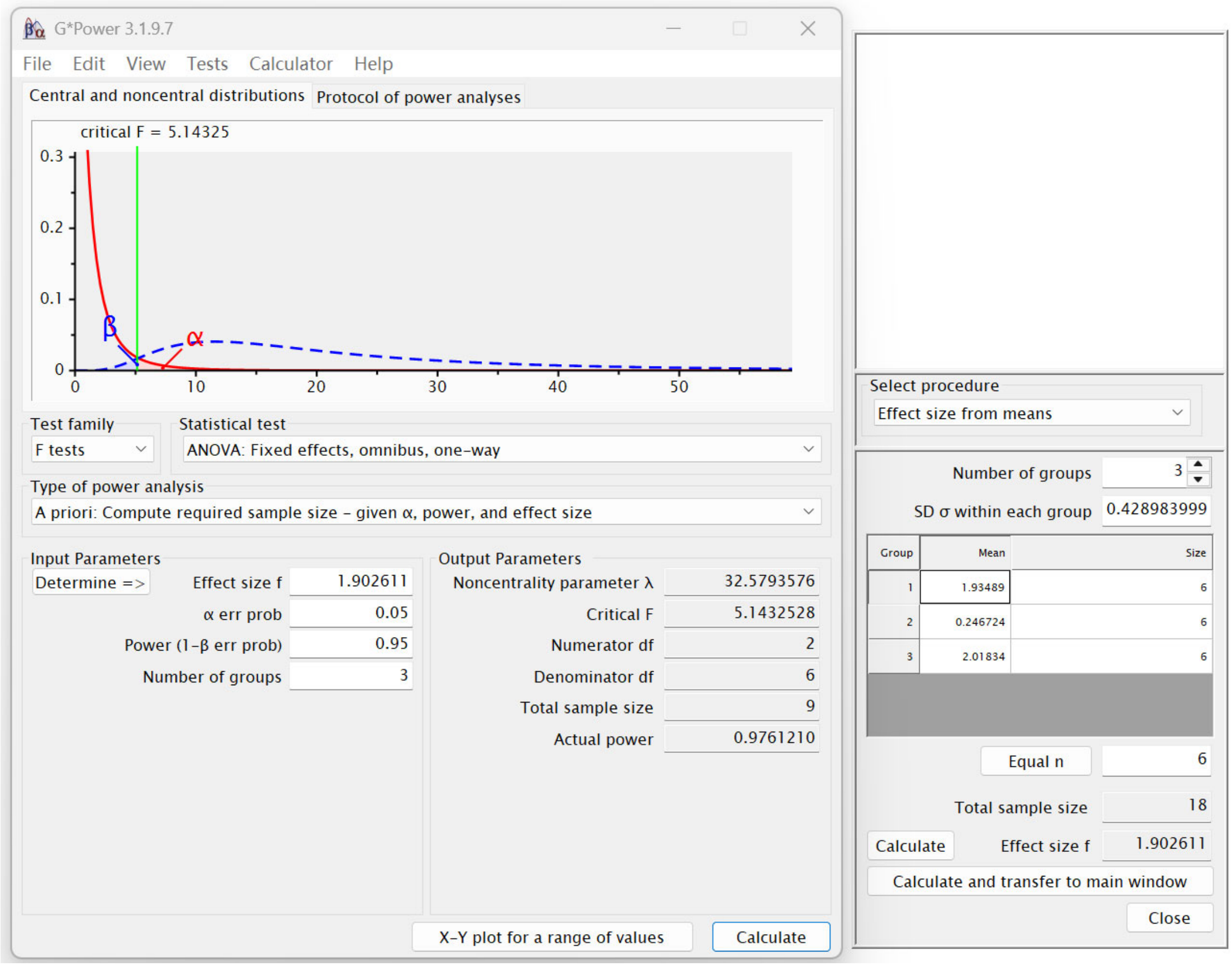

G*Power analysis (one-way ANOVA, α = 0.05, power = 0.95) under the condition of SD = 0.429 and the effect size f = 1.902 indicated that the total required sample size was 9 and n = 3 per group was sufficient. We used n=6 per group.

Fig 6.D

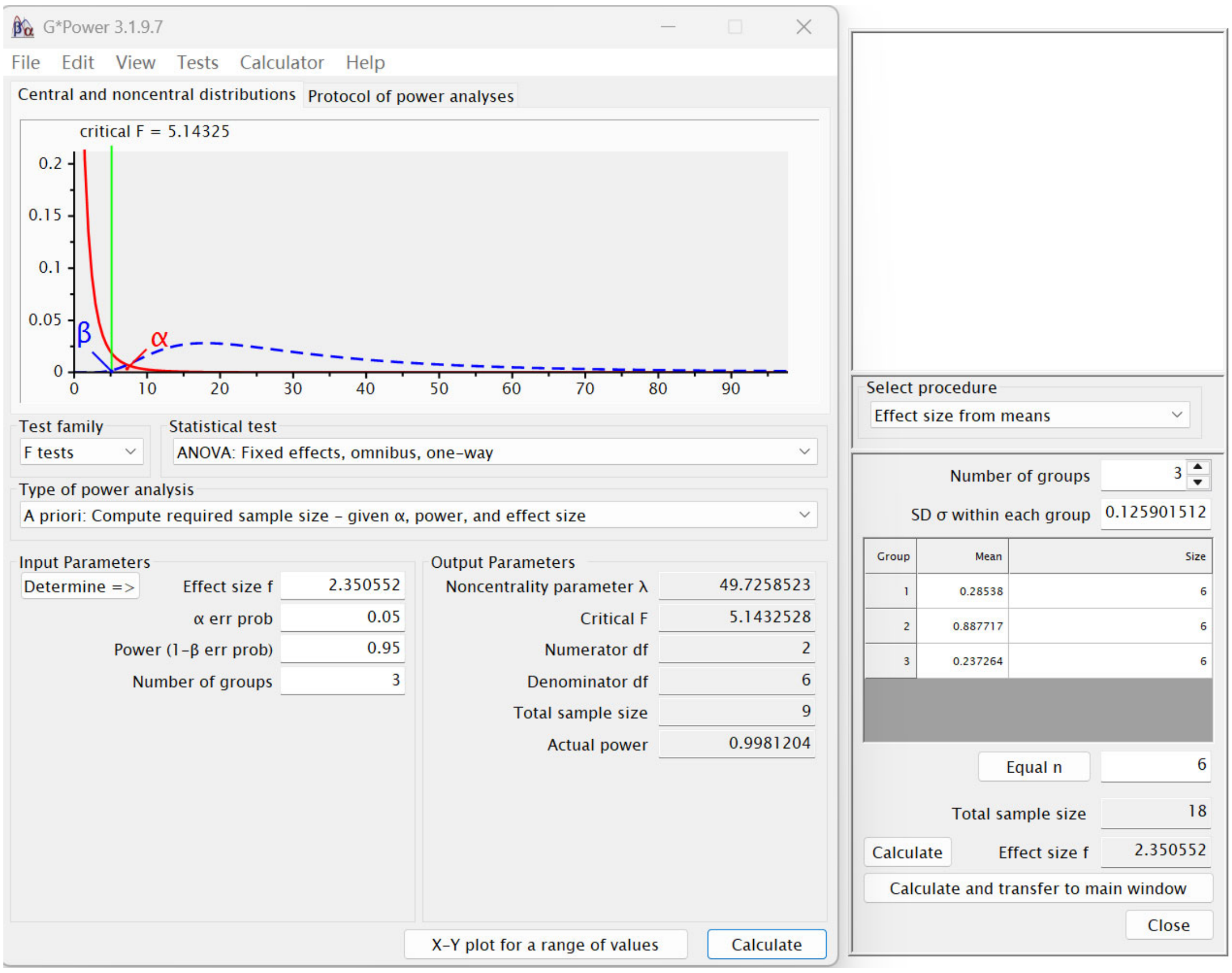

G*Power analysis (one-way ANOVA, α = 0.05, power = 0.95) under the condition of SD = 0.126 and the effect size f = 2.35 indicated that the total required sample size was 9 and n = 3 per group was sufficient. We used n=6 per group.

Fig 6.E

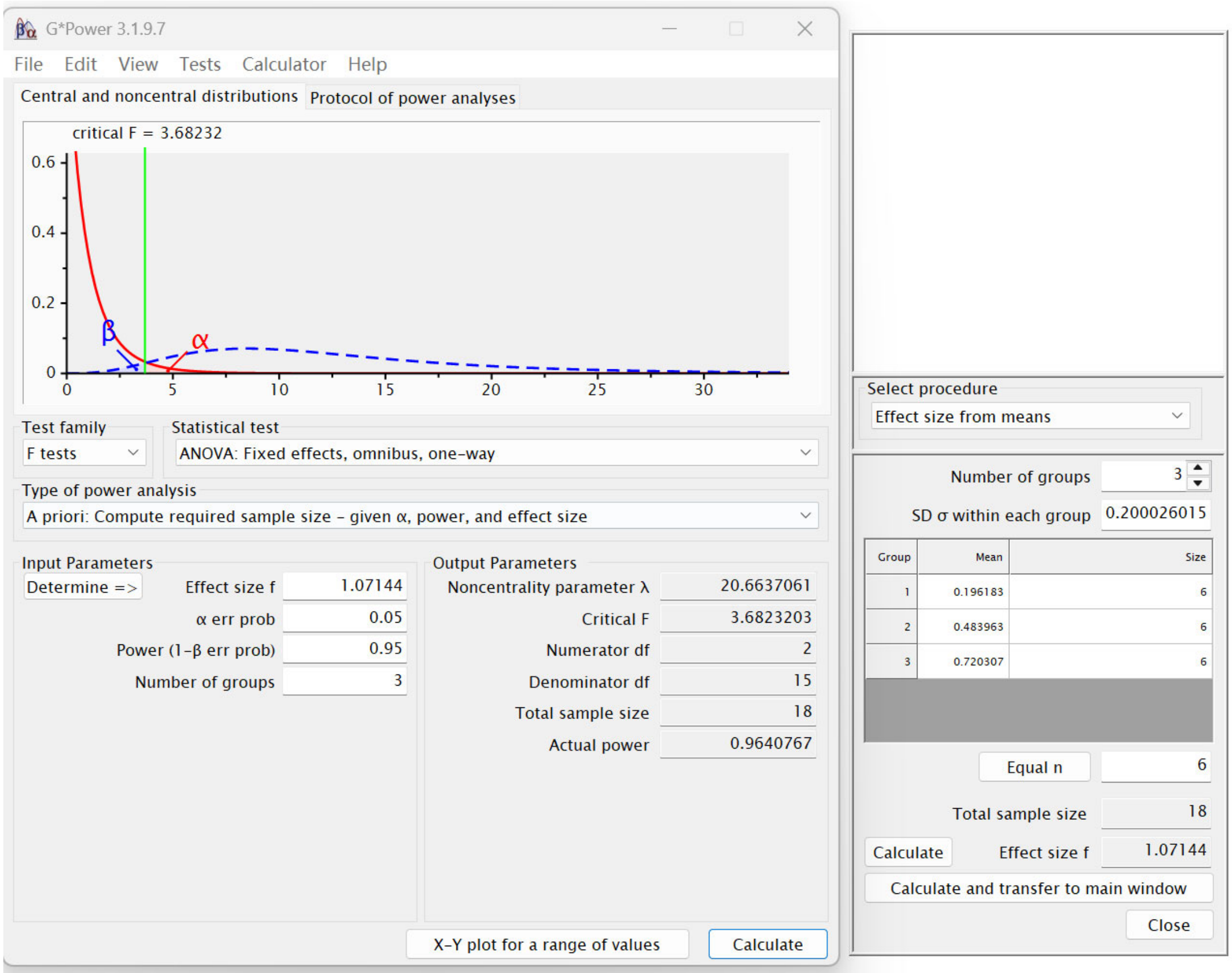

G*Power analysis (one-way ANOVA, α = 0.05, power = 0.95) under the condition of SD = 0.2 and the effect size f = 1.071 indicated that the total required sample size was 18 and n = 6 per group was sufficient. We used n=6 per group.

Fig 6.F

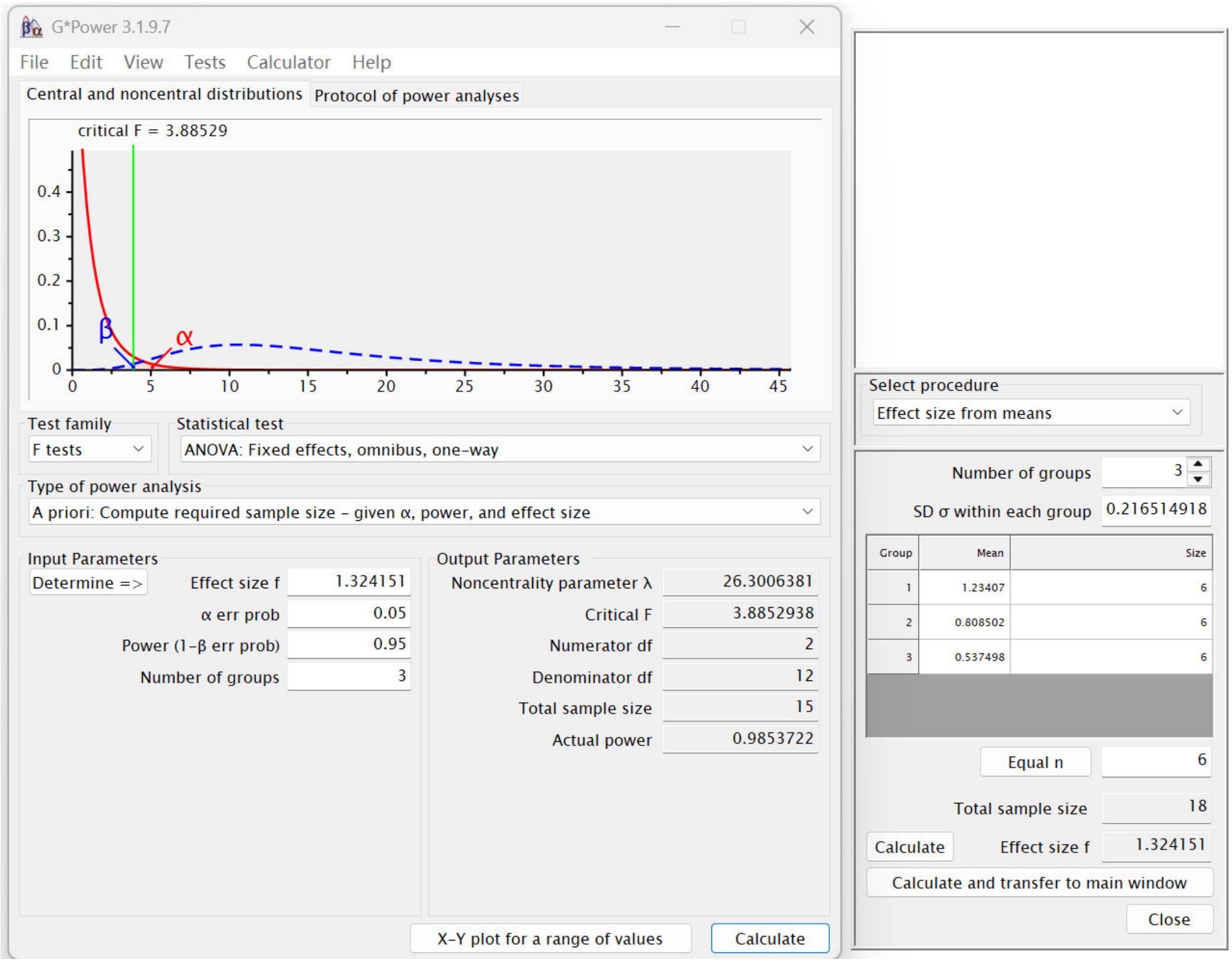

G*Power analysis (one-way ANOVA, α = 0.05, power = 0.95) under the condition of SD = 0.2165 and the effect size f = 1.324 indicated that the total required sample size was 15 and n = 5 per group was sufficient. We used n=6 per group.

Fig 6.G

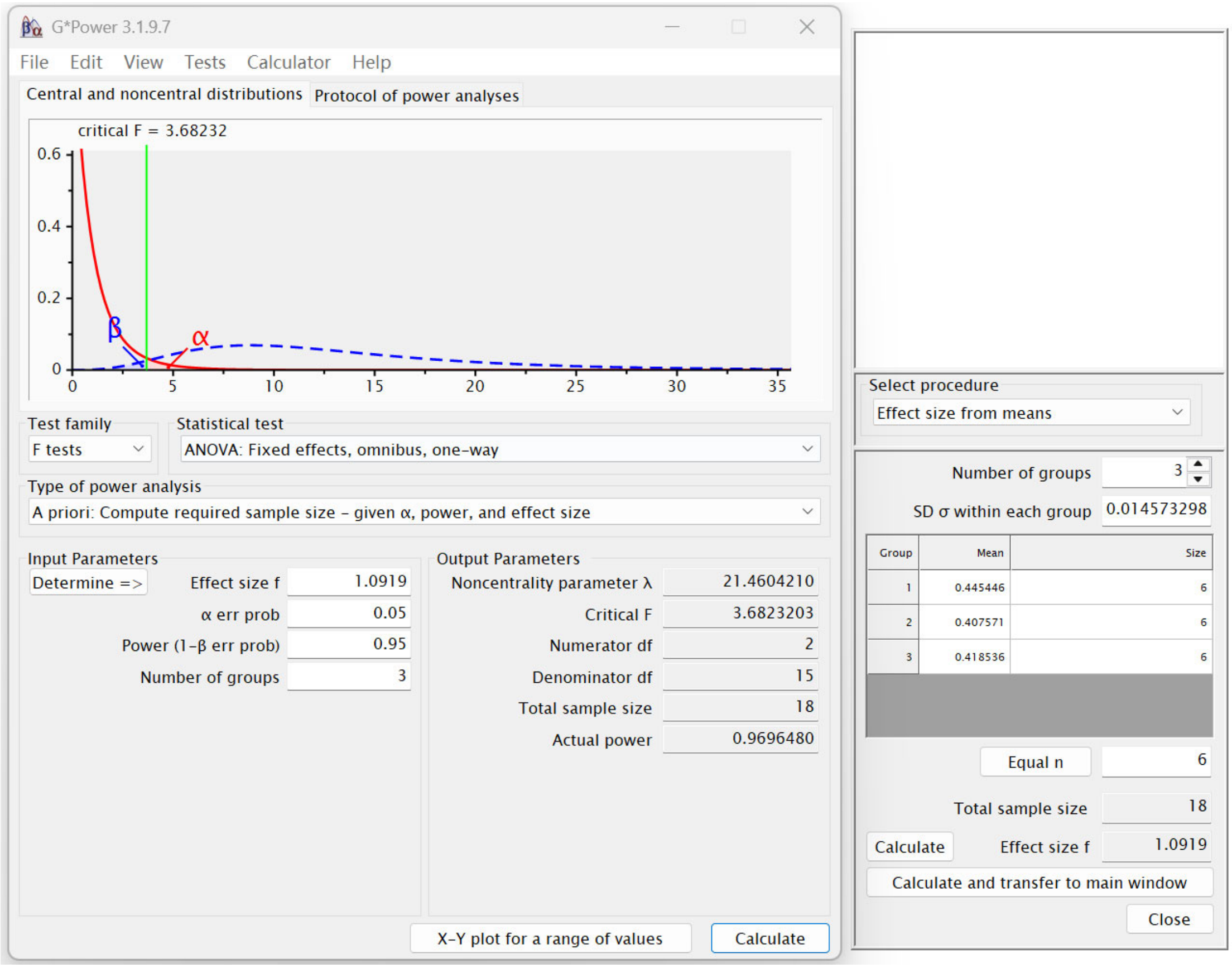

G*Power analysis (one-way ANOVA, α = 0.05, power = 0.95) under the condition of SD = 0.0146 and the effect size f = 1.091 indicated that the total required sample size was 18 and n = 6 per group was sufficient. We used n=6 per group.

Fig 6.H

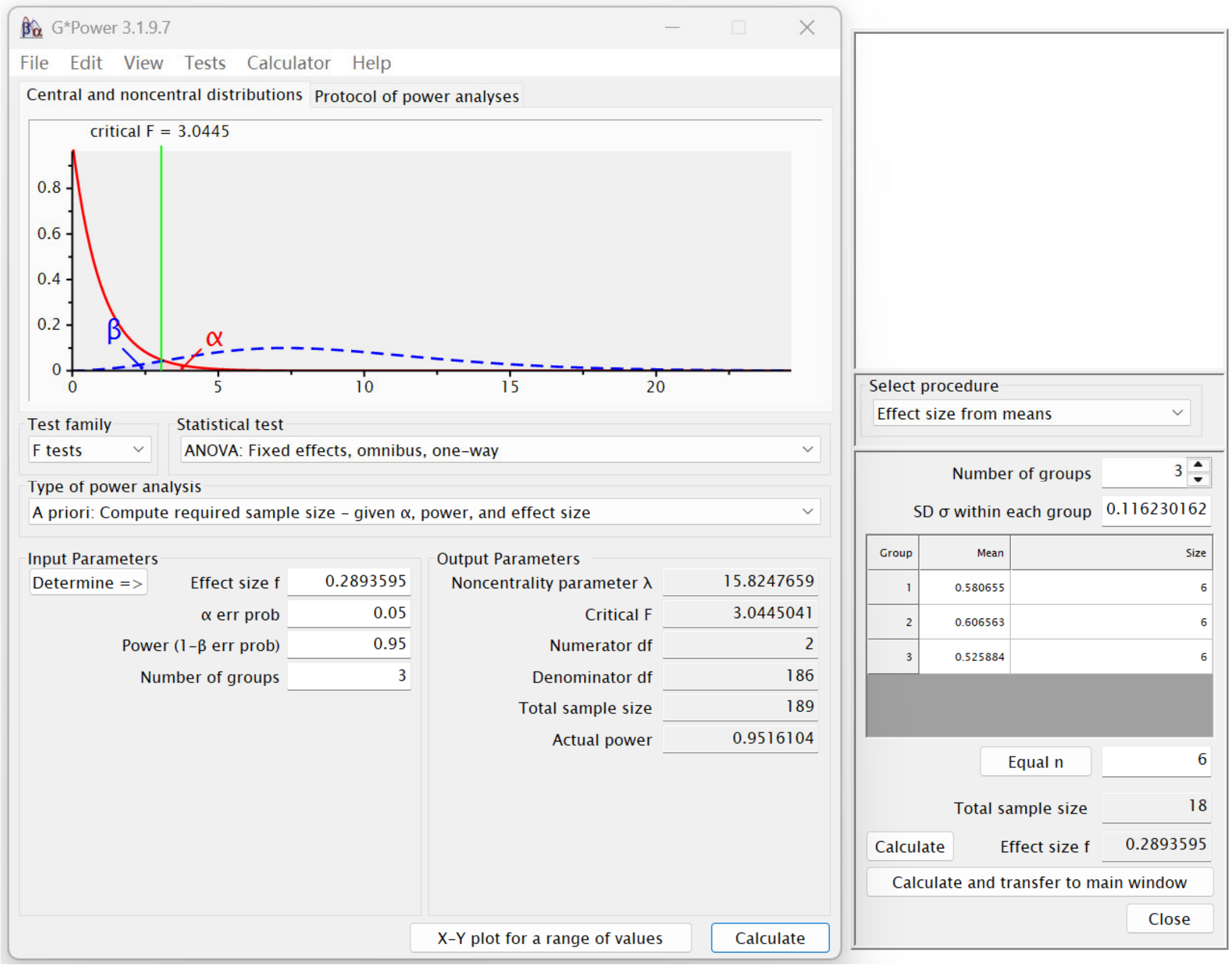

We performed an a priori power analysis using G*Power (α = 0.05, power=0.95) based on the observed variability and group differences. The analysis indicated that achieving a statistical power of 0.95 would require a total sample size of approximately 189 animals for GABA calcium detection. This large sample size requirement reflects the relatively modest biologic effect and inherent biological variability in the present model rather than a limitation in experimental design. Importantly, such a sample size is not feasible or ethically justified in animal research and is far beyond the standard practice in the field. Our sample size is consistent with previous studies using similar endpoints[1]. Therefore, the absence of statistical significance in our study likely reflects a modest or biologically limited effect rather than insufficient statistical power.

(1) Bian J, Wang XL, Yin L, et al. PVNCRF Neurons Regulate Migraine-Like Allodynia by Activating CRFR2 on Spinal Trigeminal Caudalis Glutamatergic Neurons. Adv Sci (Weinh). Published online February 28, 2026. doi:10.1002/advs.202520530

Fig 6.I

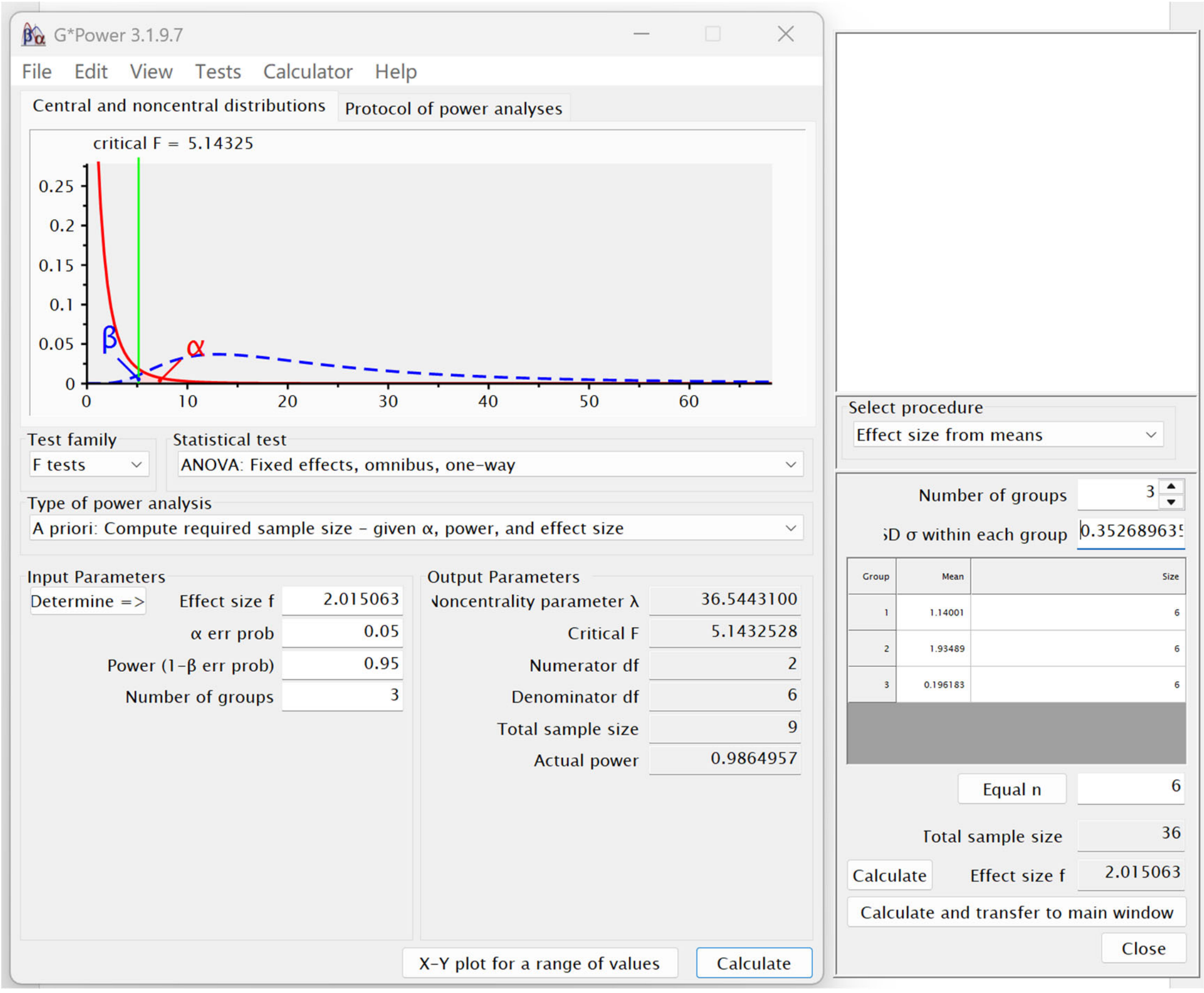

We performed an a priori power analysis using G*Power (α = 0.05, power=0.95) based on the observed variability and group differences. The analysis indicated that achieving a statistical power of 0.95 would require a total sample size of 9 animals. We used 18 mice.

Fig 6.J

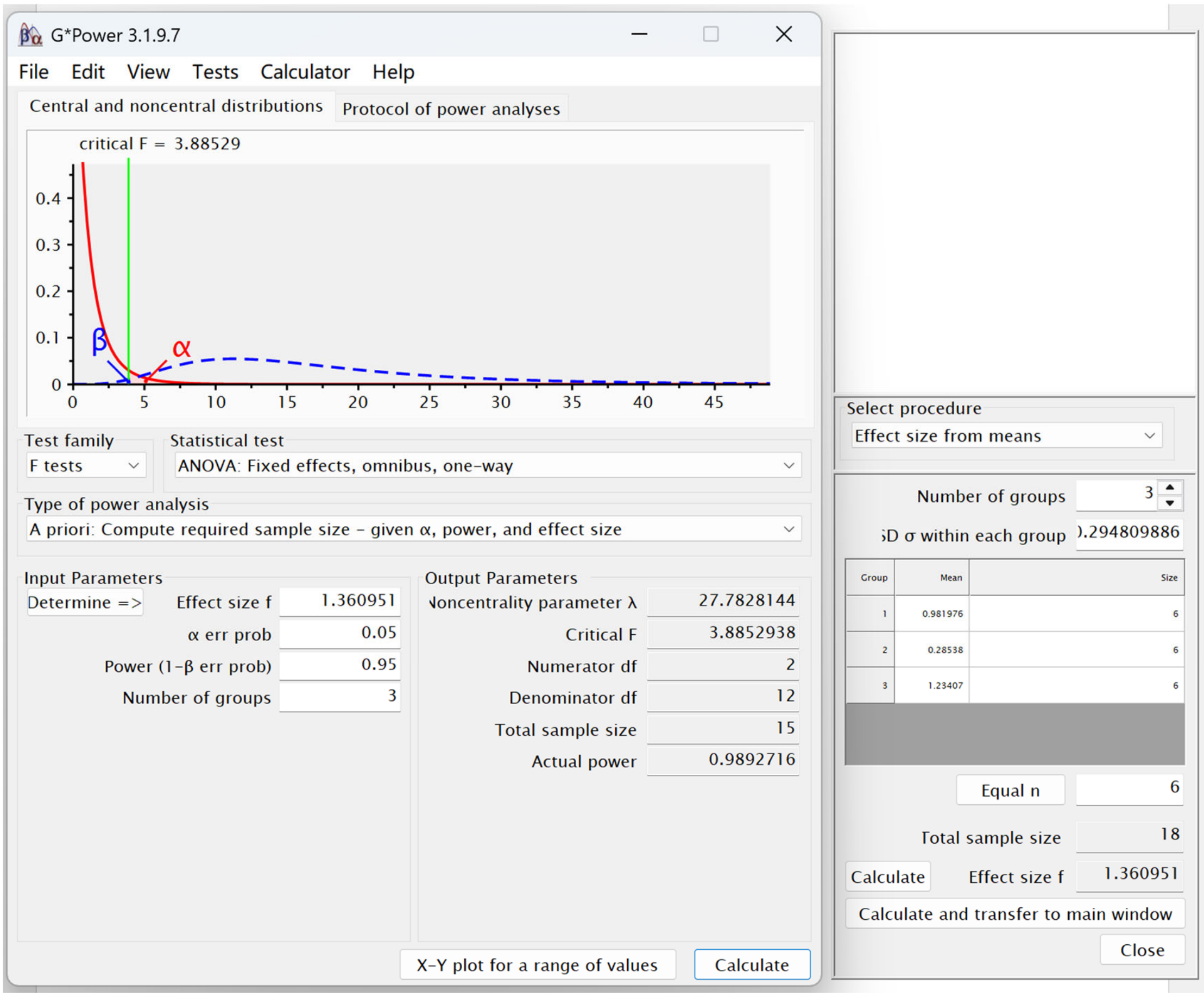

We performed an a priori power analysis using G*Power (α = 0.05, power=0.95) based on the observed variability and group differences. The analysis indicated that achieving a statistical power of 0.95 would require a total sample size of 15 animals. We used 18 mice.

Supplementary Fig 2.A

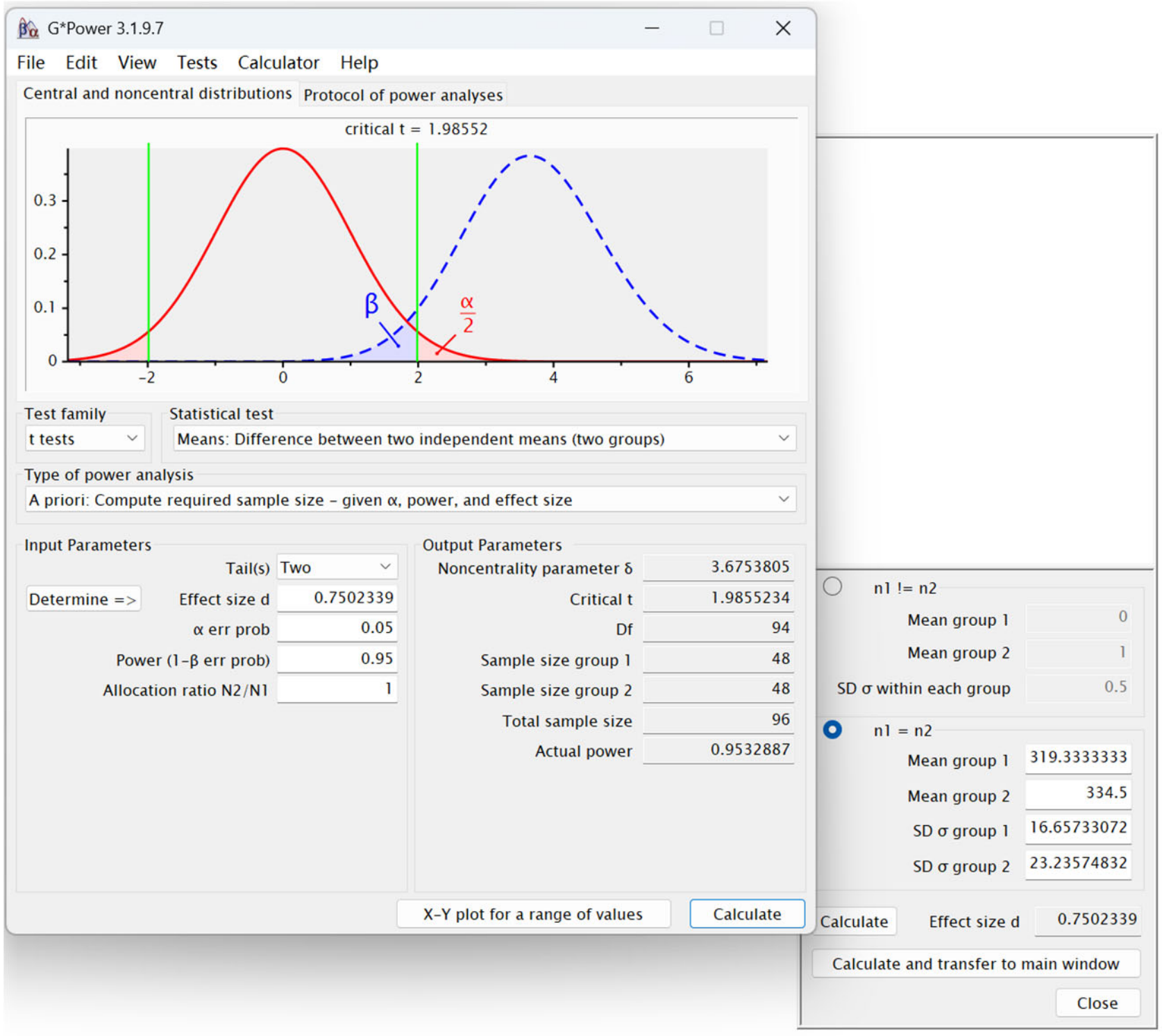

We performed an a priori power analysis using G*Power (α = 0.05, power=0.95) based on the observed variability and group differences. The analysis indicated that achieving a statistical power of 0.95 would require at least 96 animals for the number of GFAP cells in CA1. This large sample size is not feasible or ethically justified in animal research and is far beyond the standard practice in the field. Our sample size is consistent with previous studies using similar endpoints[1,2]. Therefore, the absence of statistical significance in our study likely reflects a modest or biologically limited effect rather than insufficient statistical power.

(1) Reynolds J, Huang M, Li Y, et al. Constitutive knockout of interleukin-6 ameliorates memory deficits and entorhinal astrocytosis in the MRL/lpr mouse model of neuropsychiatric lupus. *J Neuroinflammation*. 2024;21(1):89. Published 2024 Apr 10. doi:10.1186/s12974-024-03085-9.

(2) Bashir ST, Redden CR, Raj K, et al. Endometriosis leads to central nervous system-wide glial activation in a mouse model of endometriosis. J Neuroinflammation. 2023;20(1):59. Published 2023 Mar 6. doi:10.1186/s12974-023-02713-0

Supplementary Fig 2.B

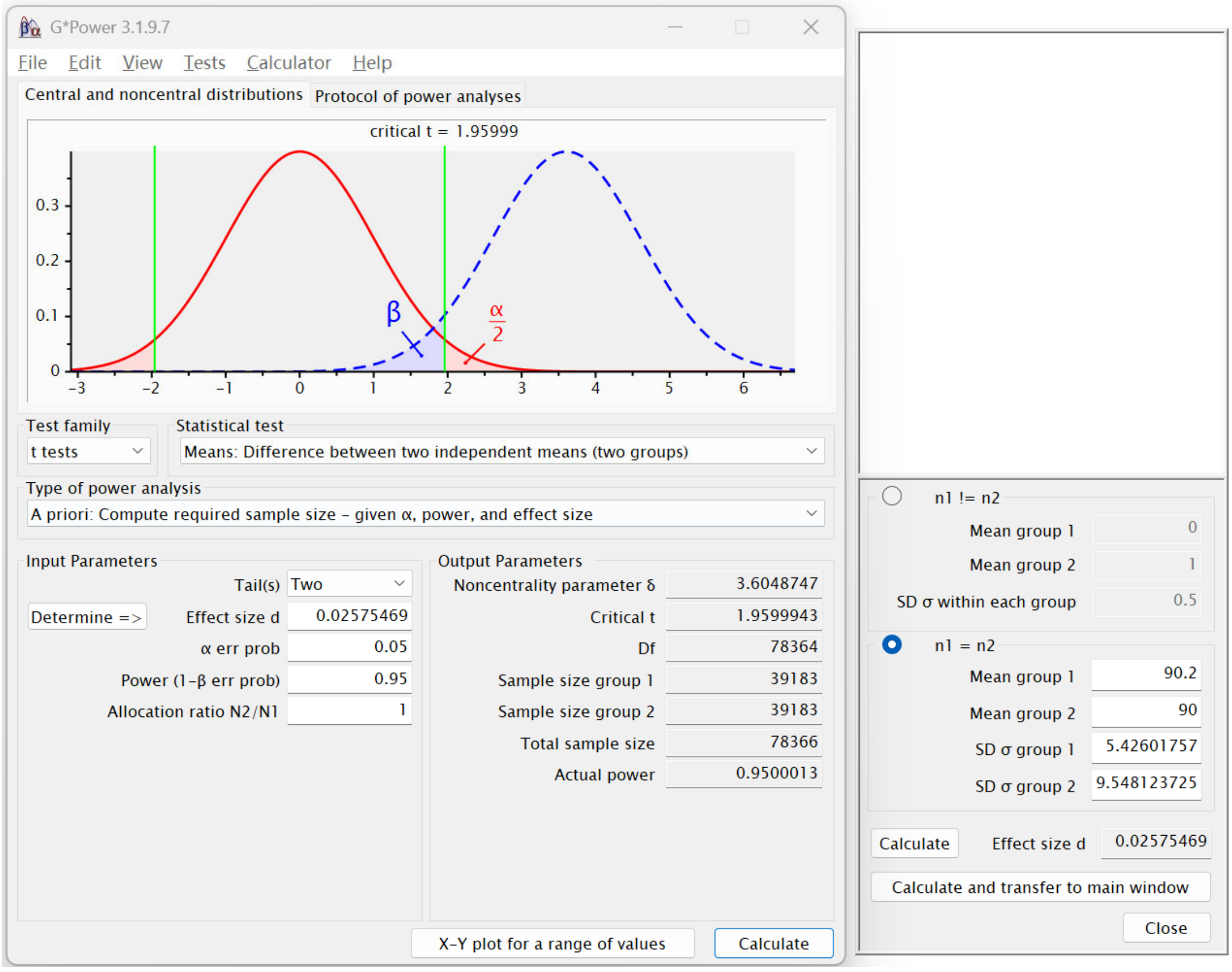

We performed an a priori power analysis using G*Power (α = 0.05, power=0.95) based on the observed variability and group differences. The analysis indicated that achieving a statistical power of 0.95 would require at least 616 animals for the number of GFAP cells in stratum oriens. This large sample size is not feasible or ethically justified in animal research and is far beyond the standard practice in the field. Our sample size is consistent with previous studies using similar endpoints[1,2]. Therefore, the absence of statistical significance in our study likely reflects a modest or biologically limited effect rather than insufficient statistical power.

(1) Reynolds J, Huang M, Li Y, et al. Constitutive knockout of interleukin-6 ameliorates memory deficits and entorhinal astrocytosis in the MRL/lpr mouse model of neuropsychiatric lupus. *J Neuroinflammation*. 2024;21(1):89. Published 2024 Apr 10. doi:10.1186/s12974-024-03085-9.

(2) Bashir ST, Redden CR, Raj K, et al. Endometriosis leads to central nervous system-wide glial activation in a mouse model of endometriosis. J Neuroinflammation. 2023;20(1):59. Published 2023 Mar 6. doi:10.1186/s12974-023-02713-0

Supplementary Fig 2.C

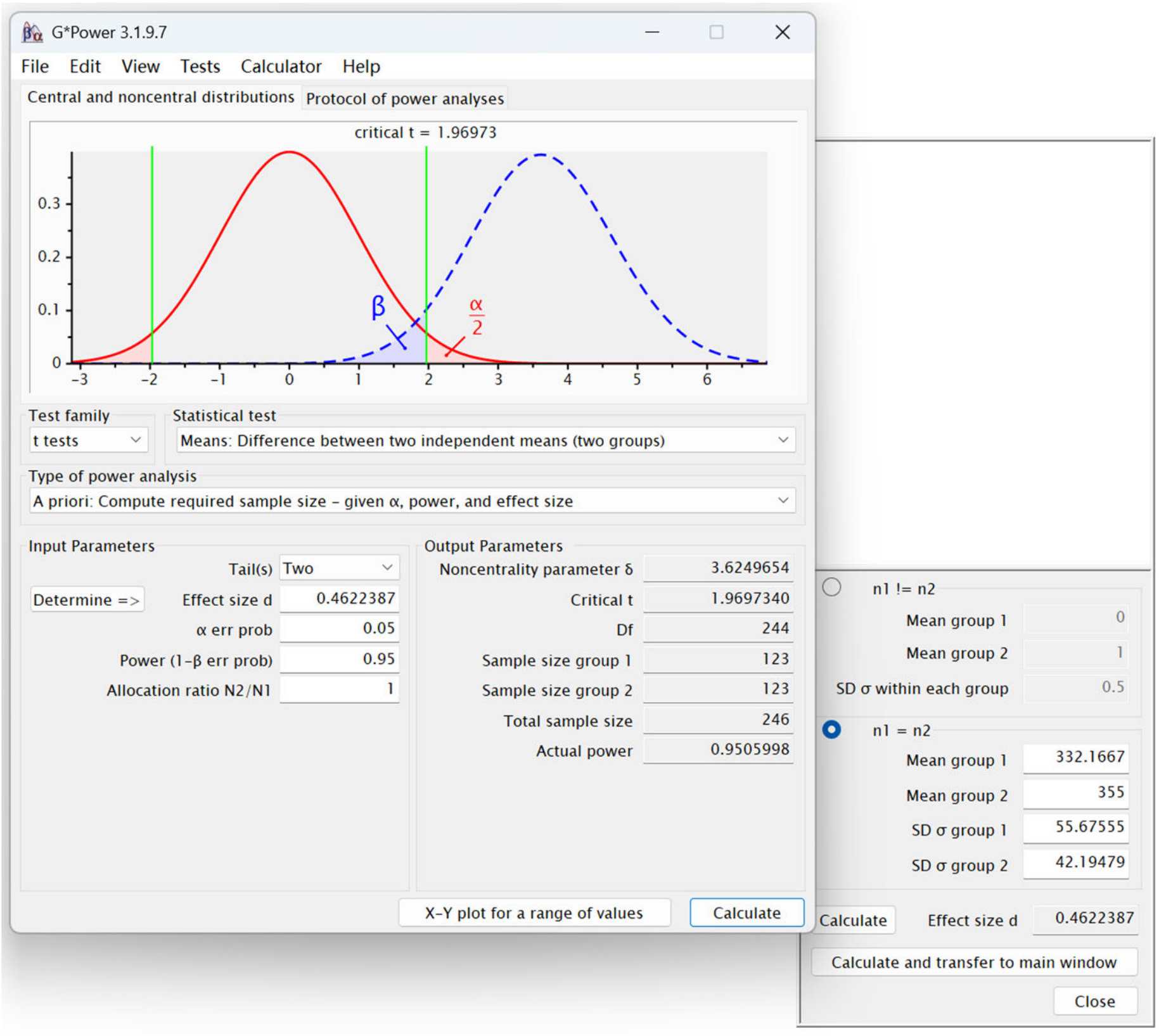

We performed an a priori power analysis using G*Power (α = 0.05, power=0.95) based on the observed variability and group differences. The analysis indicated that achieving a statistical power of 0.95 would require at least 246 animals for the number of S100β cells in CA1. This large sample size is not feasible or ethically justified in animal research and is far beyond the standard practice in the field. Our sample size is consistent with previous studies using similar endpoints[1,2]. Therefore, the absence of statistical significance in our study likely reflects a modest or biologically limited effect rather than insufficient statistical power.

(1) Reynolds J, Huang M, Li Y, et al. Constitutive knockout of interleukin-6 ameliorates memory deficits and entorhinal astrocytosis in the MRL/lpr mouse model of neuropsychiatric lupus. *J Neuroinflammation*. 2024;21(1):89. Published 2024 Apr 10. doi:10.1186/s12974-024-03085-9.

(2) Bashir ST, Redden CR, Raj K, et al. Endometriosis leads to central nervous system-wide glial activation in a mouse model of endometriosis. J Neuroinflammation. 2023;20(1):59. Published 2023 Mar 6. doi:10.1186/s12974-023-02713-0

Supplementary Fig 2.D

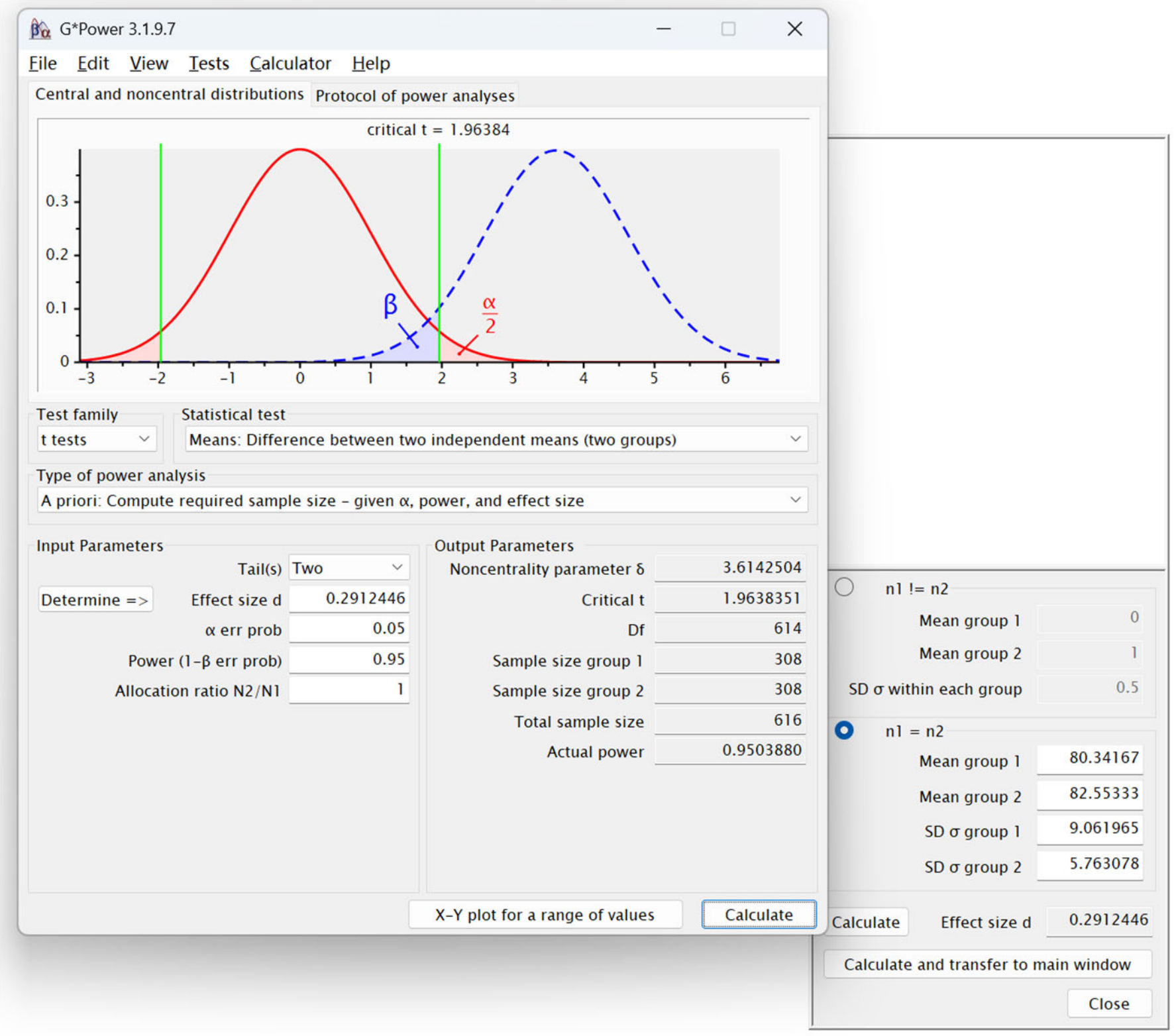

We performed an a priori power analysis using G*Power (α = 0.05, power=0.95) based on the observed variability and group differences. The analysis indicated that achieving a statistical power of 0.95 would require at least 616 animals for the number of S100β cells in stratum oriens. This large sample size is not feasible or ethically justified in animal research and is far beyond the standard practice in the field. Our sample size is consistent with previous studies using similar endpoints[1,2]. Therefore, the absence of statistical significance in our study likely reflects a modest or biologically limited effect rather than insufficient statistical power.

(1) Reynolds J, Huang M, Li Y, et al. Constitutive knockout of interleukin-6 ameliorates memory deficits and entorhinal astrocytosis in the MRL/lpr mouse model of neuropsychiatric lupus. *J Neuroinflammation*. 2024;21(1):89. Published 2024 Apr 10. doi:10.1186/s12974-024-03085-9.

(2) Bashir ST, Redden CR, Raj K, et al. Endometriosis leads to central nervous system-wide glial activation in a mouse model of endometriosis. J Neuroinflammation. 2023;20(1):59. Published 2023 Mar 6. doi:10.1186/s12974-023-02713-0

Supplementary Fig 3.A

This figure was shown as action potentials of neurons and astrocytes and we do not need to do power analysis.

Supplementary Fig 3.B

This figure was shown as current amplitudes in neurons or astrocytes and we do not need to do power analysis.

Supplementary Fig 3.C

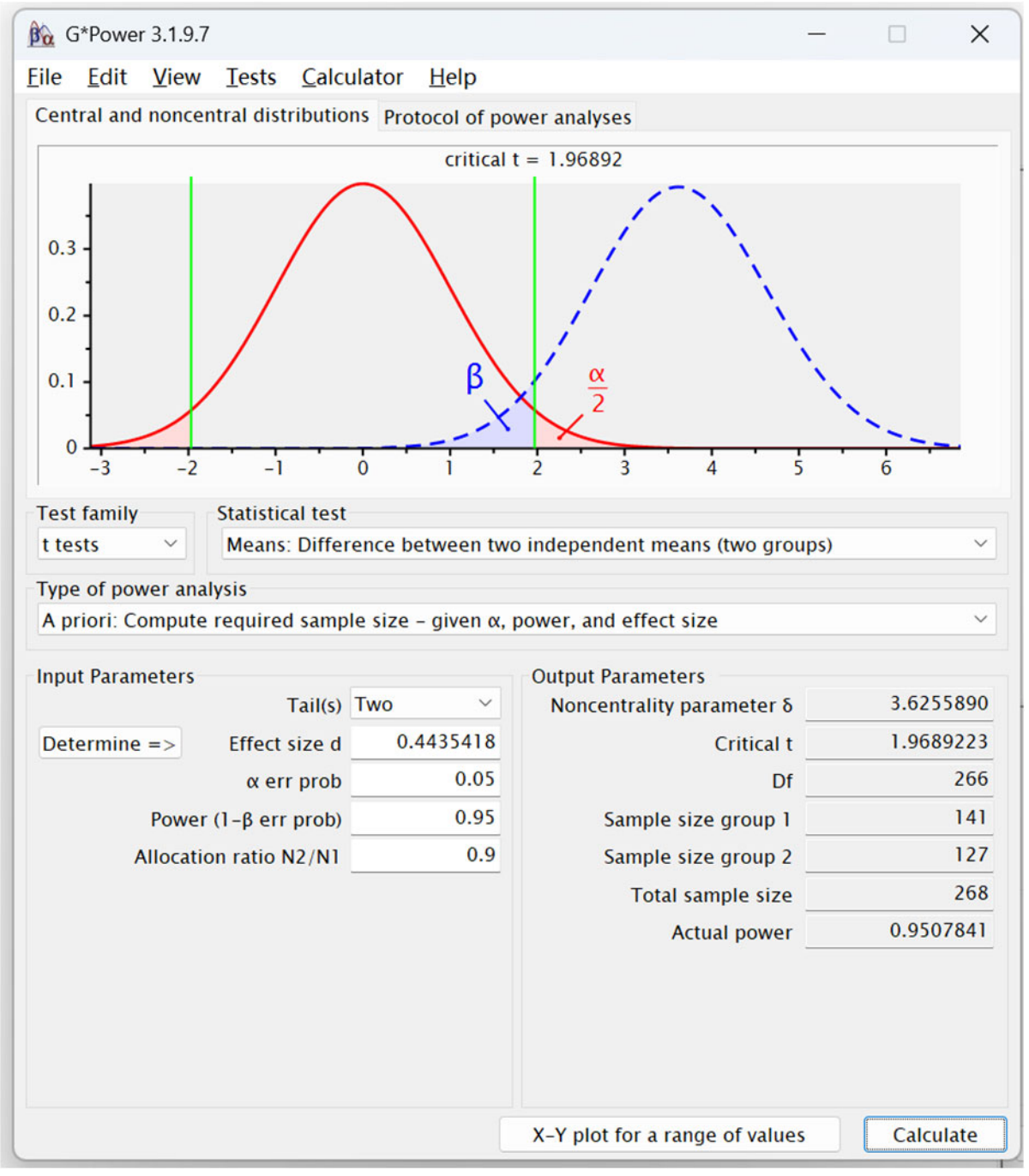

We performed an a priori power analysis using G*Power (α = 0.05, power=0.95) based on the observed variability and group differences. The analysis indicated that achieving a statistical power of 0.95 would require at least 268 animals for neuron current detection stimulated by AMPA on control and CUMS mice. This large sample size is not feasible or ethically justified in animal research and is far beyond the standard practice in the field. Our sample size is consistent with previous studies using similar endpoints[1,2,3].

[1] Zhang Y, Yin HY, Rubini P, Illes P, Tang Y. ATP indirectly stimulates hippocampal CA1 and CA3 pyramidal neurons via the activation of neighboring P2X7 receptor-bearing astrocytes and NG2 glial cells, respectively. Front Pharmacol. 2022;13:944541. Published 2022 Jul 22. doi:10.3389/fphar.2022.944541

[2] Khan MT, Deussing J, Tang Y, Illes P. Astrocytic rather than neuronal P2X7

receptors modulate the function of the tri-synaptic network in the rodent hippocampus. *Brain Res Bull*. 2019;151:164-173. doi:10.1016/j.brainresbull.2018.07.016

[3] Stephan G, Huang L, Tang Y, et al. The ASIC3/P2X3 cognate receptor is a pain-relevant and ligand-gated cationic channel. Nat Commun. 2018;9(1):1354. Published 2018 Apr 10. doi:10.1038/s41467-018-03728-5

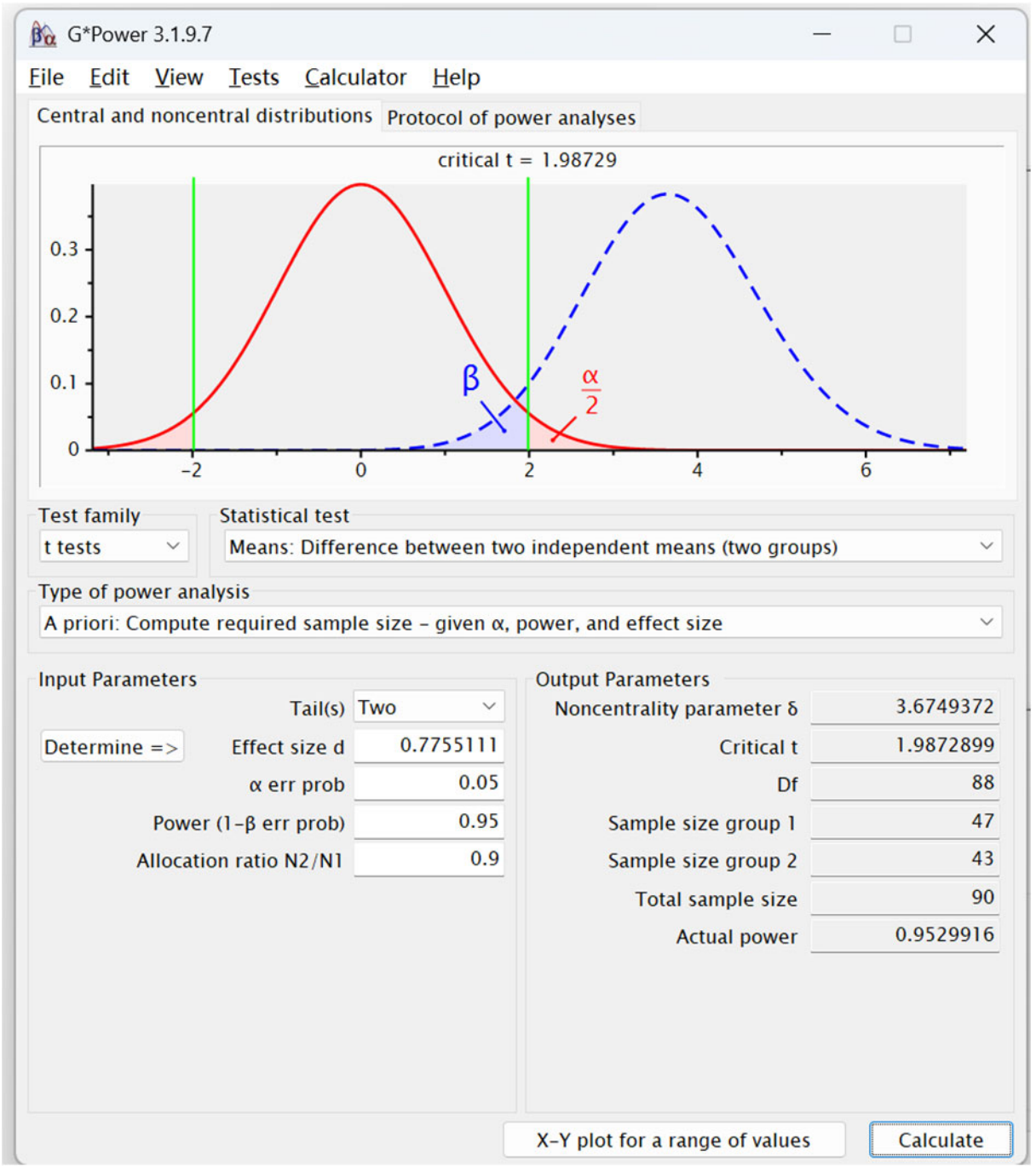

We performed an a priori power analysis using G*Power (α = 0.05, power=0.95) based on the observed variability and group differences. The analysis indicated that achieving a statistical power of 0.95 would require at least 90 animals for neuron current detection stimulated by BzATP on control and CUMS mice. This large sample size is not feasible or ethically justified in animal research and is far beyond the standard practice in the field. Our sample size is consistent with previous studies using similar endpoints[1,2,3].

[1] Zhang Y, Yin HY, Rubini P, Illes P, Tang Y. ATP indirectly stimulates hippocampal CA1 and CA3 pyramidal neurons via the activation of neighboring P2X7 receptor-bearing astrocytes and NG2 glial cells, respectively. Front Pharmacol. 2022;13:944541. Published 2022 Jul 22. doi:10.3389/fphar.2022.944541

[2] Khan MT, Deussing J, Tang Y, Illes P. Astrocytic rather than neuronal P2X7 receptors modulate the function of the tri-synaptic network in the rodent

hippocampus. *Brain Res Bull*. 2019;151:164-173. doi:10.1016/j.brainresbull.2018.07.016

[3] Stephan G, Huang L, Tang Y, et al. The ASIC3/P2X3 cognate receptor is a pain-relevant and ligand-gated cationic channel. Nat Commun. 2018;9(1):1354. Published 2018 Apr 10. doi:10.1038/s41467-018-03728-5

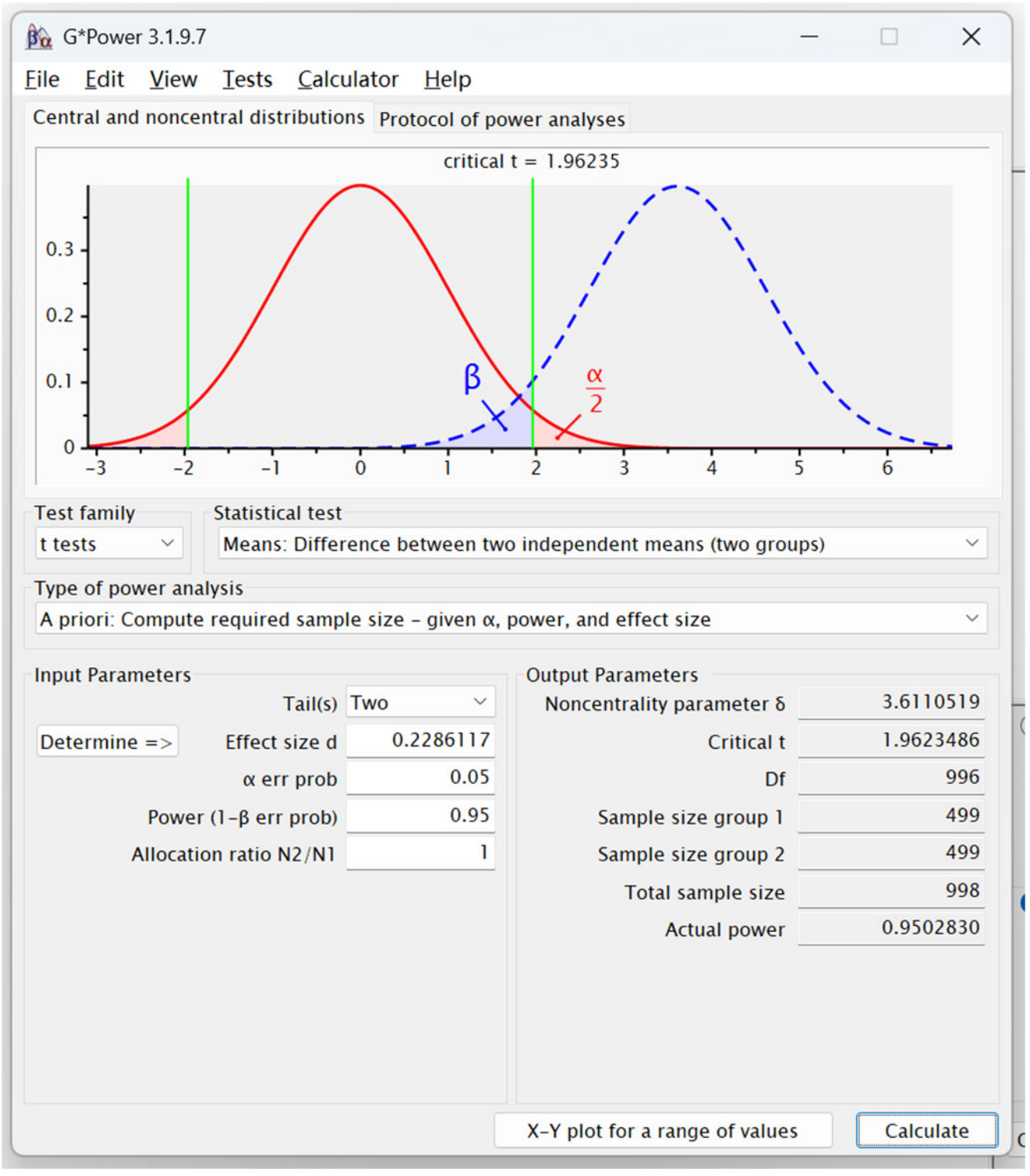

We performed an a priori power analysis using G*Power (α = 0.05, power=0.95) based on the observed variability and group differences. The analysis indicated that achieving a statistical power of 0.95 would require at least 998 animals for neuron current detection stimulated by Musc on control and CUMS mice. This large sample size is not feasible or ethically justified in animal research and is far beyond the standard practice in the field. Our sample size is consistent with previous studies using similar endpoints[1,2,3].

[1] Zhang Y, Yin HY, Rubini P, Illes P, Tang Y. ATP indirectly stimulates hippocampal CA1 and CA3 pyramidal neurons via the activation of neighboring P2X7 receptor-bearing astrocytes and NG2 glial cells, respectively. Front Pharmacol. 2022;13:944541. Published 2022 Jul 22. doi:10.3389/fphar.2022.944541

[2] Khan MT, Deussing J, Tang Y, Illes P. Astrocytic rather than neuronal P2X7

receptors modulate the function of the tri-synaptic network in the rodent hippocampus. *Brain Res Bull*. 2019;151:164-173. doi:10.1016/j.brainresbull.2018.07.016

[3] Stephan G, Huang L, Tang Y, et al. The ASIC3/P2X3 cognate receptor is a pain-relevant and ligand-gated cationic channel. Nat Commun. 2018;9(1):1354. Published 2018 Apr 10. doi:10.1038/s41467-018-03728-5

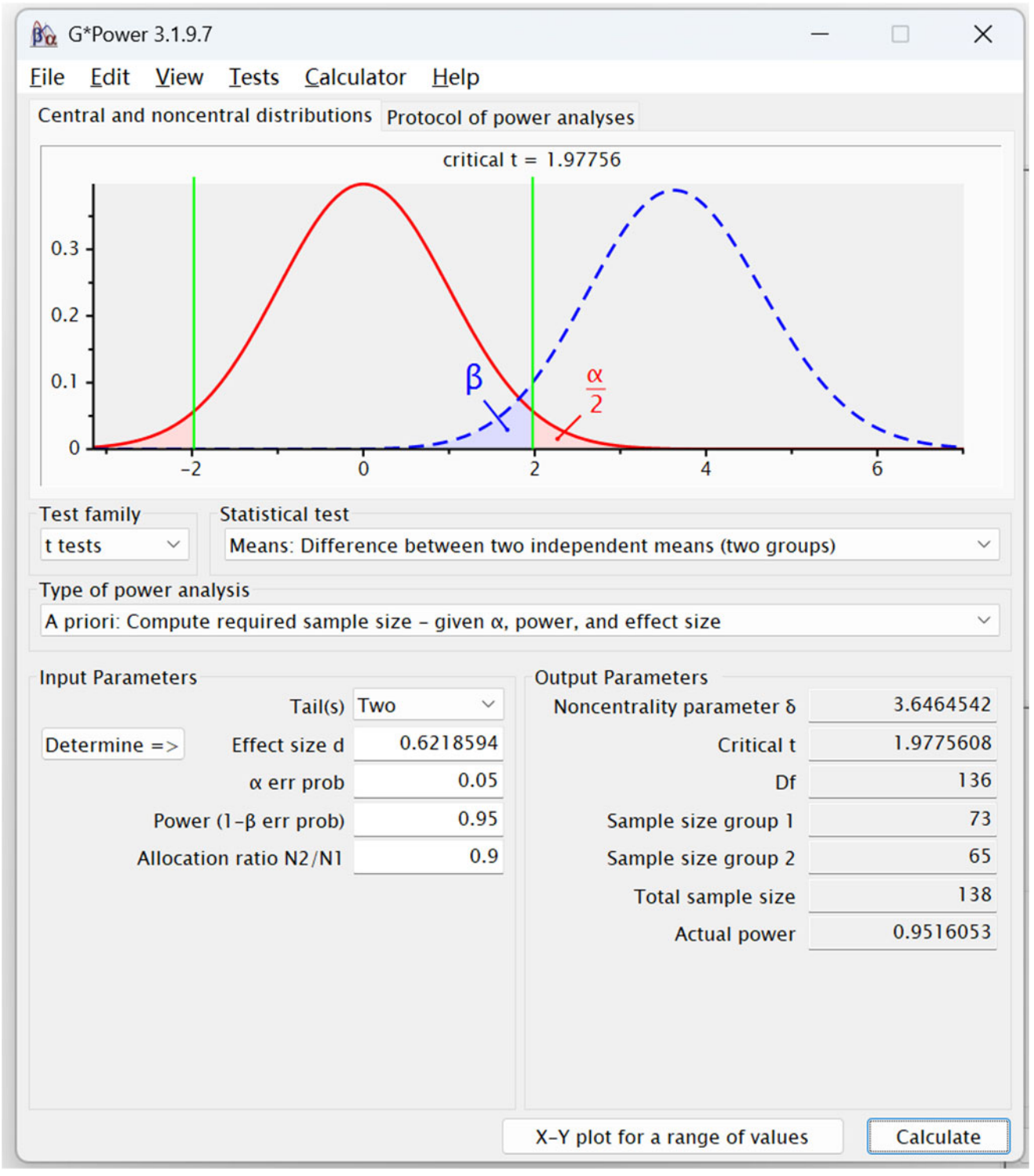

We performed an a priori power analysis using G*Power (α = 0.05, power=0.95) based on the observed variability and group differences. The analysis indicated that achieving a statistical power of 0.95 would require at least 138 animals for astrocyte current detection stimulated by AMPA on control and CUMS mice. This large sample size is not feasible or ethically justified in animal research and is far beyond the standard practice in the field. Our sample size is consistent with previous studies using similar endpoints[1,2,3].

[1] Zhang Y, Yin HY, Rubini P, Illes P, Tang Y. ATP indirectly stimulates hippocampal CA1 and CA3 pyramidal neurons via the activation of neighboring P2X7 receptor-bearing astrocytes and NG2 glial cells, respectively. Front Pharmacol. 2022;13:944541. Published 2022 Jul 22. doi:10.3389/fphar.2022.944541

[2] Khan MT, Deussing J, Tang Y, Illes P. Astrocytic rather than neuronal P2X7 receptors modulate the function of the tri-synaptic network in the rodent hippocampus. *Brain Res Bull*. 2019;151:164-173. doi:10.1016/j.brainresbull.2018.07.016

[3] Stephan G, Huang L, Tang Y, et al. The ASIC3/P2X3 cognate receptor is a pain-relevant and ligand-gated cationic channel. Nat Commun. 2018;9(1):1354. Published 2018 Apr 10. doi:10.1038/s41467-018-03728-5

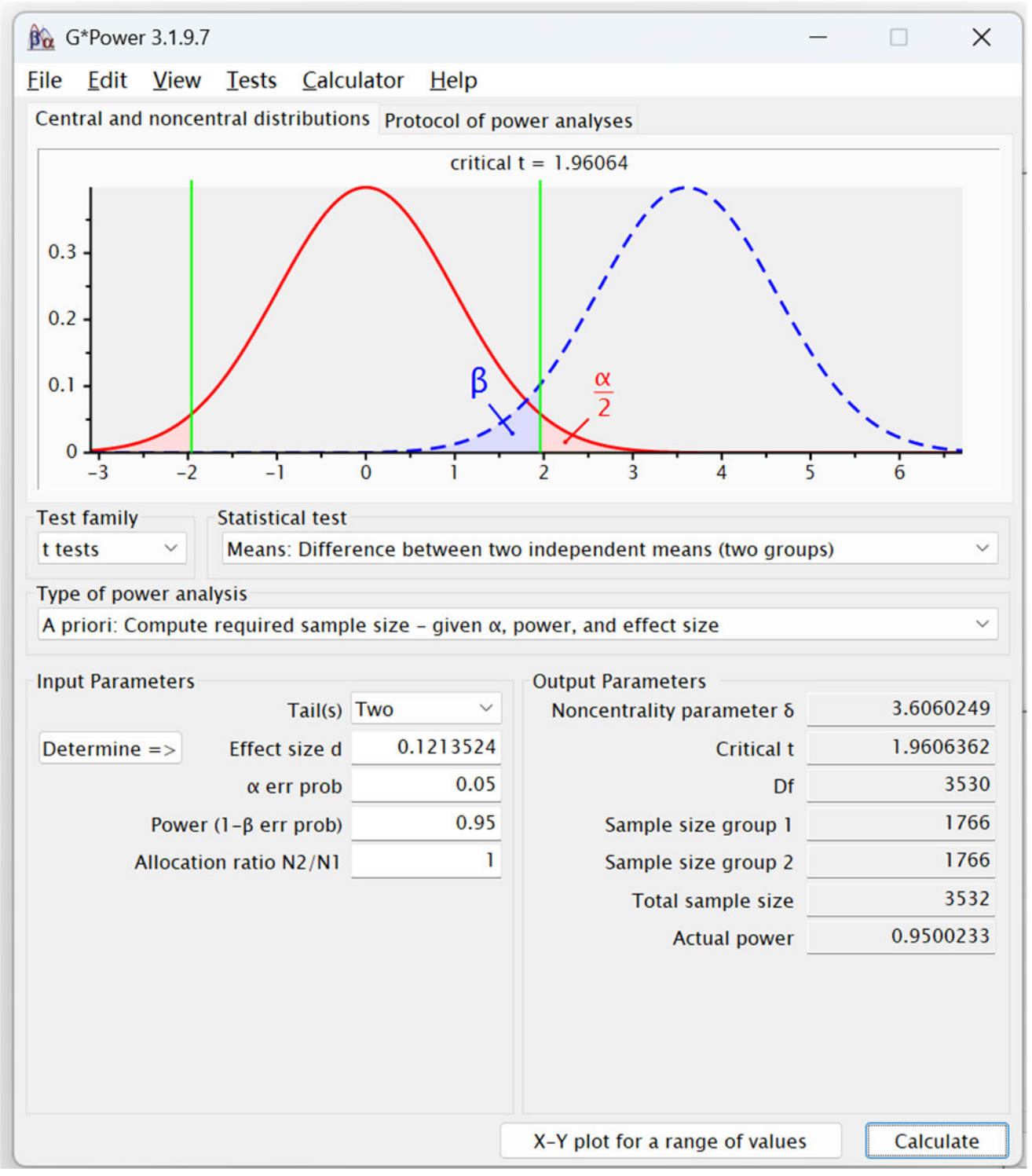

We performed an a priori power analysis using G*Power (α = 0.05, power=0.95) based on the observed variability and group differences. The analysis indicated that achieving a statistical power of 0.95 would require at least 3532 animals for astrocyte current detection stimulated by BzATP on control and CUMS mice. This large sample size is not feasible or ethically justified in animal research and is far beyond the standard practice in the field. Our sample size is consistent with previous studies using similar endpoints[1,2,3].

[1] Zhang Y, Yin HY, Rubini P, Illes P, Tang Y. ATP indirectly stimulates hippocampal CA1 and CA3 pyramidal neurons via the activation of neighboring P2X7 receptor-bearing astrocytes and NG2 glial cells, respectively. Front Pharmacol. 2022;13:944541. Published 2022 Jul 22. doi:10.3389/fphar.2022.944541

[2] Khan MT, Deussing J, Tang Y, Illes P. Astrocytic rather than neuronal P2X7 receptors modulate the function of the tri-synaptic network in the rodent hippocampus. *Brain Res Bull*. 2019;151:164-173. doi:10.1016/j.brainresbull.2018.07.016

[3] Stephan G, Huang L, Tang Y, et al. The ASIC3/P2X3 cognate receptor is a pain-relevant and ligand-gated cationic channel. Nat Commun. 2018;9(1):1354. Published 2018 Apr 10. doi:10.1038/s41467-018-03728-5

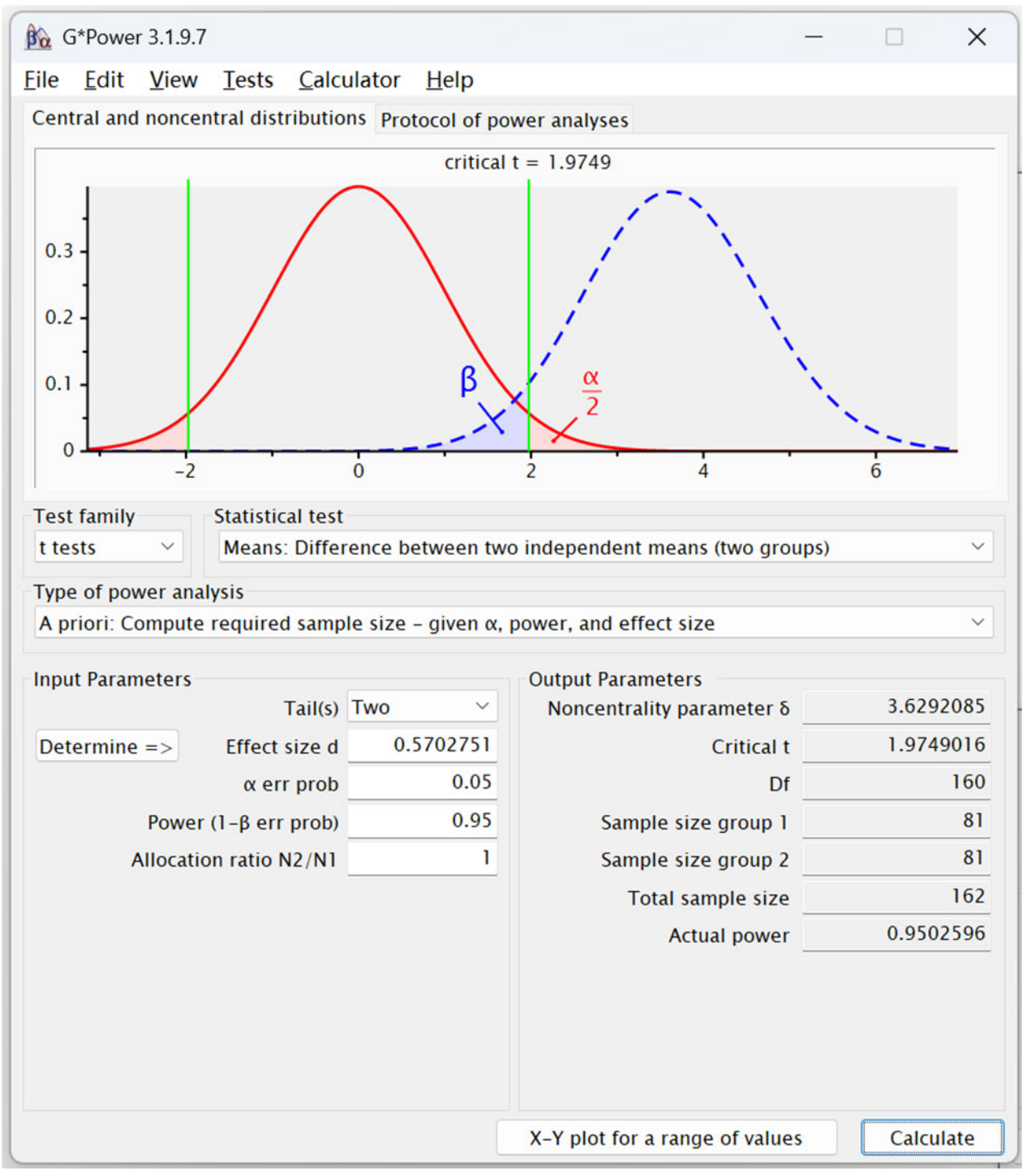

We performed an a priori power analysis using G*Power (α = 0.05, power=0.95) based on the observed variability and group differences. The analysis indicated that achieving a statistical power of 0.95 would require at least 162 animals for astrocyte current detection stimulated by Musc on control and CUMS mice. This large sample size is not feasible or ethically justified in animal research and is far beyond the standard practice in the field. Our sample size is consistent with previous studies using similar endpoints[1,2,3].

[1] Zhang Y, Yin HY, Rubini P, Illes P, Tang Y. ATP indirectly stimulates hippocampal CA1 and CA3 pyramidal neurons via the activation of neighboring P2X7 receptor-bearing astrocytes and NG2 glial cells, respectively. Front Pharmacol. 2022;13:944541. Published 2022 Jul 22. doi:10.3389/fphar.2022.944541

[2] Khan MT, Deussing J, Tang Y, Illes P. Astrocytic rather than neuronal P2X7 receptors modulate the function of the tri-synaptic network in the rodent hippocampus. *Brain Res Bull*. 2019;151:164-173. doi:10.1016/j.brainresbull.2018.07.016

[3] Stephan G, Huang L, Tang Y, et al. The ASIC3/P2X3 cognate receptor is a pain-relevant and ligand-gated cationic channel. Nat Commun. 2018;9(1):1354. Published 2018 Apr 10. doi:10.1038/s41467-018-03728-5

Suppl. Figure 5. Experimental protocols. (A) In most experiments, an astrocyte (GFAP)-specific or neuron (Syn)-specific knockdown (KD)-, over-, or control- (C)shRNA was injected to the mPFC. After an interval of two weeks, the CUMS (4 weeks), fluoxetine (10 mg/kg i.p. weekly), or EA (shamEA)-treatment (30 min each, daily) was applied. The EA, or shamEA was delivered in an identical manner to the Zusanli acupoint (ST36) and to a non-acupoint around the tail, respectively. After finishing CUMS, FST, TST, SPT, and the open-field test were carried out. Similarly, the mPFC was processed for qPCR and western-blot determinations. (B) In another series of experiments, the above-mentioned virus-complexes were injected to the mPFC, and two weeks afterwards immunohistochemistry, qPCR or western-blot determinations were made. (C) In a third type of experiments, virus-complexes for inducing the synthesis of a genetic $[Ca^{2+}]_i$ indicator were injected to the mPFC. At the same time, an optical fiber was also implanted into the mPFC. Two weeks afterwards $[Ca^{2+}]_i$ microfluorimetry was carried out at the described conditions (see Fig. 6). At the end of the CUMS period, TST, foot shock, EA, or shamEA was applied. The number of individual animals is indicated in each column.

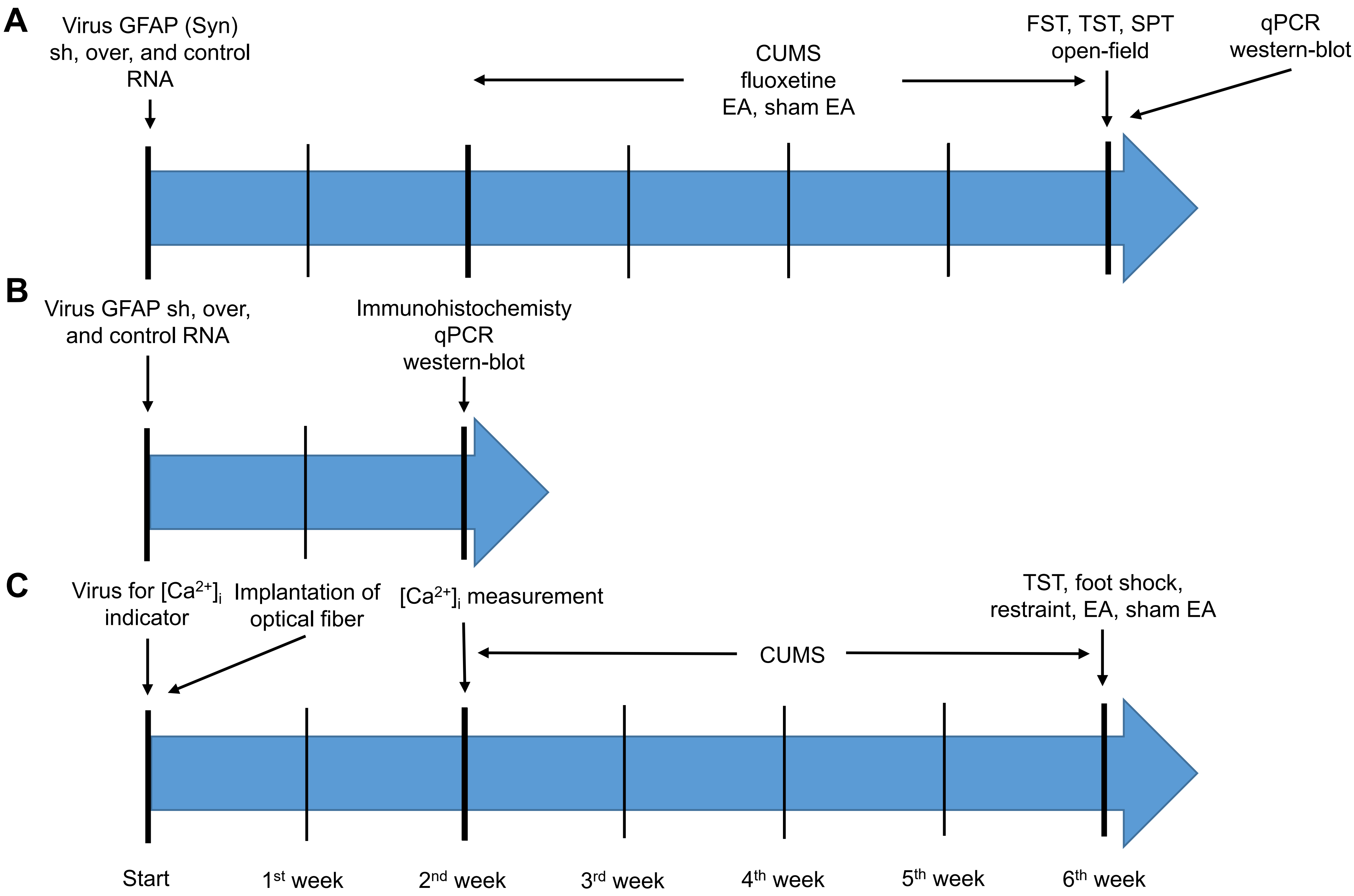
A
Virus GFAP (Syn) sh, over, and control RNA
CUMS
fluoxetine
EA, sham EA
FST, TST, SPT
open-field
qPCR
western-blot
B
Virus GFAP sh, over, and control RNA
Immunohistochemisty
qPCR
western-blot
C
Virus for [Ca2+]i indicator
Implantation of optical fiber
[Ca2+]i measurement
CUMS
TST, foot shock, restraint, EA, sham EA
Start
1st week
2nd week
3rd week
4th week
5th week
6th week

Suppl. Table 1. Random order of stressors applied over the 4-week period demonstrating the unpredictable nature of chronic “unexpected” mild stress (CUMS).

	Time	Monday	Tuesday	Wednesday	Thursday	Friday	Saturday	Sunday
**Week 1**	8:00-11:00	tail suspension		restraint				restraint
	12:00							
	13:00-15:00		swimming in 4°C water		foot shock	cage shaking		
	16:00-18:00							
	19:00-20:00	cage tilt		damp bedding	cage tilt			group housing
**Week 2**	8:00-11:00		tail suspension				foot shock	
	12:00	foot shock						restraint
	13:00-15:00				swimming in 4°C water			
	16:00-18:00		restraint					
	19:00-20:00	noise		water and food deprivation			cage tilt	
**Week 3**	8:00-11:00	tail suspension			cage shaking			foot shock
	12:00							
	13:00-15:00		foot shock	swimming in 4°C water		restraint		
	16:00-18:00							
	19:00-20:00	damp bedding	group housing		noise			cage tilt
**Week 4**	8:00-11:00			restraint				
	12:00	tail suspension				foot shock		
	13:00-15:00		cage shaking		swimming in 4°C water		cage shaking	
	16:00-18:00			tail suspension				
	19:00-20:00	water and food deprivation	noise			cage tilt	damp bedding	

Suppl. Figure 6. Original, uncropped Western Blot images with unadjusted contrast.

CD73

GAPDH

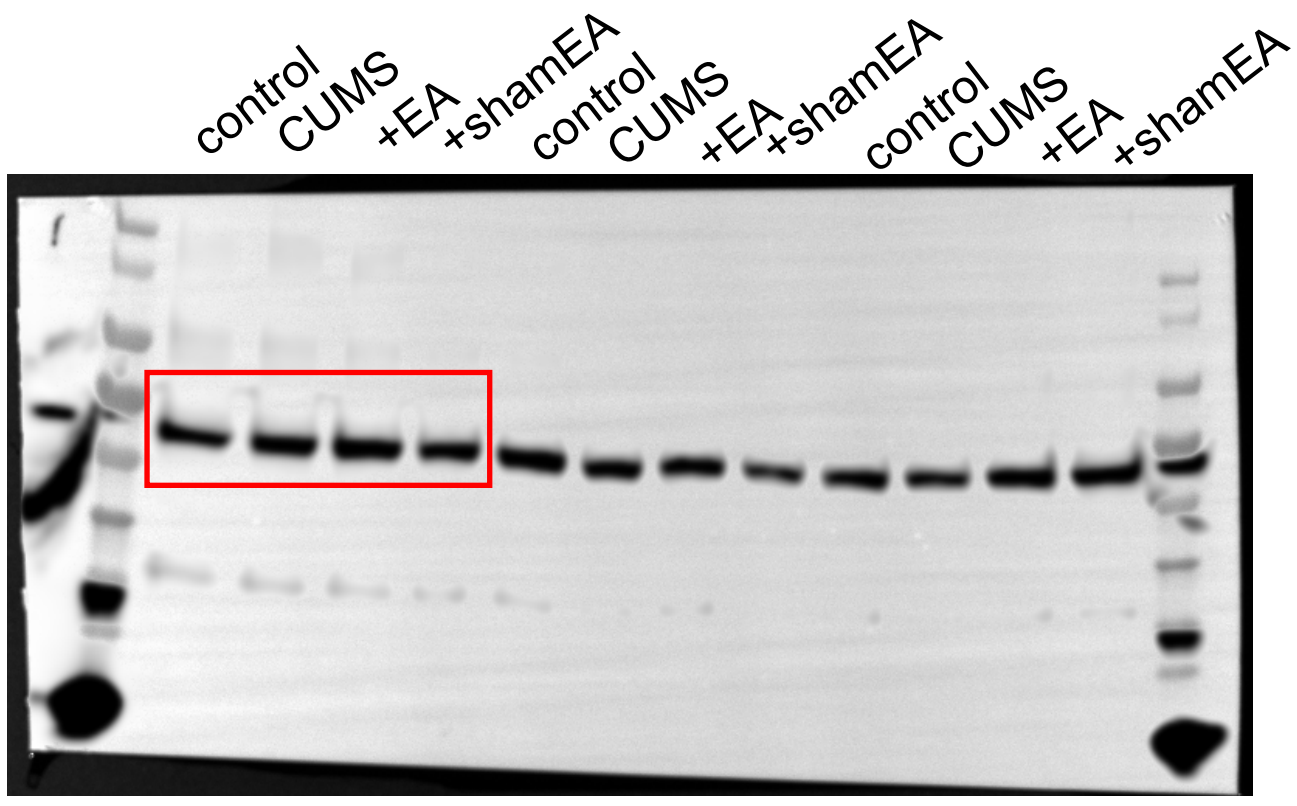
control
CUMS
+EA
+shamEA
control
CUMS
+EA
+shamEA
control
CUMS
+EA
+shamEA

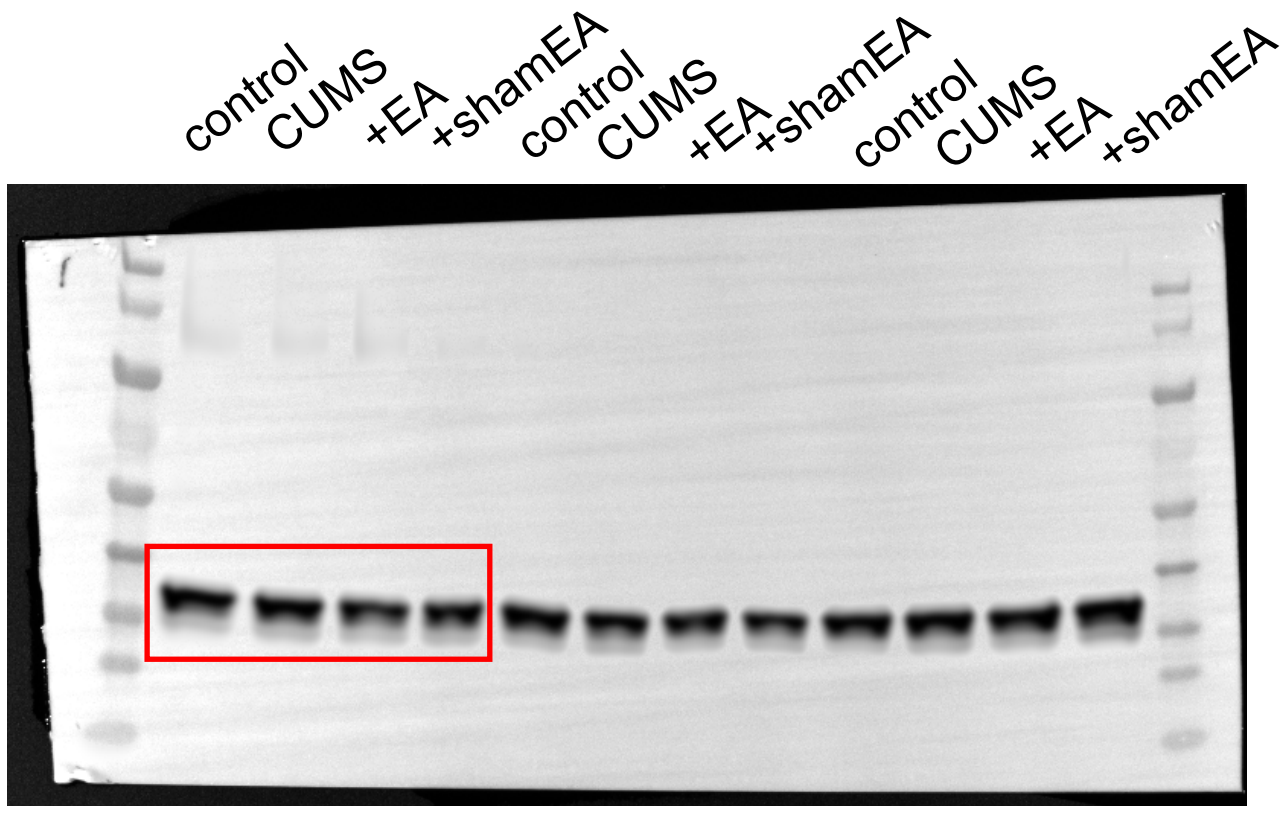
control
CUMS
+EA
+shamEA
control
CUMS
+EA
+shamEA
control
CUMS
+EA
+shamEA

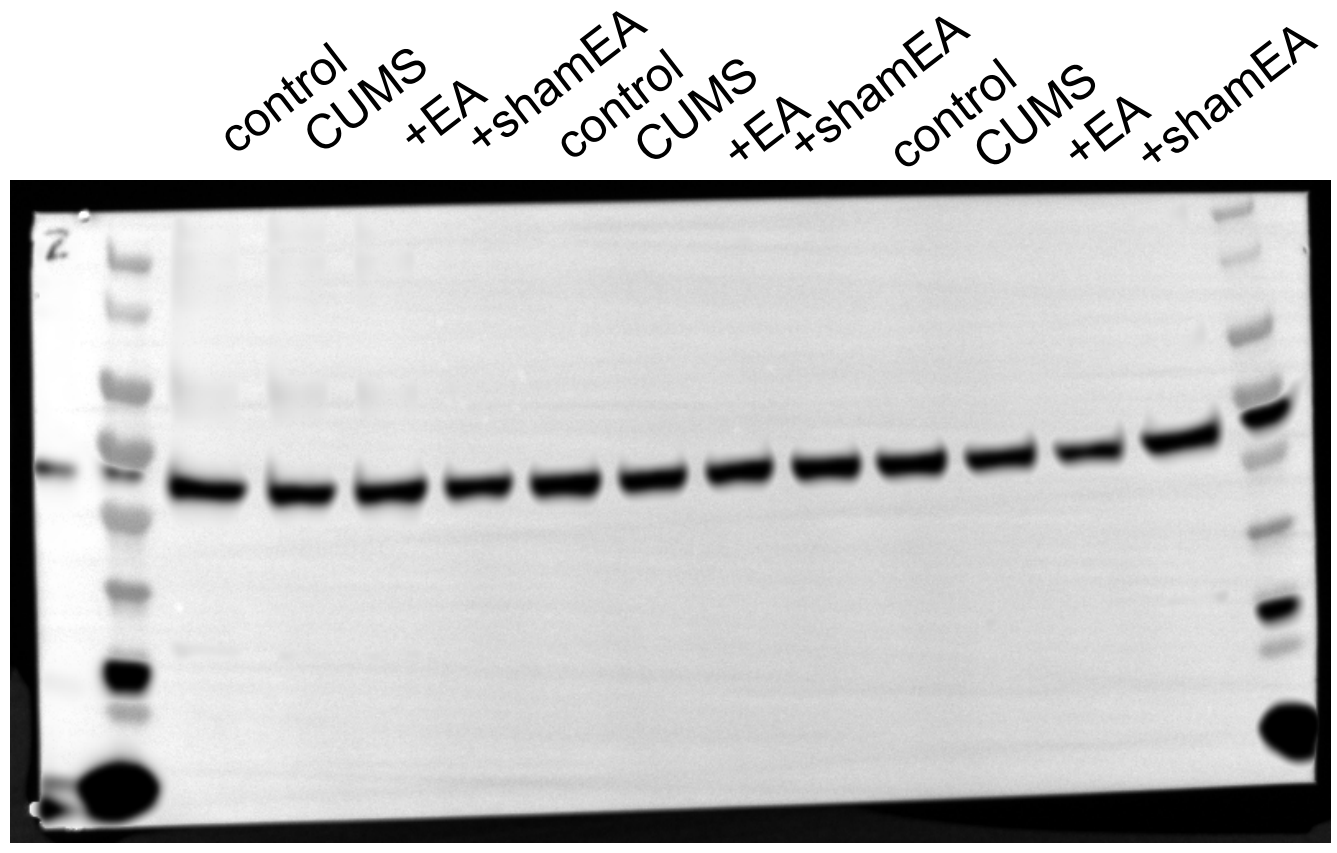
control
CUMS
+EA
+shamEA
control
CUMS
+EA
+shamEA
control
CUMS
+EA
+shamEA

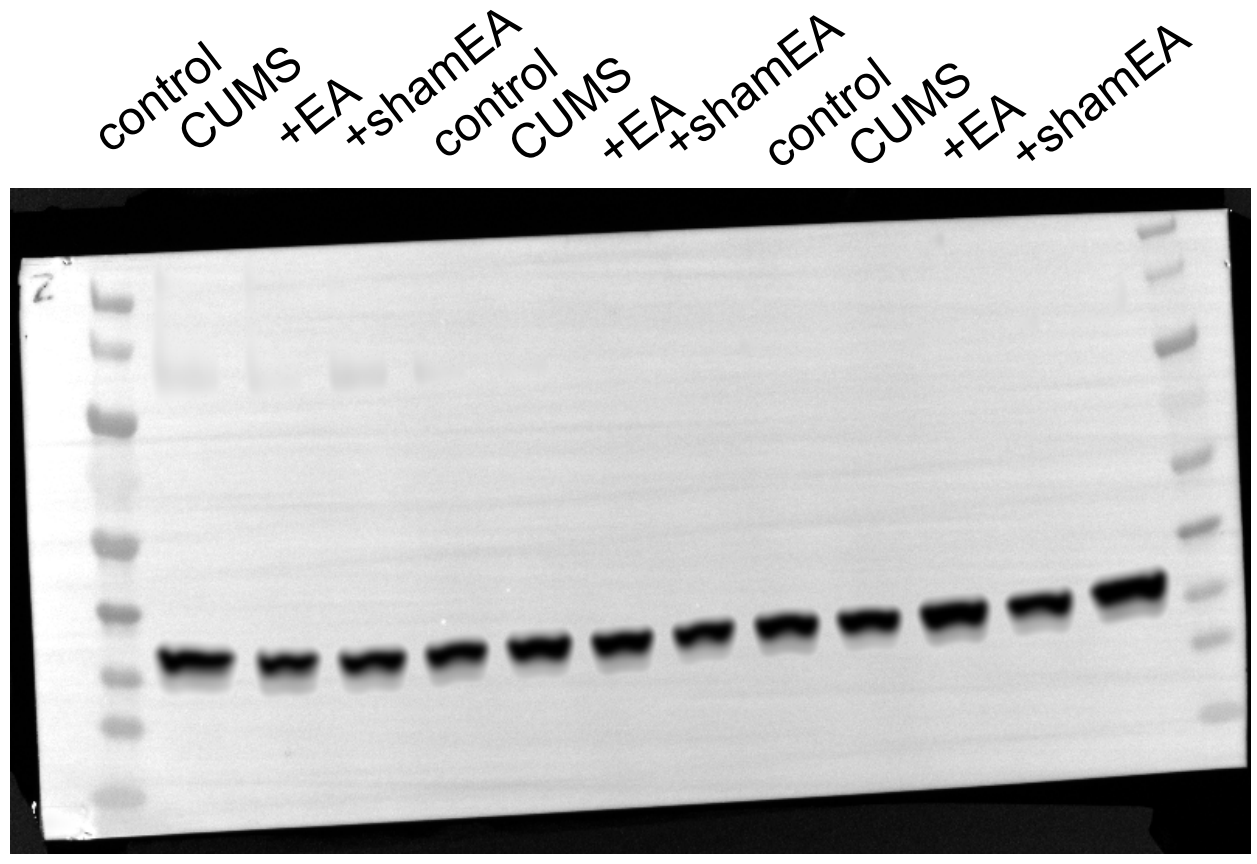
control
CUMS
+EA
+shamEA
control
CUMS
+EA
+shamEA
control
CUMS
+EA
+shamEA

SNAP23

GAPDH

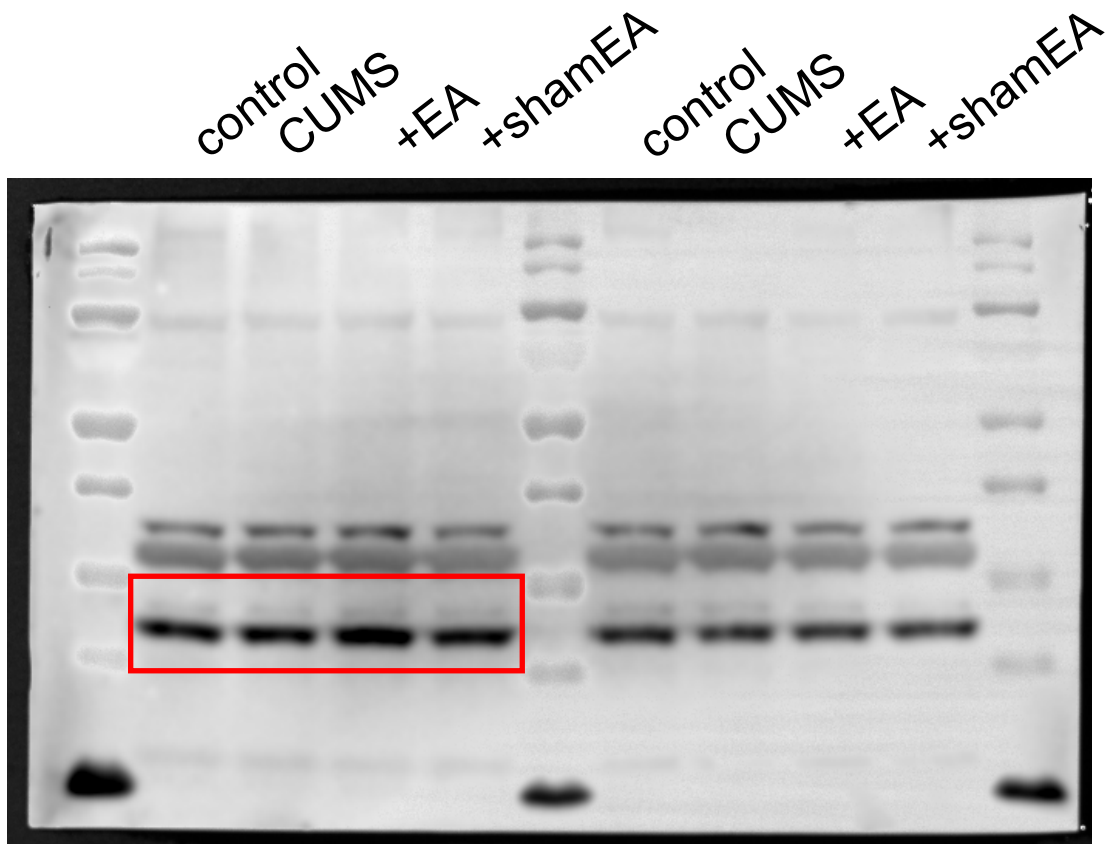

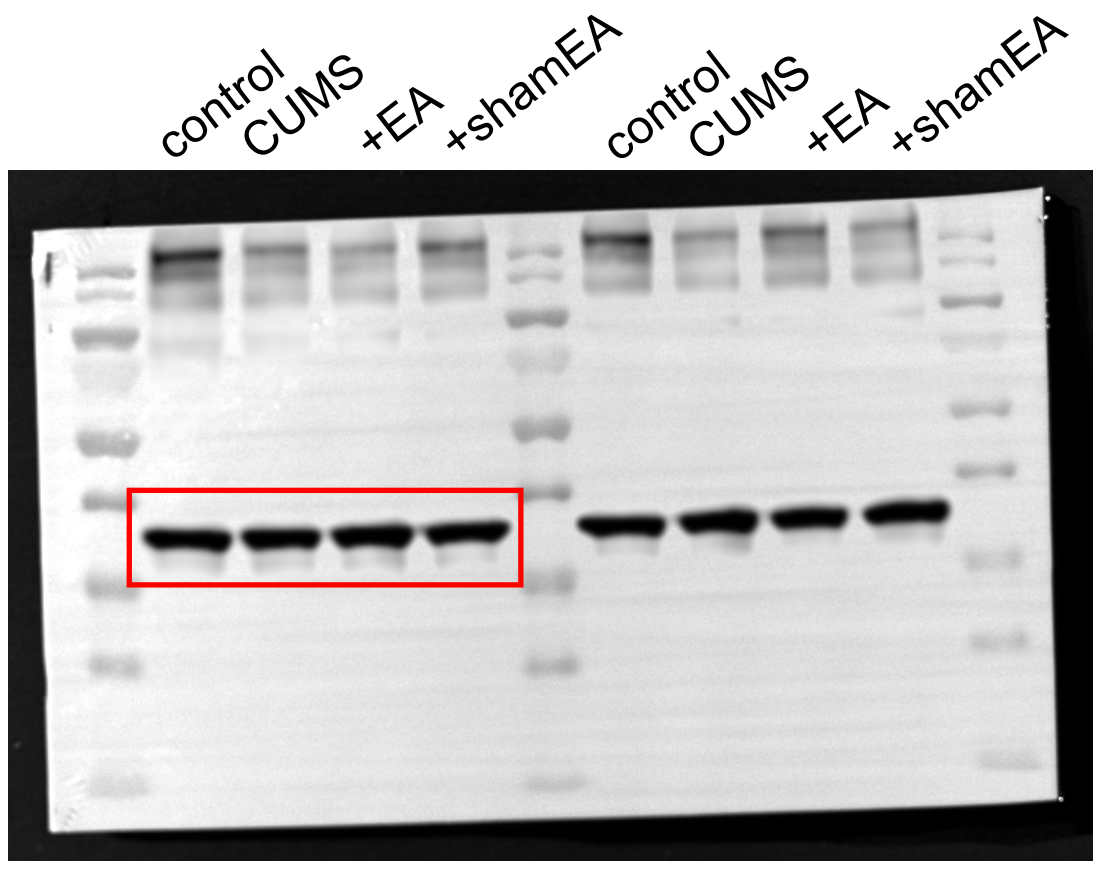

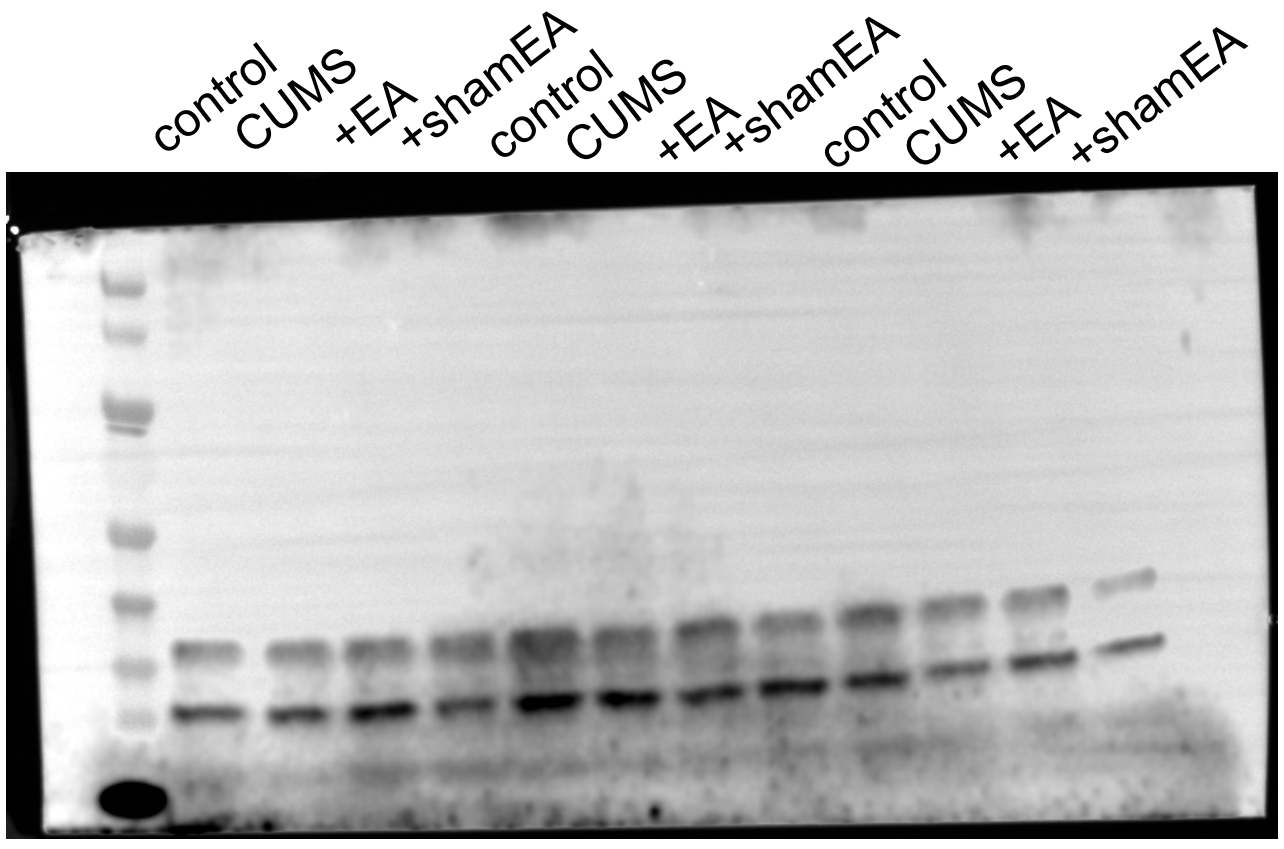

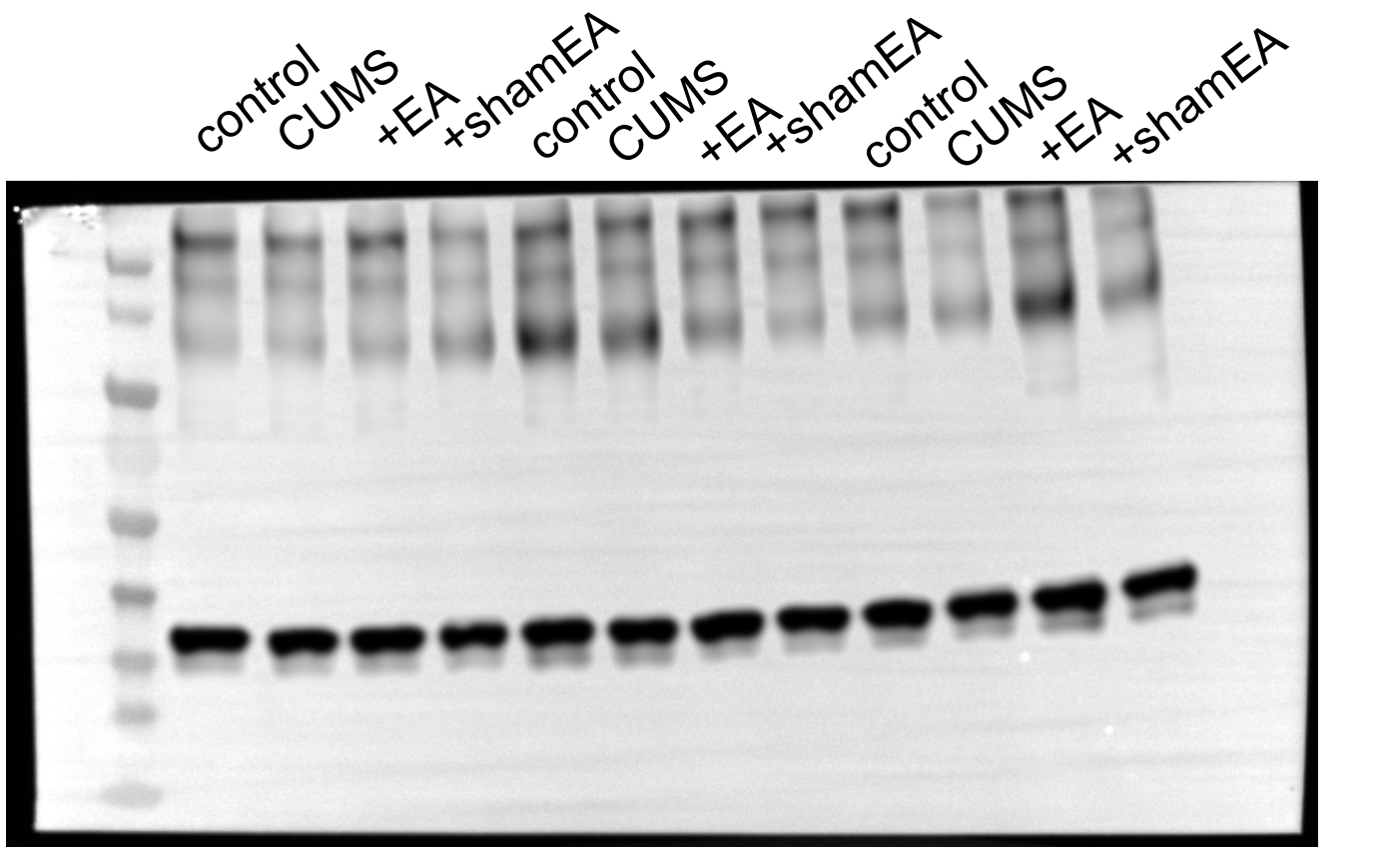

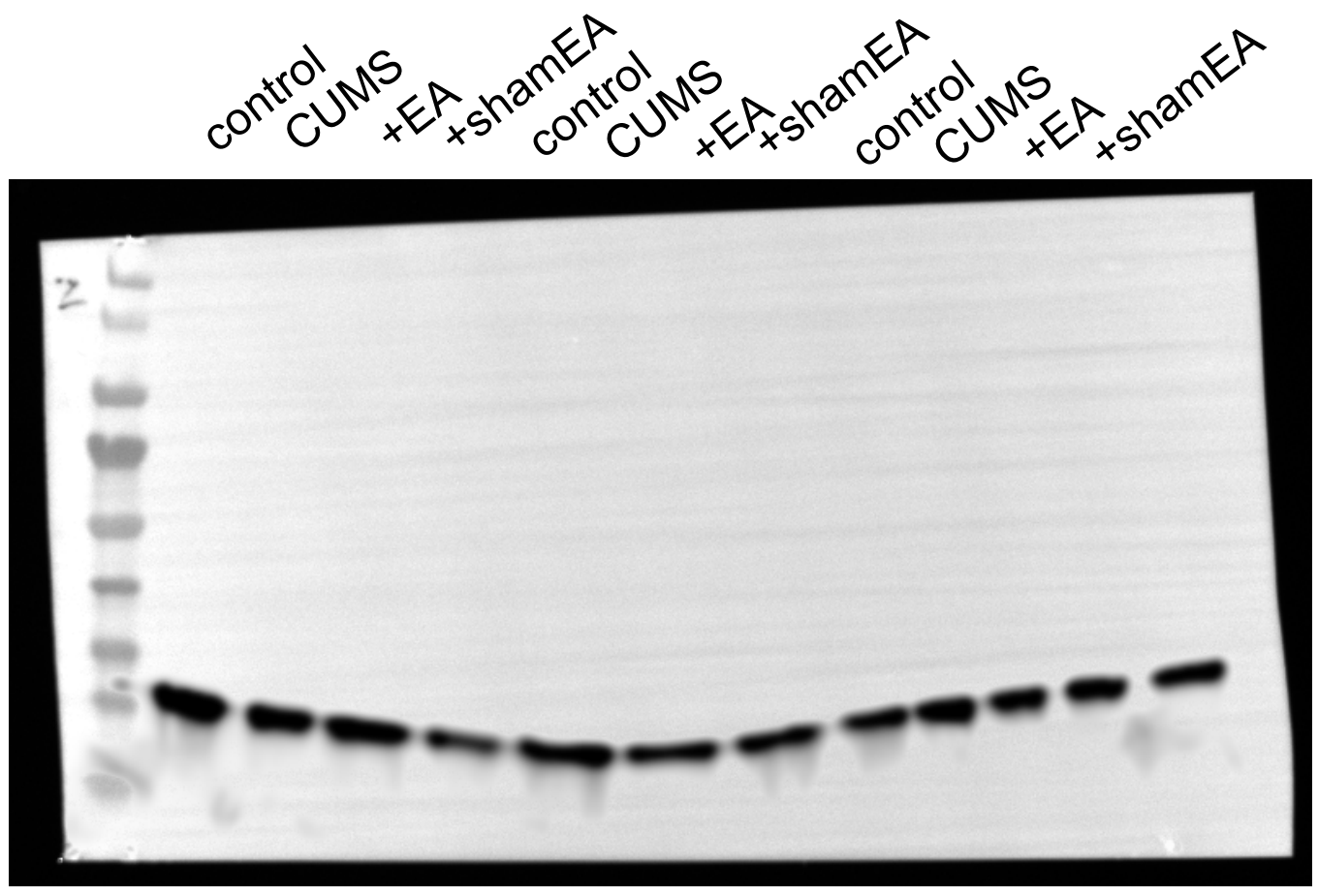

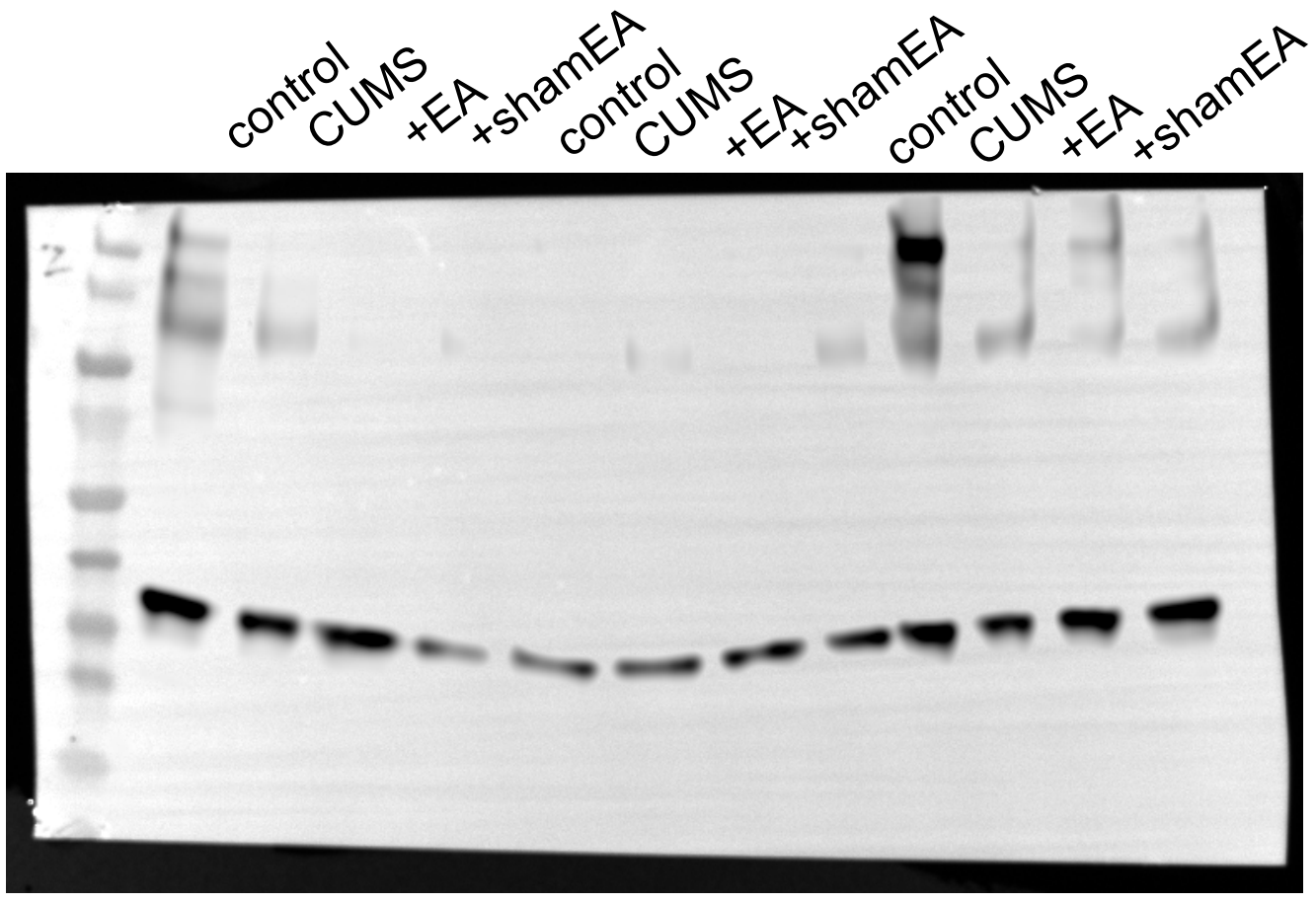

SNAP25

GAPDH

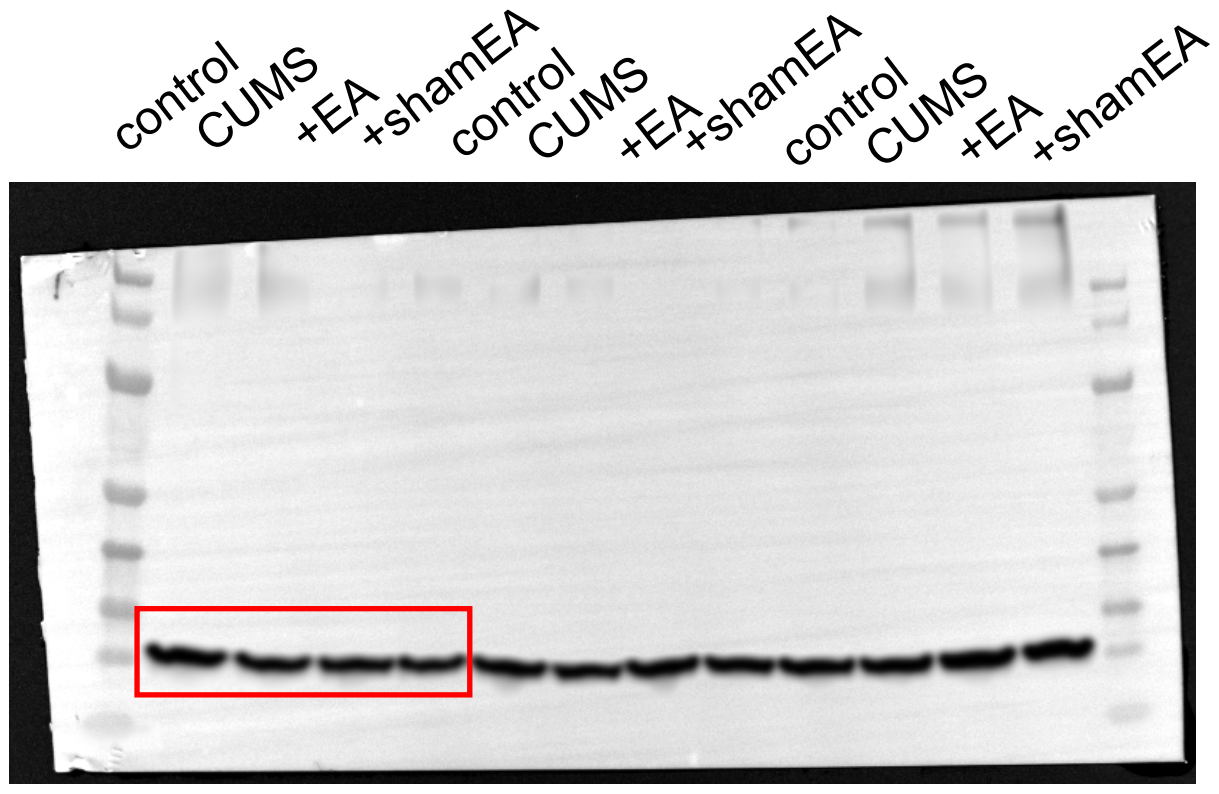

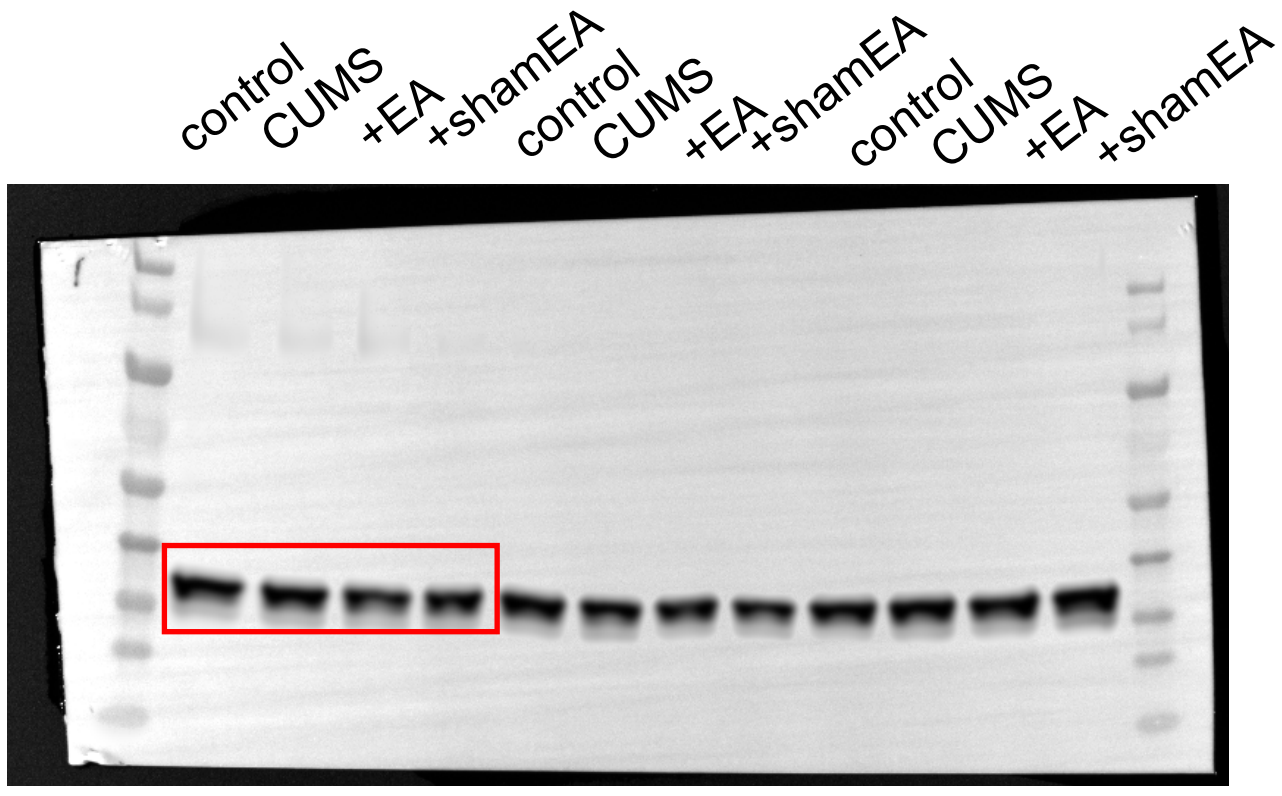

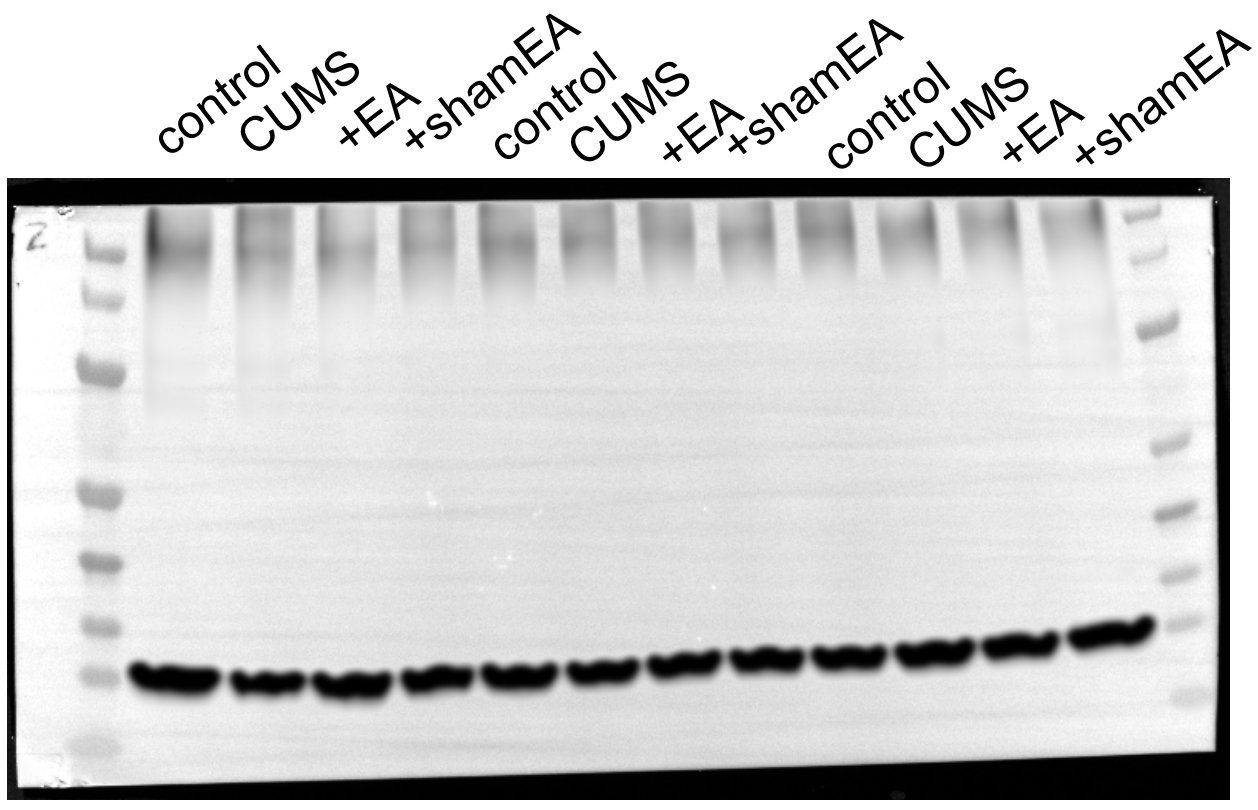

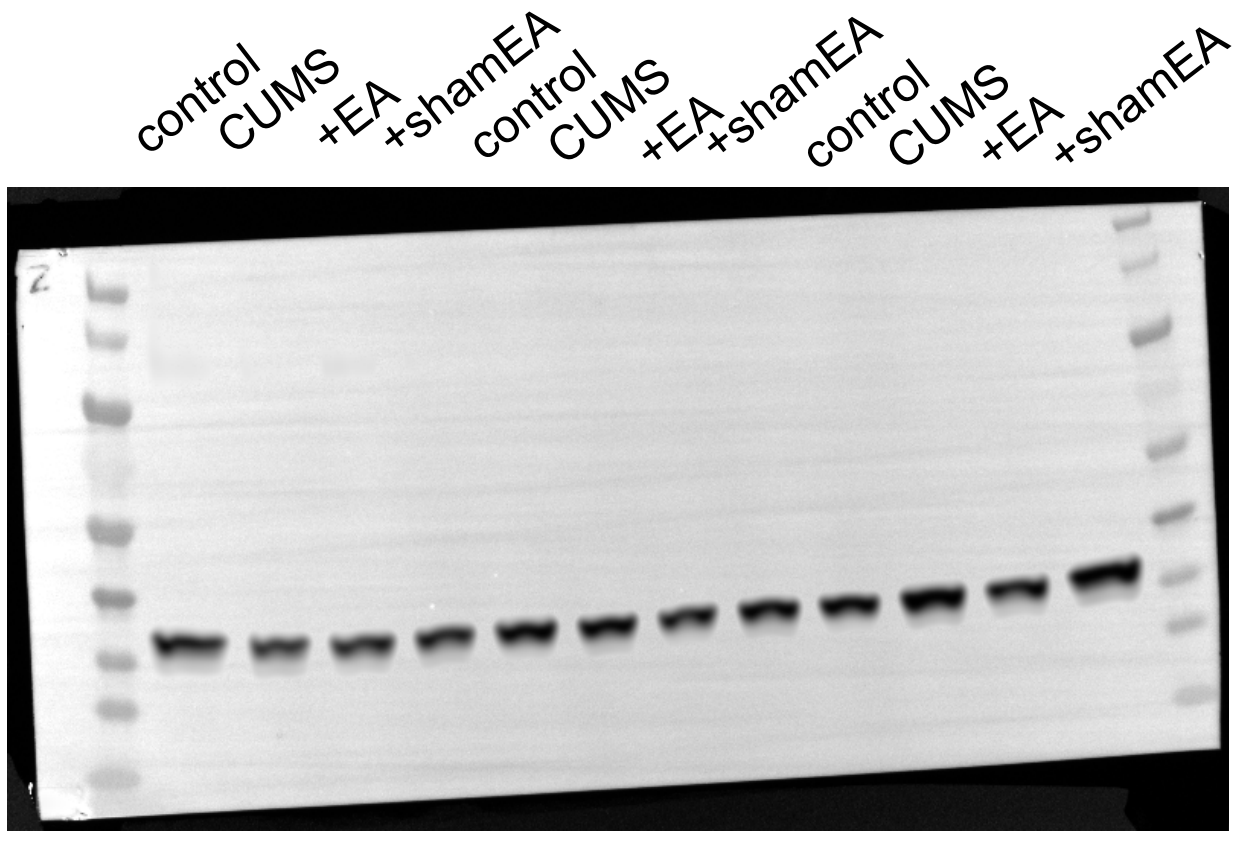

CALHM2

GAPDH

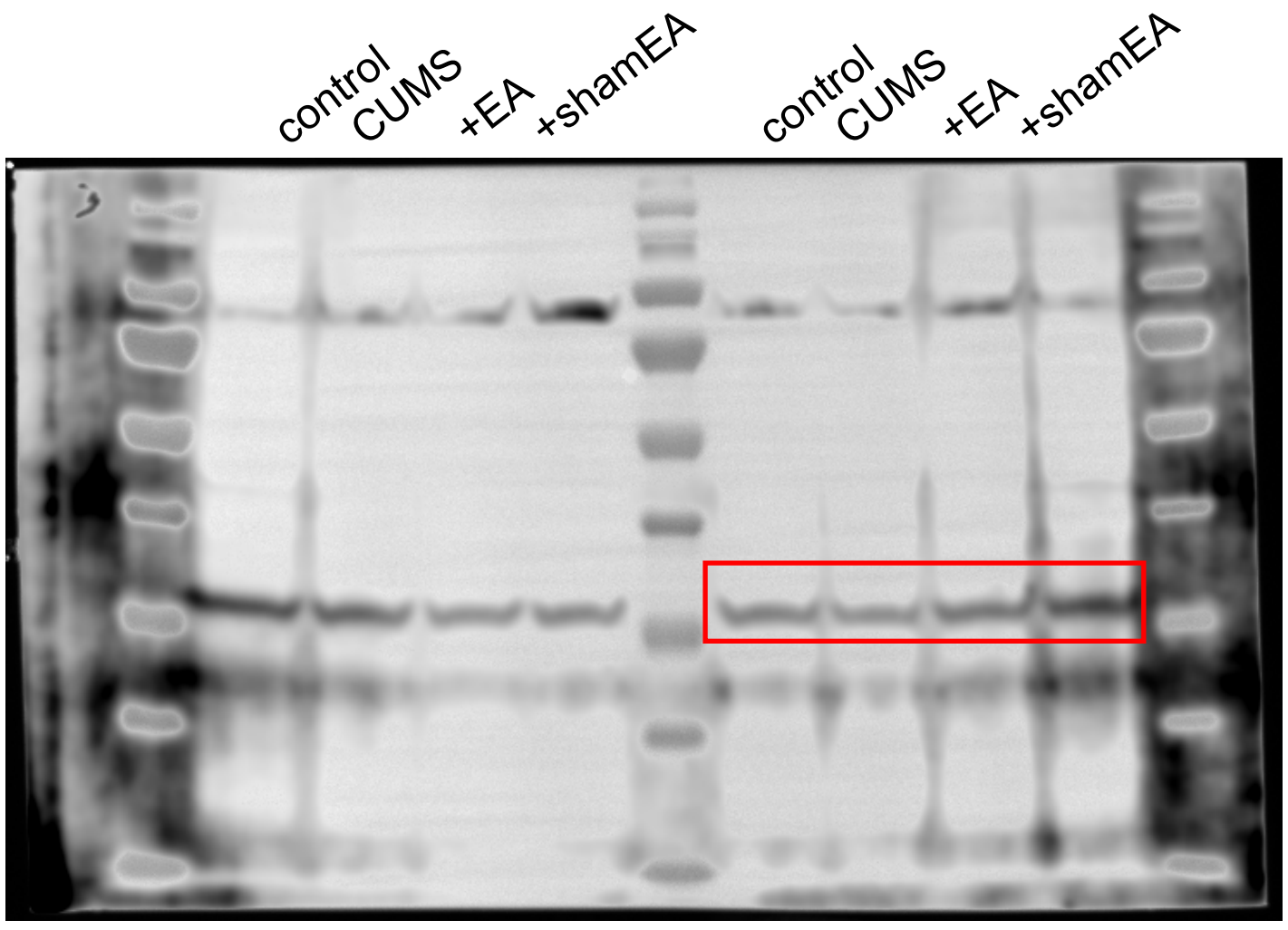

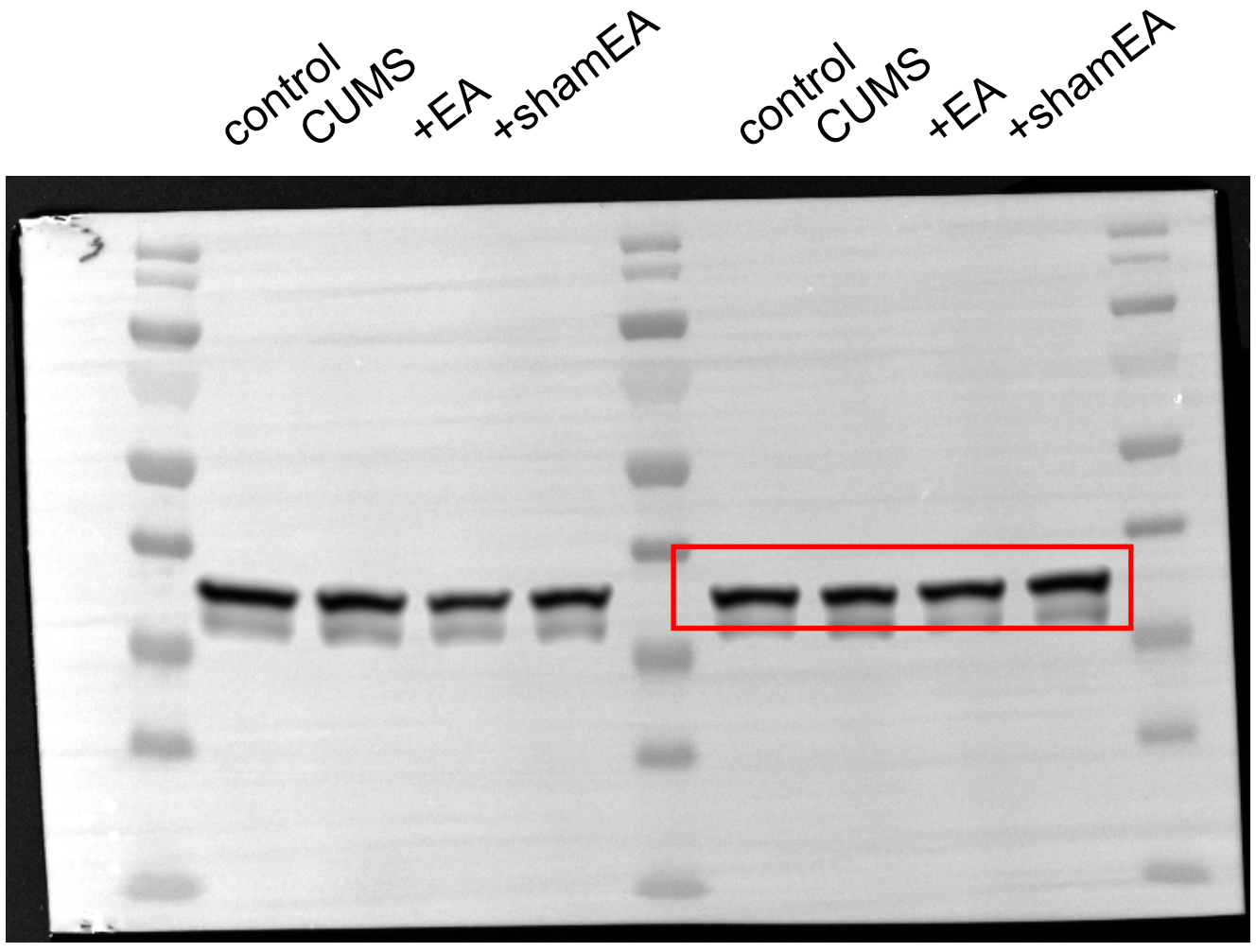

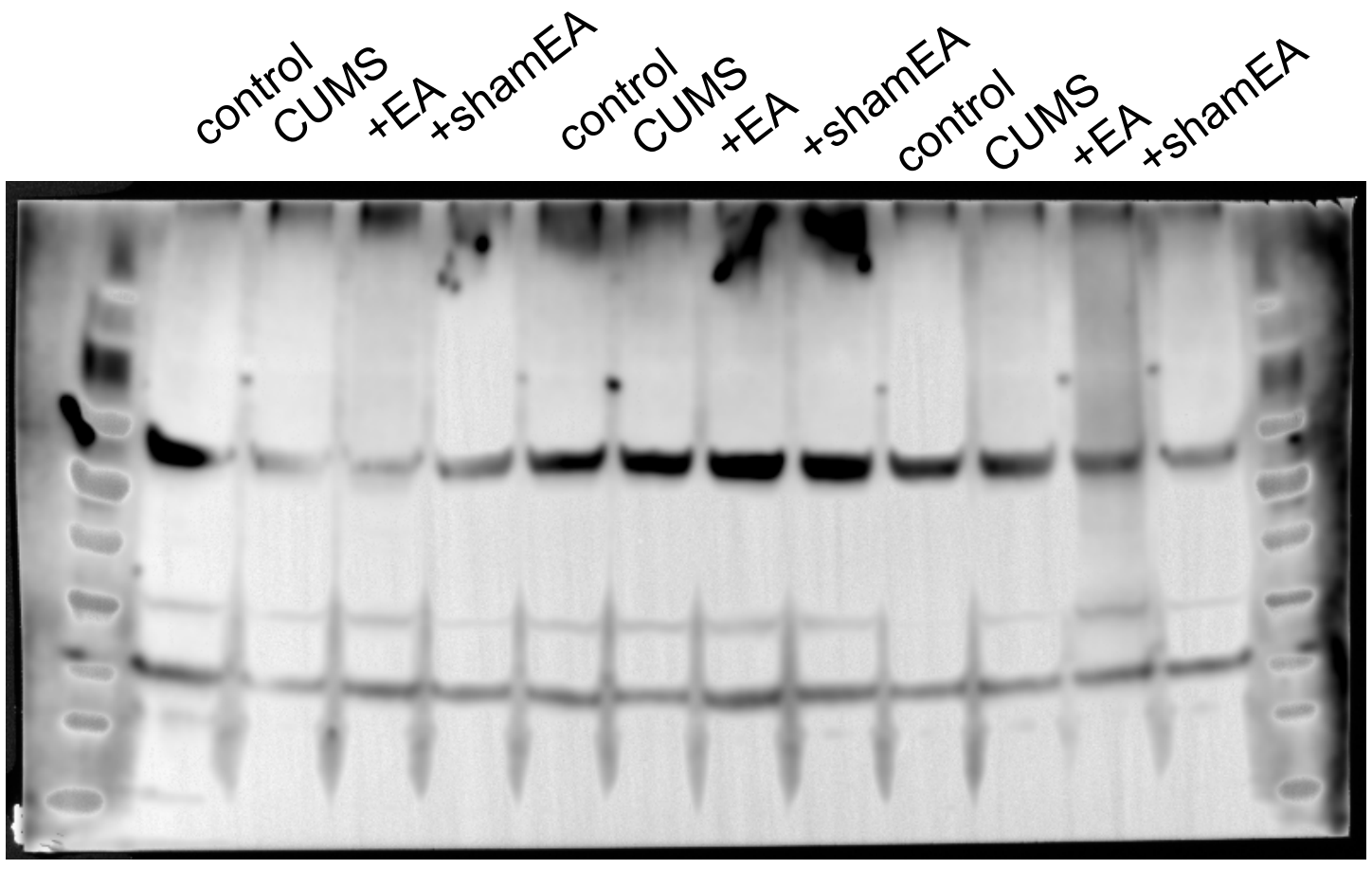

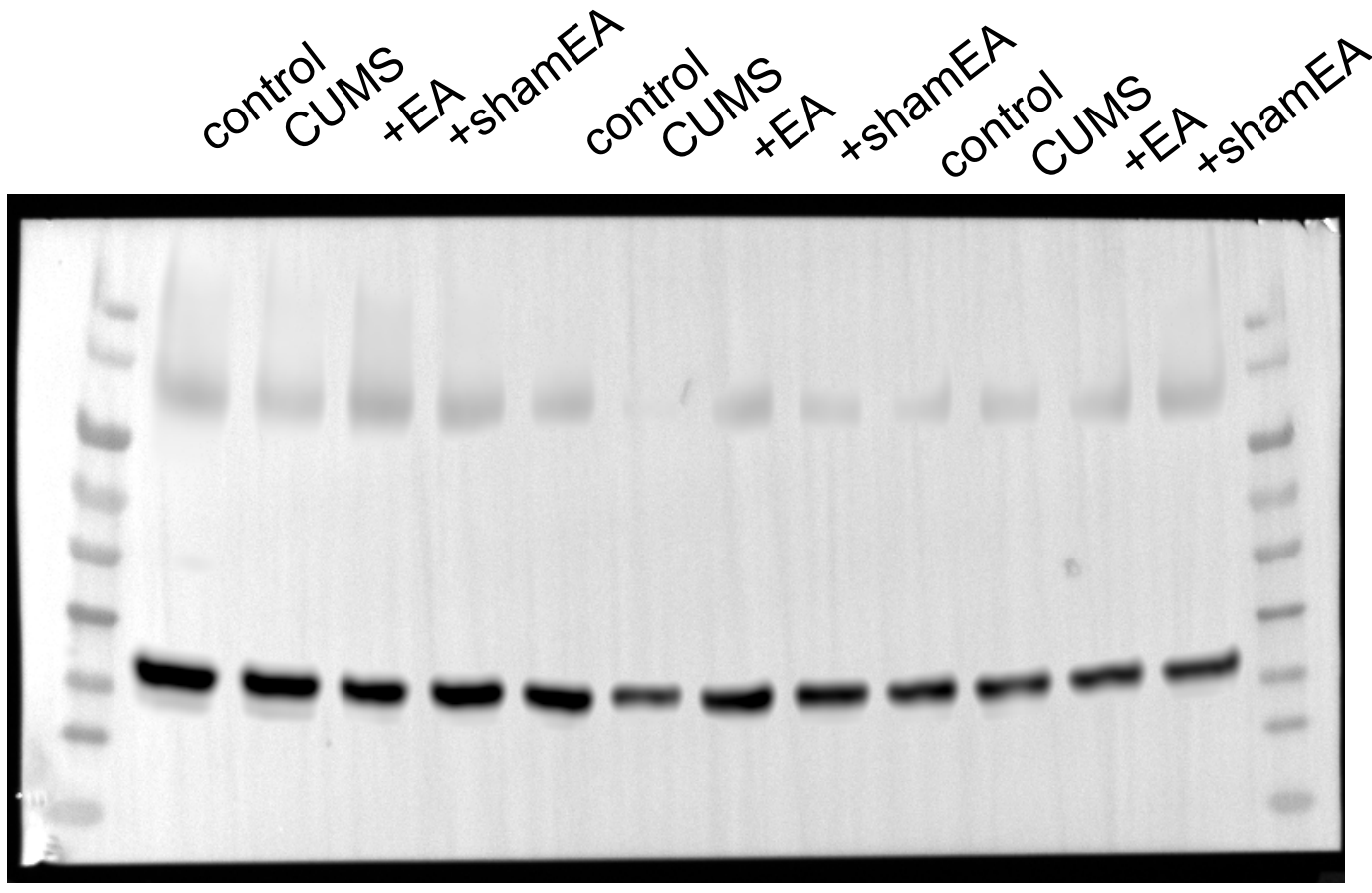

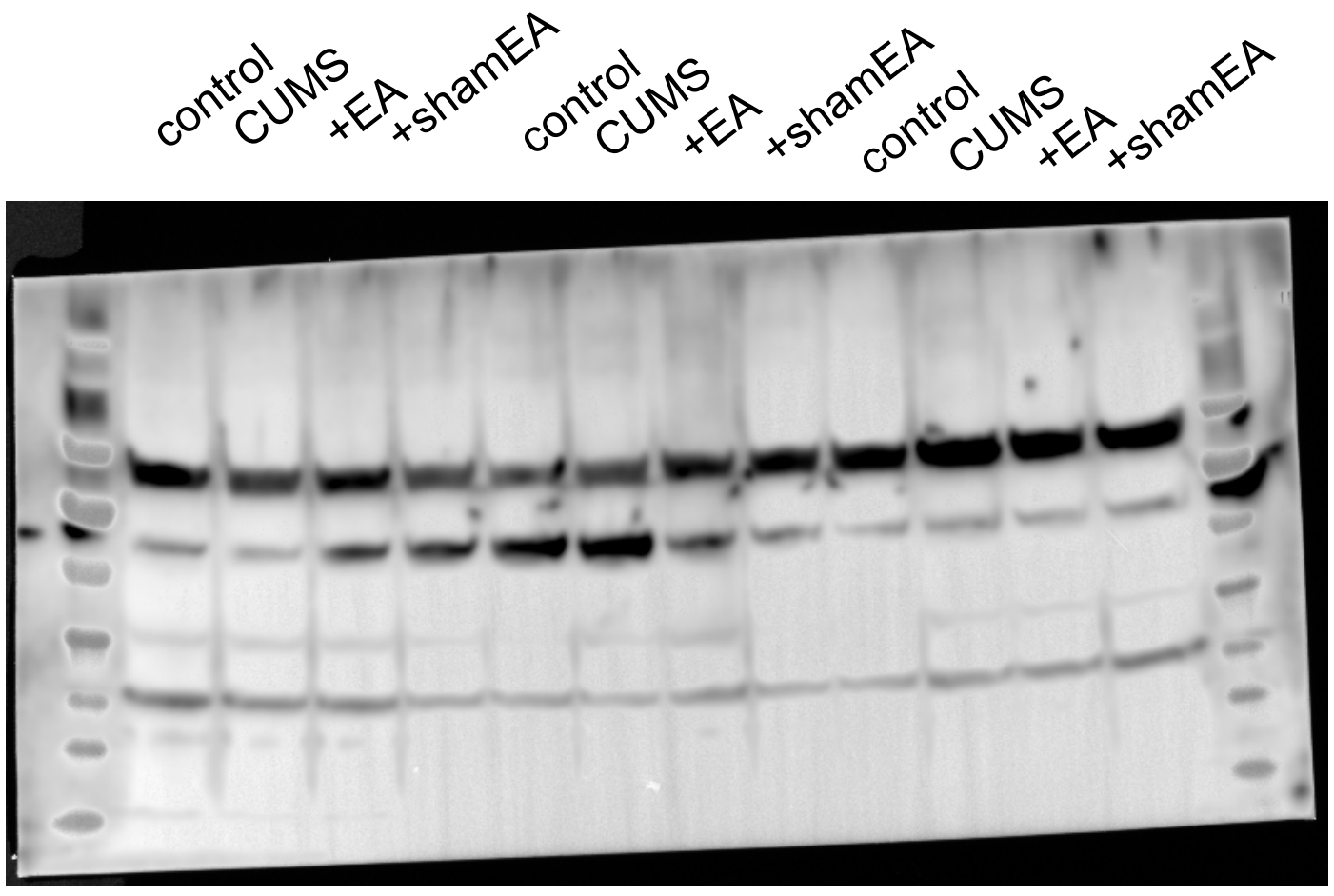

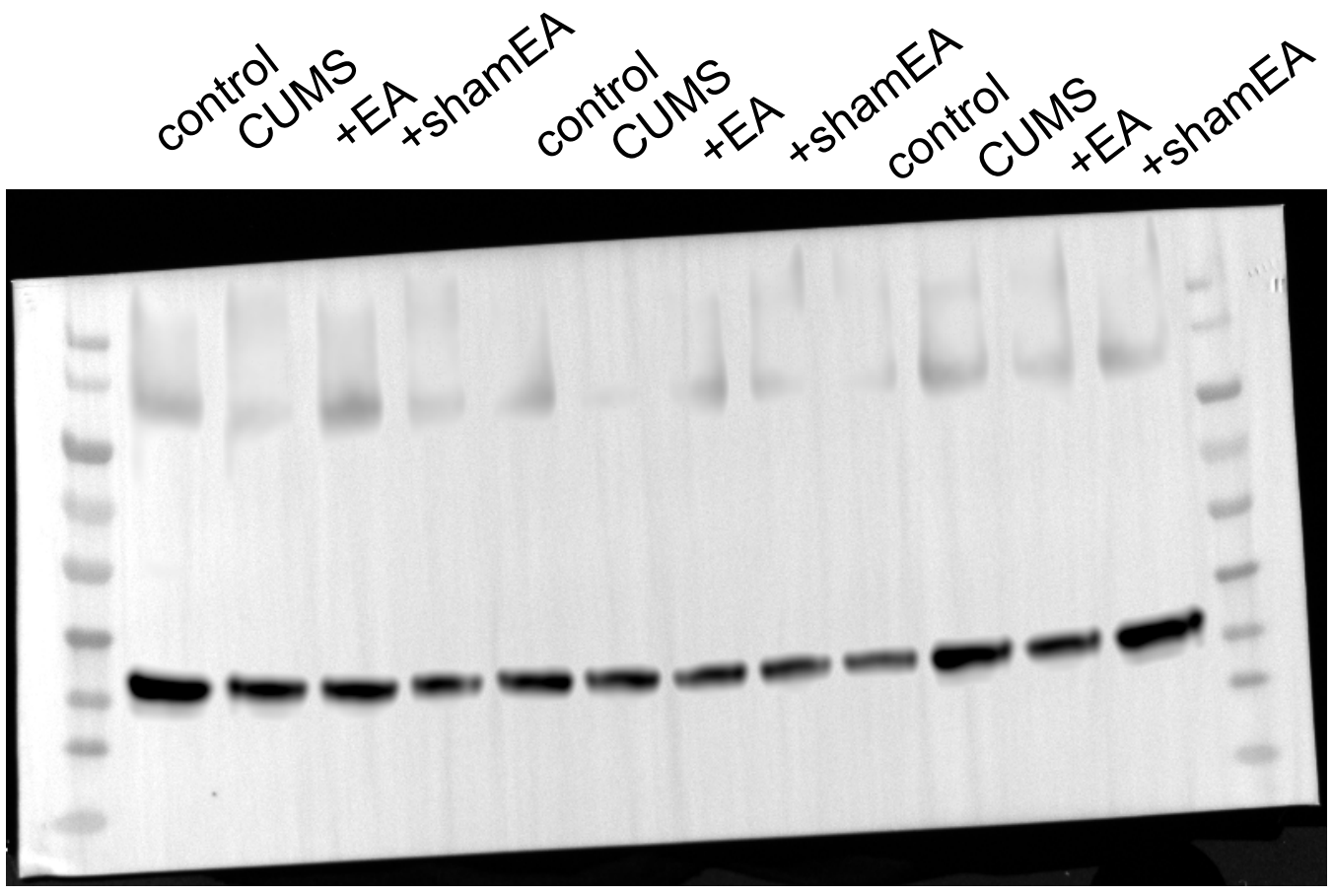

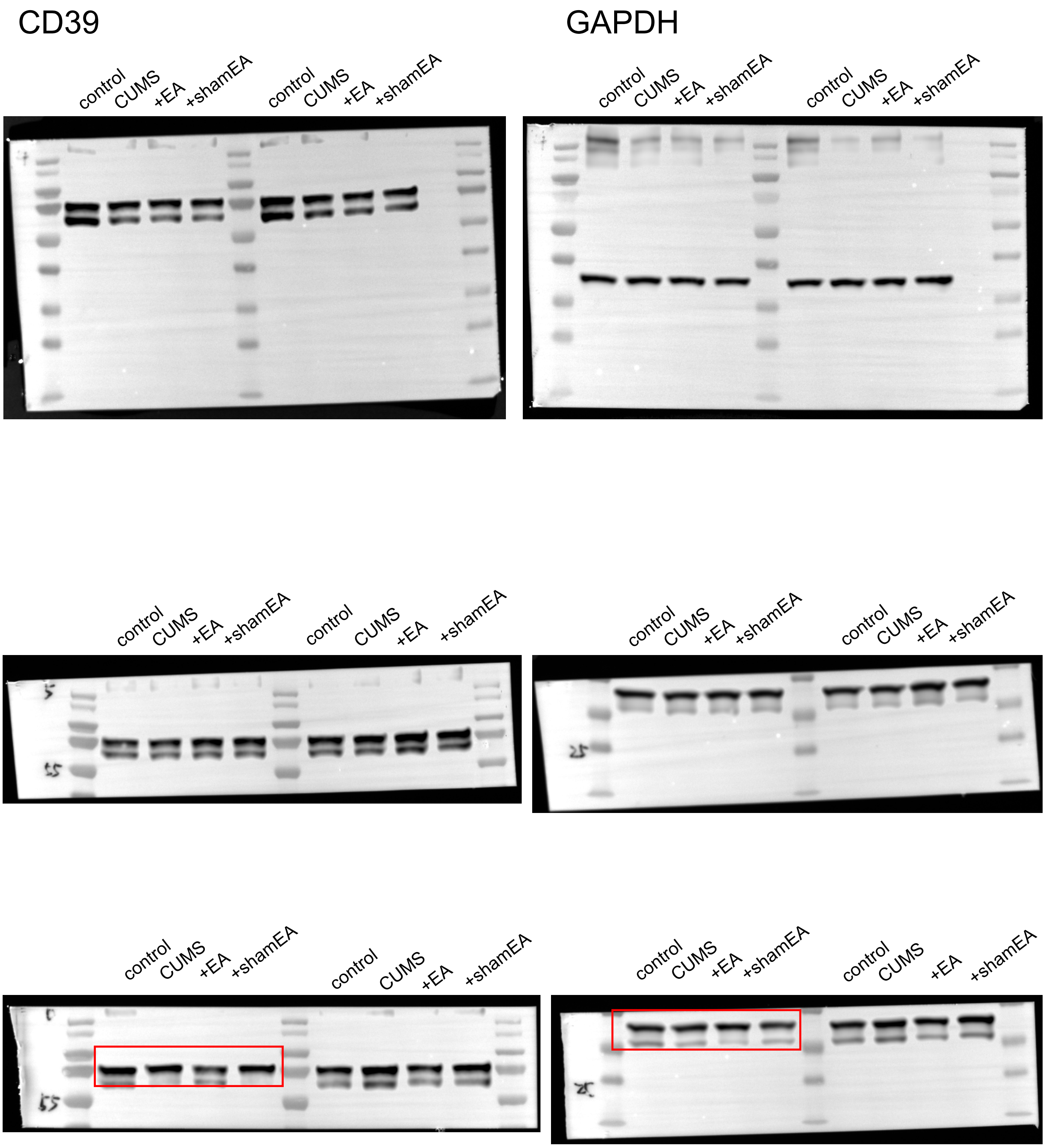

CD39
GAPDH
control CUMS +EA +shamEA control CUMS +EA +shamEA
control CUMS +EA +shamEA control CUMS +EA +shamEA
control CUMS +EA +shamEA control CUMS +EA +shamEA
control CUMS +EA +shamEA control CUMS +EA +shamEA
control CUMS +EA +shamEA control CUMS +EA +shamEA
control CUMS +EA +shamEA control CUMS +EA +shamEA

P2X7

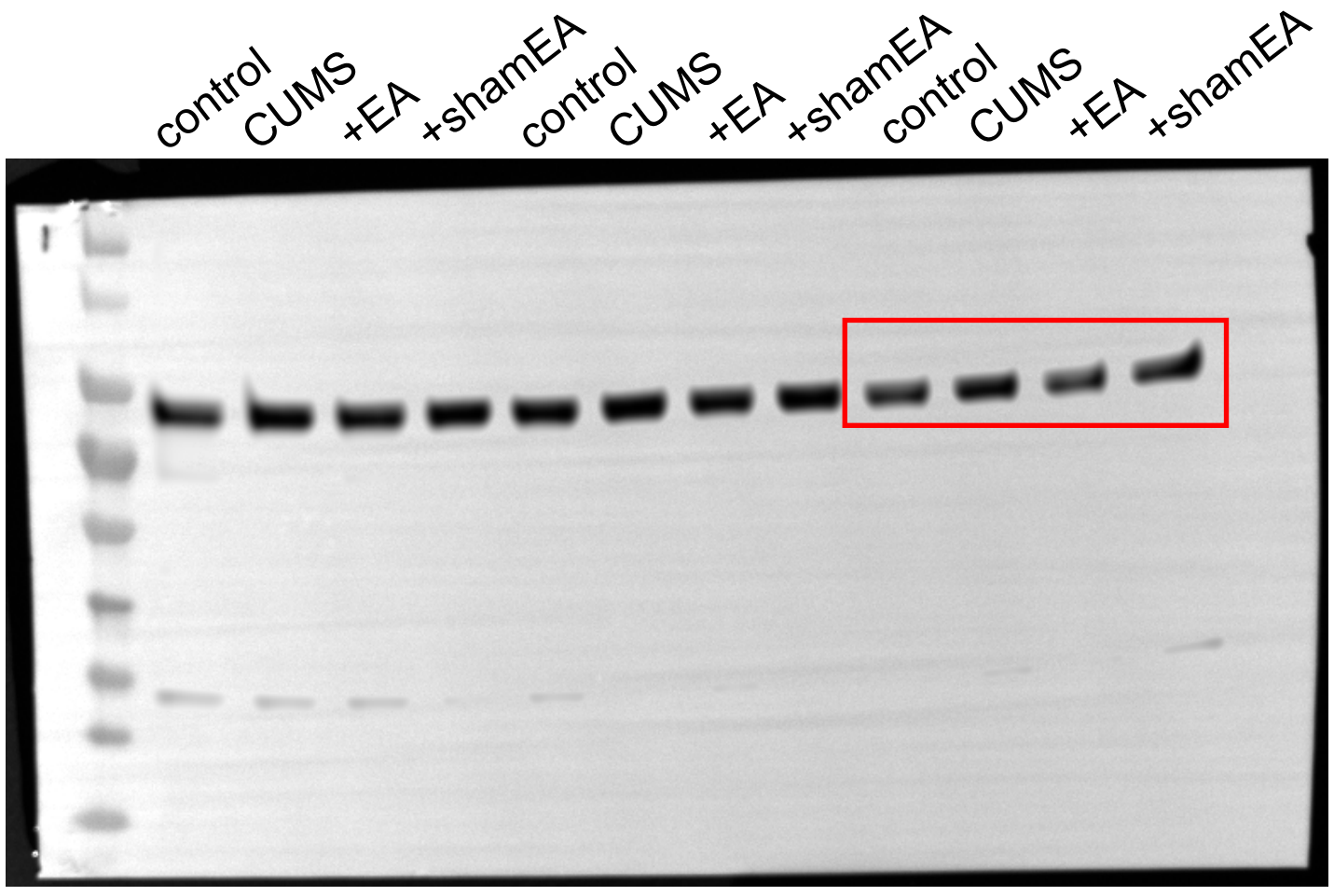
control
CUMS
+EA
+shamEA
control
CUMS
+EA
+shamEA
control
CUMS
+EA
+shamEA

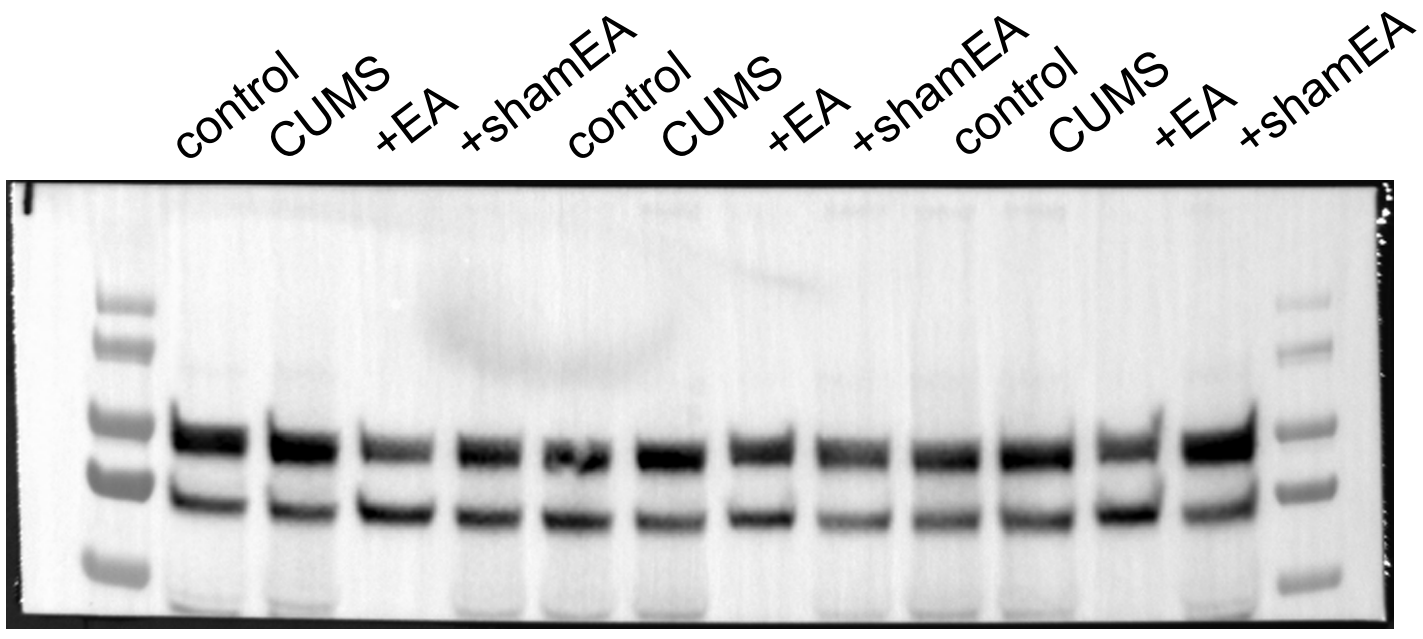
control
CUMS
+EA
+shamEA
control
CUMS
+EA
+shamEA
control
CUMS
+EA
+shamEA

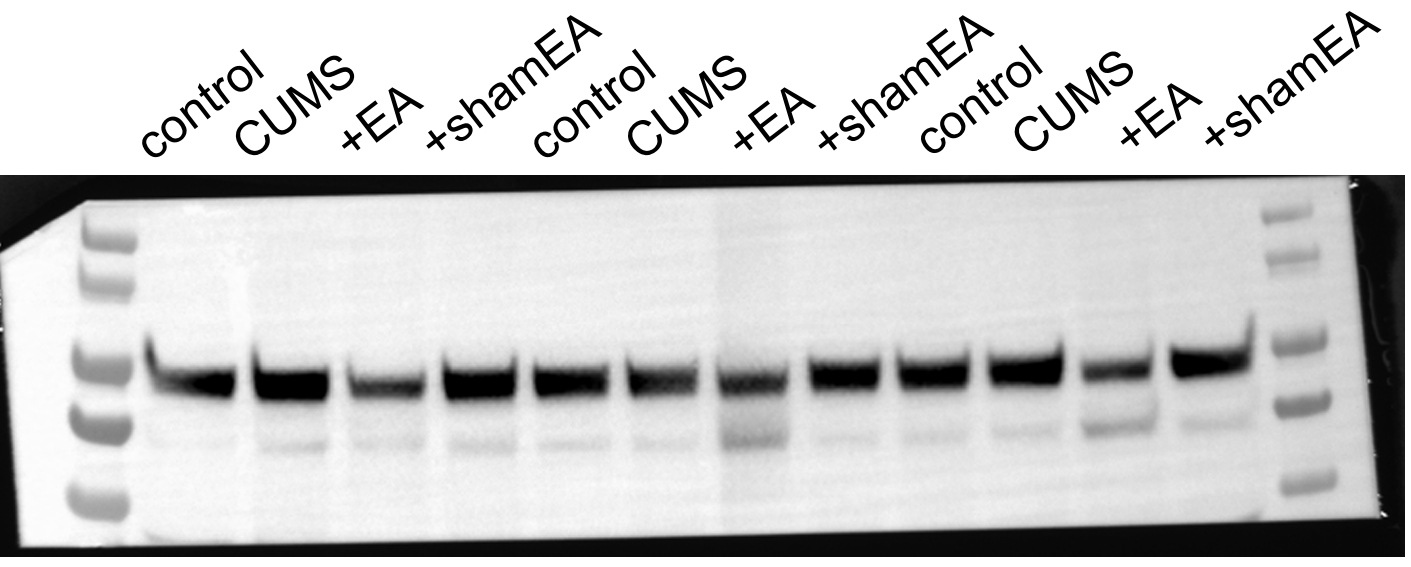
control
CUMS
+EA
+shamEA
control
CUMS
+EA
+shamEA
control
CUMS
+EA
+shamEA

GAPDH

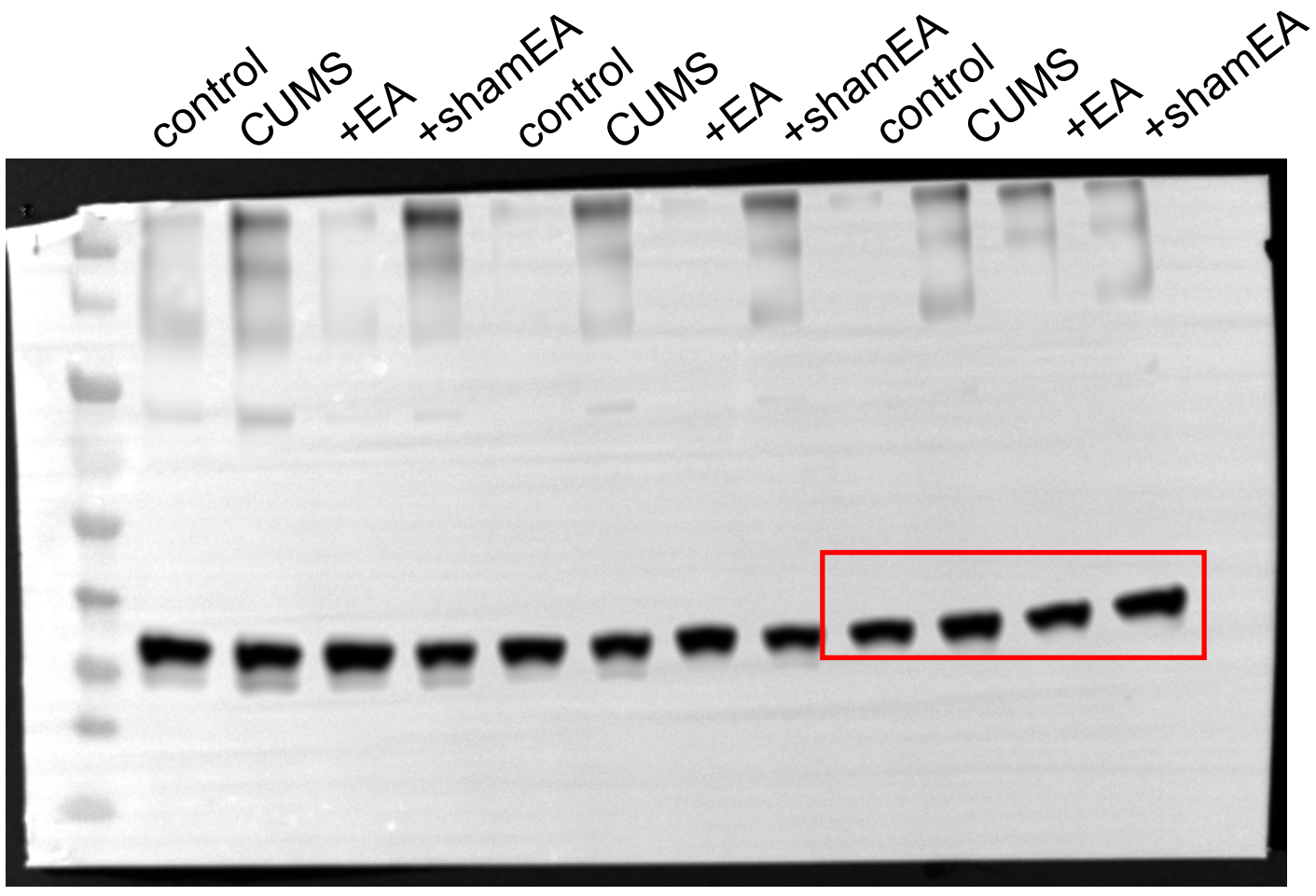
control
CUMS
+EA
+shamEA
control
CUMS
+EA
+shamEA
control
CUMS
+EA
+shamEA

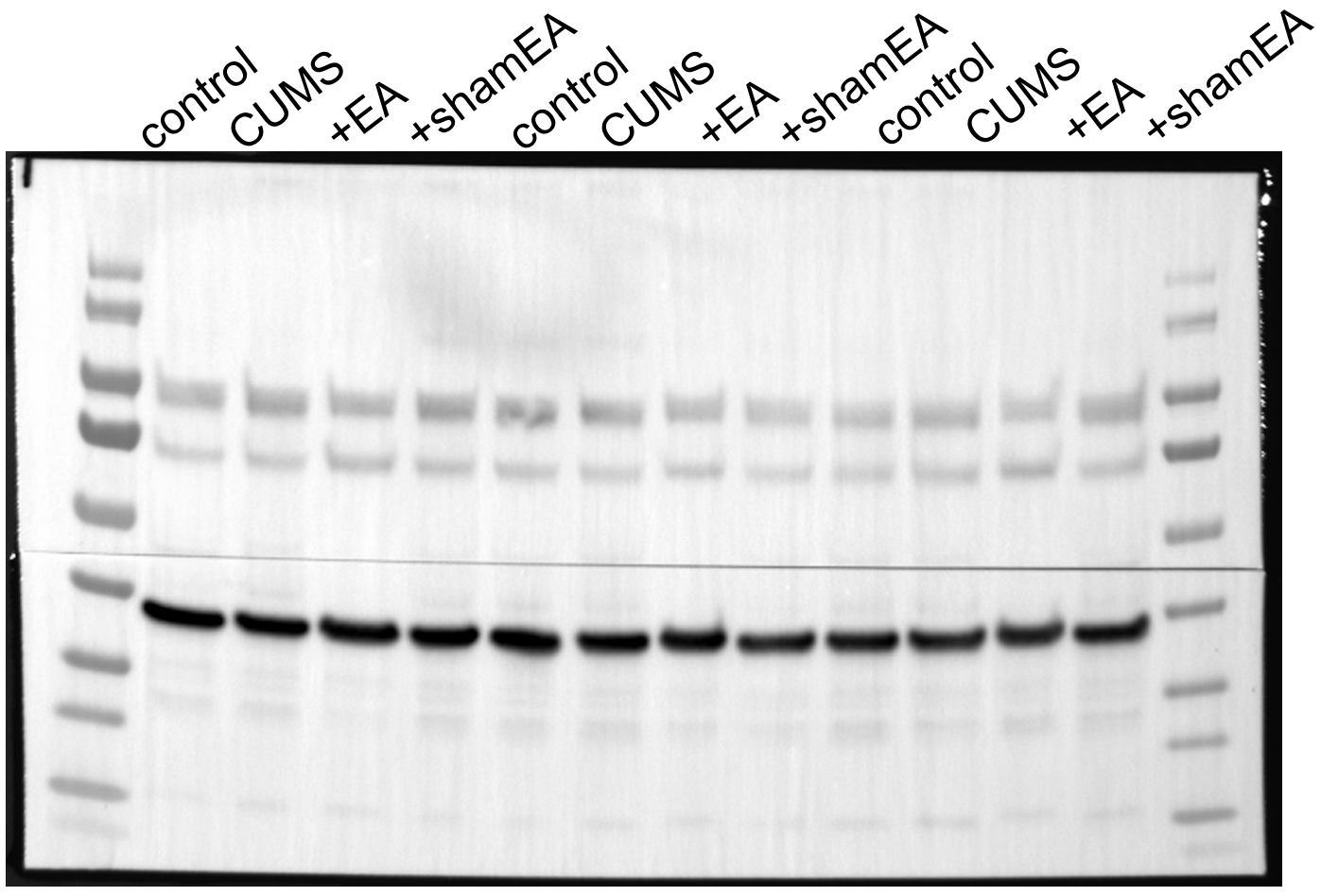
control
CUMS
+EA
+shamEA
control
CUMS
+EA
+shamEA
control
CUMS
+EA
+shamEA

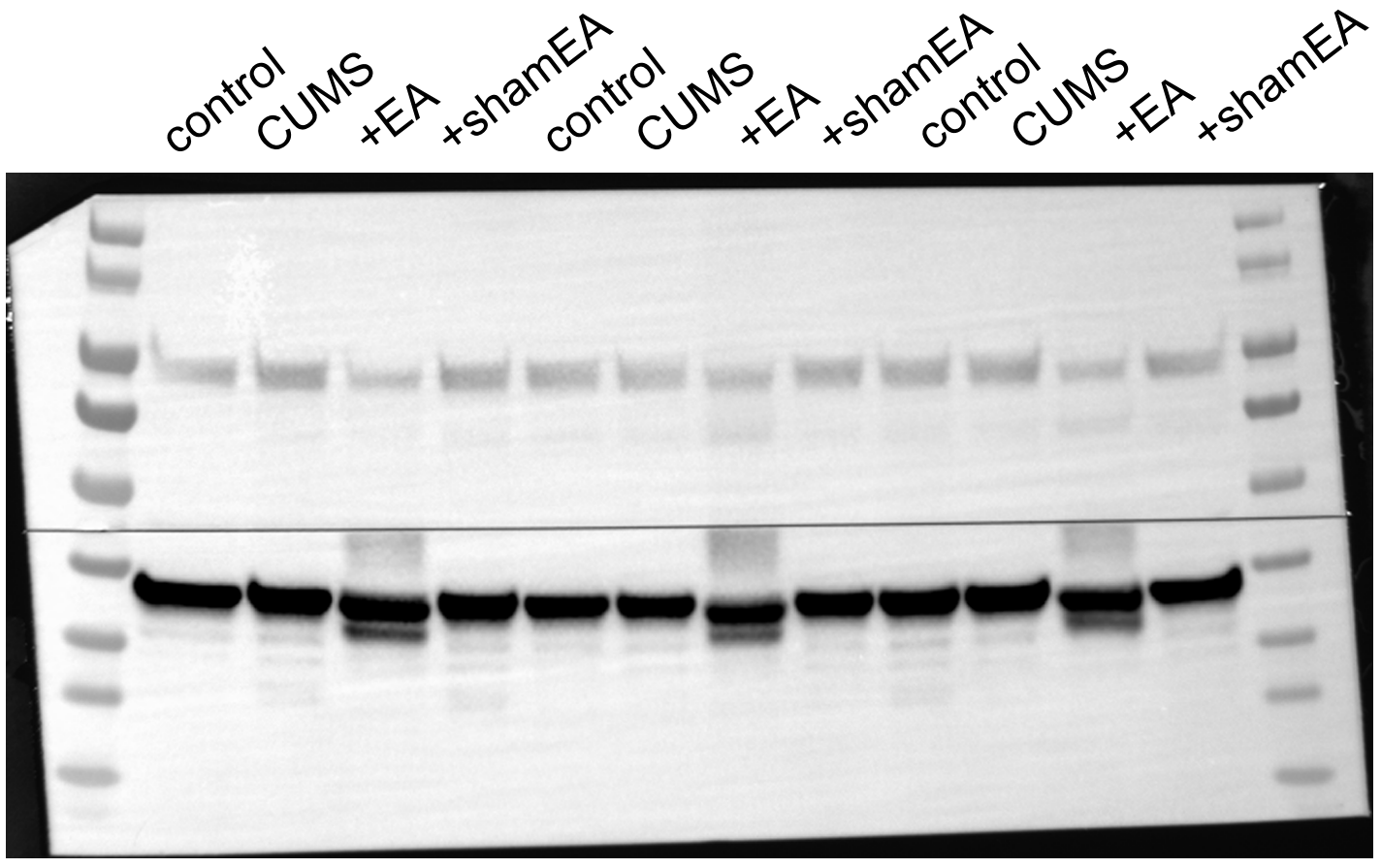
control
CUMS
+EA
+shamEA
control
CUMS
+EA
+shamEA
control
CUMS
+EA
+shamEA

P2X7

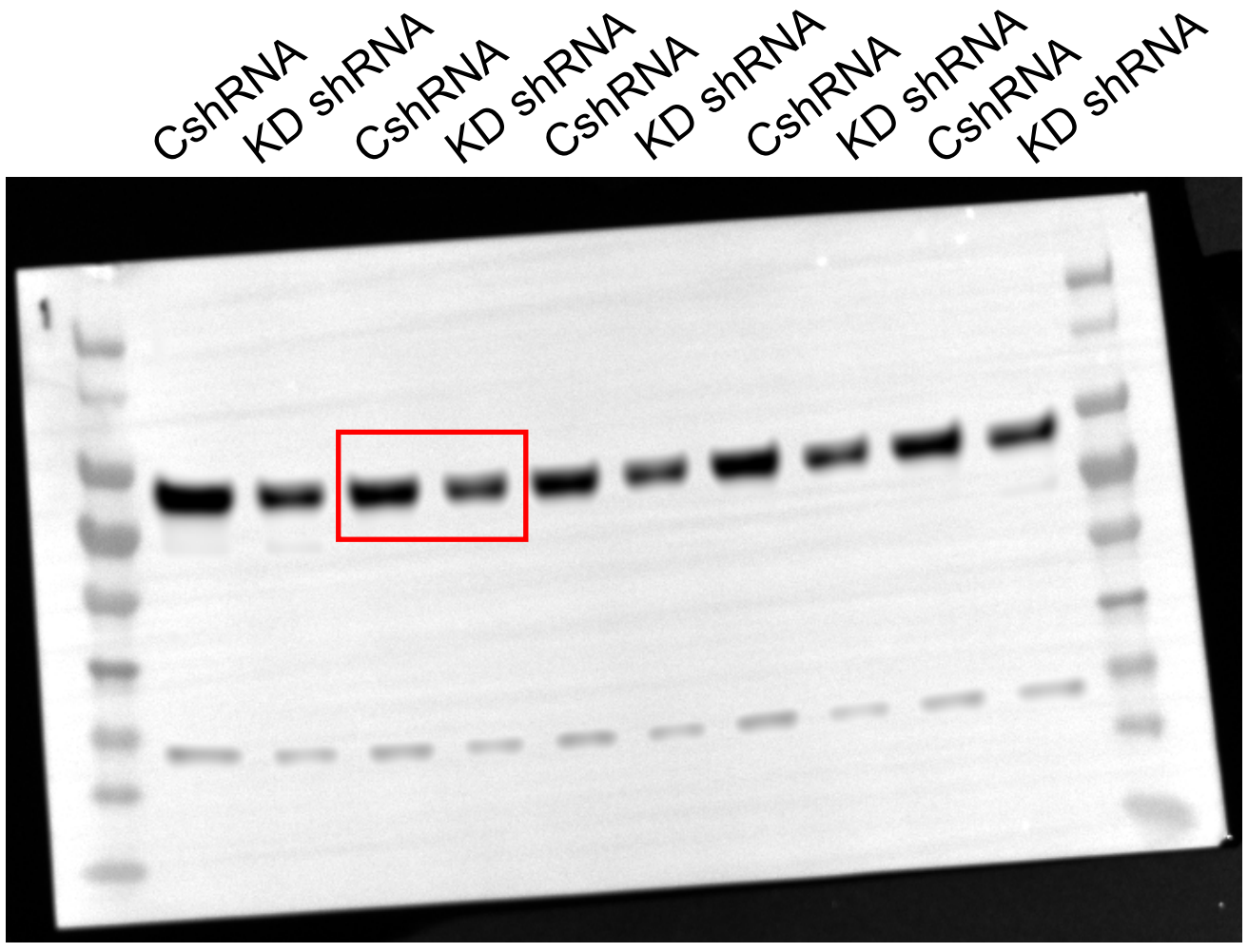
CshRNA
KD shRNA
CshRNA
KD shRNA
CshRNA
KD shRNA
CshRNA
KD shRNA
CshRNA
KD shRNA

GAPDH

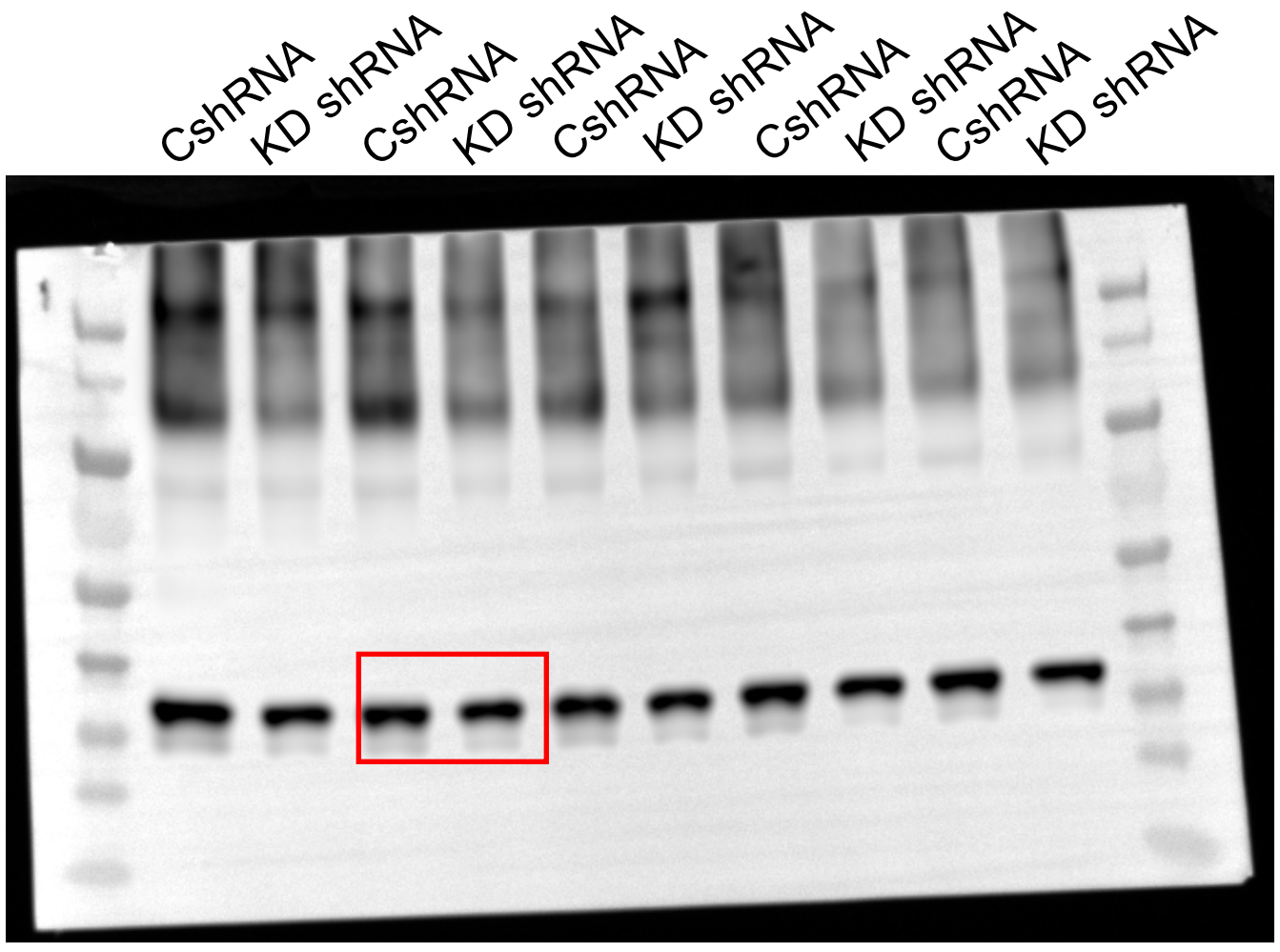
CshRNA
KD shRNA
CshRNA
KD shRNA
CshRNA
KD shRNA
CshRNA
KD shRNA
CshRNA
KD shRNA

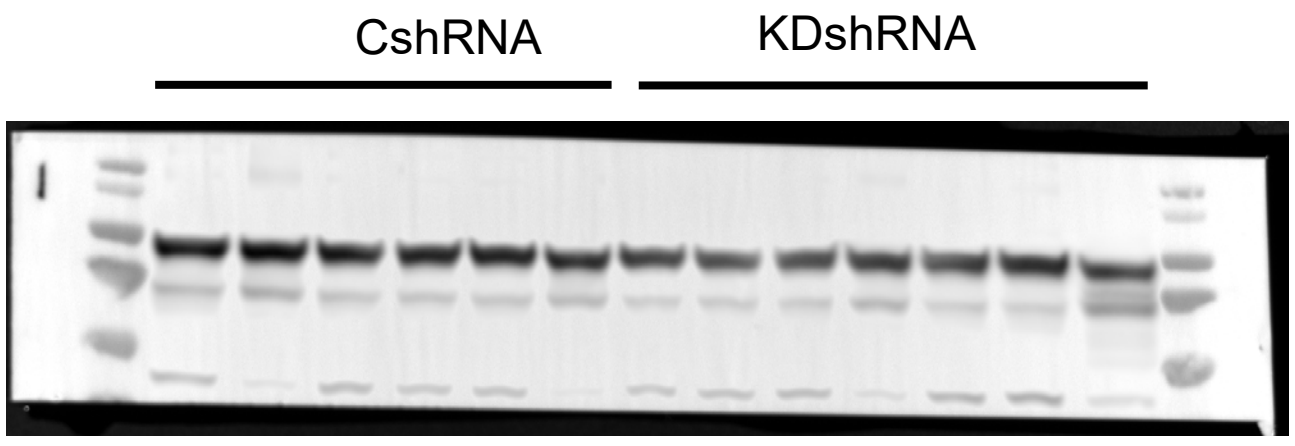
CshRNA
KDshRNA

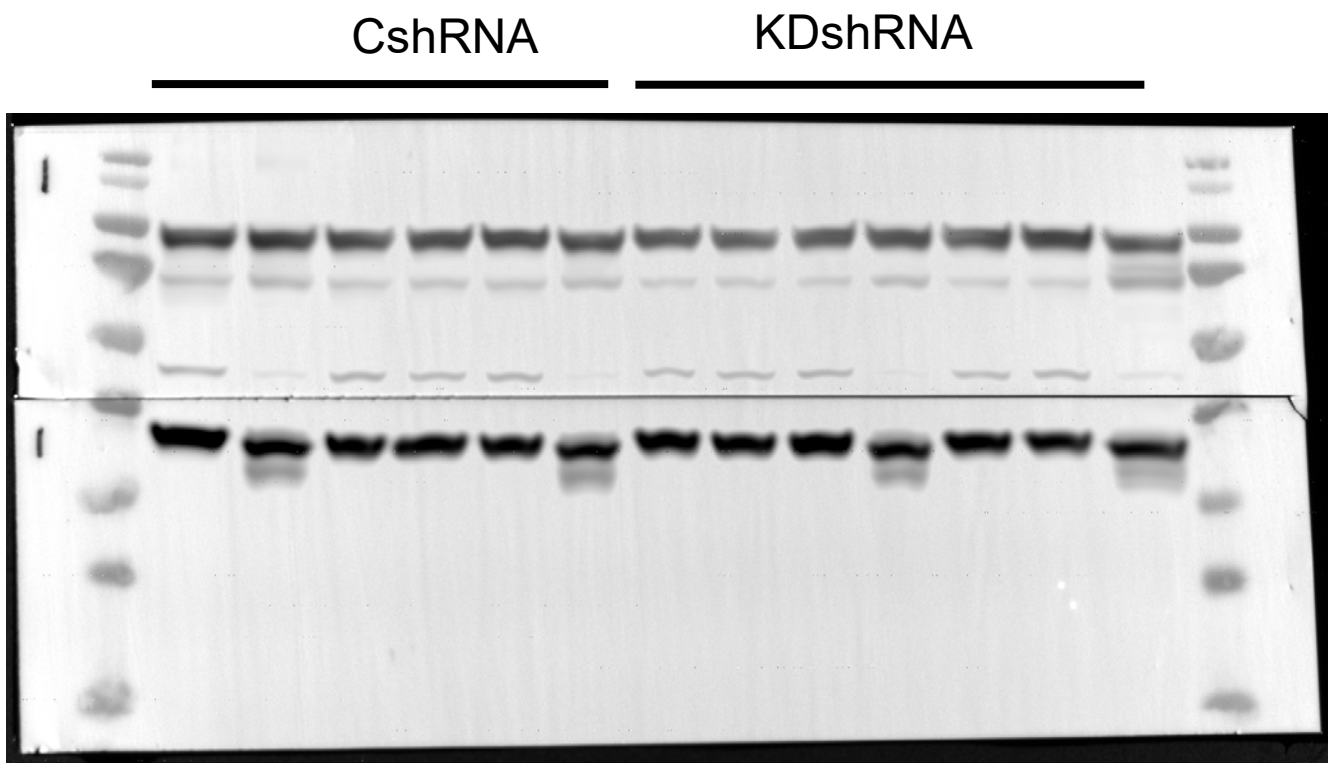
CshRNA
KDshRNA

P2X7 GAPDH

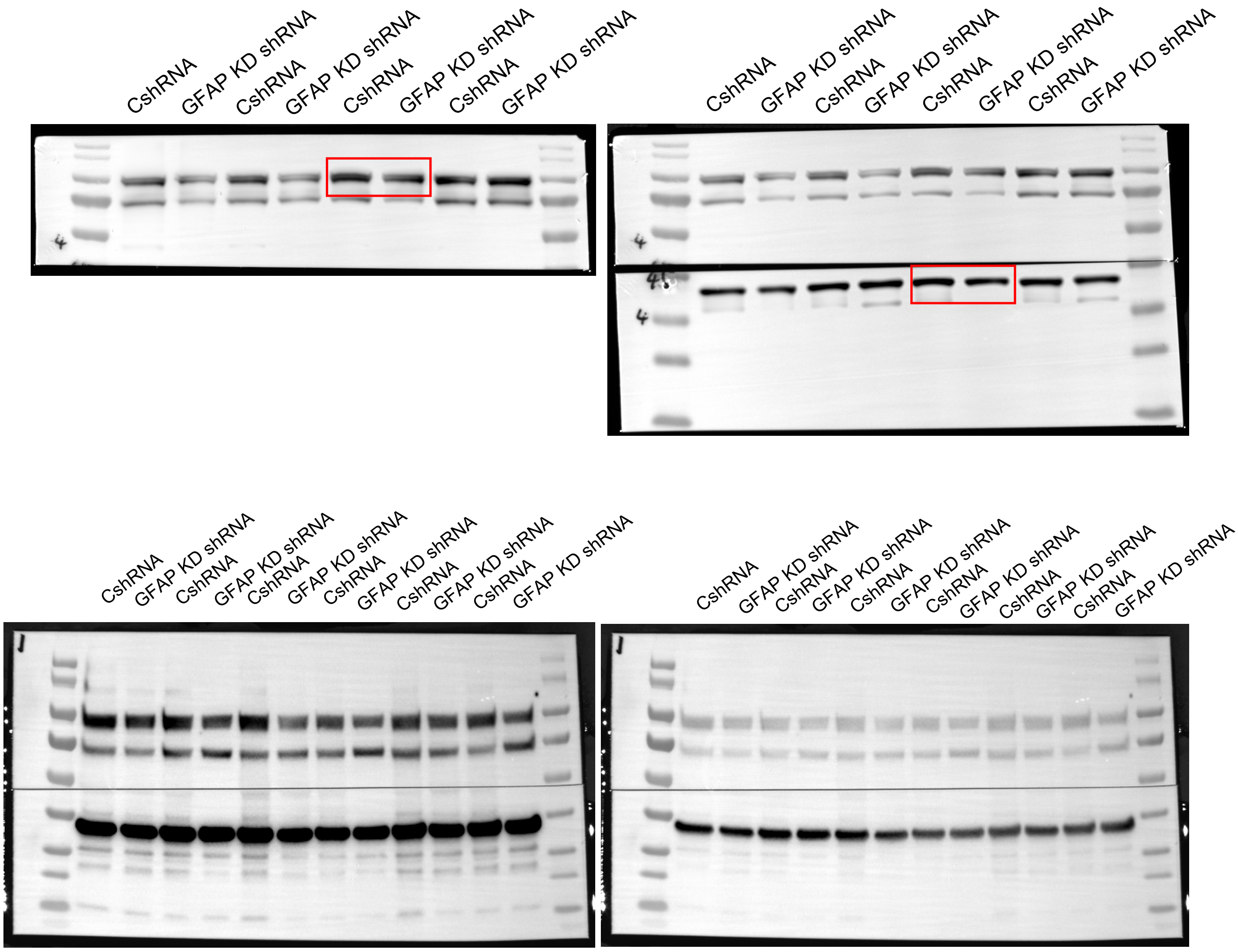

CshRNA
GFAP KD shRNA
CshRNA
GFAP KD shRNA
CshRNA
GFAP KD shRNA
CshRNA
GFAP KD shRNA

P2X7

GAPDH

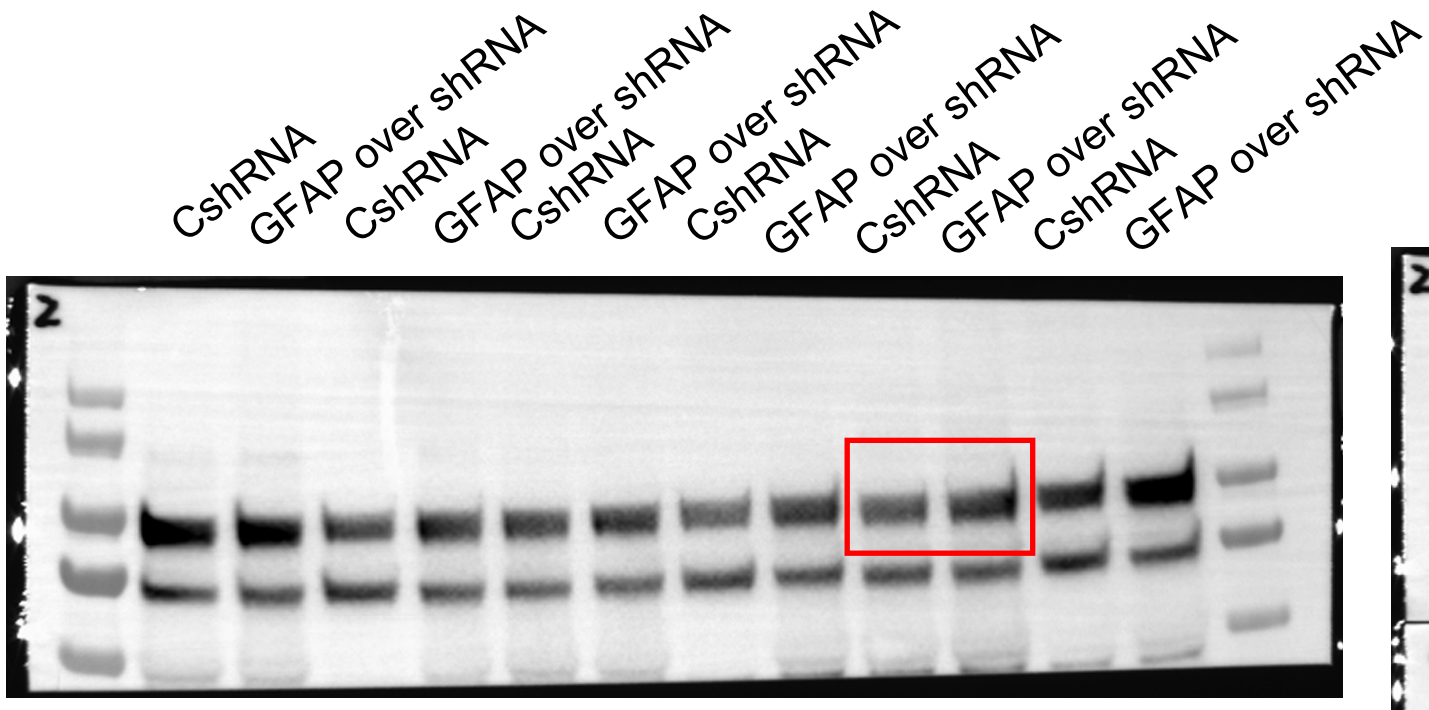
CshRNA
GFAP over shRNA
CshRNA
GFAP over shRNA
CshRNA
GFAP over shRNA
CshRNA
GFAP over shRNA
CshRNA
GFAP over shRNA
CshRNA
GFAP over shRNA

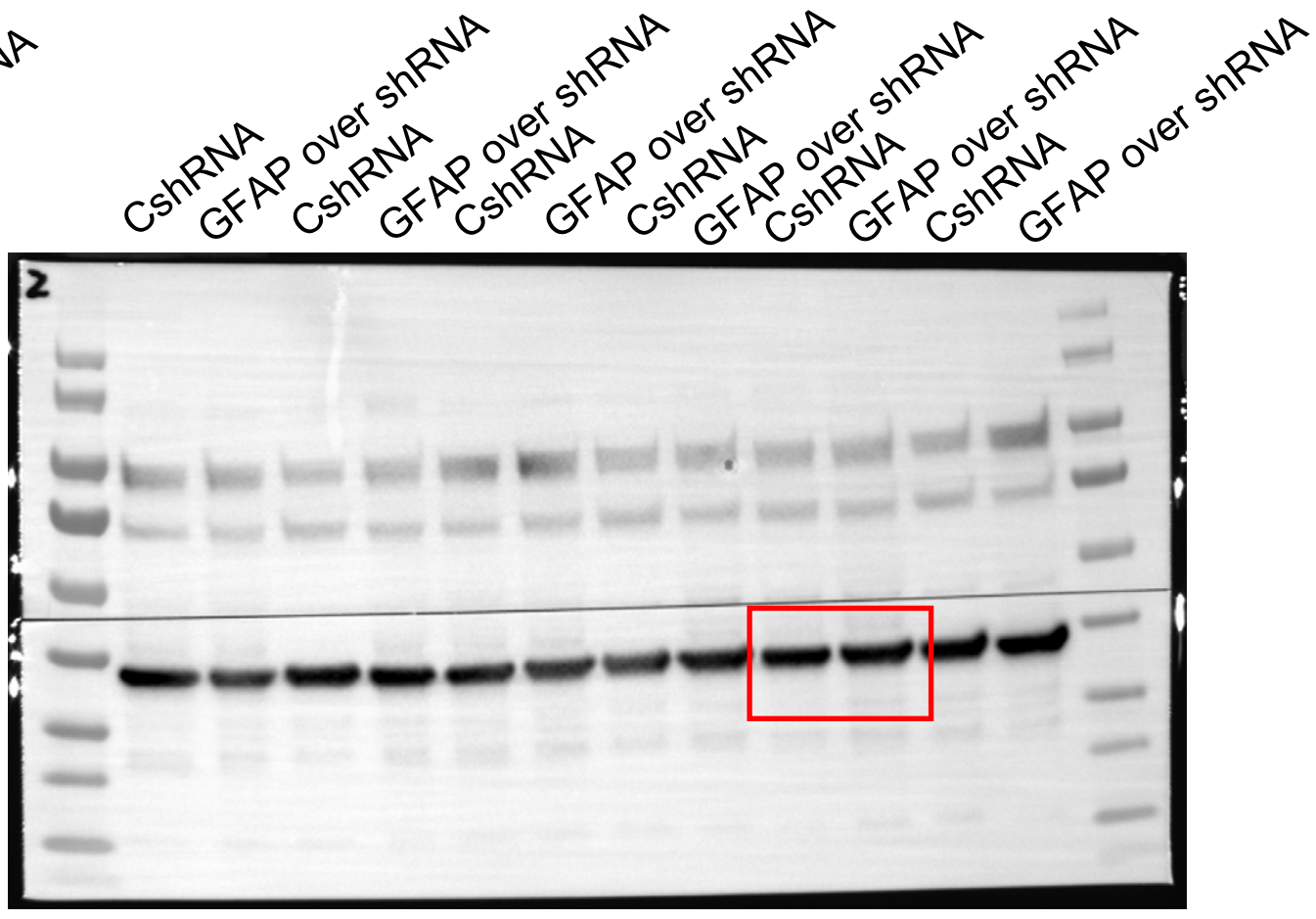
CshRNA
GFAP over shRNA
CshRNA
GFAP over shRNA
CshRNA
GFAP over shRNA
CshRNA
GFAP over shRNA
CshRNA
GFAP over shRNA
CshRNA
GFAP over shRNA

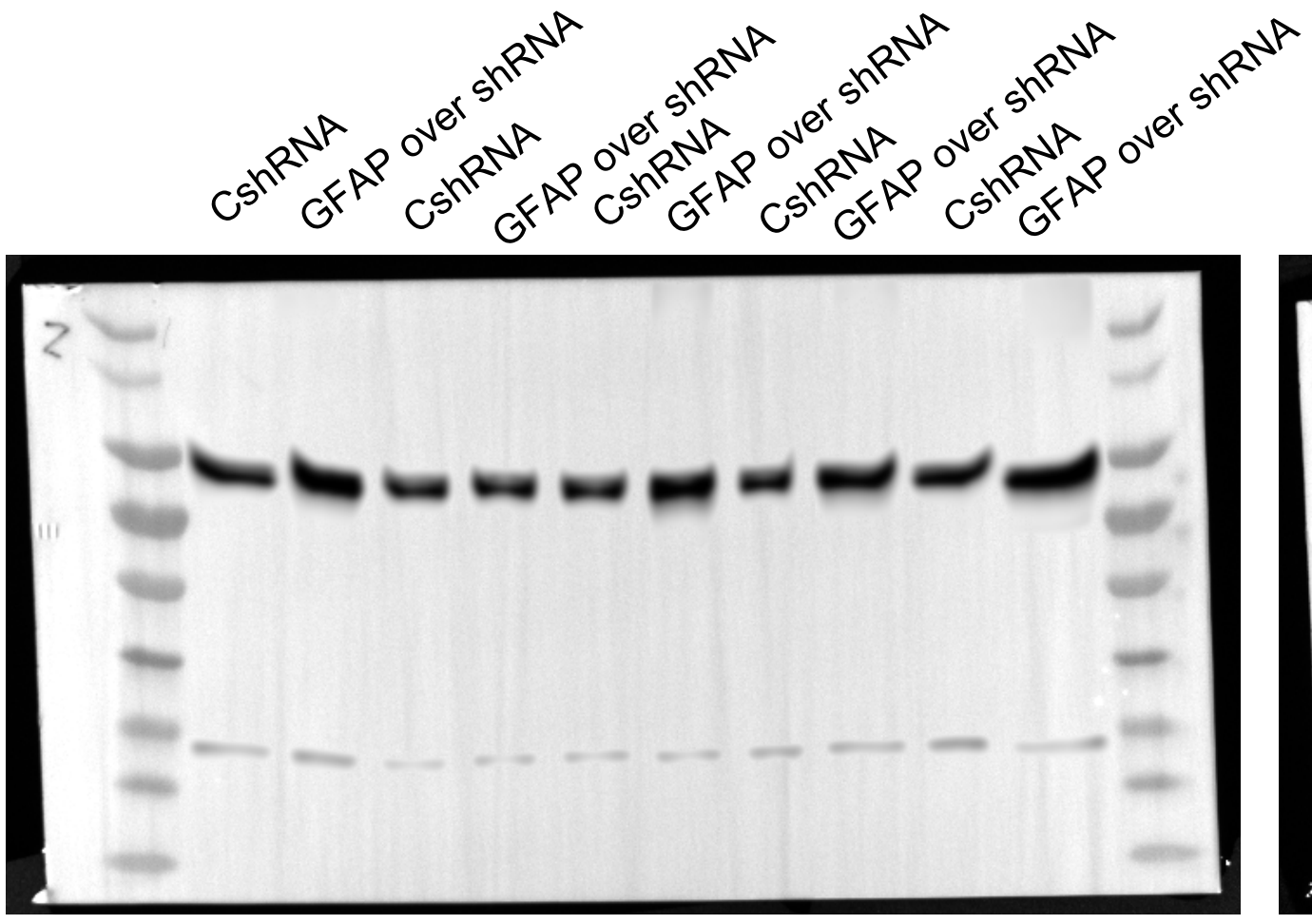
CshRNA
GFAP over shRNA
CshRNA
GFAP over shRNA
CshRNA
GFAP over shRNA
CshRNA
GFAP over shRNA
CshRNA
GFAP over shRNA

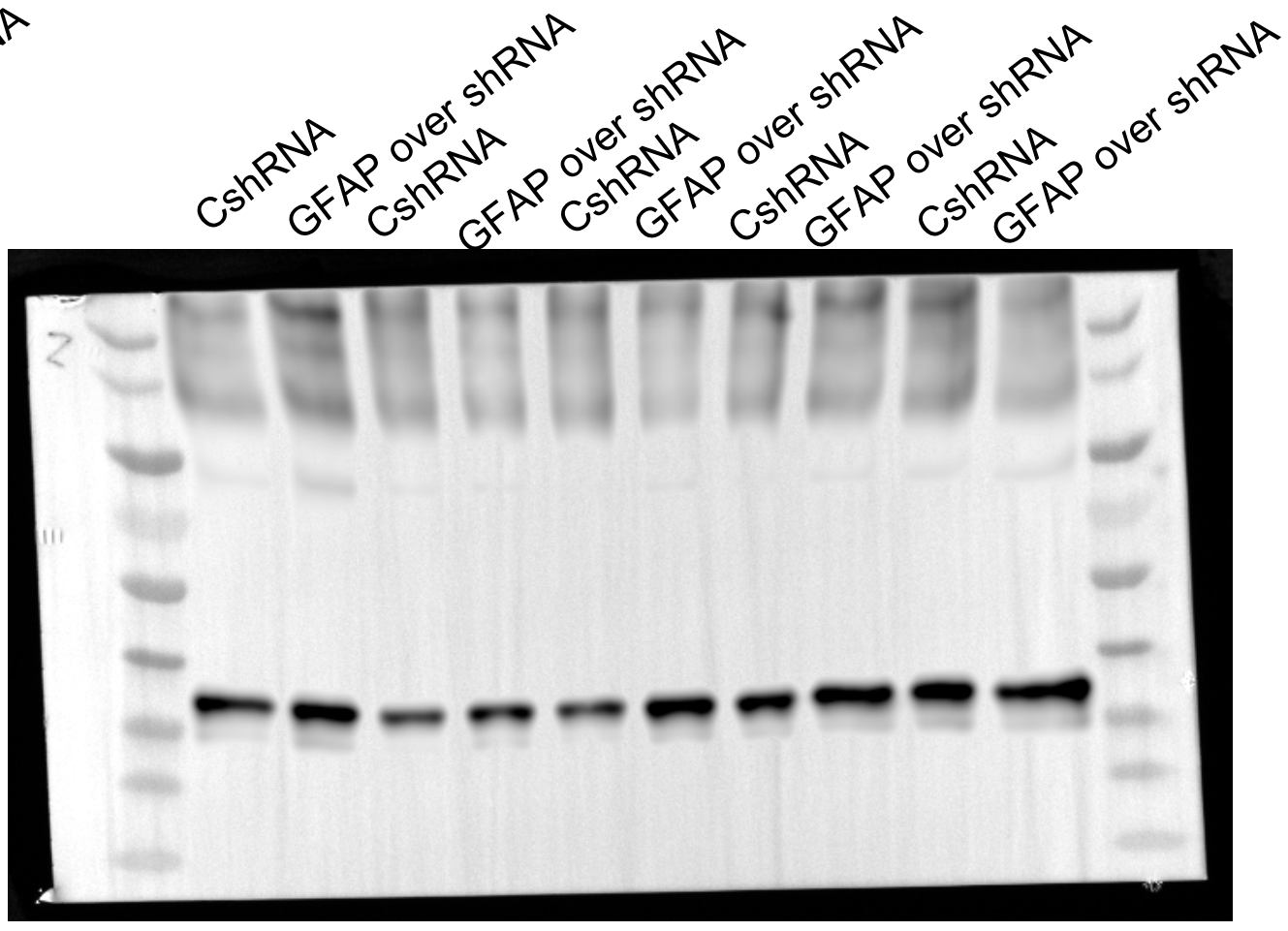
CshRNA
GFAP over shRNA
CshRNA
GFAP over shRNA
CshRNA
GFAP over shRNA
CshRNA
GFAP over shRNA
CshRNA
GFAP over shRNA

P2X7

GAPDH

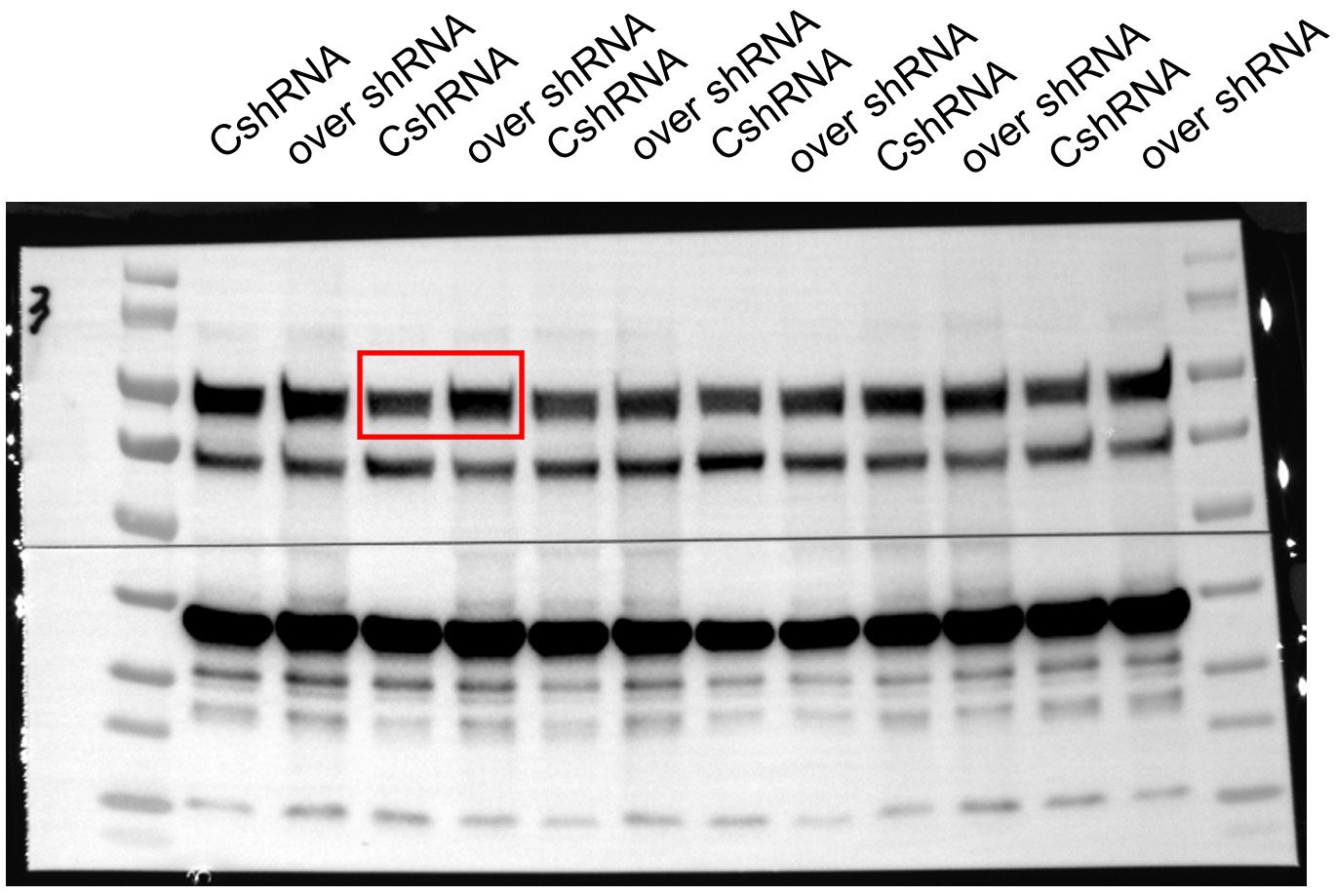
CshRNA
over shRNA
CshRNA
over shRNA
CshRNA
over shRNA
CshRNA
over shRNA
CshRNA
over shRNA
CshRNA
over shRNA
3

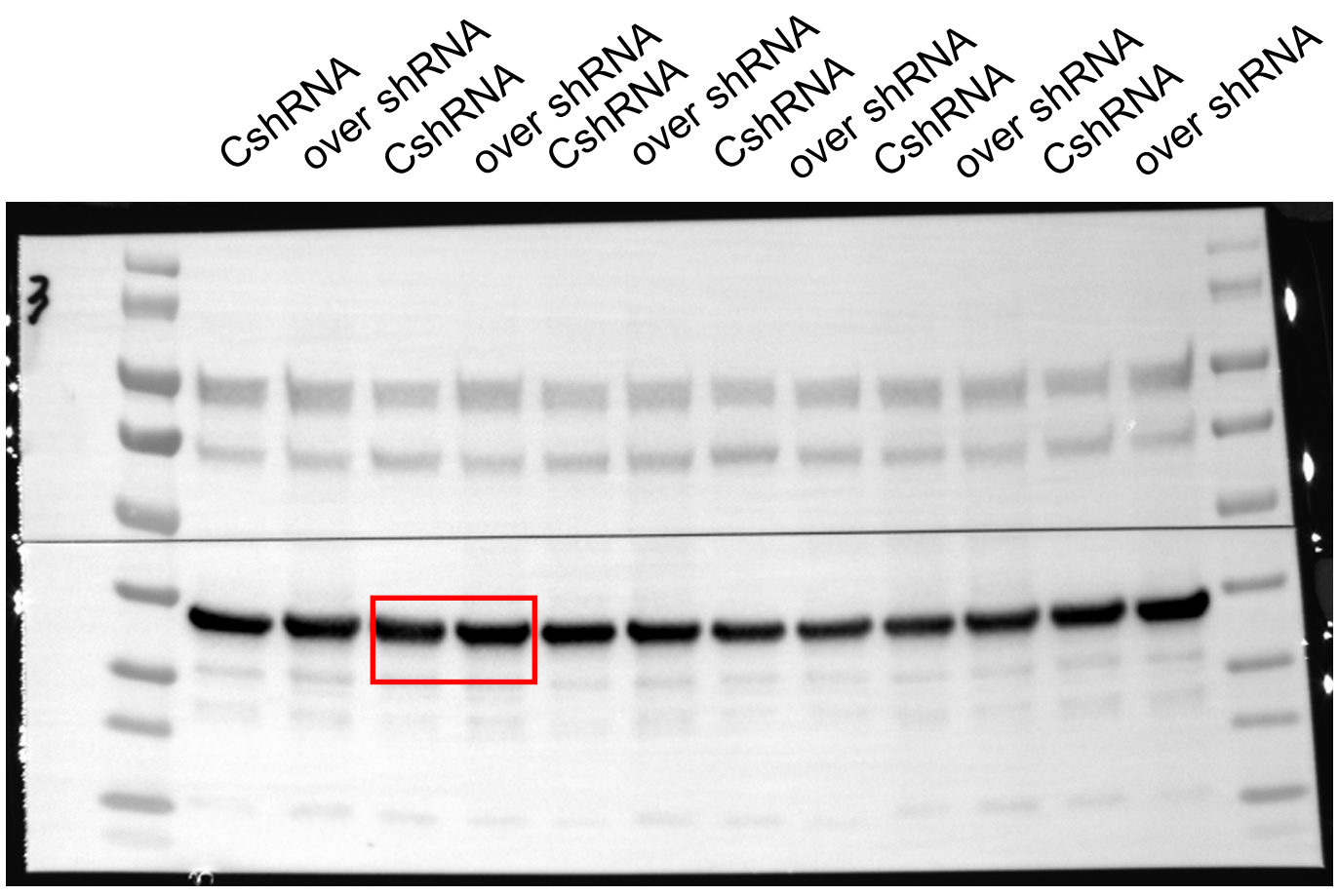
CshRNA
over shRNA
CshRNA
over shRNA
CshRNA
over shRNA
CshRNA
over shRNA
CshRNA
over shRNA
CshRNA
over shRNA
3

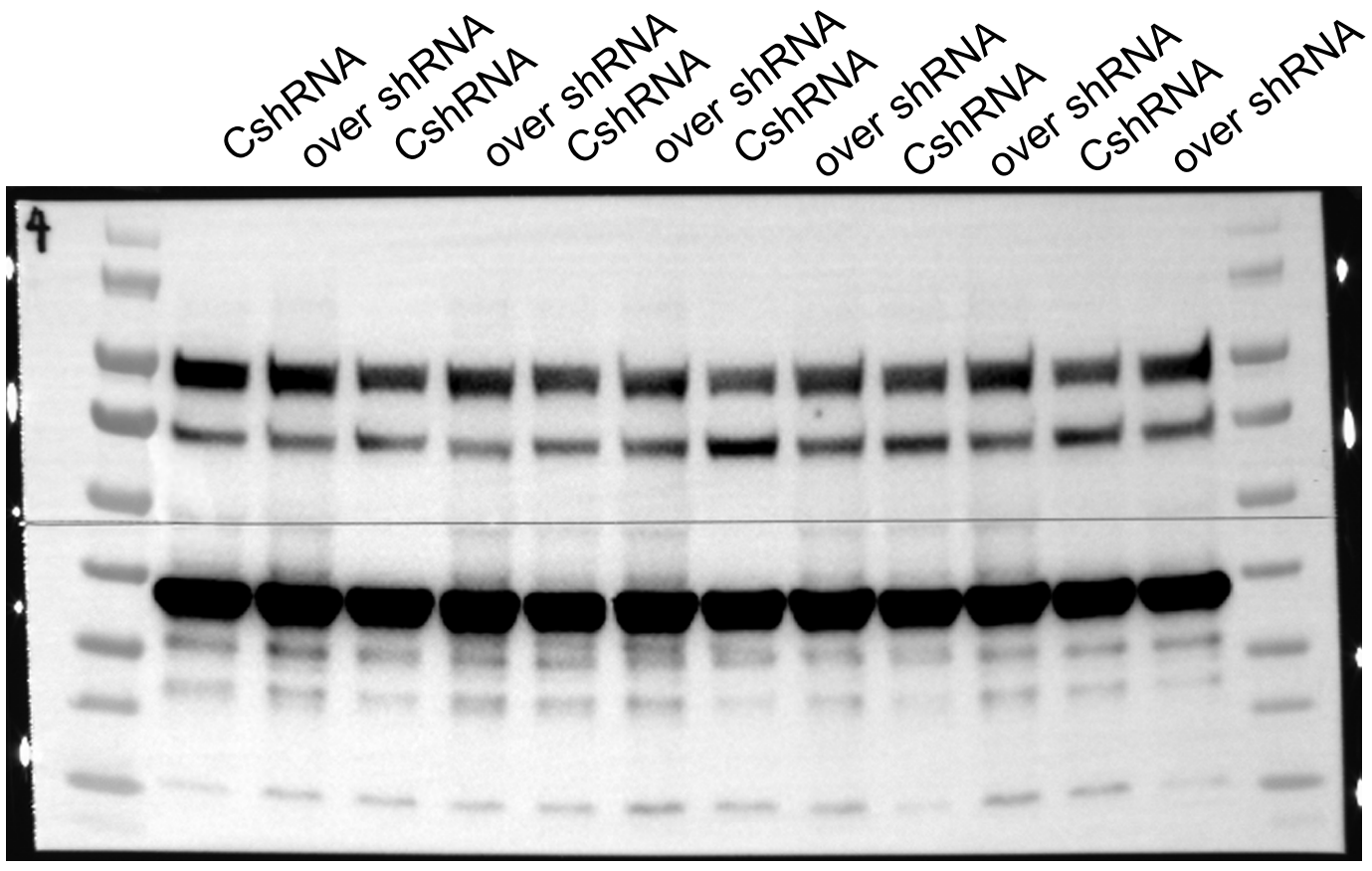
CshRNA
over shRNA
CshRNA
over shRNA
CshRNA
over shRNA
CshRNA
over shRNA
CshRNA
over shRNA
CshRNA
over shRNA
4

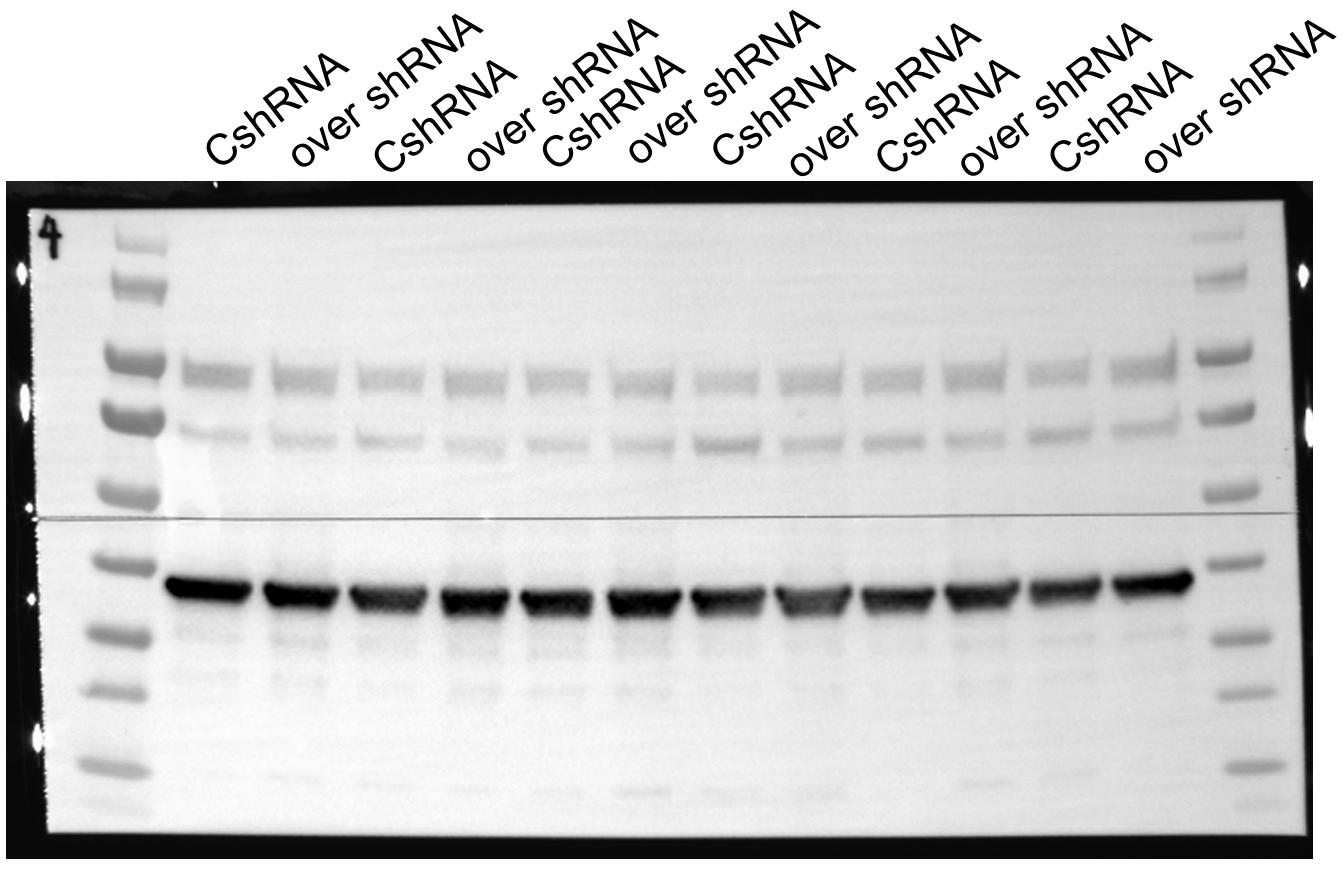
CshRNA
over shRNA
CshRNA
over shRNA
CshRNA
over shRNA
CshRNA
over shRNA
CshRNA
over shRNA
CshRNA
over shRNA
4

P2X7

GAPDH

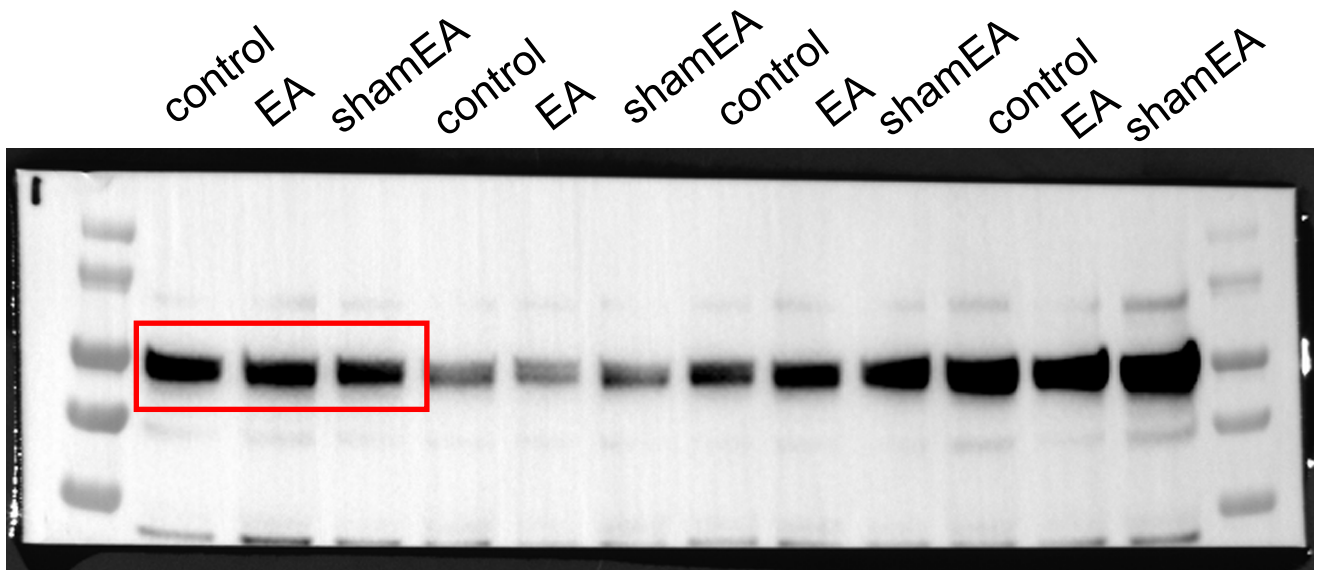
control
EA
shamEA
control
EA
shamEA
control
EA
shamEA
control
EA
shamEA

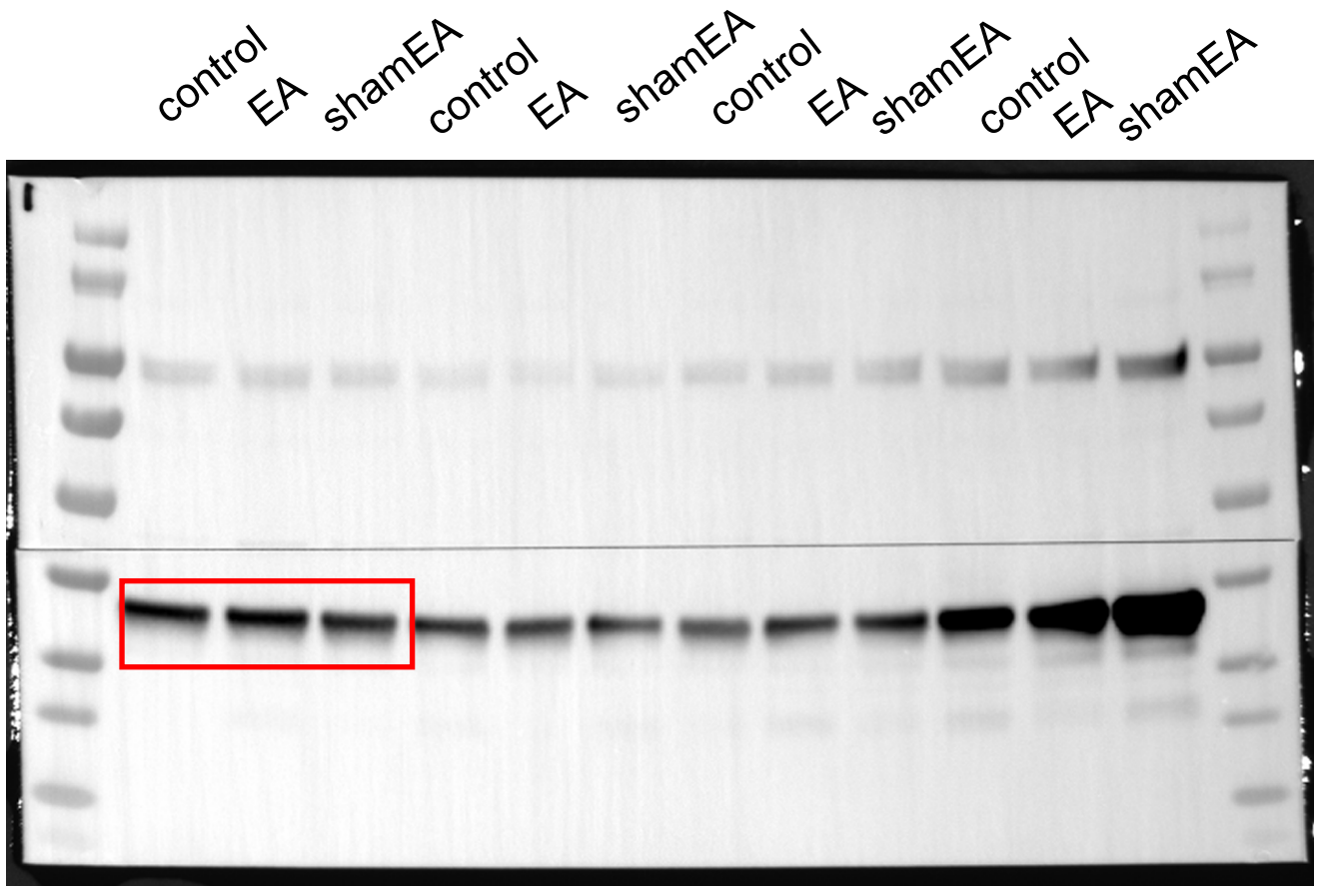
control
EA
shamEA
control
EA
shamEA
control
EA
shamEA
control
EA
shamEA

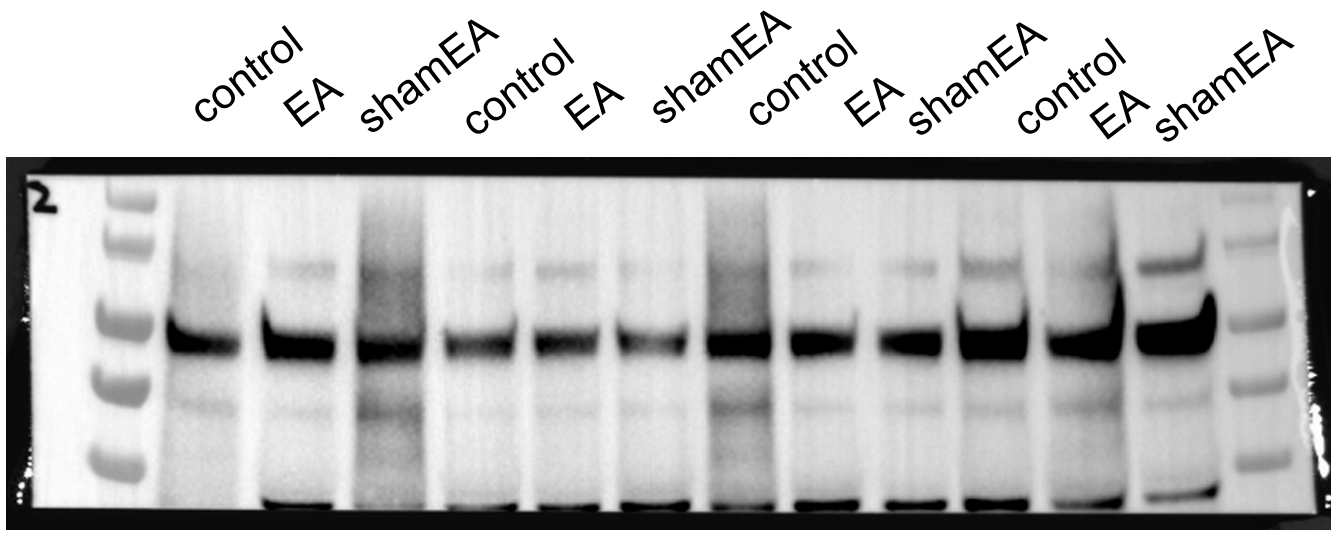
control
EA
shamEA
control
EA
shamEA
control
EA
shamEA
control
EA
shamEA

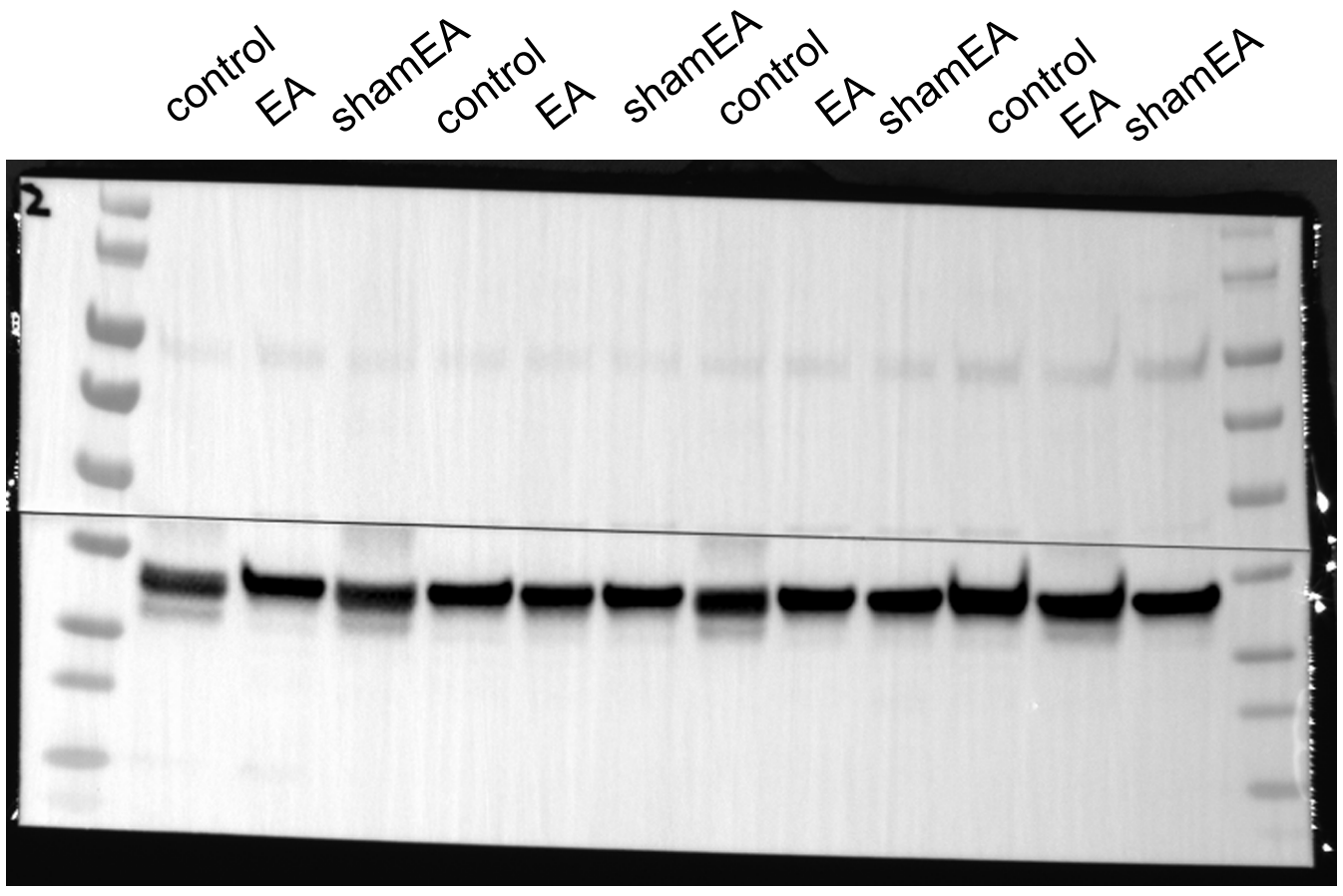
control
EA
shamEA
control
EA
shamEA
control
EA
shamEA
control
EA
shamEA
